# CBT treatment delivery formats for panic disorder: a systematic review and network meta-analysis of randomised controlled trials

**DOI:** 10.1017/S0033291722003683

**Published:** 2023-02

**Authors:** Davide Papola, Giovanni Ostuzzi, Federico Tedeschi, Chiara Gastaldon, Marianna Purgato, Cinzia Del Giovane, Alessandro Pompoli, Darin Pauley, Eirini Karyotaki, Marit Sijbrandij, Toshi A. Furukawa, Pim Cuijpers, Corrado Barbui

**Affiliations:** 1 Department of Neuroscience, Biomedicine and Movement Science, Section of Psychiatry, WHO Collaborating Centre for Research and Training in Mental Health and Service Evaluation, University of Verona, Verona, Italy; 2 Institute of Primary Health Care (BIHAM), University of Bern, Bern, Switzerland; 3 Psychiatric Rehabilitation Clinic Villa San Pietro, Trento, Italy; 4 Department of Clinical, Neuro and Developmental Psychology, WHO Collaborating Centre for Research and Dissemination of Psychological Interventions, Amsterdam Public Health Research Institute, Vrije Universiteit Amsterdam, Amsterdam, the Netherlands; 5 Departments of Health Promotion and Human Behavior, Kyoto University Graduate School of Medicine/School of Public Health, Kyoto, Japan

**Keywords:** CBT, cognitive-behavioural therapy, network meta-analysis, panic disorder, systematic review, treatment delivery formats

## Abstract

Several in-person and remote delivery formats of cognitive-behavioural therapy (CBT) for panic disorder are available, but up-to-date and comprehensive evidence on their comparative efficacy and acceptability is lacking. Our aim was to evaluate the comparative efficacy and acceptability of all CBT delivery formats to treat panic disorder. To answer our question we performed a systematic review and network meta-analysis of randomised controlled trials. We searched MEDLINE, Embase, PsycINFO, and CENTRAL, from inception to 1st January 2022. Pairwise and network meta-analyses were conducted using a random-effects model. Confidence in the evidence was assessed using Confidence in Network Meta-Analysis (CINeMA). The protocol was published in a peer-reviewed journal and in PROSPERO. We found a total of 74 trials with 6699 participants. Evidence suggests that face-to-face group [standardised mean differences (s.m.d.) −0.47, 95% confidence interval (CI) −0.87 to −0.07; CINeMA = moderate], face-to-face individual (s.m.d. −0.43, 95% CI −0.70 to −0.15; CINeMA = Moderate), and guided self-help (SMD −0.42, 95% CI −0.77 to −0.07; CINeMA = low), are superior to treatment as usual in terms of efficacy, whilst unguided self-help is not (SMD −0.21, 95% CI −0.58 to −0.16; CINeMA = low). In terms of acceptability (i.e. all-cause discontinuation from the trial) CBT delivery formats did not differ significantly from each other. Our findings are clear in that there are no efficacy differences between CBT delivered as guided self-help, or in the face-to-face individual or group format in the treatment of panic disorder. No CBT delivery format provided high confidence in the evidence at the CINeMA evaluation.

## Introduction

Cognitive-behavioural therapy (CBT) is the most viable and recommended psychological treatment for panic disorder (American Psychiatric Association, 2009; NICE, 2011; Papola et al., 2022). The last decades saw an important growth of demand for it (Kaczkurkin & Foa, 2015), an increase in waiting lists (Saunders & Allen, 2021), and consequently, pressure for more accessible and efficient forms of treatment delivery. On the other hand, the delivery of CBT may be hampered by difficulties in treatment delivery common to all forms of psychotherapies. The most frequent delivery hurdles producing gaps in the treatment of panic disorder are geographical barriers, impeding access for people living in rural areas where travelling time may be problematic and an added burden, people not living close to mental health services, commitments of people who work during the day, and only have time in the evenings (Webb, Rosso, & Rauch, 2017). Further issues are represented by financial constraints for patients and shortage of specialised CBT therapists, especially in poor resource settings (Lawton, McRae, & Gordon, 2021; Pabayo, Benny, Liu, Grinshteyn, & Muennig, 2022).

The difficulties are even greater for patients with panic disorder comorbid with agoraphobia, because they may not seek therapy due to fear of leaving their homes (Newman, Szkodny, Llera, & Przeworski, 2011). As a consequence, the development of delivery formats other than individual in-person face-to-face format has been a major challenge over the last decades, aiming to increase the accessibility and affordability of CBT, without losing efficacy, thus assisting the greatest number of people in the most efficient manner.

Furthermore, the coronavirus disease 2019 (COVID-19) outbreak gave an unprecedented momentum to the implementation of alternative treatment delivery formats in psychotherapy, as the great majority of therapists working face-to-face switched to remote synchronous delivery during the pandemic (Chevance et al., 2020; Wind, Rijkeboer, Andersson, & Riper, 2020). Such a drastic change in everyday clinical practice was forced by the contingencies and unfolded despite lack of structured knowledge on the comparative efficacy of treatment modalities other than the face-to-face individual format.

Pairwise meta-analyses have quantitatively summarised the results of trials that tested the efficacy of some of these different CBT delivery formats for depressive and anxiety disorders (Carlbring, Andersson, Cuijpers, Riper, & Hedman-Lagerlöf, 2018; Cuijpers, Donker, van Straten, Li, & Andersson, 2010; Okumura & Ichikura, 2014), but far less attention has been paid to panic disorder (Schwartze et al., 2017). Moreover, head-to-head comparisons are limited. Network meta-analysis (NMA) may overcome this limit by providing a global estimate of efficacy or safety of multiple interventions, including those that have not been directly compared before. NMA incorporates both direct and indirect effects, and allows to rank the treatments to identify which is the best or worst among them (White, 2015). Ranking CBT delivery formats based on their efficacy on panic symptomatology is of critical importance for the future of mental health care system resources, optimisation, and organisation. This especially holds true at the dawn of the post-corona era, since it is unclear how the pandemic will impact services and public mental health in the long-term (Saunders & Allen, 2021).

Against this background, we evaluated the comparative efficacy and acceptability of different types of CBT delivery formats for adults suffering from panic disorder with or without agoraphobia.

## Methods

This study report is written in accordance with the Preferred Reporting Items for Systematic Reviews and Meta-Analyses (PRISMA) guidelines specific for NMA (Page et al., 2021) (see also online Supplementary material, appendix A). The study protocol was registered with PROSPERO (CRD42020206258) and published in a peer reviewed journal (Papola et al., 2020a).

### Search strategy

We searched MEDLINE, Embase, PsycINFO and the Cochrane Register of Controlled Trials-CENTRAL, from database inception to the 1st January 2022, to identify randomised controlled trials (RCTs) examining the effects of psychotherapy for panic disorder with or without agoraphobia, compared with any other psychotherapy or control conditions (for the full search strategy, see online Supplementary material, appendix B). From this pool of RCTs we further selected only those studies testing different CBT delivery formats.

### Inclusion and exclusion criteria

Inclusion criteria were: (i) adults with a primary diagnosis of panic disorder with or without agoraphobia according to any standard operationalised criteria (Research Diagnostic Criteria, DSM up to the fifth version, ICD-10); (ii) the psychotherapeutic intervention had to be CBT, defined as a treatment that focuses on patients interoceptive fears and uses both cognitive restructuring and behavioural procedures to reduce those fears (Clark & Salkovskis, 2009; Craske & Barlow, 2006; Papola et al., 2020a, 2020b); (iii) CBT could be delivered by a therapist or as self-help. CBT and comparators were grouped into ten homogeneous groups that represented the ‘nodes’ of the network analysis: in-person face-to-face individual, remote synchronous face-to-face individual (videoconferencing or telephone), in-person face-to-face group, remote synchronous face-to-face group, remote guided self-help, remote unguided self-help, treatment as usual, waiting list, psychological placebo, placebo pill (online Supplementary material, appendix C).

### Study selection and data extraction

All records from all sources were entered into Endnote, and duplicates removed. Two independent researchers (DP and CG) checked all resulting records. If one of the researchers indicated a record possibly containing a study meeting the inclusion criteria, the full text of that paper was retrieved. The full texts were read by the same researchers for final inclusion.

In accordance with the study protocol, we worked in pairs (DP and CG, GO and MP) and independently extracted the following data from the original reports: mean age, percentage of women, percentage of agoraphobic participants in the trial, year of publication, study duration, treatment format, number of sessions of the treatment. We also rated presence/absence of pharmacotherapy co-administration, if a treatment manual was used by the therapist, if the psychotherapy was provided by a specially trained/supervised therapist, and if a treatment integrity procedure was carried out. Any discrepancies were resolved by consensus and arbitration by one of the senior authors (TAF, PC, or CB).

### Risk of bias assessment

We assessed the risk of bias of the included studies using the Cochrane ‘Risk of bias’ tool 2nd version for randomised trials (ROB 2) (Sterne et al., 2019). DP, CG, and MP independently used the ROB 2 signalling questions to form judgments on the five ROB 2 domains. Disagreements were resolved by discussion and consensus with a third author (TAF, PC, or CB).

### Outcomes

We measured efficacy in reducing panic symptoms (continuous outcome, indicated as ‘efficacy’) and all-cause discontinuation from the trial (binary outcome, indicated as ‘acceptability’). For the efficacy outcome, we selected one scale for each study using a pre-planned hierarchical algorithm, giving priority to scales specifically developed for panic disorder (online Supplementary material, appendix D) (Papola et al., 2020a, 2020b, 2022). All-cause discontinuation was measured as the proportion of participants who discontinued the trial for any reason. All outcomes referred to the acute phase treatment (study endpoint). For both outcomes, we produced a treatment hierarchy by means of surface under the cumulative ranking curve (SUCRA) and mean ranks, having treatment as usual as reference (Salanti, Ades, & Ioannidis, 2011). Participants allocated to the treatment as usual condition (condition also known as ‘standard of care’) received assessment only, with or without simple provision of informational material or minimal therapist contact or routine pharmacotherapy or all, knowing that they would not receive the active treatment in question after the trial. The participants in this condition were allowed to seek treatment as available in the community.

### Data analysis

We conducted a series of pairwise meta-analyses for all direct comparisons using a random-effects pooling model. For each outcome, we performed a NMA with a random-effects model, using the Stata *mvmeta* package. For the continuous outcome (efficacy) we pooled the standardised mean differences (SMDs) between treatment arms at endpoint. For the dichotomous outcome (acceptability), we calculated relative risks (RR) with a 95% confidence interval (CI) for each study. For continuous variables, we used intention-to-treat (ITT) data when available, and completers data when ITT data were not available. Dichotomous data were calculated on a strict ITT basis, considering the total number of randomised participants as denominator. Where participants had been excluded from the trial before the endpoint, we considered that they had a negative outcome by the end of the trial. When a study included different arms of a slightly different version of the same delivery method we pooled these arms into a single one (online Supplementary material, appendix E) (Higgins et al., 2019).

To assess the assumption of transitivity we compared the distribution of the following variables across set of interventions (percentage of women, mean age, percentage of agoraphobics, number of psychotherapy sessions, provision of psychotherapy by specifically trained therapists, treatment integrity verification, treatment manual guidance, type of outcome measure) (Veroniki et al., 2021). Furthermore, we performed meta-regression analyses on the same variables, to identify possible treatment effect moderators. We considered that distribution differences in specific study characteristics across the different set of interventions were only relevant in case of both significant imbalances according to (i) visual inspection of the distribution of variables across set of interventions (ii) the Kruskal–Wallis test (continuous variables), the Pearson χ^2^ or the Fisher exact test (categorical variables), and (iii) meta-regression analyses showing an actual impact on treatment effect.

The variance in the random-effects distribution (heterogeneity variance) was considered to measure the extent of cross-comparison and within-comparison variability of treatment effects in each network and was assessed by means of τ^2^ (low: τ^2^ ⩽ 0.010; moderate: 0.010 < τ^2^ ⩽ 0.242; high: τ^2^ > 0.242) (Huhn et al., 2019; Rhodes, Turner, & Higgins, 2015). We statistically evaluated the presence of incoherence by comparing direct and indirect evidence within each closed loop by using the Stata commands *mvmeta* and *ifplot* (Bucher, Guyatt, Griffith, & Walter, 1997) in the Stata network suite. Incoherence was further investigated through the side-splitting approach for each comparison (Palmer & Sterne, 2016). We asked trial authors to supply missing data or, alternatively, we imputed data using validated statistical methods (Higgins et al., 2019).

For the efficacy outcome, we conducted pre-planned sensitivity analyses excluding trials with imputed data, and excluding trials judged to be at ‘high risk of bias’ in case of high statistical heterogeneity (τ^2^ > 0.242) to explore the putative effects of the study quality assessed through the ROB 2 on heterogeneity (Papola et al., 2020a, 2020b). A further sensitivity analysis was conducted post-hoc to test whether the results could be influenced by the type of outcome hierarchy (online Supplementary Appendix D). In this sensitivity, measures of ‘panic frequency/severity’ were considered at the top of the hierarchy instead of being considered at the bottom. If ten or more studies were included in a direct pairwise comparison, we assessed publication bias by visually inspecting the funnel plot, testing for asymmetry with the Egger's regression test (Egger, Davey Smith, Schneider, & Minder, 1997; Sterne et al., 2011), and investigated possible reasons for funnel plot asymmetry (Chaimani, Higgins, Mavridis, Spyridonos, & Salanti, 2013).

We assessed the confidence in the body of evidence from NMA through the Confidence in Network Meta-Analysis (CINeMA) application (Nikolakopoulou et al., 2020), and we produced a treatment hierarchy by means of surface under the cumulative ranking curve (SUCRA) and mean ranks, having treatment as usual as reference (Salanti et al., 2011).

Statistical evaluations and production of network graphs and figures were done using the network and network graphs packages in STATA (version 16.1, s.e.) (Chaimani & Salanti, 2015). Appendix F in the online Supplementary material lists the differences between the original protocol and this report.

## Results

### Study selection and characteristics of studies

**Fig. 1.**
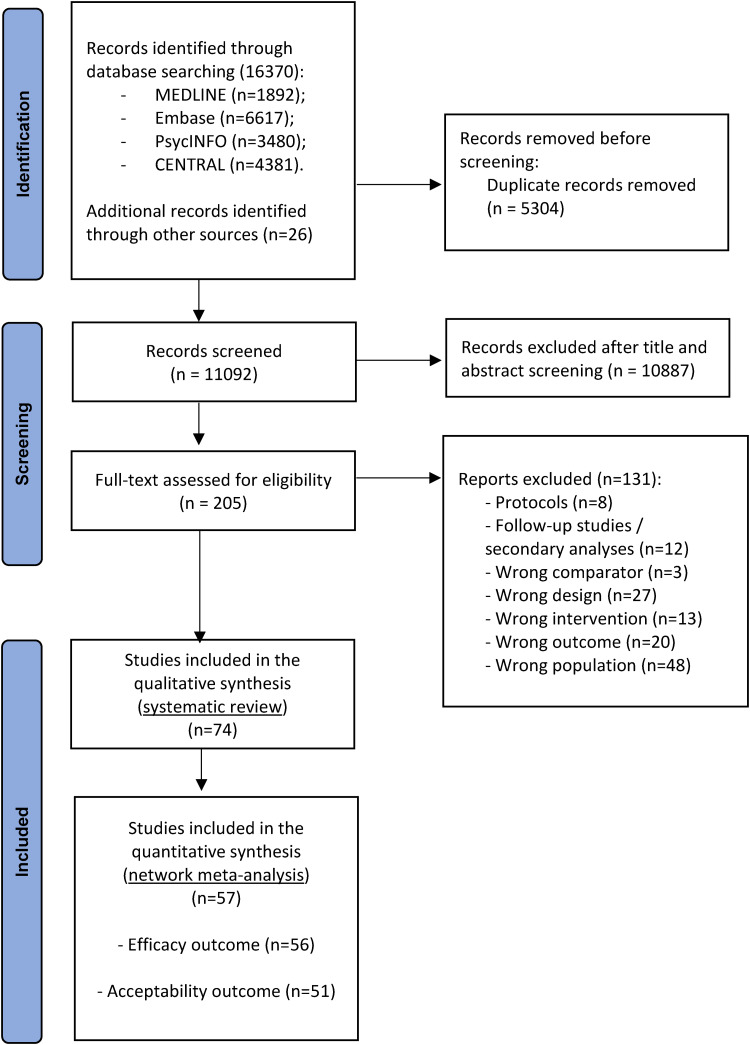
PRISMA flow diagram.

After examining a total of 16 396 titles and abstracts (11 092 after removal of duplicates), we retrieved 466 full-text articles for further consideration and excluded 392 articles (online Supplementary material appendix G and H). In total, 74 studies with 6699 patients met the inclusion criteria (see Fig. 1). Of these, 56 (76%) were eligible for the NMA (for the reasons that led to the exclusion of the 18 RCTs from the NMA see online Supplementary material, appendix E).

Participants were randomised to receive CBT in four different delivery formats: face-to-face individual, face-to-face group, guided self-help [all asynchronous but one (Ciuca, Berger, Crişan, & Miclea, 2018)], and unguided self-help; no trial delivered treatment through the individual or group remote synchronous modality. All guided self-help treatments consisted of CBT internet-based programs comprising learning modules with or without introductory and relapse prevention additional modules.

Table 1 shows selected characteristics of the included studies. Seventy-seven per cent of participants suffered from panic disorder associated with agoraphobia. The mean age was 37.3 years. The mean proportion of included women was 69.4%. All studies enroled adults between 18 and 65 years of age, with only one study including older adults (i.e. ⩾ 65 years) (Hendriks et al., 2010). Studies were distributed over 31 years (1989–2020) and generally had their main endpoint evaluation around the 12th week of treatment (range: 1 to 24). The mean number of therapy sessions was approximately 10 (range: 1 to 19). Most participants were receiving medications during the treatment period: 68 RCTs (91.9% of the total) allowed various psychotropic drugs to be taken on top of the experimental and control treatments. However, the great majority of the RCTs enroled participants only if they had been on a stable dosage for at least 1–3 months, and upon agreement to keep the dosages constant throughout the treatment period. Most treatments were implemented following the guidance of a manual (72 RCTs, 97.3%) and were delivered by licensed or specifically trained and supervised therapists (61 RCTs, 82.4%) but treatment integrity was verified in only 25 studies (33.8%) (online Supplementary material appendix I). Thirty-nine studies (70.9%) employed scales specifically designed to capture panic disorder symptoms.

**Table 1.** Characteristics of randomised controlled trials included in the systematic review and in each network of primary outcomes

	Systematic review	Network meta-analysis			
Characteristic		Efficacy network		Acceptability network	
Number of studies	74		55		50	
Number of patients included	6699		4915		4303	
Women %	69.4		70.4		70.6	
Mean age (years)	37.3		37.7		37.8	
Agoraphobics %	77.6		76.1		74.9	
	*N*	%	*N*	%	*N*	%
Year of publication						
1989–2000	26	35.1	18	32.7	15	30
2001–2010	33	44.6	25	45.5	23	46
2011–2020	15	20.3	12	21.8	12	24
Study duration						
1 to 12 weeks	25	33.8	19	34.5	18	36
13 to 24 weeks	10	13.5	9	16.4	8	16
Unclear or not reported	39	52.7	27	49.1	24	48
*N* sessions/modules						
1–6	15	20.3	13	23.6	12	24
7–12	45	60.8	33	60	30	60
13–19	12	16.2	9	16.4	7	14
Unclear	2	2.7	–	–	–	–
Pharmacotherapy allowed besides the experimental treatment						
Yes	68	91.9	51	92.7	47	94
No	5	6.7	4	7.3	3	6
Unclear	1	1.4	–	–	–	–
Was a treatment manual used?^a^						
Yes	72	97.3	53	96.4	49	98
Unclear	2	2.7	2	3.6	1	2
Provision of psychotherapy by specially trained and/or supervised therapists?						
Yes	61	82.4	46	83.64	41	82
Unclear	6	8.2	5	9	4	8
Not applicable	7	9.4	4	7.3	5	10
Was treatment integrity verified?						
Yes	25	33.8	19	45.5	18	36
No	34	45.9	25	34.5	20	40
Not applicable	15	20.1	11	20	12	24
Risk of bias						
High	40	54.2	28	50.9	24	48
Some concerns	25	33.7	18	32.7	18	36
Low	9	12.1	9	16.4	8	16
Type of outcome scale						
Scales specifically focused on panic disorder	NA	NA	43	78.2	NA	NA
Global symptoms scales	NA	NA	12	21.8	NA	NA
Scales specifically focused on agoraphobia	NA	NA	–	–	NA	NA
Scales for panic attacks only	NA	NA	–	–	NA	NA

NA, not applicable.

a Self-help interventions were automatically counted among trials using a treatment manual.

### Risk of bias of included studies

In most cases (40 RCTs, 54.2%) studies were considered to be at overall high risk of bias, for 25 (33.7%) studies there were ‘some concerns’, and 9 (12.1%) were judged to be at low risk of bias (online Supplementary material appendix J). The majority of the studies missed to adequately report the randomisation process, leading to ‘some concerns’ judgment in 58 studies (78.3%), 38 studies (51.5%) were judged to be at low risk of deviation from the intended interventions, for 45 (60.8%) there were no concerns of missing outcome data, 32 (43.2%) had low risk of bias in the measurement of the outcome, but only 7 (9.4%) were identified as having a low risk of bias in the selection of reported results.

### Network plot

**Fig. 2.**
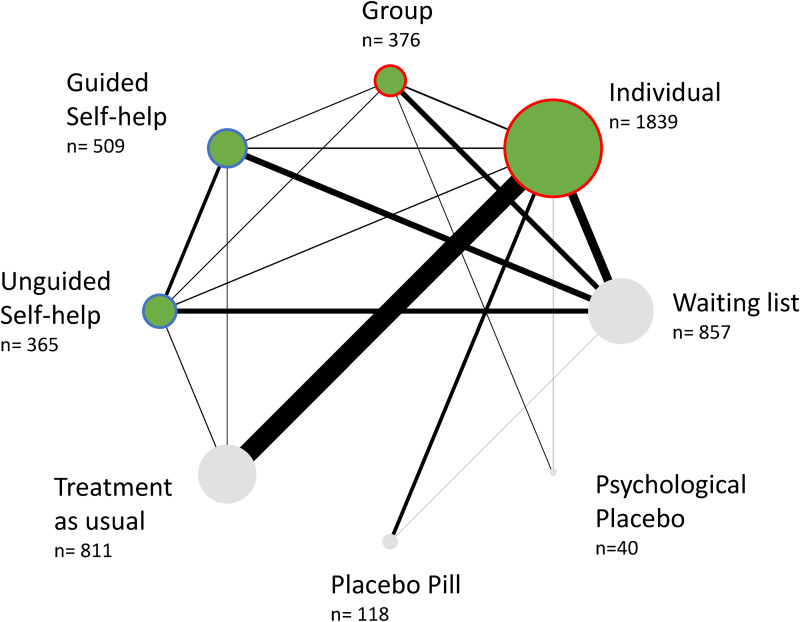
Network plot of evidence. The thickness of lines is proportional to the precision of each direct estimate and the size of circles is proportional to the number of participant randomised to that treatment. The *N* indicates the number of participants who were randomly assigned to each delivery format. Delivery formats are represented as green nodes, while controls are in grey. The nodes are circled in red where the CBT was delivered ‘in-presence face-to-face’, and in blue if the delivery was ‘by remote’. No trial delivered treatment through the individual or group remote synchronous modality.

Overall, the network was well-connected, and every treatment or control condition was included at least in one closed loop (see Fig. 2). The most examined comparisons were between individual, group, and guided self-help formats as well as the waiting list and treatment as usual control conditions. We detected a scarcity of direct comparisons between individual, group, and guided self-help CBT. Placebo and psychological placebo were weakly connected to the network, with few trials connecting them with individual CBT, group CBT and waiting list only.

**Fig. 3.**
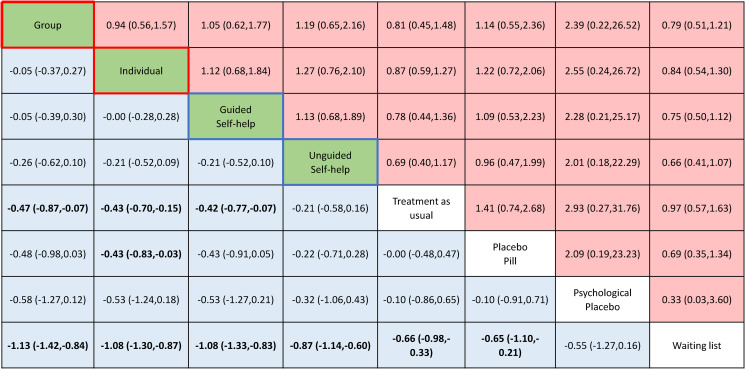
Net league table of head-to-head comparisons. Red-circled green cells = interventions delivered ‘in-presence face-to-face’. Blue-circled green cells = interventions delivered ‘by remote’. White = controls. Light blue = efficacy. Standardised mean differences (s.m.d.s) and 95% confidence intervals (CIs) are reported. s.m.d.s lower than 0 favour the column-defining treatment. Light red = acceptability. Relative risks (RRs) and 95% confidence intervals are reported. RRs lower than 1 favour the column-defining treatment. 95% CIs not including the point of no difference (0) are in boldface.

Figure 3 shows the results of the NMAs for each CBT treatment delivery format in the form of a net league table. We found no evidence of violations of the transitivity assumption when assessing the distribution of the distribution of effect modifiers across comparisons (online Supplementary material appendix K). Of the 74 studies included in the systematic review, 57 (74%, 5638 participants), provided data for at least one outcome (see Fig. 1; online Supplementary material appendix E). For each network estimate, all standard pairwise meta-analyses, NMAs, and assessments of heterogeneity, incoherence and quality of evidence are reported in the online Supplementary material appendix L and M.

### Efficacy outcome

**Fig. 4.**
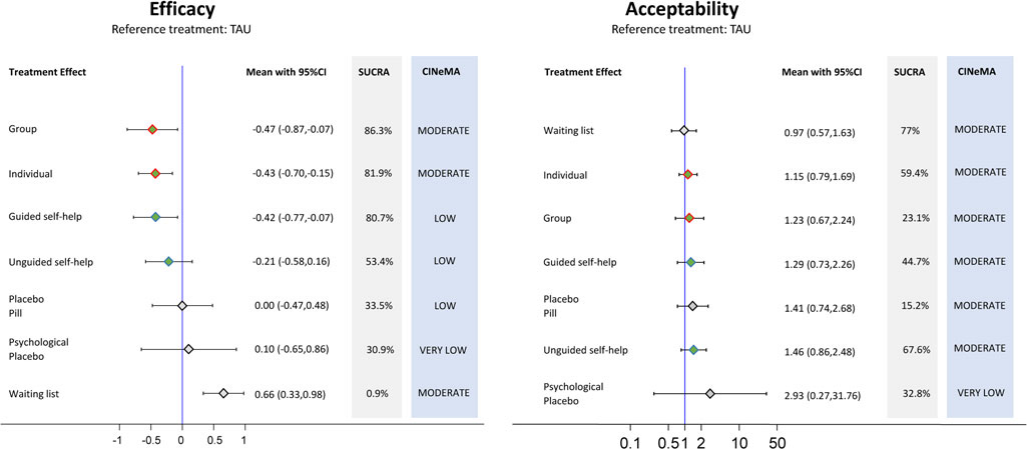
Forest plots comparing each psychotherapy with treatment as usual for efficacy and acceptability with the corresponding ranking probability (SUCRA) and certainty of evidence (CINeMA), as assessed with the CINeMA appraisal, for each intervention. Point estimates are green or grey to signal interventions or controls. Point estimates are red or blue-circled to signal ‘in-presence face-to-face’ or ‘remote’ delivery modalities, respectively. Controls are circled in black. CI, confidence interval; CINeMA, Confidence in Network Meta-Analysis; SUCRA, surface under the cumulative ranking; TAU, treatment as usual.

Group (s.m.d. −0.47; 95% CI −0.87 to −0.07; moderate confidence), Individual (s.m.d. −0.43; 95% CI −0.70 to −0.15; moderate confidence), and guided self-help CBT (s.m.d. −0.42; 95% CI −0.77 to −0.07; low confidence) were superior to treatment as usual (reference) and waiting list (s.m.d. 0.66; 95% CI 0.33–0.98; moderate confidence) in relieving the symptoms of panic disorder (see Figs 3 and 4). Unguided self-help was not superior to treatment as usual (s.m.d. −0.21; 95% CI −0.58 to 0.16). No significant differences in terms of efficacy between group, individual, guided self-help, and unguided self-help CBT were found. All CBT delivery formats were more efficacious than waiting list (see Figs 3 and 4).

Heterogeneity emerged from the network analysis (*τ*^2^ = 0.36), but the design-by-treatment interaction model indicated global coherence in the network (χ^2^ = 15.97; *p* = 0.59). Intraloop incoherence was found for only one out of 15 loops (‘individual – group – psychological placebo’), a proportion to be expected empirically (Veroniki, Vasiliadis, Higgins, & Salanti, 2013). No other incoherence factor was found. There was perfect consistency between direct and indirect estimates, as investigated through the *sidesplit all* STATA command. Regarding the confidence in the quality of evidence assessed through CINeMA, we did not rate any of the comparisons as ‘high confidence’, mainly because of within-study bias. Confidence in the estimate was moderate to low, with very low confidence only for some indirect comparisons. We identified just one comparison featuring more than ten studies (‘waiting list’ *v.* ‘individual’: 14 RCTs); both the visual inspection of the funnel plot and the Egger's test (*p* = 0.75) were negative for small study effects. Details for each analysis can be found in the online Supplementary material, appendix L.

### Acceptability outcome

No significant differences were found between different delivery formats, between delivery formats and comparators, and between comparators for the acceptability outcome. The network proved to be slightly heterogeneous (*τ*^2^ = 0.30; *p* = 0.034), but no incoherence was found in any of the 14 triangular loops investigated, and the design-by-treatment interaction model indicated global coherence in the network (χ^2^ = 16.13; *p* = 0.51). There was full consistency between all direct and indirect estimates as investigated through the sidesplit approach. No comparison gained the rating of ‘high’ confidence in the estimate. Most comparisons were rated as ‘moderate’ confidence. We identified just one comparison featuring more than ten studies (‘waiting list’ *v.* ‘individual’: 13 RCTs); both the visual inspection of the funnel plot and the Egger's test (*p* = 0.98) reassured against the possibility of small study effects. Details for each analysis can be found in the online Supplementary material, appendix M.

### Sensitivity analysis

Sensitivity showed a minimal decrease of heterogeneity after removing high risk of bias studies (*τ*^2^ = 0.30; *p* < 0.01) and studies for which we imputed data (*τ*^2^ = 0.38; *p* < 0.01), but a slight increase was noted when the data were analysed according to the modified outcome hierarchy (*τ*^2^ = 0.39; *p* < 0.01). Local and global inconsistency remained not significant for all sensitivity analyses. Although the exclusion of the 29 RCTs (51.4%) judged to be at high risk of bias led to a generalised deflation of the estimates, the positioning of the treatment delivery format did not change in terms of ranking. Same applies to the second sensitivity analysis where we explored the effects of the exclusion of imputed data on the efficacy outcome. After removing the four RCTs (7.1%) for which we imputed data only group and individual CBT retained its superiority over treatment as usual, but the network nodes retained their ranking as for the primary analysis. The treatment delivery format ranking did not change when data were analysed considering the modified outcome hierarchy – i.e. placing ‘panic frequency/severity’ measures at the top of the hierarchy (online Supplementary material, appendix N).

## Discussion

Findings from this NMA show that group, guided self-help and individual CBT delivery formats are all superior to treatment as usual having similar effect sizes, and no relevant differences emerged when they were compared head-to-head. On the other hand, CBT delivered as unguided self-help was not superior to treatment as usual. CBT delivered in any format was consistently as accepted as treatment as usual in terms of overall trial dropout rates. Our results are in line with those found by Cuijpers et al. for depression, with similar large-to-very large effect sizes found for CBT in different delivery formats against waiting list. Effect sizes of CBT against treatment as usual for the group and individual delivery formats are slightly smaller in comparison with those found by Cuijpers for the same delivery formats in depression (Cuijpers, Noma, Karyotaki, Cipriani, & Furukawa, 2019). This could be either due to random error, as a consequence of the different number of trials analysed in the two investigations (Cuijpers et al.: 155 RCTs, present investigation: 55 RCTs) or to effective differences in responding to individual or group CBT for people suffering from depression or panic disorder.

Guided self-help treatments are web-based programs enhanced by minimal, but regular, therapist support. Even if less than the individual and group delivery format, guided self-help treatments still require contact with a trained professional. On the other hand, the professional engagement required to nudge the patient toward the end of the self-help protocol is far lower in comparison with the necessity of professional commitment in case of face-to-face therapies. Asynchronous communications (emails or text messages) were the most commonly used and, on average, patient guidance did not take more than 10 min per week. So far, research on guided self-help treatments that include guidance through synchronous audio-video communication (real-time via systems such as Zoom or Skype) for panic disorder relies on a single RCT, which suggests the superiority of guided *v.* unguided self-help (Ciuca et al., 2018). At the same time, there is growing evidence that the efficacy of psychotherapy delivered completely via videoconference is equivalent to face-to-face treatment for mood and anxiety disorders (Germain, Marchand, Bouchard, Drouin, & Guay, 2009; Hilty et al., 2013; Stubbings, Rees, Roberts, & Kane, 2013; Théberge-Lapointe, Marchand, Langlois, Gosselin, & Watts, 2015), and although preliminary evidence is encouraging (Bouchard et al., 2004, 2022), to date there is no randomised evidence for the individual or group remote synchronous CBT treatment of panic disorder. The high prevalence of panic disorder and the ubiquity of internet access make guided self-help a cost-effective option to reduce the burden of disability associated with panic and agoraphobia. This holds true especially for poor resource settings, where a limited budget for mental health, poor access to services and limited infrastructure, as well as the small number of available mental health professionals contribute to huge treatment gaps (Bockting, Williams, Carswell, & Grech, 2016; Papola et al., 2020b). Guided self-help interventions can be also delivered via telephone or other media, such as a book; in our review they were all administered through the internet.

So far, several meta-analyses and reviews have suggested the superiority of guided over unguided self-help treatments in terms of efficacy for several mental health disorders (Andersson & Titov, 2014; Cuijpers et al., 2019; Palmqvist, Carlbring, & Andersson, 2007; Pauley, Cuijpers, Papola, Miguel, & Karyotaki, 2021). Our NMA can only partially confirm these results, as we found no ultimate evidence of the superiority of any treatment format over another nor in the network estimates neither when the different delivery formats were compared head-to-head in pairwise meta-analyses. Interestingly, unguided self-help is the only delivery format being not superior to treatment as usual, but not inferior to the other delivery methods at the same time.

There are two possible explanations for the absence of superiority of unguided self-help over treatment as usual. First, as unguided self-help treatments are those in which the user has to work through the material without any personal support, it is possible to indirectly identify the role of human contact and therapeutic alliance as key to improve rates of compliance. A fruitful patient–therapist relationship can be realised even with minimal contact with the therapist (Cuijpers et al., 2010), and working totally on its own the patient misses both the opportunity to benefit from the four Frank's ‘nonspecific factors’ (Frank & Frank, 1991) and to be properly involved in the so called ‘contextual model’ outlined by Wampold (Wampold, 2015). Second, variability exists between different CBT protocols, particularly for what concerns the different types of behavioural activities that can be performed during the therapy. It is known, for example, that specific CBT components such as interoceptive or exteroceptive exposure and cognitive restructuring influence positive outcomes more than other components, for e.g. breathing retraining, psychoeducation, or muscle relaxation (which has even shown to worsen the outcomes) (Craske, Rowe, Lewin, & Noriega-Dimitri, 1997; Pompoli et al., 2018; Schmidt et al., 2000; Siev & Chambless, 2007). It is possible that certain components are more prevalent or usable in certain delivery formats, thus reflecting differences more related to the blend of CBT components than to the way CBT itself is delivered.

Our findings may inform clinical practice and policy. Current guidelines on panic disorder (American Psychiatric Association, 2009; Katzman et al., 2014; NICE, 2011; Royal Australian and New Zealand College of Psychiatrists Clinical Practice Guidelines Team for Panic Disorder and Agoraphobia, 2003) lack specific indications on treatment delivery strategies and should be updated according to the following key findings. First, with the same efficacy and acceptability of CBT delivered through the group or the individual format, guided self-help CBT should be viewed as a first line option for the treatment of panic disorder, with the goal of reducing personal, social, and monetary costs, and expanding the accessibility of treatments (Fairburn & Patel, 2017; Kazdin & Blase, 2011). Second, guidelines should highlight the need to routinely implement a shared-decision making framework that considers both the preferences and the clinical situation of the patient, promoting the use of guided self-help protocols for patients that may have difficulties in leaving home or have transportation or other personal difficulties that hamper the possibility to perform face-to-face sessions. Finally, our findings might encourage policy makers to consider the implementation of a stepped care approach in the clinical routine, where people are first offered a potentially highly available, flexible and low-cost option (guided self-help programs) followed by more intensive and structured therapies (face-to-face or drug therapy) only if the first step is unsuccessful.

### Limitations and strength

Results should be interpreted in light of some potential limitations. First, the included studies were published over a long timespan, enroled different ranges of agoraphobic people, had different durations and administered more or less intensive CBT protocols in terms of number of sessions/time. This has inevitably introduced heterogeneity in terms of design, diagnostic criteria, and outcomes, which could not be explained by the preplanned sensitivity analyses. At the same time, transitivity was well preserved. Second, risk of bias was judged as high in 54% of the studies included in the systematic review. This finding may be explained by the requirements of the ROB 2 tool that we have implemented. Some of the key domains needed to grant a low risk of bias status are rarely satisfied for psychotherapy intervention trials, especially the older ones. For example, trials implementing per-protocol analyses, scarcity of details on the allocation concealment process, and the absence of a pre-planned statistical protocol had a considerable impact on the overall risk of bias assessment. As a direct consequence, the risk of bias evaluation fell back on the CINeMA assessment with no CBT treatment delivery format proving to have high confidence. As a confirmation of the robustness of the results, when we performed a sensitivity analysis without high risk of bias studies, the treatment delivery format ranking did not change, although the exclusion of 28 (50.9%) high risk of bias RCTs led to a generalised deflation of the estimates and widening of CIs. CBT, as delivered in any format, lost its superiority over treatment as usual with CIs including a possibility of benefit of the control treatment as compared to the interventions. A similar statistical artefact happened when we excluded RCTs with imputed data. Third, as no studies directly compared group CBT with treatment as usual, the position of group CBT in the rank order compared to treatment as usual is established through indirect evidence. There is the need for future studies to address this relevant knowledge gap. Fourth, although there is growing evidence that the efficacy of psychotherapy delivered completely via videoconference is equivalent to in-person face-to-face treatment for mood and anxiety disorders (Germain et al., 2009; Hilty et al., 2013; Stubbings et al., 2013; Théberge-Lapointe et al., 2015), and preliminary evidence is encouraging (Bouchard et al., 2004, 2022), we found no randomised evidence comparing the in-person and remote synchronous CBT delivery treatment formats for panic disorder.

Despite these limitations, this NMA is the first to compare alternative delivery formats at the same time and on a common metric for a specific anxiety disorder, taking advantage of all direct and indirect comparisons simultaneously, thus making the estimates more precise and consistent than previous pairwise meta-analyses (Carlbring et al., 2018; Cuijpers et al., 2010; Okumura & Ichikura, 2014). Results are coherent both at the global and local (loop) level, the transitivity assumption was preserved, and we gave priority to panic-specific scales according to a pre-planned scales hierarchy, thus enhancing the clinical reliability of the results. Finally, to minimise research waste and improving research usability we provided online Supplementary information with extensive description of interventions (Hoffmann et al., 2017).

## Conclusion

Although no CBT delivery format provided high confidence in the evidence at the CINeMA evaluation our findings suggest that there are no significant differences in terms of efficacy or acceptability when CBT is delivered via the face-to-face group, face-to-face individual, or guided self-help format, indicating that the three treatment formats are equally more effective over treatment as usual. Component analyses and further randomised studies are warranted to better clarify the role of unguided self-help protocols in treating panic disorder.

## Supplementary material

For supplementary material accompanying this paper visit https://doi.org/10.1017/S0033291722003683. click here to view supplementary material
