# Supplementary material for: CBT treatment delivery formats for panic disorder: a systematic review and network meta-analysis of randomised controlled trials
Source: Psychol Med. 2022 Dec 9;53(3):614–24. doi: 10.1017/S0033291722003683 (PMC9975966; doi:10.1017/S0033291722003683)
Supplement: Supplementary file 1 [file S0033291722003683sup001.doc]

**CBT treatment delivery formats for panic disorder: a systematic review and network meta-analysis of randomized controlled trials**

Papola et al.

**Supplementary material**

| **Appendix** |  | PRISMA NMA checklist | p. 2 |
| --- | --- | --- | --- |
| **Appendix** |  | Search strategy | p. 4 |
| **Appendix** |  | Definitions of type of CBT delivery formats and controls | p. 9 |
| **Appendix** |  | Hierarchy of symptom severity measurement scales | p. 10 |
| **Appendix** |  | Characteristics of the included studies | p. 11 |
| **Appendix** |  | Differences between protocol and review | p. 19 |
| **Appendix** |  | Trials included in the systematic review | p. 20 |
| **Appendix** |  | Trials excluded from the systematic review, with reasons | p. 24 |
| **Appendix** |  | Characteristics of the CBT interventions | p. 33 |
| **Appendix** |  | Risk of bias of the included studies | p. 42 |
| **Appendix** |  | Transitivity assessment and meta-regressions | p. 126 |
| **Appendix** | **L** | Primary outcome: efficacy | p. 131 |
| **Appendix** | **M** | Primary outcome: acceptability | p. 140 |
| **Appendix** | **N** | Sensitivity analyses | p. 148 |
|  |  |  |  |

**Appendix A - PRISMA NMA checklist**

| **Section/Topic** | **Item #** | **Checklist Item** | **Reported on Page #** |
| --- | --- | --- | --- |
| **TITLE** |  |  |  |
| Title | 1 | Identify the report as a systematic review *incorporating a network meta-analysis (or related form of meta-analysis).* | 1 |
|  |  |  |  |
| **ABSTRACT** |  |  |  |
| Structured summary | 2 | Provide a structured summary including, as applicable:  **Background:** main objectives  **Methods:** data sources; study eligibility criteria, participants, and interventions; study appraisal; and *synthesis methods, such as network meta-analysis.*  **Results:** number of studies and participants identified; summary estimates with corresponding confidence/credible intervals; *treatment rankings may also be discussed. Authors may choose to summarize pairwise comparisons against a chosen treatment included in their analyses for brevity.*  **Discussion/Conclusions:** limitations; conclusions and implications of findings.  **Other:** primary source of funding; systematic review registration number with registry name. | 2 |
|  |  |  |  |
| **INTRODUCTION** |  |  |  |
| Rationale | 3 | Describe the rationale for the review in the context of what is already known*, including mention of why a network meta-analysis has been conducted.* | 3 |
| Objectives | 4 | Provide an explicit statement of questions being addressed, with reference to participants, interventions, comparisons, outcomes, and study design (PICOS). | 3 |
|  |  |  |  |
| **METHODS** |  |  |  |
| Protocol and registration | 5 | Indicate whether a review protocol exists and if and where it can be accessed (e.g., Web address); and, if available, provide registration information, including registration number. | 4 |
| Eligibility criteria | 6 | Specify study characteristics (e.g., PICOS, length of follow-up) and report characteristics (e.g., years considered, language, publication status) used as criteria for eligibility, giving rationale. *Clearly describe eligible treatments included in the treatment network, and note whether any have been clustered or merged into the same node (with justification).* | 4,5 |
| Information sources | 7 | Describe all information sources (e.g., databases with dates of coverage, contact with study authors to identify additional studies) in the search and date last searched. | 4 |
| Search | 8 | Present full electronic search strategy for at least one database, including any limits used, such that it could be repeated. | Appendix B |
| Study selection | 9 | State the process for selecting studies (i.e., screening, eligibility, included in systematic review, and, if applicable, included in the meta-analysis). | 5 |
| Data collection process | 10 | Describe method of data extraction from reports (e.g., piloted forms, independently, in duplicate) and any processes for obtaining and confirming data from investigators. | 5 |
| Data items | 11 | List and define all variables for which data were sought (e.g., PICOS, funding sources) and any assumptions and simplifications made. | 5, Appendix E |
| **Geometry of the network** | **S1** | Describe methods used to explore the geometry of the treatment network under study and potential biases related to it. This should include how the evidence base has been graphically summarized for presentation, and what characteristics were compiled and used to describe the evidence base to readers. | 6,7 |
| Risk of bias within individual studies | 12 | Describe methods used for assessing risk of bias of individual studies (including specification of whether this was done at the study or outcome level), and how this information is to be used in any data synthesis. | 5,6, appendix J |
| Summary measures | 13 | State the principal summary measures (e.g., risk ratio, difference in means). *Also describe the use of additional summary measures assessed, such as treatment rankings and surface under the cumulative ranking curve (SUCRA) values, as well as modified approaches used to present summary findings from meta-analyses.* | 6,7 |
| Planned methods of analysis | 14 | Describe the methods of handling data and combining results of studies for each network meta-analysis. This should include, but not be limited to:   - *Handling of multi-arm trials;* - *Selection of variance structure;* - *Selection of prior distributions in Bayesian analyses; and* - *Assessment of model fit.* | 6,7 |
| **Assessment of Inconsistency** | **S2** | Describe the statistical methods used to evaluate the agreement of direct and indirect evidence in the treatment network(s) studied. Describe efforts taken to address its presence when found. | 6,7 |
| Risk of bias across studies | 15 | Specify any assessment of risk of bias that may affect the cumulative evidence (e.g., publication bias, selective reporting within studies). | 5, appendix J |
| Additional analyses | 16 | Describe methods of additional analyses if done, indicating which were pre-specified. This may include, but not be limited to, the following:   - Sensitivity or subgroup analyses; - Meta-regression analyses; - *Alternative formulations of the treatment network; and* - *Use of alternative prior distributions for Bayesian analyses (if applicable).* | 7 |
|  |  |  |  |
| **RESULTS†** |  |  |  |
| Study selection | 17 | Give numbers of studies screened, assessed for eligibility, and included in the review, with reasons for exclusions at each stage, ideally with a flow diagram. | 8 |
| **Presentation of network structure** | **S3** | Provide a network graph of the included studies to enable visualization of the geometry of the treatment network. | Figure 2 |
| **Summary of network geometry** | **S4** | Provide a brief overview of characteristics of the treatment network. This may include commentary on the abundance of trials and randomized patients for the different interventions and pairwise comparisons in the network, gaps of evidence in the treatment network, and potential biases reflected by the network structure. | 9 |
| Study characteristics | 18 | For each study, present characteristics for which data were extracted (e.g., study size, PICOS, follow-up period) and provide the citations. | 8,9, Table 1 |
| Risk of bias within studies | 19 | Present data on risk of bias of each study and, if available, any outcome level assessment. | 9, appendix J, Table 1 |
| Results of individual studies | 20 | For all outcomes considered (benefits or harms), present, for each study: 1) simple summary data for each intervention group, and 2) effect estimates and confidence intervals. *Modified approaches may be needed to deal with information from larger networks.* | 9, 10 appendix L, appendix M |
| Synthesis of results | 21 | Present results of each meta-analysis done, including confidence/credible intervals. *In larger networks, authors may focus on comparisons versus a particular comparator (e.g. placebo or standard care), with full findings presented in an appendix. League tables and forest plots may be considered to summarize pairwise comparisons.* If additional summary measures were explored (such as treatment rankings), these should also be presented. | 9, 10, 11, Figure 3, figure 4, appendix L, appendix M |
| **Exploration for inconsistency** | **S5** | Describe results from investigations of inconsistency. This may include such information as measures of model fit to compare consistency and inconsistency models, *P* values from statistical tests, or summary of inconsistency estimates from different parts of the treatment network. | 9, 10, appendix L, appendix M |
| Risk of bias across studies | 22 | Present results of any assessment of risk of bias across studies for the evidence base being studied. | 9, appendix J |
| Results of additional analyses | 23 | Give results of additional analyses, if done (e.g., sensitivity or subgroup analyses, meta-regression analyses*, alternative network geometries studied, alternative choice of prior distributions for Bayesian analyses,* and so forth). | 11, appendix K, appendix N |
|  |  |  |  |
| **DISCUSSION** |  |  |  |
| Summary of evidence | 24 | Summarize the main findings, including the strength of evidence for each main outcome; consider their relevance to key groups (e.g., healthcare providers, users, and policy-makers). | 11,12 |
| Limitations | 25 | Discuss limitations at study and outcome level (e.g., risk of bias), and at review level (e.g., incomplete retrieval of identified research, reporting bias). *Comment on the validity of the assumptions, such as transitivity and consistency. Comment on any concerns regarding network geometry (e.g., avoidance of certain comparisons).* | 13, 14 |
| Conclusions | 26 | Provide a general interpretation of the results in the context of other evidence, and implications for future research. | 15 |
| **FUNDING** |  |  | 16 |
| Funding | 27 | Describe sources of funding for the systematic review and other support (e.g., supply of data); role of funders for the systematic review. This should also include information regarding whether funding has been received from manufacturers of treatments in the network and/or whether some of the authors are content experts with professional conflicts of interest that could affect use of treatments in the network. |  |

PICOS = population, intervention, comparators, outcomes, study design.

* Text in italics indicates wording specific to reporting of network meta-analyses that has been added to guidance from the PRISMA statement.

† Authors may wish to plan for use of appendices to present all relevant information in full detail for items in this section.

# Appendix B - Search strategy

A comprehensive anxiety literature database was used as the information source of the present review and has been registered at the open science framework (https://osf.io/9xe2g/). Development of the database began with a systematic search on 25 April 2019 and it is kept update on every January by two independent researchers. A systematic search was conducted using a full range of terms related to the applicable interventions, disorders and outcomes. Similar databases exist for depression (1) treatments of suicide (2) post-traumatic stress disorder (3) and mental health problems in children and adolescents (4). Each of these includes several hundreds of randomized trials. We have recently named such living systematic reviews focusing on a specific research area as ‘Meta-Analytic Research Domains’ (MARDs) (5). From our anxiety MARD we selected trials on panic disorder and published in 2020 a protocol for two overarching network meta-analyses (6). The first has been released in 2021, the second is described in the present manuscript.

(1) Cuijpers P. Four decades of outcome research on psychotherapies for adult depression: an overview of a series of meta-analyses. Can Psychol 2017;58:7–19

(2) Hu MX, Palantza C, Setkowski K, Gilissen R, Karyotaki E, Cuijpers P, Riper H, de Beurs D, Nuij C, Christensen H, Calear A, Werner-Seidler A, Hoogendoorn A, van Balkom A, Eikelenboom M, Smit J, van Ballegooijen W. Comprehensive database and individual patient data meta-analysis of randomised controlled trials on psychotherapies reducing suicidal thoughts and behaviour: study protocol. BMJ Open. 2020 Dec 4;10(12):e037566.

(3) U.S. Department of Veterans Affairs. National center for PTSD, 2022. Available: https://www.ptsd.va.gov/ptsdrepository/index.asp.

(4) Weisz JR, Kuppens S, Ng MY, Eckshtain D, Ugueto AM, Vaughn-Coaxum R, Jensen-Doss A, Hawley KM, Krumholz Marchette LS, Chu BC, Weersing VR, Fordwood SR. What five decades of research tells us about the effects of youth psychological therapy: A multilevel meta-analysis and implications for science and practice. Am Psychol. 2017 Feb-Mar;72(2):79-117.

(5) Cuijpers P, Miguel C, Papola D, Harrer M, Karyotaki E. From living systematic reviews to meta-analytical research domains. Evid Based Ment Health. 2022 Jul 19:ebmental-2022-300509.

(6) Papola D, Ostuzzi G, Gastaldon C, Purgato M, Del Giovane C, Pompoli A, Karyotaki E, Sijbrandij M, Furukawa TA, Cuijpers P, Barbui C. Which psychotherapy is effective in panic disorder? And which delivery formats are supported by the evidence? Study protocol for two systematic reviews and network meta-analyses. BMJ Open. 2020 Dec 28;10(12):e038909.

Last update: January 1^st^, 2022

**PubMed**

**Search Strings for PubMed**

Psychotherapy[MH] OR psychotherap*[All Fields] OR cbt[All Fields] OR "behavior therapies"[All Fields] OR "behavior therapy"[All Fields] OR "behavior therapeutic"[All Fields] OR "behavior therapeutical"[All Fields] OR "behavior therapeutics"[All Fields] OR "behavior therapeutist"[all Fields] OR "behavior therapeutists"[All Fields] OR "behavior treatment"[All Fields] OR "behavior treatments"[All Fields] OR "behaviors therapies"[All Fields] OR "behaviors therapy"[All Fields] OR "behaviors therapeutics"[All Fields] OR "behaviors therapeutic"[All Fields] OR "behaviors therapeutical"[All Fields] OR "behaviors therapeutist"[All Fields] OR "behaviors therapeutists"[All Fields] OR "behaviors treatment"[All Fields] OR "behaviors treatments"[All Fields] OR "behavioral therapies"[All Fields] OR "behavioral therapy"[All Fields] OR "behavioral therapeutics"[All Fields] OR "behavioral therapeutic"[All Fields] OR "behavioral therapeutical"[All Fields] OR "behavioral therapeutist"[All Fields] OR "behavioral therapeutists"[All Fields] OR "behavioral treatment"[All Fields] OR "behavioral treatments"[All Fields] OR "behaviour therapies"[All Fields] OR "behaviour therapy"[All Fields] OR "behaviour therapeutic"[All Fields] OR "behaviour therapeutical"[All Fields] OR "behaviour therapeutics"[All Fields] OR "behaviour therapeutist"[all Fields] OR "behaviour therapeutists"[All Fields] OR "behaviour treatment"[All Fields] OR "behaviour treatments"[All Fields] OR "behaviours therapies"[All Fields] OR "behaviours therapy"[All Fields] OR "behaviours therapeutics"[All Fields] OR "behaviours therapeutic"[All Fields] OR "behaviours therapeutical"[All Fields] OR "behaviours therapeutist"[All Fields] OR "behaviours therapeutists"[All Fields] OR "behaviours treatment"[All Fields] OR "behaviours treatments"[All Fields] OR "behavioural therapies"[All Fields] OR "behavioural therapy"[All Fields] OR "behavioural therapeutics"[All Fields] OR "behavioural therapeutic"[All Fields] OR "behavioural therapeutical"[All Fields] OR "behavioural therapeutist"[All Fields] OR "behavioural therapeutists"[All Fields] OR "behavioural treatment"[All Fields] OR "behavioural treatments"[All Fields] OR "cognition therapies"[All Fields] OR "cognition therapie"[All Fields] OR "cognition therapy"[All Fields] OR "cognition therapeutical"[All Fields] OR "cognition therapeutic"[All Fields] OR "cognition therapeutics"[All Fields] OR "cognition therapeutist"[All Fields] OR "cognition therapeutists"[All Fields] OR "cognition treatment"[All Fields] OR "cognition treatments"[All Fields] OR psychodynamic[All Fields] OR Psychoanalysis[MH] OR psychoanalysis[All Fields] OR psychoanalytic*[All Fields] OR counselling[All Fields] OR counseling[All Fields] OR Counseling[MH] OR "problem-solving"[All Fields] OR mindfulness[All Fields] OR (acceptance[All Fields] AND commitment[All Fields] ) OR "assertiveness training"[All Fields] OR "behavior activation"[All Fields] OR "behaviors activation"[All Fields] OR "behavioral activation"[All Fields] OR "cognitive therapies"[All Fields] OR "cognitive therapy"[All Fields] OR "cognitive therapeutic"[All Fields] OR "cognitive therapeutics"[All Fields] OR "cognitive therapeutical"[All Fields] OR "cognitive therapeutist"[All Fields] OR "cognitive therapeutists"[All Fields] OR "cognitive treatment"[All Fields] OR "cognitive treatments"[All Fields] OR "cognitive restructuring"[All Fields] OR (("compassion-focused"[All Fields] OR "compassion-focussed"[All Fields]) AND (therapy[SH] OR therapies[All Fields] OR therapy[All Fields] OR therape*[All Fields] OR therapis*[All Fields]OR Therapeutics [OR treatment*[All Fields])) OR ((therapy[SH] OR therapies[All Fields] OR therapy [All Fields] OR therape*[All Fields] OR therapis*[All Fields] OR Therapeutics[MH] OR treatment*[All Fields]) AND constructivist*[All Fields]) OR "metacognitive therapies"[All Fields] OR "metacognitive therapy"[All Fields] OR "metacognitive therapeutic"[All Fields] OR "metacognitive therapeutics"[All Fields] OR "metacognitive therapeutical"[All Fields] OR "metacognitive therapeutist"[All Fields] OR "metacognitive therapeutists"[All Fields] OR "metacognitive treatment"[All Fields] OR "metacognitive treatments"[All Fields] OR "meta-cognitive therapies"[All Fields] OR "meta-cognitive therapy"[All Fields] OR "meta-cognitive therapeutic"[All Fields] OR "meta-cognitive therapeutics"[All Fields] OR "meta-cognitive therapeutical"[All Fields] OR "meta-cognitive therapeutist"[All Fields] OR "meta-cognitive therapeutists"[All Fields] OR "meta-cognitive treatment"[All Fields] OR "meta-cognitive treatments"[All Fields] OR "solution-focused therapies"[All Fields] OR "solution-focused therapy"[All Fields] OR "solution-focused therapeutic"[All Fields] OR "solution-focused therapeutics"[All Fields] OR "solution-focused therapeutical"[All Fields] OR "solution focused therapies"[All Fields] OR "solution focused therapy"[All Fields] OR "solution focused therapeutic"[All Fields] OR "solution focused therapeutics"[All Fields] OR "solution focused therapeutical"[All Fields]OR "solution-focussed therapies"[All Fields] OR "solution-focussed therapy"[All Fields] OR "solution-focussed therapeutic"[All Fields] OR "solution-focussed therapeutics"[All Fields] OR "solution-focussed therapeutical"[All Fields]OR "solution focussed therapies"[All Fields] OR "solution focussed therapy"[All Fields] OR "solution focussed therapeutic"[All Fields] OR "solution focussed therapeutics"[All Fields] OR "solution focussed therapeutical"[All Fields] OR "self-control therapies"[All Fields] OR "self-control therapy"[All Fields] OR "self-control therapeutics"[All Fields] OR "self-control therapeutical"[All Fields] OR "self-control therapeutic"[All Fields] OR "self-control training"[All Fields] OR "self-control trainings"[All Fields] OR "self control therapies"[All Fields] OR "self control therapy"[All Fields] OR "self control therapeutics"[All Fields] OR "self control therapeutical"[All Fields] OR "self control therapeutic"[All Fields] OR "self control training"[All Fields] OR "self control trainings"[All Fields] OR exposure[All Fields] OR relaxation[All Fields] OR EMDR[All Fields] OR ("eye movement" and desensiti*[All Fields]) OR "panic management"[All Fields] OR "response prevention"[All Fields] OR ERP[All Fields]

AND

"social anxiety"[All Fields] OR shy[All Fields] OR ("shyness"[MeSH Terms] OR "shyness"[All Fields]) OR "test anxiety"[All Fields] OR gad[All Fields] OR "generalized anxiety"[All Fields] OR "generalised anxiety"[All Fields] OR worry[All Fields] OR ("panic"[MeSH Terms] OR "panic"[All Fields]) OR (agoraphobi[All Fields] OR agoraphobia[All Fields] OR agoraphobia'[All Fields] OR agoraphobia's[All Fields] OR agoraphobias[All Fields] OR agoraphobic[All Fields] OR agoraphobic's[All Fields] OR agoraphobics[All Fields] OR agoraphobics'[All Fields] OR agoraphobie[All Fields] OR agoraphobien[All Fields]) OR "anxiety disorder"[All Fields] OR "social phobia"[All Fields] OR "social anxiety disorder"[All Fields] OR Arachnophobia[All Fields] OR Ophidiophobia[All Fields] OR Acrophobia[All Fields] OR Agoraphobia[All Fields] OR Cynophobia[All Fields] OR Claustrophobia[All Fields] OR Mysophobia[All Fields] OR Aerophobia[All Fields] OR Trypophobia[All Fields] OR Carcinophobia[All Fields] OR Thanatophobia[All Fields] OR Glossophobia[All Fields] OR Monophobia[All Fields] OR Ornithophobia[All Fields] OR Alektorophobia[All Fields] OR Trypanophobia[All Fields] OR Anthropophobia[All Fields] OR Aquaphobia[All Fields] OR Autophobia[All Fields] OR Hemophobia[All Fields] OR Xenophobia[All Fields] OR Ailurophobia[All Fields] OR Nyctophobia[All Fields] OR Phobophobia[All Fields] OR Philophobia[All Fields] OR Triskaidekaphobia[All Fields] OR Emetophobia[All Fields] OR Entomophobia[All Fields] OR Zoophobia[All Fields] OR Scelerophobia[All Fields] OR Cibophobia[All Fields] OR Tokophobia[All Fields] OR Pseudodysphagia[All Fields] OR Gerascophobia[All Fields] OR Technophobia[All Fields] OR Ergophobia[All Fields] OR Coulrophobia [All Fields] OR Photophobia[All Fields] OR Numerophobia[All Fields] OR Taphophobia

**PsycINFO**

DE "Psychotherapy" OR "Psychotherapy" OR "psychotherapies" OR "psychotherapeutic" OR "psychotherapeutical" OR "psychotherapeutics" OR DE "Behavior Therapy" OR DE "Cognitive Behavior Therapy" OR "CBT" OR "behavior therapies" OR "behavior therapy" OR "behavior therapeutic" OR "behavior therapeutical" OR "behavior therapeutics" OR "behavior therapeutist" OR "behavior therapeutists" OR "behavior treatment" OR "behavior treatments" OR "behaviors therapies" OR "behaviors therapy" OR "behaviors therapeutics" OR "behaviors therapeutic" OR "behaviors therapeutical" OR "behaviors therapeutist" OR "behaviors therapeutists" OR "behaviors treatment" OR "behaviors treatments" OR "behavioral therapies" OR "behavioral therapy" OR "behavioral therapeutics" OR "behavioral therapeutic" OR "behavioral therapeutical" OR "behavioral therapeutist" OR "behavioral therapeutists" OR "behavioral treatment" OR "behavioral treatments" OR "behaviour therapies" OR "behaviour therapy" OR "behaviour therapeutic" OR "behaviour therapeutical" OR "behaviour therapeutics" OR "behaviour therapeutist" OR "behaviour therapeutists" OR "behaviour treatment" OR "behaviour treatments" OR "behaviours therapies" OR "behaviours therapy" OR "behaviours therapeutics" OR "behaviours therapeutic" OR "behaviours therapeutical" OR "behaviours therapeutist" OR "behaviours therapeutists" OR "behaviours treatment" OR "behaviours treatments" OR "behavioural therapies" OR "behavioural therapy" OR "behavioural therapeutics" OR "behavioural therapeutic" OR "behavioural therapeutical" OR "behavioural therapeutist" OR "behavioural therapeutists" OR "behavioural treatment" OR "behavioural treatments" OR "cognition therapies" OR "cognition therapie" OR "cognition therapy" OR "cognition therapeutical" OR "cognition therapeutic" OR "cognition therapeutics" OR "cognition therapeutist" OR "cognition therapeutists" OR "cognition treatment" OR "cognition treatments" OR "cognitive therapies" OR "cognitive therapy" OR "cognitive therapeutic" OR "cognitive therapeutics" OR "cognitive therapeutical" OR "cognitive therapeutist" OR "cognitive therapeutists" OR "cognitive treatment" OR "cognitive treatments" OR "cognitive restructuring" OR DE "Emotion Focused Therapy" OR DE "Psychoanalysis" OR "psychoanalysis" OR "psychoanalytic" OR "psychoanalytical "OR DE "Psychodynamic Psychotherapy" OR "psychodynamic" OR DE "Psychotherapeutic Counseling" OR "counselling" OR "counseling" OR "problem-solving" OR "problem solving" OR "mindfulness" OR "acceptance and commitment" OR "assertiveness training" OR "behavior activation" OR "behaviors activation" OR "behavioral activation" OR "behaviour activation" OR "behaviours activation" OR "behavioural activation" OR "metacognitive therapies" OR "metacognitive therapy" OR "metacognitive therapeutic" OR "metacognitive therapeutics" OR "metacognitive therapeutical" OR "metacognitive therapeutist" OR "metacognitive therapeutists" OR "metacognitive treatment" OR "metacognitive treatments" OR "meta-cognitive therapies" OR "meta-cognitive therapy" OR "meta-cognitive therapeutic" OR "meta-cognitive therapeutics" OR "meta-cognitive therapeutical" OR "meta-cognitive therapeutist" OR "meta-cognitive therapeutists" OR "meta-cognitive treatment" OR "meta-cognitive treatments" OR DE "Solution Focused Therapy" OR "solution-focused therapies" OR "solution-focused therapy" OR "solution-focused therapeutic" OR "solution-focused therapeutics" OR "solution-focused therapeutical" OR "solution-focussed therapies" OR "solution-focussed therapy" OR "solution-focussed therapeutic" OR "solution-focussed therapeutics" OR "solution-focussed therapeutical" OR "solution focused therapies" OR "solution focused therapy" OR "solution focused therapeutic" OR "solution focused therapeutics" OR "solution focused therapeutical" OR "solution focussed therapies" OR "solution focussed therapy" OR "solution focussed therapeutic" OR "solution focussed therapeutics" OR "solution focussed therapeutical" OR "self-control therapies" OR "self-control therapy" OR "self-control therapeutics" OR "self-control therapeutical" OR "self-control therapeutic" OR "self-control training" OR "self-control trainings" OR "self control therapies" OR "self control therapy" OR "self control therapeutics" OR "self control therapeutical" OR "self control therapeutic" OR "self control training" OR "self control trainings" OR "compassion-focused" OR "compassion-focussed" OR "compassion focused" OR "compassion focussed" OR "therapies" OR "therapy" OR "therapie" OR "therapist" OR "therapists" OR "therapeut" OR "treatment" OR "treatments" OR "constructivist" OR "therapies" OR "therapy" OR "therapie" OR "therapist" OR "therapists" OR "therapeut" OR "treatment" OR "treatments" OR "exposure" OR "relaxation" OR "EMDR" OR "eye movement and reprocessing" OR "panic management" OR "response prevention" OR "ERP"

AND

"social anxiety" or "shy" or "shyness" or "test anxiety" or "gad" or "generalized anxiety" or "generalised anxiety" or "worry" or "panic" or "agoraphobi*" OR "anxiety disorder" or "anxiety disorders" or "SAD" or "social phobia" or "social anxiety disorder" or "arachnophobia" or "ophidiophobia" or "acrophobia" or "agoraphobia" or "cynophobia or "claustrophobia" or "mysophobia or "aerophobia" or "trypophobia or "carcinophobia" or "thanatophobia" or "glossophobia" or "monophobia" or "ornithophobia" or "alektorophobia" or "trypanophobia" or "anthropophobia" or "aquaphobia" or "autophobia" or "hemophobia" or "xenophobia" or "ailurophobia" or "nyctophobia" or "phobophobia" or "philophobia" or "triskaidekaphobia" or "emetophobia" or "entomophobia" or "zoophobia" or "scelerophobia" or "cibophobia" or "tokophobia" or "pseudodysphagia" or "gerascophobia" or "technophobia" or "ergophobia" or "coulrophobia" or "hotophobia" or "numerophobia" or "taphophobia"

**Embase**

'psychotherapy'/exp OR psychotherap* OR 'psychotherapy' OR 'psychotherapy' OR ‘psychotherapies’ OR ‘psychotherapeutic’ OR ‘psychotherapeutical’ OR ‘psychotherapeutics’ OR ‘Behavior Therapy’/exp OR ‘Cognitive Behavioral Therapy’/exp OR ‘CBT’ OR ‘behavior therapies’ OR ‘behavior therapy’ OR ‘behavior therapeutic’ OR ‘behavior therapeutical’ OR ‘behavior therapeutics’ OR ‘behavior therapeutist’ OR ‘behavior therapeutists’ OR ‘behavior treatment’ OR ‘behavior treatments’ OR ‘behaviors therapies’ OR ‘behaviors therapy’ OR ‘behaviors therapeutics’ OR ‘behaviors therapeutic’ OR ‘behaviors therapeutical’ OR ‘behaviors therapeutist’ OR ‘behaviors therapeutists’ OR ‘behaviors treatment’ OR ‘behaviors treatments’ OR ‘behavioral therapies’ OR ‘behavioral therapy’ OR ‘behavioral therapeutics’ OR ‘behavioral therapeutic’ OR ‘behavioral therapeutical’ OR ‘behavioral therapeutist’ OR ‘behavioral therapeutists’ OR ‘behavioral treatment’ OR ‘behavioral treatments’ OR ‘behaviour therapies’ OR ‘behaviour therapy’ OR ‘behaviour therapeutic’ OR ‘behaviour therapeutical’ OR ‘behaviour therapeutics’ OR ‘behaviour therapeutist’ OR ‘behaviour therapeutists’ OR ‘behaviour treatment’ OR ‘behaviour treatments’ OR ‘behaviours therapies’ OR ‘behaviours therapy’ OR ‘behaviours therapeutics’ OR ‘behaviours therapeutic’ OR ‘behaviours therapeutical’ OR ‘behaviours therapeutist’ OR ‘behaviours therapeutists’ OR ‘behaviours treatment’ OR ‘behaviours treatments’ OR ‘behavioural therapies’ OR ‘behavioural therapy’ OR ‘behavioural therapeutics’ OR ‘behavioural therapeutic’ OR ‘behavioural therapeutical’ OR ‘behavioural therapeutist’ OR ‘behavioural therapeutists’ OR ‘behavioural treatment’ OR ‘behavioural treatments’ OR ‘cognition therapies’ OR ‘cognition therapie’ OR ‘cognition therapy’ OR ‘cognition therapeutical’ OR ‘cognition therapeutic’ OR ‘cognition therapeutics’ OR ‘cognition therapeutist’ OR ‘cognition therapeutists’ OR ‘cognition treatment’ OR ‘cognition treatments’ OR ‘cognitive therapies’ OR ‘cognitive therapy’ OR ‘cognitive therapeutic’ OR ‘cognitive therapeutics’ OR ‘cognitive therapeutical’ OR ‘cognitive therapeutist’ OR ‘cognitive therapeutists’ OR ‘cognitive treatment’ OR ‘cognitive treatments’ OR ‘cognitive restructuring’ OR ‘Emotion Focused Therapy’/exp OR ‘Psychoanalysis’/exp OR ‘psychoanalysis’ OR ‘psychoanalytic’ OR ‘psychoanalytical‘ OR ‘Psychodynamic Psychotherapy’/exp OR ‘psychodynamic’ OR ‘Psychotherapeutic Counseling’/exp OR ‘counselling’ OR ‘counseling’ OR ‘problem-solving’ OR ‘problem solving’ OR ‘mindfulness’ OR ‘acceptance and commitment’ OR ‘assertiveness training’ OR ‘behavior activation’ OR ‘behaviors activation’ OR ‘behavioral activation’ OR ‘behaviour activation’ OR ‘behaviours activation’ OR ‘behavioural activation’ OR ‘metacognitive therapies’ OR ‘metacognitive therapy’ OR ‘metacognitive therapeutic’ OR ‘metacognitive therapeutics’ OR ‘metacognitive therapeutical’ OR ‘metacognitive therapeutist’ OR ‘metacognitive therapeutists’ OR ‘metacognitive treatment’ OR ‘metacognitive treatments’ OR ‘meta-cognitive therapies’ OR ‘meta-cognitive therapy’ OR ‘meta-cognitive therapeutic’ OR ‘meta-cognitive therapeutics’ OR ‘meta-cognitive therapeutical’ OR ‘meta-cognitive therapeutist’ OR ‘meta-cognitive therapeutists’ OR ‘meta-cognitive treatment’ OR ‘meta-cognitive treatments’ OR ‘Solution Focused Therapy’/exp OR ‘solution-focused therapies’ OR ‘solution-focused therapy’ OR ‘solution-focused therapeutic’ OR ‘solution-focused therapeutics’ OR ‘solution-focused therapeutical’ OR ‘solution-focussed therapies’ OR ‘solution-focussed therapy’ OR ‘solution-focussed therapeutic’ OR ‘solution-focussed therapeutics’ OR ‘solution-focussed therapeutical’ OR ‘solution focused therapies’ OR ‘solution focused therapy’ OR ‘solution focused therapeutic’ OR ‘solution focused therapeutics’ OR ‘solution focused therapeutical’ OR ‘solution focussed therapies’ OR ‘solution focussed therapy’ OR ‘solution focussed therapeutic’ OR ‘solution focussed therapeutics’ OR ‘solution focussed therapeutical’ OR ‘self-control therapies’ OR ‘self-control therapy’ OR ‘self-control therapeutics’ OR ‘self-control therapeutical’ OR ‘self-control therapeutic’ OR ‘self-control training’ OR ‘self-control trainings’ OR ‘self control therapies’ OR ‘self control therapy’ OR ‘self control therapeutics’ OR ‘self control therapeutical’ OR ‘self control therapeutic’ OR ‘self control training’ OR ‘self control trainings’ OR ‘compassion-focused’ OR ‘compassion-focussed’ OR ‘compassion focused’ OR ‘compassion focussed’ OR ‘exposure’ OR ‘relaxation’ OR ‘EMDR’ OR ‘eye movement and reprocessing’ OR ‘panic management’ OR ‘response prevention’ OR ‘ERP’

AND

'anxiety disorder'/exp OR 'anxiety disorder' OR 'gad'/exp OR 'gad' OR 'generalized anxiety disorder'/exp OR 'generalized anxiety disorder' OR 'generalised anxiety disorder'/exp OR 'generalised anxiety disorder' OR 'generalized anxiety' OR 'generalised anxiety' OR 'worry' OR 'social phobia'/exp OR 'social phobia' OR 'social anxiety disorder'/exp OR 'social anxiety disorder' OR 'social anxiety' OR 'acute stress disorder'/exp OR 'acute stress disorder' OR 'acute stress' OR 'panic'/exp OR 'panic' OR ‘agorophobia' OR ’Arachnophobia’ OR ’Ophidiophobia’ OR ’Acrophobia’ OR ’Agoraphobia’ OR ’Cynophobia’ OR ’Claustrophobia’ OR ’Mysophobia’ OR ’Aerophobia’ OR ’Trypophobia’ OR ’Carcinophobia’ OR ’Thanatophobia’ OR ’Glossophobia’ OR ’Monophobia’ OR ’Ornithophobia’ OR ’Alektorophobia’ OR ’Trypanophobia’ OR ’Anthropophobia’ OR ’Aquaphobia’ OR ’Autophobia’ OR ’Hemophobia’ OR ’Xenophobia’ OR ’Ailurophobia’ OR ’Nyctophobia’ OR ’Phobophobia’ OR ’Philophobia’ OR ’Triskaidekaphobia’ OR ’Emetophobia’ OR ’Entomophobia’ OR ’Zoophobia’ OR ’Scelerophobia’ OR ’Cibophobia’ OR ’Tokophobia’ OR ’Pseudodysphagia’ OR ’Gerascophobia’ OR ’Technophobia’ OR ’Ergophobia’ OR ’Coulrophobia‘ OR ’Photophobia’ OR ’Numerophobia’ OR ’Taphophobia’

**Cochrane Library**

ID Search

#1 "social anxiety disorder":ti,ab,kw (Word variations have been searched)

#2 "GAD":ti,ab,kw (Word variations have been searched)

#3 "social anxiety":ti,ab,kw (Word variations have been searched)

#4 "test anxiety":ti,ab,kw (Word variations have been searched)

#5 "generalised anxiety disorder":ti,ab,kw (Word variations have been searched)

#6 "generalised anxiety":ti,ab,kw (Word variations have been searched)

#7 "worry":ti,ab,kw (Word variations have been searched)

#8 "panic":ti,ab,kw (Word variations have been searched)

#9 "panic disorder":ti,ab,kw (Word variations have been searched)

#10 "agoraphobia":ti,ab,kw (Word variations have been searched)

#11 "phobia":ti,ab,kw (Word variations have been searched)

#12 #1 OR #2 OR  #3 OR  #4 OR  #5 OR  #6 OR  #7 OR  #8 OR  #9 OR #10 OR #11

#13 "psychotherapy":kw (Word variations have been searched)

#14 "CBT":ti,ab,kw (Word variations have been searched)

#15 cognitive behavi* therap*:ti,ab,kw (Word variations have been searched)

#16 psychodynamic:ti,ab,kw (Word variations have been searched)

#17 "psychoanalysis":ti,ab,kw (Word variations have been searched)

#18 psychoanalys*:ti,ab,kw (Word variations have been searched)

#19 "counseling":ti,ab,kw (Word variations have been searched)

#20 "problem solving":ti,ab,kw (Word variations have been searched)

#21 "acceptance and commitment":ti,ab,kw (Word variations have been searched)

#22 "assertiveness training":ti,ab,kw (Word variations have been searched)

#23 "behavioral activation":ti,ab,kw (Word variations have been searched)

#24 "behavioural activation":ti,ab,kw (Word variations have been searched)

#25 "mindfulness":ti,ab,kw (Word variations have been searched)

#26 "metacognitive therap*":ti,ab,kw (Word variations have been searched)

#27 "solution focused therap*":ti,ab,kw (Word variations have been searched)

#28 "self-control therap*":ti,ab,kw (Word variations have been searched)

#29 "self-control training":ti,ab,kw (Word variations have been searched)

#30 "exposure":ti,ab,kw (Word variations have been searched)

#31 "relaxation":ti,ab,kw (Word variations have been searched)

#32 "EMDR":ti,ab,kw (Word variations have been searched)

#33 "eye movement desensitization":ti,ab,kw (Word variations have been searched)

#34 "panic management":ti,ab,kw (Word variations have been searched)

#35 "response prevention":ti,ab,kw (Word variations have been searched)

#36 #13 OR #14 OR  #15 OR  #16 OR  #17 OR  #18 OR  #19 OR  #20 OR  #21 OR #22 OR #23 #24 OR #25 OR  #26 OR  #27 OR  #28 OR  #29 OR  #30 OR  #31 OR  #32 OR #33 OR #34 OR #35

#37 #12 AND #36 in Trials

**Appendix C - Definitions of interventions, controls and type of delivery formats**

Table C1. CBT treatment delivery formats and their definitions.

| Treatment delivery formats | Definition |
| --- | --- |
| In-person face-to-face individual | The psychotherapy is delivered by the therapist in a face-to-face individual setting. |
| Remote synchronous face-to-face individual | The psychotherapy is delivered individually by the therapist via videoconference. |
| In-person face-to-face group | The psychotherapy is delivered by the therapist in a face-to-face group setting. |
| Remote synchronous face-to-face group | The psychotherapy is delivered by the therapist via videoconference to a group of patients. |
| Remote guided self-help | A psychotherapy in which a professional therapist is involved in the treatment process, offering guidance to the patient using the self-help materials (administered through the internet, telephone or other media, such as a book). |
| Remote unguided self-help | A psychotherapy in which no professional guidance is provided to the patient using the self-help materials (internet-based or not). Technical assistance with the software provided to the patent or general encouragement to pursue the program did not count as guidance |

Table C2. Control conditions and their definitions

| Control group | Definition |
| --- | --- |
| Treatment as usual (TAU) | Participants receive assessment only, with or without simple provision of informational material or minimal therapist contact or routine pharmacotherapy or all, and they know that they will not receive the active treatment in question after the trial. The participants in this condition are usually allowed to seek treatment as available in the community; when such additive treatments are substantive, we will include such trials only if it is balanced between the two arms to be compared. |
| Waiting list (WL) | Participants receive assessment, with or without simple provision of informational material or minimal therapist contact, or both, and they know that they will receive the active treatment in question after the waiting phase. |
| Psychological placebo (PP) | Participants receive a face-to-face inactive intervention that can be perceived both as ineffective or effective. |
| Placebo pill | Placebo pill. |

**Appendix D - Hierarchy of symptom severity measurement scales**

| Hierarchy | Symptom severity rating scales | Abbreviation |
| --- | --- | --- |
| 1 | Panic Disorder Severity Scale | PDSS |
| 2 | Panic and Agoraphobia Scale | PAS |
| 3 | Anxiety Sensitivity Index - Revised | ASI-R |
| 4 | Anxiety Sensitivity Index | ASI |
| 5 | Agoraphobic Cognitions Questionnaire | ACQ |
| 6 | Body Sensations Questionnaire | BSQ |
| 7 | Other scales specifically focused on panic disorder | |
| 8 | Hamilton anxiety scale | HAMA |
| 9 | Beck depression inventory | BAI |
| 9 | Clinical Global Impression Severity Scale | CGI-S |
| 10 | Clinical Global  Impression – Improvement scale | CGI-I |
| 11 | Global Assessment Scale | GAS |
| 12 | Global Assessment of Functioning | GAF |
| 13 | Other global anxiety and psychological symptoms scales | |
| 14 | Fear Questionnaire-agoraphobia subscale | FQ-agoraphobia |
| 15 | Fear Questionnaire- global | FQ global |
| 16 | Mobile Inventory for Agoraphobia-  Avoidance-Alone | MI-AAL |
| 17 | Mobility Inventory -Avoidance-Accompanied | MIAAC |
| 18 | Other scales specifically focused on agoraphobia | |
| 19 | Panic frequency | |
| 20 | Panic severity | |
| 21 | Other scales specific for panic attacks only | |

# Appendix E - Characteristics of the included studies

|  | | | **P** | | | | | **I** | | | | **C** | | **O** | | | | pharmacotherapy | Analysis ITT? | ROB 2 | Contributes to the network? |
| --- | --- | --- | --- | --- | --- | --- | --- | --- | --- | --- | --- | --- | --- | --- | --- | --- | --- | --- | --- | --- | --- |
| Unique ID | First author, year | country | Participant diagnosis | Type of recruitment | Mean age (years) | Prop% women | Prop% agoraphobics | name of the CBT intervention | Description of the CBT intervention | format | N sessions /modules | Type of comparison | Assigned subgroup (network node) | Rating scale | Self reported scale? | study end-point (weeks) | other measurements (weeks) |  |  |  |  |
| 1 | Addis, 2004 | USA | DSM-IV criteria for a panic disorder with or without agoraphobia | community | 39,9 | 70 | 73 | Panic control therapy (PCT) | PCT is a manual-based 12–15-session cognitive– behavioral treatment protocol. The treatment includes education about the causes and maintenance of panic disorder, breathing retraining, cognitive restructuring, and interoceptive and agoraphobic exposure components. | individual | 12-15 | TAU | TAU | PDSS | No | 22 | 22,34 | Yes | Yes | Low risk | Yes |
| 2 | Allen 2016 | Australia | DSM-IV criteria for a panic disorder with or without agoraphobia | Clinical | 38 | 84 | not specified for PDA sub-sample | Panic course | A unguided five-lesson online CBT programme. Automatic emails are sent congratulating the patient when they complete a lesson. Patients have 8 weeks to complete the entire programme. | Unguided self-help | 5 | WL | WL | PDSS-SR | Yes | 8 | 12 | Yes | Yes | Low risk | yes |
| 3 | Bakker, 1999 | Europe | DSM III-R primary diagnosis of panic disorder | Clinical | 34 | 64 | 54 (had moderate or severe agoraphobia) | Cognitive therapy | Cognitive therapy was based on the cognitive theory of Clark. By means of Socratic dialogue with the therapist, patients were challenged to replace their socalled causal catastrophic misinterpretations of benign bodily sensations by alternative, rational, and nondistressing thoughts. During the treatment, behavioral experiments were introduced to test the empirical basis for the causal catastrophic misinterpretations. | individual | 12 | placebo | placebo | HAMA | no | 12 | / | No BZP allowed | No | some concerns | Yes |
|  |  |  |  |  |  |  |  | Paroxetine | *Not includable in the network* | - | - |  |  |  |  |  |  |  |  |  |  |
|  |  |  |  |  |  |  |  | clomipramine | *Not includable in the network* | - | - |  |  |  |  |  |  |  |  |  |  |
| 4 | Barlow, 1989 | USA | DSMIII-R diagnosis of panic disorder with mild or no agoraphobic avoidance | Mixed clinical and community | 31,7 | 77 | 0 | Applied progressive muscle relaxation | *Not includable in the network* | - | - | WL | WL | HAMA | yes | post-treatment | 12,24,48,96 after treatment completion | Yes | no | High risk | Yes |
|  |  |  |  |  |  |  |  | Exposure + cognitive restructuring | A skills training approach was implemented in which cognitive skills were acquired for coping with anxiety and for re-evaluating beliefs and appraisals about environmental and internal physiological cues. | individual | 15 |  |  |  |  |  |  |  |  |  |  |
|  |  |  |  |  |  |  |  | Exposure + cognitive restructuring + relaxation |  |  | 15 |  |  |  |  |  |  |  |  |  |  |
| 5 | Barlow, 2000 | USA | principal diagnosis of PD with or without mild agoraphobia | community | 36 | 62,5 | 0 | CBT | Cognitive-behavioral therapy for PD, developed at the Boston site, combines interoceptive exposure, cognitive restructuring, and breathing re- training. | individual | 11 | placebo | placebo | PDSS | no | 12 | 36,60 | yes | yes | some concerns | yes |
|  |  |  |  |  |  |  |  | Imipramine | *Not includable in the network* | - | - |  |  |  |  |  |  |  |  |  |  |
|  |  |  |  |  |  |  |  | CBT + imipramine | *Not includable in the network* | - | - |  |  |  |  |  |  |  |  |  |  |
|  |  |  |  |  |  |  |  | CBT + placebo | *Not includable in the network* | - | - |  |  |  |  |  |  |  |  |  |  |
| 6 | Beck, 1992^12^ | USA | DSM-III criteria for panic disorder or agoraphobia with panic attacks | Clinical | Not reported | Not reported | 18 | focused cognitive therapy | A brief, structured psychotherapy based on the hypothesis that panic attacks are the result of a vicious cycle involving fear or imminent physical or psychological disaster arising from the misinterpretation of certain bodily sensations or psychological experiences | individual | 12 | 8 weeks of brief supportive psychotherapy | WL* | BAI | yes | 4 | 8 | Yes | no | High risk | yes |
| 7 | Berger, 2017 | Europe | primary diagnosis of SAD, PDA or GDA as indicated by the MD and confirmed by the SCID | Mixed clinical and community | Not specified for the PDA sub-sample | | | unguided ICBT | Internet-based self-help programme ‘Velibra’. Treatment is cognitive–behavioural in orientation and emphasize transdiagnostic principles, such as anxiety as an evolutionary adaptive emotion, the ‘false alarm’ model of anxiety, experiential avoidance, and the role of approach v. avoidance motivation. Dialogue’ emerges between programme and user. Tailoring and personalization are achieved by adjusting content to expressed preferences and endorsed characteristics (e.g. current symptoms, desire for background detail or wish to skip optional sections). | Unguided self-help | 6 | WL | WL | ACQ | yes | 9 | 24 | yes | yes | Low risk | yes |
| 8 | Bergstrom, 2010 | Europe | DSM-IV criteria for panic disorder with or without agoraphobia | Clinical | 34 | 61,5 | 84,5 | CBT | A standard CBT approach. | Group | 10 | / | / | PDSS | no | post-treatment (10?) | 24 | yes | yes | some concerns | yes |
|  |  |  |  |  |  |  |  | internet CBT | self-help modules which were based on established CBT principles: psychoeducation (module 1), cognitive restructuring (modules 2 and 3), interoceptive exposure (modules 4 and 5), exposure in-vivo (for agoraphobic situations; modules 6 to 9), and relapse prevention (module 10). Each module ended with a number of questions to be answered by the patient through interactive forms (e.g. homework assignments). After reviewing these answers, the psychologist gave access to the next module and provided feedback. At any moment the patient could post a message if he or she needed further help. Messages were answered within 24 hours on regular weekdays. | Guided self-help | 10 |  |  |  |  |  |  |  |  |  |  |
| 9 | Bohni, 2009 | Europe | primary DSM-IV diagnosis of panic disorder with agoraphobia or without agoraphobia | Clinical | 35 | 72 | 90 | Massed CBT (M-CBT) | M-CBT consisted of daily 4-h sessions for 5 days in week 1, two 2-h sessions in week 2 and one 2-h session in week 3, | Group | 8 | / | / | - | - | post-treatment | 12,24,72 after treatment completion | yes | yes | some concerns | No. study groups are not suitable for the network |
|  |  |  |  |  |  |  |  | Standard CBT (S-CBT) | Standard CBT (S-CBT) consisted of 13 weekly sessions each lasting 2 h. | Group | 13 |  |  |  |  |  |  |  |  |  |  |
| 10 | Botella, 1999 | Europe | All patients received DSM-III-R (APA, 1987) diagnosis of panic disorder, 17 with agoraphobia (74%) and 6 without agoraphobia (26%) | Clinical | 29 | 78 | 74 | BRTC supported by self-help materials. | This treatment was adapted from the Standard CB. The total contact time with the therapist was 4.16 hours. | Individual | 5 | / | / | - | - | post-treatment | 48 after treatment completion | yes | no | High risk | No. study groups are not suitable for the network |
|  |  |  |  |  |  |  |  | Standard CBT | The treatment components were: (a) Educational (b) Cognitive therapy, which involved identifying and modifying catastrophic interpretations of bodily sensations in panic attacks. (c) Breathing retraining (d) Relapse prevention. Total time of contact with the therapist was about 8.33 hours. | individual | 10 |  |  |  |  |  |  |  |  |  |  |
| 11 | Botella, 2007^20^ | Europe | DSM-IV criteria for the diagnosis of PDA | Clinical | 34,7 | 72,5 | 82,9 | In vivo exposure (IVE) | The treatment was composed of (1) education about anxiety and PDA, cognitive restructuring and breathing training; exposure to internal and external stimuli (IVE or VRE) and relapse prevention. The difference between the two treatment conditions was the exposure component, which was delivered in vivo in the IVE group and in a computer-generated environment in the VRE group. | individual | 9 | WL | WL | PDSS | no | post-treatment | 48 | yes | unclear | some concerns | yes |
|  |  |  |  |  |  |  |  | Virtual reality exposure (VRE) |  |  |  |  |  |  |  |  |  |  |  |  |  |
| 12 | Brown, 1997^22^ | USA | DSM-III-R criteria for panic disorder with or without Agoraphobia | community | 33 | 67,5 | 75 | Focused cognitive therapy | According to the cognitive model, panic attacks are often triggered and perpetuated by misinterpreting benign physical or psychological sensations as indications of an immediately impending catastrophe. This cognitive abnormality then feeds into a vicious feedback loop in which the catastrophic misinterpretations produce everincreasing anxiety which, in turn, strengthens the feared sensations culminating in panic attacks. Several cognitive and behavioral techniques are used in FCT in order to assist patients in reattributing their feared sensations to more benign etiologies. | individual | 12-18 | / | / | - | - | post-treatment | 24,48 after treatment completion | yes | yes | some concerns | No. study groups are not suitable for the network |
|  |  |  |  |  |  |  |  | Standard cognitive therapy | This treatment is based on the assumption that panic attacks represent an exacerbation, or a “spiking” of the general symptomatology of the anxious patient. Hence, SCT helps patients manage and cope with anxiety-provoking situations. In accordance with this theoretical approach, lowering the general level of anxiety should reduce the frequency, intensity, and duration of panic attacks. | individual | 12-18 |  |  |  |  |  |  |  |  |  |  |
| 13 | Carlbring, 2006 | Europe | DSM-IV criteria for panic disorder | community | 36,7 | 60 | Not reported | Internet-based bibliotherapy self- help program | The treatment includable modules on psychoeducation and socialization; breathing retraining and hyperventilation test; cognitive restructuring; interoceptive exposure; exposure in vivo; relapse prevention and assertiveness training. The total number of reciprocal contacts between therapist and client ranged from seven to 29 (mean=13.5, SD=4.4). Because the e-mail responses to the participants often were very similar, much text could be recycled while care was being taken not to miss the specifics of each individual client. The mean time spent on each participant per week was approximately 12 minutes, including administration and responding to e-mail. Weekly telephone calls were made by the therapists to each participant. The purpose was to provide positive feedback and answer questions about the modules. Telephone conversations were timed and lasted an average of 11.8 minutes per week (range=9.6–15.6). | Guided self-help | 10 | WL | WL | BSQ | yes | post-treatment | 36 after treatment completion | yes | yes | Some concerns | yes |
| 14 | Carlbring, 2005 | Europe | DSM-IV criteria for PD | community | 35 | 71 | 51 | CBT | The treatment was manualized and divided into 10 modules: (1–2) psychoeducation and socialization, (3) breathing retraining and hyperventilation test, (4–5) cognitive restructuring, (6–7) interoceptive exposure, (8–9) exposure in vivo, and finally (10) relapse prevention and assertiveness training. Each module consisted of approximately 25 pages. | individual | 10 | / | / | BSQ | yes | post-treatment | 48 after treatment completion | yes | yes | Low risk | yes |
|  |  |  |  |  |  |  |  | internet-based CBT | Each module was converted into web pages and was accessible via the internet. , a subjective assessment was made by the therapist of whether the participant was ready to continue; if so, the password to the next module was sent. If not, the participant received instructions on what needed to be completed before proceeding to the next module. All contact was exclusively via e-mail. The participants were encouraged to come up with questions or reflections during treatment, and they were free to send an unlimited number of emails. The total number of reciprocal contacts (receive and send) ranged from 4 to 31. As the e-mail response to the participants often were very similar much text could be recycled. The mean total time spent on each participant was approximately 150 min, including administration, and responding to the e-mails. | Guided self-help | 10 |  |  |  |  |  |  |  |  |  |  |
| 15 | Carlbring, 2001 | Europe | DSM-IV criteria for PD | community | 34 | 70,5 | Not reported | internet-based CBT | There were 6 modules, consisting of psychoeducation, breathing retraining, working on their thought processes in relation to anxiety (to identify and reveal catastrophic interpretations of physical symptoms and then to produce alternative interpretations), interoceptive exposure, exposure in vivo, and reducing the risk of a relapse. Individual feedback was given within 24 hours of the participants sending their answers via e-mail. On the basis of these e-mails, an assessment was made to judge whether the participant was ready to continue; if so, the password to the next module was sent. If not, the participant received instructions on what needed to be completed to be able to get to the next step. | Guided self-help | 6 | WL | WL | BSQ | yes | post-treatment | None | yes | yes | High risk | yes |
| 16 | Carter, 2003 | USA | DSM-IV criteria for panic disorder with agoraphobia | community | 41 | 100 | 100 | Panic control therapy (PCT) | therapy consisted of providing a cognitive rationale that emphasized the importance of catastrophic cognitions in the genesis and maintenance of panic attacks. All participants were instructed on breathing retraining, where they were taught to slow their breathing and to breathe diaphragmatically. Participants also engaged in interoceptive exposure, systematically exposing themselves to the physiological sensations of anxiety via a number of predetermined exercises. | group | 11 | WL | WL | ASI | yes | 11 | None | no | no | High risk | yes |
| 17 | Choi, 2005 | East Asia | DSM-IV criteria for panic disorder with agoraphobia | Clinical | 36 | 50 | 100 | Experiential cognitive therapy (ExCT) | It integrated traditional cognitive behavior therapy (CBT) with virtual reality exposure for the treatment of panic disorder with agoraphobia | individual | 4 | / | / | - | - | post-treatment | 24 after treatment completion | Yes | unclear | Some concerns | No. study groups are not suitable for the network |
|  |  |  |  |  |  |  |  | Panic control therapy (PCT) | PCP consisted of several components, which were psychoeducation, breathing retraining and muscle relaxation training, cognitive restructuring, interoceptive exposure, and in vivo exposure. | individual | 12 |  |  |  |  |  |  |  |  |  |  |
| 18 | Christoforou, 2017 | UK | Agoraphobia | community | 39,5 | 59 | 100 | "Agoraphobia free" app | based on CBT principles, comprising psychoeducation, reflection, cognitive restructuring, interoceptive exposure, and systematic desensitization. | Unguided self-help | 10 | / | / | - | - | 12 | 6 | Unclear | yes | Some concerns | No. study groups are not suitable for the network |
|  |  |  |  |  |  |  |  | "Stress free" app | focused on teaching relaxation techniques and generic CBT skills though a virtual therapist. The app also includable a few distraction techniques presented in the form of games that required attention to help individuals cope with acute anxiety. | Unguided self-help | 10 |  |  |  |  |  |  |  |  |  |  |
| 19 | Ciuca, 2018 | Europe | DSM-IV panic disorder | community | 35,2 | 68 | 52 | Skype guided PAXPD | The therapy protocol addresses: psychoeducation on the disorder and means of intervention; techniques for decreasing neurophysiological hyperarousal; cognitive restructuring; exposure to feared somatic sensations, alongside with situational (in vivo) exposures to reduce agoraphobic avoidance; positive emotions training; problem-solving training; behavioral activation and cognitive restructuring exercises to reduce symptoms of depression; relapse prevention. During the sessions, the psychotherapist checked if the participant has completed and understood each module, answered questions and helped the participant carry out the recommended exercises. Remote, synchronous | Guided self-help | 16 | WL | WL | PDSS-SR | yes | post-treatment | 12,24 after treatment completion | yes | yes | Some concerns | yes |
|  |  |  |  |  |  |  |  | Skype unguided PAXPD | Same intervention, but unguided | Unguided self-help | 16 |  |  |  |  |  |  |  |  |  |  |
| 20 | Clark, 1999^33^ | UK | DSM-III-R criteria for panic disorder with no, mild, or moderate agoraphobic avoidance | Clinical | 34 | 62 | 85 | Brief cognitive therapy | The full intervention was the same as in the Clark et al. (1994) study and comprised a mixture of cognitive techniques and behavioral experiments, all intended to modify misinterpretations of body sensations and the processes that maintain them. | individual | 12 | WL | WL | ACQ | yes | post-treatment | 12, 48 after treatment completion | yes | no | High risk | yes |
|  |  |  |  |  |  |  |  | Full cognitive therapy | The brief intervention was a modified version of the full treatment. The same range of procedures was used, but many were first introduced in self-study modules. Patients read the self-study modules and completed the written exercises and the homework outlined in the modules before discussing a module's topic with their therapist. Patients studied a different module before each of the first four sessions. | individual | 5 |  |  |  |  |  |  |  |  |  |  |
| 21 | Craske, 2003^35^ | USA | DSM-IV principal diagnosis of panic disorder with or without agoraphobia | community | 34,6 | 60,3 | 82 | PCT + IV | Panic control therapy + in vivo exposure | Group | 16 | / | / | - | - | post-treatment | 24 after treatment completion | Yes | unclear | High risk | No. study groups are not suitable for the network |
|  |  |  |  |  |  |  |  | PCT | Panic control therapy alone | Group | 16 |  |  |  |  |  |  |  |  |  |  |
| 22 | Craske, 2007 | USA | DSM-IV criteria for a principal diagnosis of panic disorder with or without agoraphobia, and at least one comorbid anxiety or mood disorder | Mixed clinical and community | 36,8 | 60 | 50 | PDA | group and individual treatment sessions targeting panic disorder/agoraphobia | Group | 12 | / | / | - | - | post-treatment | 24,48 after treatment completion | Yes | no | Some concerns | No. study groups are not suitable for the network |
|  |  |  |  |  |  |  |  | PDA+C | group sessions targeting panic disorder/ agoraphobia, plus individual sessions targeted the most severe comorbid disorder | Group and individual | 12 group, 6 individual |  |  |  |  |  |  |  |  |  |  |
| 23 | Craske, 2005 | USA | DSM-IV criteria for a principal diagnosis of panic disorder with or without agoraphobia | Mixed clinical and community | 35,12 | 51 | 29,2 | CBT | CBT adapted for nocturnal panic | Individual | 11 | WL | WL | ASI | yes | post-treatment | 36 after treatment completion | Yes | no | High risk | yes |
| 24 | Craske, 2011 | USA | DSM-IV diagnoses of GAD, PD, SAD, or PTSD | clinical | Not specified for the PDA sub-sample | | | Coordinated Anxiety Learning and Management (CALM) | cognitive behaviour therapy and pharmacotherapy medication recommendations. Medication consultation was available from a local study psychiatrist who provided single-session medication management training | Individual | 4 | TAU | TAU | PDSS-SR | yes | 24 | 48,72 | Yes | no | High risk | Yes |
| 25 | Craske, 1997 | USA | Eligibility was dependent on a principal diagnosis of PDA, rated as 4 or more on a 0-8 point distress impairment scale | Mixed clinical and community | Not reported | 53 | 100 | cognitive therapy plus interoceptive exposure plus in vivo exposure (CIE) | cognitive restructuring, interoceptive exposure (i.e. repeated exposureto fearedbodily sensations)and in vivo exposure to agoraphobic situations | Group | 12 | / | / | - | - | post-treatment | 24 after treatment completion | Yes | no | High risk | No. study groups are not suitable for the network |
|  |  |  |  |  |  |  |  | cognitive therapy plus breathing retraining plus in vivo exposure (CBE) | cognitive restructuring, breathing retraining and in viuo exposure to agoraphobic situations | Group | 12 |  |  |  |  |  |  |  |  |  |  |
| 26 | de Beurs, 1995^43^ | Europe | DSM-III-R criteria for panic disorder with moderate or severe agoraphobia. | Mixed clinical and community | 38,8 | 75 | 100 | Fluvoxamine + exposure | *Not includable in the network* | - | - | Placebo + exposure | *Not includable in the network* | - | - | post-treatment | none | Yes | no | High risk | No. study groups are not suitable for the network |
|  |  |  |  |  |  |  |  | Panic management + exposure | The cognitive model of panic was presented , and there was an explanation of the vicious circle of bodily sensations and panic expectancy, which results in panic attacks and avoidance behaviour | individual | 12 |  |  |  |  |  |  |  |  |  |  |
|  |  |  |  |  |  |  |  | Exposure | *Not includable in the network* | - | - |  |  |  |  |  |  |  |  |  |  |
| 27 | de Ruiter, 1989 | Europe | panic disorder with agoraphobia (DSM-IIIR) | clinical | 34 | 49 | 100 | Breathing Retraining / cognitive Restrlicturing (BRCR) | Treatment consisted of: voluntary hyperventilation; explanation of how hyperventilation plus catastrophic cognitions cause panic attacks; relaxation training and training in slow breathing. | Individual | 8 | / | / | - | - | post-treatment | 24, 72 | Yes | No | High risk | No. study groups are not suitable for the network |
|  |  |  |  |  |  |  |  | BRCR + Exposure | Exposure therapy. Treatment consisted of graded self-exposure in vivo | Individual | 8 |  |  |  |  |  |  |  |  |  |  |
|  |  |  |  |  |  |  |  | Exposure | *Not includable in the network* | - | - |  |  |  |  |  |  |  |  |  |  |
| 28 | Erickson, 2007 | Canada | Diagnosis of either panic disorder with or without agoraphobia,OCD, social phobia, generalised anxiety disorder, specific phobia or PTSD. | clinical | Not specified for the PDA sub-sample | | | CBT | The content of the group sessions, summarized in the box on this page, was designed to include most common CBT elements, as well as many disorder-specific treatment elements. In general, the behavioral components were prominent in the first half of the protocol. In the second half, starting with the sixth session, the cognitive elements were prominent. | Group | 11 | WL | WL | BAI | yes | post-treatment | None | Yes | Unclear | High risk | yes |
| 29 | Fogliati, 2016 | Australia | DSM-IV criteria for a panic disorder with or without agoraphobia | Online application | 41 | 79 | Not reported | CBT | Participants in the clinician-guided condition (CG-CBT) received weekly contact via telephone or a secure email messaging system. The mean clinician time per participant in CG-CBT group was 36.79 minutes (SD = 21.35), which comprised answering and making calls (total calls = 453; range = 0–14 calls; mean time = 26.13; SD = 23.67), as well as reading, sending and responding to secure emails (total emails = 768; range = 0–12 emails; mean time = 10.67; SD = 8.57) | Guided self-help | 5 | / | / | PDSS-SR | yes | 8 | 12,48,96 | Yes | Yes | Low risk | yes |
|  |  |  |  |  |  |  |  |  | Participants in the self-guided condition did not receive weekly contact, but were monitored throughout treatment by the clinicians and were able to contact the clinic if technical assistance was required, or if they were experiencing a mental health crisis. The mean total clinician time per participant for SG-CBT was 5.5 min (SD = 1.88), which comprised answering and making calls (total calls = 2; range = 0–1 call; mean time = .11; SD = .83), as well as reading, sending and responding to secure emails (total emails = 10; range = 0–2 emails; mean time = .44 SD = 1.60). This contact was focused on assessing and managing mental health crises rather than the provision of treatment or course-related clinical support. | Unguided self-help | 5 |  |  |  |  |  |  |  |  |  |  |
| 30 | Gensichen, 2019 | Europe | adult patients diagnosed with PDA (ICD-10:F41.0 or F40.01) | Clinical | 46 | 74 | 75 | practice team–supported exposure training | The practice team–supported exposure training comprised evidence-based elements of CBT (psychoeducation, interoceptive and situational anxiety exposure exercises) as well as intervention elements from the chronic care model. | Individual | 4 | TAU | TAU | BAI | yes | 24 | 48 | Yes | Yes | Low risk | yes |
| 31 | Gloster, 2011 | Europe | DSM-IV TR for PD with agoraphobia | Clinical | 35,5 | 77 | 100 | CBT (T+ variant) | therapists planned and supervised exposure in situ exercises outside the therapy room. | Individual | 12 | WL | WL | PAS | no | post-treatment | 24 | yes | yes | Low risk | yes |
|  |  |  |  |  |  |  |  | CBT (T- variant) | T- group therapists planned and discussed patients’ in situ exposure exercises but did not accompany them. | Individual | 12 |  |  |  |  |  |  |  |  |  |  |
| 32 | Gould, 1993 | USA | DSM-III-R panic disorder | Community | 35,7 | 65 | 94 | Bibliotherapy (BT) | It focuses on (1) educating individuals about the etiology and nature of panic disorder; (2) teaching them a variety of cognitive and behavioral strategies that include relaxation, cognitive restructuring, breathing retraining, and exposure; and (3) advising them on how to implement these strategies. The book can be used either with or without the guidance of a therapist. Subjects were told that this treatment approach was designed to help them cope better with their panic attacks, and that they would be contacted at weeks 2 and 4 by the experimenter to assess their progress in reading the book. Each phone call lasted approximately 10 minutes, during which the experimenter followed a written protocol and did not answer questions about subject's treatment. Experimenters questioned subjects about specific material covered in the book to ascertain if subjects were reading the book and to determine their level of comprehension. | Unguided self-help | 0 | WL | WL | ASI | yes | 4 | none | Yes | no | High risk | yes |
|  |  |  |  |  |  |  |  | Individual therapy using Guided Imaginal Coping  (ITGIC) | These plans were derived primarily from material in Coping with Panic and were designed to treat panic using cognitive and behavioral approaches. | Individual | 8 |  |  |  |  |  |  |  |  |  |  |
| 33 | Hazen 1996 | USA | DSM-III-R diagnosis of panic disorder with or without agoraphobia | Clinical | 37,12 | 73,5 | 92,4 | self-help manual | The content of the treatment program includable psycheducational information about anxiety, and cognitive-behavioral treatment strategies, including relaxed breathing, progressive muscle relaxation, cognitive restructuring, and graduated exposure to feared situations. Subjects assigned to the self-help manual condition were instructed to complete one section of the self-help manual weekly for 14 weeks. | Unguided self-help | 0 | WL | WL | ASI | yes | post-treatment | None | Yes | no | High risk | yes |
|  |  |  |  |  |  |  |  | self-help group | to read and go through the manual without the guidance of a professional |  | 13 |  |  |  |  |  |  |  |  |  |  |
|  |  |  |  |  |  |  |  | professionally led group | Sessions were structured around the content of the self-help manual, and includable discussion of reading and practice homework assignments. | group | 13 |  |  |  |  |  |  |  |  |  |  |
| 34 | Hecker, 1996 | USA | DSM-III-R panic disorder | Community | 41 | 81,2 | 37,5 | self-directed CBT | clients were instructed to work through the Mastery of Your Anxiety and Panic (Barlow & Craske, 1989) workbook on their own. They met with a therapist four times over 12 weeks. No therapeutic interventions were delivered during these meetings. | Individual | 4 | / | / | - | - | post-treatment | 24 | Yes | no | High risk | No. study groups are not suitable for the network |
|  |  |  |  |  |  |  |  | therapist-directed CBT | the therapist and client worked through the material covered in the MAP workbook. Therapists used a treatment manual designed to accompany the manual to guide treatment sessions. | Individual | 12 |  |  |  |  |  |  |  |  |  |  |
| 35 | Hendriks, 2010 | Europe | DSM-IV criteria for PDor PD with agoraphobia | Clinical | 68,6 | 30 | 48 | CBT | The standardized programme comprised the following five components: (1) education about panic and anxiety, (2) relaxation techniques, (3) interoceptive exposure, (4) cognitive therapy and (5) exposure in vivo. | Individual | 14 | WL | WL | ACQ | yes | 14 | 8,26 | yes | Yes | Some concerns | yes |
|  |  |  |  |  |  |  |  | paroxetine | *Not includable in the network* | - | - |  |  |  |  |  |  |  |  |  |  |
| 36 | Kenardy, 2003 | Australia | DMS-IV criteria for panic disorder with or without agoraphobia | Clinical | 36,8 | 75,5 | 76,1 | CBT-12 | Standard treatment involved 12 weekly 1-hr individual sessions with the therapist. | Individual | 12 | WL | WL | ACQ | Yes | post-treatment | 24 | Yes | Yes | High risk | yes |
|  |  |  |  |  |  |  |  | CBT-6 | The 6-week treatment protocols, 6 sessions of therapist-delivered CBT. |  | 6 |  |  |  |  |  |  |  |  |  |  |
|  |  |  |  |  |  |  |  | Computer-augmented CBT6 (CBT-6-CA) | CBT6-CA, constituted a condensed version of the standard CBT12 regime, including individual sessions with the therapist, the same content, and the same supplementary handouts. |  | 6 |  |  |  |  |  |  |  |  |  |  |
| 37 | Kiropoulos, 2008 | Australia | DSM IV panic disorder with or without agoraphobia | community | 38,96 | 72,1 | 58 | CBT | During the first face-to-face therapy session, participants were given a copy of the manual ‘‘Mastery of Your Anxiety and Panic – Third Edition’’ (MAP-3; Barlow & Craske, 2000) free of charge. This manual presents the rationale for the 12 week CBT treatment and focuses on teaching participants a variety of cognitive and behavioral strategies that include controlled breathing, cognitive restructuring, and interoceptive and situational exposure similar to that used in the PO condition in this study. | Individual | 12 | / | / | PDSS | No | post-treatment | None | Yes | Yes | Some concerns | yes |
|  |  |  |  |  |  |  |  | Panic Online (PO) | PO is a structured program comprised of an introductory module, four learning modules, and a relapse prevention module. PO includable common treatment methods used in standard CBT for panic disorder (i.e., instructions for controlled breathing, cognitive restructuring, and interoceptive and situational exposure). Nine registered and one probationary psychologist (overall seven female and two male), all trained in CBT for PD, made contact via email with the PO treatment participants assigned to them and guided each participant through the internet-based program. All psychologists interacted with their participant via email, which allowed the psychologist to provide individualized support and feedback to the participant, according to the participants’ individual needs. | Guided self-help | 6 |  |  |  |  |  |  |  |  |  |  |
| 38 | Klein, 2009 | Australia | DSM IV panic disorder with or without agoraphobia | community | 39,49 | 82,4 | 56 | Panic Online x1 contact/week | This was a two-step Internet-based intervention. PO Step 1 consists of five online open-access modules containing psychoeducational information on PD. PO Step 2 is a password-protected PD CBT Internet-based treatment program comprising four learning modules and introductory and relapse prevention modules. Therapist interaction occurred via e-mail, enabling the therapist to provide support and feedback to participants and to guide them through the program. | Guided self-help | 6 | / | / | - | - | 8 | none | Yes | Yes | Some concerns | No. study groups are not suitable for the network |
|  |  |  |  |  |  |  |  | Panic Online x3 contacts/week |  | Guided self-help | 6 |  |  |  |  |  |  |  |  |  |  |
| 39 | Klein, 2001 | Australia | panic disorder by DMS-IV criteria. | community | 40 | 86,3 | Not reported | internet-based program | The first component of the Internet-based program focused on the nature, effects and causes of panic, and the second on useful and non-useful ways of managing panic. Negative self-statements were discussed as were errors in thinking. Brief techniques on how to overcome these cognitive errors were explained. This study incorporated some therapist-participant involvement in that the senior author needed to show participants how to access and navigate the Internet-based program, and to check that they were accessing it during the active treatment phase. More detailed instructions on the use of the program may have reduced this time but the optimal conditions for delivering these type of interventions require further intervention. | UnGuided self-help | Not reported | self-monitoring | TAU | Only acceptability analyzed | yes | post-treatment | none | Yes | unclear | High risk | yes |
| 40 | Klein, 2006 | Australia | DSM IV panic disorder with or without agoraphobia | community | Not reporter (between 18 an 70) | 80 | 82 | Panic Online | PO was a 6-week structured program comprised of an introductory module, four learning modules, and a relapse prevention module. The program includable common treatment methods used in standard CBT for PD (i.e., instructions for controlled breathing, cognitive restructuring, and interoceptive and situational exposure). Therapist interaction occurred via email, enabling the therapist to provide individualised support and feedback to the participant, according to their requests and needs. | Guided self-help | 6 | information only control | WL | ACQ | no | post-treatment | 12 | Yes | Yes | High risk | yes |
|  |  |  |  |  |  |  |  | manualized CBT workbook (MAN) | This manual presents the rationale for the treatment and focuses on teaching participants a variety of cognitive and behavioural strategies that include controlled breathing, cognitive restructuring, and interoceptive and situational exposure. These participants were instructed to work through this manual on their own; however, they were informed that a therapist would telephone them at home once weekly to assist them and monitor their progress. The telephone therapists were two Doctor of Psychology (Clinical) students (one male and one female) both trained in CBT for PD. The therapist was required to keep a log of amount of time spent on each MAN participant. |  | To read a book in 6 weeks |  |  |  |  |  |  |  |  |  |  |
| 41 | Klosko, 1990 | USA | DSM-III-R primary diagnosis of panic disorder with a clinician’s severity rating of at least 4 on a 0 to 8 scale (moderate severity) | Mixed clinical and community | 37 | 74 | 79 | Panic control treatment (PCT) | PCT is a manual-based 12–15-session cognitive– behavioral treatment protocol. The treatment includes education about the causes and maintenance of panic disorder, breathing retraining, cognitive restructuring, and interoceptive and agoraphobic exposure components. | Individual | 15 | Waiting list | WL | ADIS-R | no | post-treatment | none | Yes | no | High risk | yes |
|  |  |  |  |  |  |  |  | alprazolam | *Not includable in the network* | - | - | placebo | placebo |  |  |  |  |  |  |  |  |
| 42 | Koszycki, 2011 | Canada | DSM-IV criteria for PD with or without agoraphobia | Mixed clinical and community | 36,16 | 64,7 | 71 | self-administered CBT (SCBT) + placebo | SCBT consisted of 12 audiotapes and a workbook that contained monitoring forms for homework. Each tape described the principles of treatment and provided detailed instructions and homework. Treatment components includable extensive psychoeducation about anxiety and the cognitive model of PD, breathing retraining and relaxation skills, cognitive restructuring that addressed misappraisal of panic symptoms, interoceptive and situational exposure, and relapse prevention. | Unguided self-help | 12 audiotapes and a workbook | placebo | TAU | ACQ | Yes | 16 | 20,24 | Yes | Yes | Some concerns | yes |
|  |  |  |  |  |  |  |  | sertraline | *Not includable in the network* | - | - |  |  |  |  |  |  |  |  |  |  |
|  |  |  |  |  |  |  |  | SCBT + sertraline | *Not includable in the network* | - | - |  |  |  |  |  |  |  |  |  |  |
| 43 | Lidren, 1994 | USA | DSM-III-R criteria for PD with or without agoraphobia | Mixed clinical and community | 33,7 | 69,5 | 83,3 | Bibliotherapy | The bibliotherapy condition used Clum's (1990) Coping with Panic book. | Unguided self-help | read a book in 8 weeks | WL | WL | Panic attack symptoms questionnaire | yes | post-treatment | 12,24 | Yes | Unclear | High risk | yes |
|  |  |  |  |  |  |  |  | Group therapy | Subjects in this condition also used Clum's (1990) Coping with Panic text to ensure similarity of treatment techniques and, like those in the BT condition, completed weekly practice records. However, subjects in the GT condition met weekly for 90 min with a therapist in groups of six to process and practice material covered in the text. | Group | 8 |  |  |  |  |  |  |  |  |  |  |
| 44 | Loerch, 1999 | Europe | DSM-III-R criteria for PD with agoraphobia | Not reported | 35,13 | 74,5 | 100 | Placebo + CBT | Patients receiving CBT attended nine individual sessions for 50 minutes within eight weeks. In addition, two therapist-assisted exposure sessions with an average length of six hour day were administered on consecutive days in Week 3. | individual | 8 | Placebo + clinical management (described as “double placebo”) | APP | HAMA | yes | post-treatment | 4,12,24 | Yes | Yes | High risk | yes |
|  |  |  |  |  |  |  |  | Moclobemide + clinical management | *Not includable in the network* | - | - |  |  |  |  |  |  |  |  |  |  |
|  |  |  |  |  |  |  |  | Moclobemide + CBT | *Not includable in the network* | - | - |  |  |  |  |  |  |  |  |  |  |
| 45 | Marchand, 2008 | Canada | DSM-III-R criteria for PD with agoraphobia | Mixed clinical and community | 36,67 | 68,8 | 100 | Cognitive therapy + graded exposure | CT+graded exposure | Group | 14 | Placebo | Not includable in the network due to lack of data | ACQ | yes | post-treatment | 12,24,48 | Yes | No | High risk | yes |
|  |  |  |  |  |  |  |  | graded exposure (GE) | *Not includable in the network* | - | - |  |  |  |  |  |  |  |  |  |  |
|  |  |  |  |  |  |  |  | Cognitive therapy (CT) | *Not includable in the network* | - | - | supportive therapy (explicitly used as control condition) | APP |  |  |  |  |  |  |  |  |
|  |  |  |  |  |  |  |  | Imipramine | *Not includable in the network* | - | - |  |  |  |  |  |  |  |  |  |  |
| 46 | Marchand, 2007 | Canada | DSM IV panic disorder with agoraphobia | community | 35 | 73 | 100 | Standard CBT | 14-session of a standard cognitive-behavioural treatment | individual | 14 | / | / | - | - | post-treatment | 12,24 | No | No | High risk | No. study groups are not suitable for the network |
|  |  |  |  |  |  |  |  | Brief cognitive behaviour therapy alone (BCBT-A) | 7-session brief cognitive behaviour therapy alone, that is, without the help of a partner along with a self-study manual. | Individual | 7 |  |  |  |  |  |  |  |  |  |  |
|  |  |  |  |  |  |  |  | Brief cognitive behaviour therapy with partner (BCBT-P) | 7-session brief cognitive behaviour therapy involving a partner along with a self-study manual | individual | 7 |  |  |  |  |  |  |  |  |  |  |
| 47 | Meulenbeek, 2010 | Europe | People with subthreshold or mild panic disorder, defined as having symptoms of panic disorder falling below the cut-off of 13 on the PDSS–SR | community | 42 | 71 | 62 | ‘Don’t Panic’ course | The Don’t Panic’ course is based on cognitive– behavioural principles that have been shown to be effective in the treatment of the full-blown disorder. The course was developed specifically for adults. It consisted of 8 weekly sessions of 2 h each in groups of 6–12 participants. | Group | 8 | WL | WL | PDSS-SR | yes | 12 | 36 | Yes | Yes | Low risk | yes |
| 48 | Newman, 1997 | USA | DSM-III criteria for PD with or without agoraphobia | clinical | 38 | 83 | 70 | Standard CBT  (CBT12) | Treatment includable cognitive restructuring, breathing retraining, progressive muscle relaxation, exposure to interoceptive cues, and exposure to feared situations. | individual | 12 | / | / | - | - | 13 | 24 | Yes | No | High risk | No. study groups are not suitable for the network |
|  |  |  |  |  |  |  |  | Computer assisted CBT (CBT4-CA) | After the fourth therapy session, clients continued using the computer in the treatment-plus-diary mode for 8 weeks. In this way, CBT4-CA clients made use of the computer therapy program for the same amount of time the CBT12 clients were in treatment (12 weeks) | individual | 4 |  |  |  |  |  |  |  |  |  |  |
| 49 | Nordin, 2010 | Europe | Diagnostic criteria for panic disorder with or without agoraphobia | Community | 37,7 | 72,5 | 60 | bibliotherapy | During the 10-week self-help program, the participants were instructed to work on one chapter for 1 week before moving on to the next one. “Bibliotherapy without therapist input”. | Unguided self-help | Book, 10 chapters | WL | WL | PDSS | No | 10 | 12 | yes | yes | Some concerns | yes |
| 50 | Oh, 2020 | East Asia | diagnosis of panic disorder (with or without agoraphobia) based on the Mini- International Neuropsychiatric Interview (MINI) | clinical | 41 | 51 | Unclear | Chat bot APP | The chatbot was a newly-developed mobile service that provided a variety of information based on using AI and chat functions | Unguided self-help | 4 week access to the APP or to the book | / | / | - | - | 4 | None | Yes | No | Some concerns | No. study groups are not suitable for the network |
|  |  |  |  |  |  |  |  | bibliotherapy | The control group was provided with a paperback book entitled “Goodbye Panic Disorder” | Unguided self-help |  |  |  |  |  |  |  |  |  |  |  |
| 51 | Oromendia, 2016 | Europe | DSM-IV PD with or without agoraphobia | Community | 40,7 | 68,8 | Not reported | “Free from Anxiety” web program | Free from Anxiety is a transdiagnostic, Internet-based, self-help program for anxiety disorders. This program is presented as an interactive course with 8 modules, and it uses several therapeutic CBT components such as a psychoeducational section and a homework section with exercises to practice on a daily basis. During the treatment, four participants of the NPS group asked for help only once, and they were contacted by phone by a psychologist who spent a mean of 8.25 minutes (SD = 2.16) on each call. As the participants of this group practically did not ask for support, we could only analyze the effect of the psychologist’s time among the participants who received scheduled support (SPS). | Unguided self-help | 8 | WL | WL | PDSS-SR | yes | 8 | 24 | yes | yes | High risk | yes |
|  |  |  |  |  |  |  |  | “Free from Anxiety” web program +scheduled support | scheduled support: one phone call per week initiated by the therapist | Guided self-help | 8 |  |  |  |  |  |  |  |  |  |  |
| 52 | Ost, 2004 | Europe | DSM-IV criteria for panic disorder with agoraphobia | Community | 36,1 | 68,5 | 100 | Exposure | *Not includable in the network* | - | - | WL | WL | HAMA | No | post-treatment | 48 | Yes | Yes | Some concerns | yes |
|  |  |  |  |  |  |  |  | CBT | This method is based on the cognitive theory of panic of Beck  and Clark and makes use of both cognitive and behavioral techniques. | individual | 12 to 16 |  |  |  |  |  |  |  |  |  |  |
| 53 | Pelissolo, 2012 | Europe | DSM-IV PDA | clinical | 37 | 67,5 | 100 | VRET | *Not includable in the network* | - | - | WL | WL (not includable in the network: no data available) | - | - | 12 | 24,48 | No | No | High risk | No. no data. Impossible to reach out to the authors |
|  |  |  |  |  |  |  |  | CBT | Cognitive and behavior methods used were those classically recommended for PDA, and were highly structured and reproducible. Detailed manuals with guidelines for each session and checklists of the techniques were provided to the therapists and information sheets were given to the patient. | individual | 12 |  |  |  |  |  |  |  |  |  |  |
| 54 | Petterson, 1996 | USA | DSM-III-R diagnosis of panic disorder | Mixed clinical and community | 37,8 | 63 | Not reported | CBT | Treatment consisted of definition of panic disorder, description of predisposing factors, outline of the panic cycle, method of observation of panic, homework, cognitive-behavioural modelling, identification, modification of automatic thoughts, interoceptive conditioning, and introducing the concept of choice versus forced control. | individual | 6 | no treatment | TAU | ASI | yes | post-treatment | none | Yes | No | High risk | yes |
| 55 | Pitti, 2015 | Europe | ICD-10 for the diagnosis of agoraphobia with/without panic disorder | clinical | 39 | 70 | 100 | paroxetine+CBT (PX+CBT) | Standard CBT. | individual | 11 | / | / | ACQ | Yes | post-treatment | 24 | Yes | Unclear | High risk | yes |
|  |  |  |  |  |  |  |  | paroxetine+CBT+virtual exposure (PX+CBT+VRET) | The PXCBT-VRET group also underwent four 12-15 minute VR exposure, as part of exposure sessions. | individual | 11 |  |  |  |  |  |  |  |  |  |  |
|  |  |  |  |  |  |  |  | Paroxetine (PX) | The psychopharmacological treatment was paroxetine, at a mean dose of 22.60 mg/day. The dose was kept stable during the therapeutic process. | individual | 11 |  |  |  |  |  |  |  |  |  |  |
| 56 | Reinecke, 2013 | UK | DSM-IV diagnosis of panic disorder with or without agoraphobia | Not reported | 35,5 | 83,3 | 92,5 | exposure-based CBT | The treatment was a very condensed version of psychological intervention recommended for delivery in routine clinical care. It involved explanation of the learning mechanisms underlying the maintenance and treatment of panic (15 min), focusing on the role of safety strategies and exposure to an individually agoraphobic situation (stress test situation; 15 min) while dropping safety behaviour. | individual | 1 | WL | WL | ACQ | yes | day 2 | 4 | No | Unclear | High risk | yes |
| 57 | Richards, 2006 | Australia | DSM-IV PD with or without agoraphobia | Community | 36,6 | 68,5 | 78 | Internet-based CBT (PO1) | The program includable common treatment methods used in standard CBT for PD (i.e., instructions for controlled breathing, progressive muscle relaxation, cognitive restructuring and interoceptive and situational exposure). Three Doctor of Psychology (Clinical) students and one practising clinical psychologist, all experienced in CBT for PD made email contact with the Internet-based treatment (Panic Online: PO1) participants assigned to them and guided each through the program. PO1 comprised four learning modules and introductory and relapse prevention modules that were designed to be completed over 8 weeks. | Guided self-help | 6 modules | Internet-based information-only control condition | WL | ACQ | no | 8 | 12 | Yes | Yes | High risk | yes |
|  |  |  |  |  |  |  |  | Internet-based CBT plus stress management (P02) | PO2 was essentially the same as PO1 but it also contained a stress management program that includable six learning modules on coping with daily stresses, time and anger management, tuning into one’s thoughts, relaxation, and social connectedness. |  | 6 modules |  |  |  |  |  |  |  |  |  |  |
| 58 | Roberge, 2008 | Canada | DSM-IV criteria for panic disorder with agoraphobia | Mixed clinical and community | 39 | 79 | 100 | standard CBT | Treatment integrity was maintained using a structured and manualized treatment protocol adapted from Craske and Barlow’s (1993) Mastery of Anxiety and Panic Program. | individual | 14 | / | / | ACQ | yes | post-treatment | 12 | Yes | Yes | Some concerns | yes |
|  |  |  |  |  |  |  |  | brief CBT | Brief CBT includable seven 1-hour sessions with a therapist. Contrary to standard individual or group treatment, the therapist did not participate with the patient in the in vivo exposure. The goal of the sessions was to ensure that participants understood the material, practiced exercises, planned cognitive restructuring or exposure, and followed up on exercises and progress. |  | 7 |  |  |  |  |  |  |  |  |  |  |
|  |  |  |  |  |  |  |  | group CBT | Group CBT followed the same schedule as standard CBT. | group | 14 |  |  |  |  |  |  |  |  |  |  |
| 59 | Roy-Byrne, 2005 | USA | DSM-IV criteria for panic disorder | clinical | 41,2 | 67 | 100 | CBT modified for primary care setting. | Subjects were to complete the 6 CBT sessions within the first  3 months of the study. For subjects who were able to complete at least 3 sessions in person, subsequent sessions could be conducted over the telephone if preferred by the patient but had to be finished within 3 months. | individual | Up to 6 | TAU | TAU | ASI | yes | 12 | 24,48 | yes | yes | High risk | yes |
| 60 | Roy-Byrne, 2010 | USA | DSM-IV criteria for 1 or more of PD, GAD, SAD, or PTSD | clinical | Not specified for the PDA sub-sample | | | Coordinated Anxiety Learning and Management (CALM) | TheCBT program, a repackaging based on already validated CBT treatments,includable 5 generic modules (education, selfmonitoring, hierarchy development, breathing training, and relapse prevention) and 3 modules (cognitive restructuring and exposure to internal and external stimuli) tailored to the 4 specific anxiety disorders. | individual | 8 | TAU | TAU | BSI-12 | no | 24 | 48,72 | yes | yes | Low risk | yes |
| 61 | Ruwaard, 2010 | Europe | at least subsyndromal PD/A was established, according to DSM-IV | community | 38 | 72 | Not reported | WEB CBT | The treatment involves common CBT strategies for panic  disorder, such as psycho-education, awareness training, applied relaxation, cognitive restructuring and (interoceptive) exposure techniques. At specific occasions indicated in the manual, therapists post feedback and further instructions on the basis of the contents of this workbook. Therapists take about 20–40 min to read a client’s assignment, and to prepare feedback. The manual includes 14 of these feedback moments, so that a full treatment requires between 5 and 9 h of therapist time. | Guided self-help | 7 modules | WL | WL | PDSS-SR | yes | 13 | none | yes | Yes | Some concerns | yes |
| 62 | Schmidt, 1997a | USA | DSM-IV PD with or without agoraphobia | clinical | 31.8 | 54 | 58 | CBT | The treatment protocol includes four major components: (1) psychoeducation, (2) cognitive therapy techniques aimed at helping the patient to identify and alter faulty appraisals of threat that contribute to panic occurrence, (3) interoceptive exposure and (4) in vivo exposure | group | 12 | WL | WL | - | - | 9 | 21 | Yes | no | High risk | No, due to lack of data |
| 63 | Schmidt, 1997b | USA | DSM-IV diagnosis of panic disorder | clinical | 36 | 67 | Not reported | CBT | The treatment protocol includes four major components: (1) psychoeducation, (2) cognitive therapy techniques aimed at helping the patient to identify and alter faulty appraisals of threat that contribute to panic occurrence, (3) interoceptive exposure and (4) in vivo exposure. | group | 12 | WL | WL | ASI | yes | 12 | none | Yes | no | High risk | yes |
|  |  |  |  |  |  |  |  | CBT + respiratory training | Those assigned to the CBT-R group also received respiratory training techniques (i.e. diaphragmatic breathing). |  |  |  |  |  |  |  |  |  |  |  |  |
| 64 | Sharp, 1997 | UK | DSM-III PD with or without agoraphobia | clinical | 37,4 | 77,1 | Not reported | fluvoxamine | *Not includable in the network* | - | - | placebo | placebo | GHQ | yes | 12 | 24 | Yes | No | High risk | yes |
|  |  |  |  |  |  |  |  | CBT | The CBT employed emphasized both gross exposure techniques and cognitive and behavioural panic management techniques. The approach in treatment was similar to that of Barlow and co-workers,20,21 emphasizing the altering of action tendencies associated with panic, and also the hypervigilant and avoidant information-processing strategies and behaviours typical of patients with panic disorder and agoraphobia. | individual | 9 |  |  |  |  |  |  |  |  |  |  |
|  |  |  |  |  |  |  |  | CBT+placebo | *Not includable in the network* | - | - |  |  |  |  |  |  |  |  |  |  |
|  |  |  |  |  |  |  |  | fluvoxamine+CBT | *Not includable in the network* | - | - |  |  |  |  |  |  |  |  |  |  |
| 65 | Sharp, 2000 | UK | DSM-III PD with or without agoraphobia | clinical | 37,5 | Not reported | Not reported | CBT (standard) | Patients in the standard contact condition received the standard treatment manual and eight sessions of 45 minutes’ duration over 12 weeks with sessions at Days 0, 7, 14, 28, 42, 56, 70, and 84; a total of six hours’ therapist contact. | individual | 8 | / | / | global symptom severity scale | No | 21 | none | Yes | Unclear | Some concerns | yes |
|  |  |  |  |  |  |  |  | CBT (minimum contact) | Patients in the minimum contact condition received the treatment manual and six sessions, with sessions involving assessments (at Days 0, 42, and 84) being 30 minutes’ duration and the other sessions (at Days 7, 21, and 63) being 10 minutes’ duration; a total of two hours’ therapist contact. |  | 6 |  |  |  |  |  |  |  |  |  |  |
|  |  |  |  |  |  |  |  | bibliotherapy | Patients in the bibliotherapy condition received the treatment manual and assessment sessions at Days 0, 42, and 84. The one hour and 30 minutes of therapist contact in this condition was for assessment only, with treatment instruction provided solely by the treatment manual. | Unguided self-help | A book Manual |  |  |  |  |  |  |  |  |  |  |
| 66 | Sharp, 2004 | UK | DSM-IV PD with or without agoraphobia | clinical | 38,3 | Not reported | Not reported | Group CBT | Patients in the group treatment were randomly allocated to closed groups of six to eight participants. Groups met for 1-h sessions over 12 weeks with sessions on days 0, 7, 14, 28, 42, 56, 70, and 84. | group | 8 | WL | WL | HAMA | no | 21 | 12 | Yes | yes | High risk | yes |
|  |  |  |  |  |  |  |  | Individual CBT | Patients in the individual treatment group received individual treatment to exactly the same schedule as the group treatments that is, sessions on day 0, 7, 14, 28, 42, 56, 70, and 84. | individual | 8 |  |  |  |  |  |  |  |  |  |  |
| 67 | Shear, 2001 | USA | DSM-IV criteria for panic disorder with no more than mild agoraphobia | Mixed clinical and community | 36,15 | 63,7 | Not reported | emotion-focused psychotherapy | *Not includable in the network* | - | - | placebo | Placebo | PDSS | no | post-treatment | none | No | Yes | Some concerns | yes |
|  |  |  |  |  |  |  |  | imipramine | *Not includable in the network* | - | - |  |  |  |  |  |  |  |  |  |  |
|  |  |  |  |  |  |  |  | CBT | Cognitive behavior therapy targeted fear of bodily sensations. A patient handout contained information about anxiety and panic attacks and includable a presentation of the fear of bodily sensations model used in this treatment. | individual | 12 |  |  |  |  |  |  |  |  |  |  |
| 68 | Silfvernagel, 2012 | Europe | The participants had to have reoccurring panic attacks to be includable. | community | 32,4 | 65 | 83 | Internet CBT | cognitive restructuring (2 modules); panic disorder (2 modules); agoraphobia (1 module); generalized anxiety (3 modules); social anxiety (2 modules); behavioral activation (2 modules); applied relaxation (1 module); stress (1 module); mindfulness (1 module); problem solving (1 module); and insomnia (1 module). Therapist guidance was included in the trial, since it has been found to improve outcomes when compared with most unguided treatments | Guided self-help | 19 modules | WL | WL | PDSS | no | 10 | 48 | yes | Yes | Some concerns | yes |
| 69 | Telch, 1993 | USA | DSM-III-R criteria for panic disorder with or without agoraphobia | Mixed clinical and community | 34,6 | 73,1 | Not reported | CBT | The treatment consisted of education and corrective information; cognitive therapy; training in diaphragmatic breathing; and interoceptive exposure. | group | 12 | WL | WL | ASI | yes | post-treatment | none | yes | unclear | Some concerns | yes |
| 70 | Titov, 2010 | Australia | DSM-IV diagnostic criteria for GAD, social phobia, and/or panic disorder. | community | Not specified for the PDA sub-sample | | | iCBT - the Anxiety program | A transdiagnostic Internet based cognitive behavioural treatment program. One clinical psychologist (ER) provided all clinical contact with treatment group participants and another clinical psychologist (JS) provided all clinical contact with the control group. A research assistant provided administrative support to collate data. Every contact with each participant was recorded as was the total therapist time spent per participant. Therapists were encouraged to actively engage with participants in treatment, but advised to limit weekly contact time to approximately 10 min per participant, except if more time was clinically indicated. | Guided self-help | 6 modules | WL | WL | PDSS-SR | yes | post-treatment | 12 | yes | yes | Some concerns | yes |
| 71 | Tyrer, 1988^128^ | UK | DSM-III diagnosis of generalised anxiety disorder, panic disorder or dysthymic disorder | unclear | Not specified for the PDA sub-sample | | | CBT | Patients had five one-hour treatments. relaxation training was includable and the cognitive therapy includable records of dysfunctional thoughts and activity schedules.2O,21 Behavioural diaries were also completed and patients were trained to cope with attacks of anxiety and panic without reinforcement of these symptoms or avoidance behaviour. | individual | 5 | placebo | placebo | - | - | 10 | 2,4,6 | Yes | no | High risk | No, due to lack of data |
|  |  |  |  |  |  |  |  | Self-help treatment package | Patients allocated to the self-help treatment package saw the same team of community nurses at the same treatment intervals, but on each occasion were seen for 15 minutes only, given a relaxation tape vijthout any specific training, and given a list of self-help organisations and groups for people with anxiety and depression. Patients in this group were told initially that drugs were potentially therapist, dangerous because of dependence and that individual psychotherapy could lead to similar dependence on a therefore it was wise to avoid these where possible and to work out one’s own treatment programme. Both cognitive and behaviour therapy and the self-help treatment package were completed after these five sessions. | - | - |  |  |  |  |  |  |  |  |  |  |
|  |  |  |  |  |  |  |  | Diazepam | *Not includable in the network* | - | - |  |  |  |  |  |  |  |  |  |  |
|  |  |  |  |  |  |  |  | Dothiepin | *Not includable in the network* | - | - |  |  |  |  |  |  |  |  |  |  |
| 72 | van Ballegooijen, 2013 | Europe | 97 (77.0%) of the includable participants met the DSM-IV criteria for PD with or without agoraphobia. | community | 36,6 | 67,4 | 63 | Don’t Panic Online | The course consists of 6 sessions in which the participants learn to control their panic symptoms by applying various cognitive and behavioral techniques and skills. The participants in the intervention group were coached by trained, Master’s-level clinical psychology students. Every week, these participants received an email from their coach, asking how they were doing and whether they were experiencing any difficulty in following the program. The coaches responded to questions about the course and the associated exercises. They also gave brief replies to questions about the participant’s mental health. The coaches were supervised by the first author. On average, the total time spent on each participant was 1 to 2 hours | Guided self-help | 6 | WL | WL | PDSS-SR | yes | 12 | None | yes | yes | High risk | Yes |
| 73 | Williams, 1996 | USA | DSM-III-R diagnosis of panic disorder with or without agoraphobia | Community | 38 | 87,5 | 92 | Cognitive therapy | *Not includable in the network* | - | - | Delayed-treatment control | WL | ACQ | yes | post-treatment | 6 | yes | Unclear | High risk | yes |
|  |  |  |  |  |  |  |  | performance treatment | *Not includable in the network* | - | - |  |  |  |  |  |  |  |  |  |  |
|  |  |  |  |  |  |  |  | combined treatment | Subjects in the combined cognitive-plus-performance condition were treated using the same techniques given the groups receiving these treatments separately, but with a condensed amount of time and less elaboration on each treatment component, as required to keep the total treatment time constant across all three treatments. | individual | 8 |  |  |  |  |  |  |  |  |  |  |
| 74 | Wims, 2010 | Australia | DSM-IV criteria for panic disorder with or without agoraphobia | Community | 42 | 76 | 100 | Panic program | six online lessons, homework assignments, participation in an online discussion forum, and regular email contact with a mental health clinician. After completing each lesson participants were emailed by the therapist. The themes of the therapist’s emails varied from reinforcement for continued participation and efforts, encouragement to practice the relevant treatment skills, encouragement to complete lessons and homework assignments, enquiries about progress, and responses to questions. | Guided self-help | 6 modules | WL | WL | PDSS | no | post-treatment | 4 | Yes | Yes | High risk | yes |

*the definition of brief supportive therapy as given in the manuscript (“by taking a nondirective approach, the therapist did not apply specific interventions focused on alleviating panic symptoms”) fits the protocol requirements for the waiting list group (“Participants receive assessment, with or without simple provision of informational material or minimal therapist contact, or both, and they know that they will receive the active treatment in question after the waiting phase”).

ACQ: Agoraphobia cognition questionnaire; AD: antidepressant; APP: Attention or psychological placebo; ASI: Anxiety sensitivity index; BAI: Beck anxiety inventory; BSI: Brief Symptom Inventory; BSQ: The Body Sensations Questionnaire; BZP: benzodiazepine; CBT: Cognitive Behavioural Therapy; CGI: Clinical Global Impression; CGI-S: Clinical Global Impression - Severity of Illness; DSM: Diagnostic and Statistical Manual of Mental Disorders; HAMA: Hamilton Rating Scale for Anxiety; ICD: International Classification of Diseases; NT: no treatment; PAS: the Panic and Agoraphobia Scale; PD: Panic disorder; PDA: Panic disorder with agoraphobia; PDSS: Panic Disorder Severity Scale; PDSS-SR: Panic Disorder Severity Scale, Self-Report version; SSRI: serotonin selective reuptake inhibitor; TAU: Treatment as usual; TCA: tricyclic antidepressant; UK: United Kingdom; USA: United States of America. WL: waiting list.

### Supplement F - Differences between protocol and review

The differences between this review and its registered protocol are:

1. as the dropout rate was higher than what we expected we used the relative risk instead of the odds ratio to evaluate acceptability (binary outcome).
2. Heterogeneity was evaluated through 𝜏^2^ instead of I^2^
3. We added two nodes: remote synchronous face-to-face individual and remote synchronous face-to-face group.
4. We considered the “digital assisted” as “guided self-help” or “unguided self-help” according to the presence o absence of therapist guidance

**Supplement G** **- Trials included in the systematic review**

1. Addis ME, Hatgis C, Krasnow AD, Jacob K, Bourne L, Mansfield A. Effectiveness of cognitive--behavioral treatment for panic disorder versus treatment as usual in a managed care setting. *J Consult Clin Psychol* 2004; **72**(4): 625-35.

2. Allen AR, Newby JM, Mackenzie A, et al. Internet cognitive-behavioural treatment for panic disorder: randomised controlled trial and evidence of effectiveness in primary care. *BJPsych Open* 2016; **2**(2): 154-62.

3. Bakker A, van Dyck R, Spinhoven P, van Balkom AJ. Paroxetine, clomipramine, and cognitive therapy in the treatment of panic disorder. *J Clin Psychiatry* 1999; **60**(12): 831-8.

4. Barlow DH, Gorman JM, Shear MK, Woods SW. Cognitive-behavioral therapy, imipramine, or their combination for panic disorder: A randomized controlled trial. *JAMA* 2000; **283**(19): 2529-36.

5. Barlow DH CM, Cerny JA, Klosko JS. Behavioral treatment of panic disorder. *Behav Ther* 1989; **20**(2): 261-82.

6. Beck AT, Sokol L, Clark DA, Berchick R, Wright F. A crossover study of focused cognitive therapy for panic disorder. *Am J Psychiatry* 1992; **149**(6): 778-83.

7. Berger T, Urech A, Krieger T, et al. Effects of a transdiagnostic unguided Internet intervention ('velibra') for anxiety disorders in primary care: results of a randomized controlled trial. *Psychol Med* 2017; **47**(1): 67-80.

8. Bergstrom J, Andersson G, Ljotsson B, et al. Internet-versus group-administered cognitive behaviour therapy for panic disorder in a psychiatric setting: a randomised trial. *BMC Psychiatry* 2010; **10**: 54.

9. Bohni MK, Spindler H, Arendt M, Hougaard E, Rosenberg NK. A randomized study of massed three-week cognitive behavioural therapy schedule for panic disorder. *Acta Psychiatr Scand* 2009; **120**(3): 187-95.

10. Botella C, García-Palacios A. The possibility of reducing therapist contact and total length of therapy in the treatment of panic disorder. *Behav Cogn Psychother* 1999; **27**(3): 231-47.

11. Botella C, García-Palacios A, Villa H, et al. Virtual reality exposure in the treatment of panic disorder and agoraphobia: A controlled study. *Clin Psychol Psychother* 2007; **14**(3): 164-75.

12. Brown GK, Beck AT, Newman CF, Beck JS, Tran GQ. A comparison of focused and standard cognitive therapy for panic disorder. *J Anxiety Disord* 1997; **11**(3): 329-45.

13. Carlbring P, Bohman S, Brunt S, et al. Remote treatment of panic disorder: a randomized trial of internet-based cognitive behavior therapy supplemented with telephone calls. *Am J Psychiatry* 2006; **163**(12): 2119-25.

14. Carlbring P, Nilsson-Ihrfelt E, Waara J, et al. Treatment of panic disorder: live therapy vs. self-help via the Internet. *Behav Res Ther* 2005; **43**(10): 1321-33.

15. Carlbring P, Westling BE, Ljungstrand P, Ekselius L, Andersson G. Treatment of panic disorder via the Internet: A randomized trial of a self-help program. *Behav Ther* 2001; **32**(4): 751-64.

16. Carter MM, Sbrocco T, Gore KL, Marin NW, Lewis EL. Cognitive-behavioral group therapy versus a wait-list control in the treatment of African American women with panic disorder. *Cognit Ther Res* 2003; **27**(5): 505‐18.

17. Choi YH, Vincelli F, Riva G, Wiederhold BK, Lee JH, Park KH. Effects of group experiential cognitive therapy for the treatment of panic disorder with agoraphobia. *Cyberpsychol Behav* 2005; **8**(4): 387-93.

18. Christoforou M, Fonseca JAS, Tsakanikos E. Two novel cognitive behavioral therapy–based mobile apps for agoraphobia: Randomized controlled trial. *J Med Internet Res* 2017; **19**(11).

19. Ciuca AM, Berger T, Crişan LG, Miclea M. Internet-based treatment for panic disorder: A three-arm randomized controlled trial comparing guided (via real-time video sessions) with unguided self-help treatment and a waitlist control. PAXPD study results. *J Anxiety Disord* 2018; **56**: 43-55.

20. Clark DM, Salkovskis PM, Hackmann A, Wells A, Ludgate J, Gelder M. Brief cognitive therapy for panic disorder: a randomized controlled trial. *J Consult Clin Psychol* 1999; **67**(4): 583-9.

21. Craske MG, DeCola JP, Sachs AD, Pontillo DC. Panic control treatment for agoraphobia. *J Anxiety Disord* 2003; **17**(3): 321-33.

22. Craske MG, Farchione TJ, Allen LB, Barrios V, Stoyanova M, Rose R. Cognitive behavioral therapy for panic disorder and comorbidity: more of the same or less of more? *Behav Res Ther* 2007; **45**(6): 1095-109.

23. Craske MG, Lang AJ, Aikins D, Mystkowski JL. Cognitive behavioral therapy for nocturnal panic. *Behav Ther* 2005; **36**(1): 43‐54.

24. Craske MG, Rowe M, Lewin M, Noriega-Dimitri R. Interoceptive exposure versus breathing retraining within cognitive-behavioural therapy for panic disorder with agoraphobia. *Br J Clin Psychol* 1997; **36 ( Pt 1)**: 85-99.

25. Craske MG, Stein MB, Sullivan G, et al. Disorder-specific impact of coordinated anxiety learning and management treatment for anxiety disorders in primary care. *Arch Gen Psychiatry* 2011; **68**(4): 378-88.

26. de Beurs E, van Balkom AJ, Lange A, Koele P, van Dyck R. Treatment of panic disorder with agoraphobia: comparison of fluvoxamine, placebo, and psychological panic management combined with exposure and of exposure in vivo alone. *Am J Psychiatry* 1995; **152**(5): 683-91.

27. de Ruiter C, Ryken H, Garssen B, Kraaimaat F. Breathing retraining, exposure and a combination of both, in the treatment of panic disorder with agoraphobia. *Behav Res Ther* 1989; **27**(6): 647-55.

28. Erickson DH, Janeck AS, Tallman K. A cognitive-behavioral group for patients with various anxiety disorders. *Psychiatr Serv* 2007; **58**(9): 1205-11.

29. Fogliati VJ, Dear BF, Staples LG, et al. Disorder-specific versus transdiagnostic and clinician-guided versus self-guided internet-delivered treatment for panic disorder and comorbid disorders: A randomized controlled trial. *J Anxiety Disord* 2016; **39**: 88-102.

30. Gensichen J, Hiller TS, Breitbart J, et al. Panic Disorder in Primary Care. *Deutsches Arzteblatt international* 2019; **116**(10): 159-66.

31. Gloster AT, Wittchen HU, Einsle F, et al. Psychological treatment for panic disorder with agoraphobia: a randomized controlled trial to examine the role of therapist-guided exposure in situ in CBT. *J Consult Clin Psychol* 2011; **79**(3): 406-20.

32. Gould RA, Clum GA, Shapiro D. The use of bibliotherapy in the treatment of panic: A preliminary investigation. *Behav Ther* 1993; **24**(2): 241-52.

33. Hazen AL, Walker JR, Eldridge GD. Anxiety sensitivity and treatment outcome in panic disorder. 1996; **2**(1): 34-9.

34. Hecker JE, Losee MC, Fritzler BK, Fink CM. Self-directed versus therapist-directed cognitive behavioral treatment for panic disorder. *J Anxiety Disord* 1996; **10**(4): 253-65.

35. Hendriks GJ, Keijsers GP, Kampman M, et al. A randomized controlled study of paroxetine and cognitive-behavioural therapy for late-life panic disorder. *Acta Psychiatr Scand* 2010; **122**(1): 11-9.

36. Kenardy JA, Dow MG, Johnston DW, Newman MG, Thomson A, Taylor CB. A comparison of delivery methods of cognitive-behavioral therapy for panic disorder: an international multicenter trial. *J Consult Clin Psychol* 2003; **71**(6): 1068-75.

37. Kiropoulos LA, Klein B, Austin DW, et al. Is internet-based CBT for panic disorder and agoraphobia as effective as face-to-face CBT? *J Anxiety Disord* 2008; **22**(8): 1273-84.

38. Klein B, Austin D, Pier C, et al. Internet-based treatment for panic disorder: does frequency of therapist contact make a difference? *Cogn Behav Ther* 2009; **38**(2): 100-13.

39. Klein B, Richards JC. A brief internet-based treatment for panic disorder. *Behav Cogn Psychother* 2001; **29**(1): 113-7.

40. Klein B, Richards JC, Austin DW. Efficacy of internet therapy for panic disorder. *J Behav Ther Exp Psychiatry* 2006; **37**(3): 213-38.

41. Klosko JS, Barlow DH, Tassinari R, Cerny JA. A comparison of alprazolam and behavior therapy in treatment of panic disorder. *J Consult Clin Psychol* 1990; **58**(1): 77-84.

42. Koszycki D, Taljaard M, Segal Z, Bradwejn J. A randomized trial of sertraline, self-administered cognitive behavior therapy, and their combination for panic disorder. *Psychol Med* 2011; **41**(2): 373-83.

43. Lidren DM, Watkins PL, Gould RA, Clum GA, Asterino M, Tulloch HL. A comparison of bibliotherapy and group therapy in the treatment of panic disorder. *J Consult Clin Psychol* 1994; **62**(4): 865-9.

44. Loerch B, Graf-Morgenstern M, Hautzinger M, et al. Randomised placebo-controlled trial of moclobemide, cognitive-behavioural therapy and their combination in panic disorder with agoraphobia. *Br J Psychiatry* 1999; **174**: 205-12.

45. Marchand A, Coutu MF, Dupuis G, et al. Treatment of panic disorder with agoraphobia: randomized placebo-controlled trial of four psychosocial treatments combined with imipramine or placebo. *Cogn Behav Ther* 2008; **37**(3): 146-59.

46. Marchand A, Todorov C, Borgeat F, Pelland M-È. Effectiveness of a brief cognitive behavioural therapy for panic disorder with agoraphobia and the impact of partner involvement. *Behav Cogn Psychother* 2007; **35**(5): 613-29.

47. Meulenbeek P, Willemse G, Smit F, van Balkom A, Spinhoven P, Cuijpers P. Early intervention in panic: pragmatic randomised controlled trial. *Br J Psychiatry* 2010; **196**(4): 326-31.

48. Newman MG, Kenardy J, Herman S, Taylor CB. Comparison of palmtop-computer-assisted brief cognitive-behavioral treatment to cognitive-behavioral treatment for panic disorder. *J Consult Clin Psychol* 1997; **65**(1): 178-83.

49. Nordin S, Carlbring P, Cuijpers P, Andersson G. Expanding the limits of bibliotherapy for panic disorder: randomized trial of self-help without support but with a clear deadline. *Behav Ther* 2010; **41**(3): 267-76.

50. Oh J, Jang S, Kim H, Kim JJ. Efficacy of mobile app-based interactive cognitive behavioral therapy using a chatbot for panic disorder. *Int J Med Inform* 2020; **140**: 104171.

51. Oromendia P, Orrego J, Bonillo A, Molinuevo B. Internet-based self-help treatment for panic disorder: a randomized controlled trial comparing mandatory versus optional complementary psychological support. *Cogn Behav Ther* 2016; **45**(4): 270-86.

52. Ost LG, Thulin U, Ramnero J. Cognitive behavior therapy vs exposure in vivo in the treatment of panic disorder with agoraphobia (corrected from agrophobia). *Behav Res Ther* 2004; **42**(10): 1105-27.

53. Pelissolo A, Zaoui M, Aguayo G, et al. Virtual reality exposure therapy versus cognitive behavior therapy for panic disorder with agoraphobia: a randomized comparison study. *J Cyber Ther Rehabil* 2012; **5**(1): 35‐43.

54. Petterson K, Cesare S. Panic disorder: A cognitive-behavioural approach to treatment. *Couns Psychol Q* 1996; **9**(2): 191-201.

55. Pitti CT, Penate W, de la Fuente J, et al. The combined use of virtual reality exposure in the treatment of agoraphobia. *Actas Esp Psiquiatr* 2015; **43**(4): 133-41.

56. Reinecke A, Waldenmaier L, Cooper MJ, Harmer CJ. Changes in automatic threat processing precede and predict clinical changes with exposure-based cognitive-behavior therapy for panic disorder. *Biol Psychiatry* 2013; **73**(11): 1064-70.

57. Richards JC, Klein B, Austin DW. Internet cognitive behavioural therapy for panic disorder: does the inclusion of stress management information improve end-state functioning? *Clin Psychol* 2006; **10**(1): 2‐15.

58. Roberge P, Marchand A, Reinharz D, Savard P. Cognitive-behavioral treatment for panic disorder with agoraphobia: a randomized, controlled trial and cost-effectiveness analysis. *Behav Modif* 2008; **32**(3): 333-51.

59. Roy-Byrne P, Craske MG, Sullivan G, et al. Delivery of evidence-based treatment for multiple anxiety disorders in primary care: a randomized controlled trial. *JAMA* 2010; **303**(19): 1921-8.

60. Roy-Byrne PP, Craske MG, Stein MB, et al. A randomized effectiveness trial of cognitive-behavioral therapy and medication for primary care panic disorder. *Arch Gen Psychiatry* 2005; **62**(3): 290-8.

61. Ruwaard J, Broeksteeg J, Schrieken B, Emmelkamp P, Lange A. Web-based therapist-assisted cognitive behavioral treatment of panic symptoms: a randomized controlled trial with a three-year follow-up. *J Anxiety Disord* 2010; **24**(4): 387-96.

62. Schmidt NB, Staab JP, Trakowski JH, Jr., Sammons M. Efficacy of a brief psychosocial treatment for panic disorder in an active duty sample: implications for military readiness. *Mil Med* 1997; **162**(2): 123-9.

63. Schmidt NB, Trakowski JH, Staab JP. Extinction of panicogenic effects of a 35% CO2 challenge in patients with panic disorder. *J Abnorm Psychol* 1997; **106**(4): 630-8.

64. Sharp DM, Power KG, Simpson RJ, Swanson V, Anstee JA. Global measures of outcome in a controlled comparison of pharmacological and psychological treatment of panic disorder and agoraphobia in primary care. *Br J Gen Pract* 1997; **47**(416): 150-5.

65. Sharp DM, Power KG, Swanson V. Reducing therapist contact in cognitive behaviour therapy for panic disorder and agoraphobia in primary care: global measures of outcome in a randomised controlled trial. *Br J Gen Pract* 2000; **50**(461): 963-8.

66. Sharp DM, Power KG, Swanson V. A comparison of the efficacy and acceptability of group versus individual cognitive behaviour therapy in the treatment of panic disorder and agoraphobia in primary care. *Clinical Psychology and Psychotherapy* 2004; **11**(2): 73-82.

67. Shear MK, Houck P, Greeno C, Masters S. Emotion-focused psychotherapy for patients with panic disorder. *Am J Psychiatry* 2001; **158**(12): 1993-8.

68. Silfvernagel K, Carlbring P, Kabo J, et al. Individually tailored internet-based treatment for young adults and adults with panic attacks: randomized controlled trial. *J Med Internet Res* 2012; **14**(3): e65.

69. Telch MJ, Lucas JA, Schmidt NB, Hanna HH, LaNae Jaimez T, Lucas RA. Group cognitive-behavioral treatment of panic disorder. *Behav Res Ther* 1993; **31**(3): 279-87.

70. Titov N, Andrews G, Johnston L, Robinson E, Spence J. Transdiagnostic Internet treatment for anxiety disorders: A randomized controlled trial. *Behav Res Ther* 2010; **48**(9): 890-9.

71. Tyrer P, Seivewright N, Murphy S, et al. The Nottingham study of neurotic disorder: comparison of drug and psychological treatments. *Lancet* 1988; **2**(8605): 235-40.

72. van Ballegooijen W, Riper H, Klein B, et al. An Internet-based guided self-help intervention for panic symptoms: randomized controlled trial. *J Med Internet Res* 2013; **15**(7): e154.

73. Williams SL, Falbo J. Cognitive and performance-based treatments for panic attacks in people with varying degrees of agoraphobic disability. *Behav Res Ther* 1996; **34**(3): 253-64.

74. Wims E, Titov N, Andrews G, Choi I. Clinician-assisted Internet-based treatment is effective for panic: A randomized controlled trial. *Aust N Z J Psychiatry* 2010; **44**(7): 599-607.

**Supplement H** **- Trials excluded from the systematic review, with reason**

**Appendix H** **- Trials excluded from the systematic review, with reasons**

Protocols (n=8)

Follow-up studies / secondary analyses (n=12)

Addis, M. E., Hatgis, C., Cardemil, E., Jacob, K., Krasnow, A. D., & Mansfield, A. (2006). Effectiveness of cognitive-behavioral treatment for panic disorder versus treatment as usual in a managed care setting: 2-Year follow-up. *Journal of Consulting and Clinical Psychology, 74*, 377-85.

Biondi, M., & Picardi, A. (2003). Increased probability of remaining in remission from panic disorder with agoraphobia after drug treatment in patients who received concurrent cognitive-behavioural therapy: A follow-up study. *Psychotherapy and Psychosomatics, 72*, 34-42.

Bryant, R. A., Moulds, M. L., Nixon, R. D. V., Mastrodomenico, J., Felmingham, K., & Hopwood, S. (2006). Hypnotherapy and cognitive behaviour therapy of acute stress disorder: A 3-year follow-up. *Behaviour Research and Therapy, 44*, 1331-35.

Buckner, J. D., & Schmidt, N. B. (2009). A randomized pilot study of motivation enhancement therapy to increase utilization of cognitive-behavioral therapy for social anxiety. *Behaviour Research and Therapy, 47*, 710-5.

Carlbring, P., Maurin, T., Sjomark, J., Maurin, L., Westling, B. E., Ekselius, L., Cuijpers, P., & Andersson, G. (2011). All at once or one at a time? A randomized controlled trial comparing two ways to deliver bibliotherapy for panic disorder. *Cognitive Behaviour Therapy, 40*, 228-35.

Emmrich, A., Beesdo-Baum, K., Gloster, A. T., Knappe, S., Höfler, M., Arolt, V., Deckert, J., Gerlach, A. L., Hamm, A., Kircher, T., Lang, T., Richter, J., Ströhle, A., Zwanzger, P., & Wittchen, H.-U. (2012). Depression does not affect the treatment outcome of CBT for panic and agoraphobia: Results from a multicenter randomized trail. *Psychotherapy and Psychosomatics, 81*, 161-72.

Haug, T., Nordgreen, T., Öst, L.-G., Kvale, G., Tangen, T., Andersson, G., Carlbring, P., Heiervang, E. R., & Havik, O. E. (2015). Stepped care versus face-to–face cognitive behavior therapy for panic disorder and social anxiety disorder: Predictors and moderators of outcome. *Behaviour Research and Therapy, 71*, 76-89.

Kenardy, J., Robinson, S., & Dob, R. (2005). Cognitive behaviour therapy for panic disorder: Long-term follow up. *Cognitive Behaviour Therapy, 34*, 75-78.

Marchand, A., Roberge, P., Primiano, S., & Germain, V. (2009). A randomized, controlled clinical trial of standard, group and brief cognitive-behavioral therapy for panic disorder with agoraphobia: a two-year follow-up. *Journal of Anxiety Disorders, 23*, 1139-47.

Park, J. M., Mataix-Cols, D., Marks, I. M., Ngamthipwatthana, T., Marks, M., Araya, R., & Al-Kubaisy, T. (2001). Two-year follow-up after a randomised controlled trial of self- and clinician-accompanied exposure for phobia/panic disorders. *British Journal of Psychiatry, 178*, 543-8.

van Apeldoorn, F. J., Timmerman, M. E., Mersch, P. P., van Hout, W. J., Visser, S., van Dyck, R., & den Boer, J. A. (2010). A randomized trial of cognitive-behavioral therapy or selective serotonin reuptake inhibitor or both combined for panic disorder with or without agoraphobia: treatment results through 1-year follow-up. *Journal of Clinical Psychiatry, 71*, 574-86.

Van Apeldoorn, F. J., Van Hout, W. J., Timmerman, M. E., Mersch, P. P., & den Boer, J. A. (2013). Rate of improvement during and across three treatments for panic disorder with or without agoraphobia: cognitive behavioral therapy, selective serotonin reuptake inhibitor or both combined. *Journal of Affective Disorders, 150*, 313-9.

Wrong comparators (n=3)

Andersch, S., Hanson, L., & Hällström, T. (1997). Panic disorder: A five-year follow-up study in 52 patients. *The European Journal of Psychiatry, 11*, 145-55.

de Beurs, E., van Balkom, A. J., Van Dyck, R., & Lange, A. (1999). Long-term outcome of pharmacological and psychological treatment for panic disorder with agoraphobia: a 2-year naturalistic follow-up. *Acta Psychiatrica Scandinavica, 99*, 59-67.

Gloster, A. T., Hauke, C., Hofler, M., Einsle, F., Fydrich, T., Hamm, A., Sthrohle, A., & Wittchen, H. U. (2013). Long-term stability of cognitive behavioral therapy effects for panic disorder with agoraphobia: a two-year follow-up study. *Behaviour Research and Therapy, 51*, 830-9.

Wrong design (n=27)

Arntz, A., & de Jong, P. (1993). Anxiety, attention and pain. *Journal of Psychosomatic Research, 37*, 423-31.

Arolt, V., Zwanzger, P., Ströhle, A., Hamm, A., Gerlach, A., Kircher, T., & Deckert, J. (2009). The research network PANIC-NET: Improving the treatment of panic disorder - From a better understanding of fear circuit mechanisms to more effective psychological treatment and routine care. *PPmP Psychotherapie Psychosomatik Medizinische Psychologie, 59*, 124-31.

Baillie, A. J., & Rapee, R. M. (2004). Predicting who benefits from psychoeducation and self help for panic attacks. *Behaviour Research and Therapy, 42*, 513-27.

Bandelow, B., Seidler-Brandler, U., Becker, A., Wedekind, D., & Rüther, E. (2007). Meta-analysis of randomized controlled comparisons of psychopharmacological and psychological treatments for anxiety disorders. *World Journal of Biological Psychiatry, 8*, 175-87.

Barlow, D. H., Cohen, A. S., & Waddell, M. T. (1984). Panic and generalized anxiety disorders: nature and treatment. *Behavior Therapy, 15*, 431‐49.

Basoglu, M., Marks, I. M., Kilic, C., Brewin, C. R., & Swinson, R. P. (1994). Alprazolam and exposure for panic disorder with agoraphobia. Attribution of improvement to medication predicts subsequent relapse. *British Journal of Psychiatry, 164*, 652-9.

Bischoff, S., Wieder, G., Einsle, F., Petzold, M. B., Janßen, C., Mumm, J. L. M., Wittchen, H.-U., Fydrich, T., Plag, J., & Ströhle, A. (2018). Running for extinction? Aerobic exercise as an augmentation of exposure therapy in panic disorder with agoraphobia. *Journal of Psychiatric Research, 101*, 34-41.

Bouchard, S., Paquin, B., Payeur, R., Allard, M., Rivard, V., Fournier, T., Renaud, P., & Lapierre, J. (2004). Delivering cognitive-behavior therapy for panic disorder with agoraphobia in videoconference. *Telemedicine Journal and e-Health, 10*, 13‐25.

Busch, F. N., Milrod, B. L., & Sandberg, L. S. (2009). A study demonstrating efficacy of a psychoanalytic psychotherapy for panic disorder: Implications for psychoanalytic research, theory, and practice. *Journal of the American Psychoanalytic Association, 57*, 131-48.

Cammin-Nowak, S., Helbig-Lang, S., Lang, T., Gloster, A. T., Fehm, L., Gerlach, A. L., Strohle, A., Deckert, J., Kircher, T., Hamm, A. O., Alpers, G. W., Arolt, V., & Wittchen, H. U. (2013). Specificity of homework compliance effects on treatment outcome in CBT: evidence from a controlled trial on panic disorder and agoraphobia. *Journal of Clinical Psychology, 69*, 616-29.

Cuijpers, P., Cristea, I. A., Karyotaki, E., Reijnders, M., & Huibers, M. J. H. (2016). How effective are cognitive behavior therapies for major depression and anxiety disorders? A meta-analytic update of the evidence. *World psychiatry, 15*, 245‐58.

Cuijpers, P., Marks, I. M., van Straten, A., Cavanagh, K., Gega, L., & Andersson, G. (2009). Computer-aided psychotherapy for anxiety disorders: A meta-analytic review. *Cognitive Behaviour Therapy, 38*, 66-82.

Dear, B. F., Zou, J. B., Ali, S., Lorian, C. N., Johnston, L., Terides, M. D., Staples, L. G., Gandy, M., Fogliati, V. J., Klein, B., & Titov, N. (2015). Examining self-guided internet-delivered cognitive behavior therapy for older adults with symptoms of anxiety and depression: Two feasibility open trials. *Internet Interventions, 2*, 17-23.

Ebert, D. D., Gollwitzer, M., Riper, H., Cuijpers, P., Baumeister, H., & Berking, M. (2013). For whom does it work? moderators of outcome on the effect of a transdiagnostic internet-based maintenance treatment after inpatient psychotherapy: randomized controlled trial. *Journal of Medical Internet Research, 15*, e191.

Hecker, J. E., Fink, C. M., Vogeltanz, N. D., Thorpe, G. L., & Sigmon, S. T. (1998). Cognitive restructuring and interoceptive exposure in the treatment of panic disorder: a crossover study. *Behavioural and Cognitive Psychotherapy, 26*, 115‐31.

Hedman, E., Ljótsson, B., Rück, C., Bergström, J., Andersson, G., Kaldo, V., Jansson, L., Andersson, E., Andersson, E., Blom, K., El Alaoui, S., Falk, L., Ivarsson, J., Nasri, B., Rydh, S., & Lindefors, N. (2013). Effectiveness of Internet‐based cognitive behaviour therapy for panic disorder in routine psychiatric care. *Acta Psychiatrica Scandinavica, 128*, 457-67.

Kállai, J., Kosztolányi, P., Osváth, A., & Jacobs, W. J. (1999). Attention fixation training: Training people to form cognitive maps helps to control symptoms of panic disorder with agoraphobia. *Journal of Behavior Therapy and Experimental Psychiatry, 30*, 273-88.

Kim, B., Lee, S. H., Kim, Y. W., Choi, T. K., Yook, K., Suh, S. Y., Cho, S. J., & Yook, K. H. (2010). Effectiveness of a mindfulness-based cognitive therapy program as an adjunct to pharmacotherapy in patients with panic disorder. *Journal of Anxiety Disorders*.

Lebrecque, J., Marchand, A., Dugas, M. J., & Letarte, A. (2007). Efficacy of cognitive-behavioral therapy for comorbid panic disorder with agoraphobia and generalized anxiety disorder. *Behavior Modification, 31*, 616-37.

Meuret, A. E., Twohig, M. P., Rosenfield, D., Hayes, S. C., & Craske, M. G. (2012). Brief acceptance and commitment therapy and exposure for panic disorder: A pilot study. *Cognitive and Behavioral Practice, 19*, 606-18.

Miretzky, A., Horn, R., Koehler, K., & Moller, H. J. (1992). Combination of alprazolam, antidepressive drugs and cognitive behavior therapy in the treatment of panic disorder. *Clinical Neuropharmacology, 15*, 536.

Néron, S., Lacroix, D., & Chaput, Y. (1995). Group vs individual cognitive behaviour therapy in panic disorder: An open clinical trial with a six month follow-up. *Canadian Journal of Behavioural Science / Revue canadienne des sciences du comportement, 27*, 379-92.

Nuthall, A., & Townend, M. (2007). CBT-based early intervention to prevent panic disorder: a pilot study. *Behavioural and Cognitive Psychotherapy, 35*, 15‐30.

Otto, M. W., Pollack, M. H., Penava, S. J., & Zucker, B. G. (1999). Group cognitive-behavior therapy for patients failing to respond to pharmacology for panic disorder: A clinical case series. *Behaviour Research and Therapy, 37*, 763-70.

Peñate, W., Pitti, C. T., Bethencourt, J. M., de la Fuente, J., & Gracia, R. (2008). The effects of a treatment based on the use of virtual reality exposure and cognitive-behavioral therapy applied to patients with agoraphobia. *International Journal of Clinical and Health Psychology, 8*, 5-22.

Van Dyck, R., & Spinhoven, P. (1997). Does preference for type of treatment matter? A study of exposure in vivo with or without hypnosis in the treatment of panic disorder with agoraphobia. *Behavior Modification, 21*, 172-86.

Vincelli, F., Anolli, L., Bouchard, S., Wiederhold, B. K., Zurloni, V., & Riva, G. (2003). Experiential cognitive therapy in the treatment of panic disorders with agoraphobia: a controlled study. *Cyberpsychol Behav, 6*, 321-8.

Wrong intervention (n=13)

Bakker, A., Spinhoven, P., van Balkom, A. J., Vleugel, L., & van Dyck, R. (2000). Cognitive therapy by allocation versus cognitive therapy by preference in the treatment of panic disorder. *Psychotherapy and Psychosomatics, 69*, 240-3.

de Manincor, M., Bensoussan, A., Smith, C. A., Barr, K., Schweickle, M., Donoghoe, L. L., Bourchier, S., & Fahey, P. (2016). INDIVIDUALIZED YOGA FOR REDUCING DEPRESSION AND ANXIETY, AND IMPROVING WELL-BEING: A RANDOMIZED CONTROLLED TRIAL. *Depression and Anxiety, 33*, 816-28.

Forsyth, A., Deane, F. P., & Williams, P. (2015). A lifestyle intervention for primary care patients with depression and anxiety: A randomised controlled trial. *Psychiatry Research, 230*, 537-44.

Gaudlitz, K., Plag, J., Dimeo, F., & Strohle, A. (2015). Aerobic exercise training facilitates the effectiveness of cognitive behavioral therapy in panic disorder. *Depression and Anxiety, 32*, 221-8.

Lattari, E., Budde, H., Paes, F., Neto, G. A. M., Appolinario, J. C., Nardi, A. E., Murillo-Rodriguez, E., & Machado, S. (2018). Effects of aerobic exercise on anxiety symptoms and cortical activity in patients with Panic Disorder: A pilot study. *Clinical Practice and Epidemiology in Mental Health, 14*.

Muntingh, A., van der Feltz-Cornelis, C., van Marwijk, H., Spinhoven, P., Assendelft, W., de Waal, M., Ader, H., & van Balkom, A. (2014). Effectiveness of collaborative stepped care for anxiety disorders in primary care: a pragmatic cluster randomised controlled trial. *Psychotherapy and Psychosomatics, 83*, 37-44.

Muntingh, A., Van Der Feltz-Cornelis, C., Van Marwijk, H., Spinhoven, P., Assendelft, W., De Waal, M., Adèr, H., & Van Balkom, A. (2013). Effectiveness of collaborative stepped care for anxiety disorders in primary care: A pragmatic cluster randomised controlled trial. *Psychotherapy and Psychosomatics, 83*, 37-44.

Roy-Byrne, P. P., Katon, W., Cowley, D. S., & Russo, J. (2001). A randomized effectiveness trial of collaborative care for patients with panic disorder in primary care. *Archives of General Psychiatry, 58*, 869-76.

Schmidt, N. B., Wollaway-Bickel, K., Trakowski, J. H., Santiago, H. T., & Vasey, M. (2002). Antidepressant discontinuation in the context of cognitive behavioral treatment for panic disorder. *Behaviour Research and Therapy, 40*, 67-73.

Taylor, C. B., Kenigsberg, M. L., & Robinson, J. M. (1982). A controlled comparison of relaxation and diazepam in panic disorder. *Journal of Clinical Psychiatry, 43*, 423-5.

Tyrer, P. J., & Remington, M. (1979). Controlled comparison of day-hospital and outpatient treatment for neurotic disorders. *Lancet, 1*, 1014-6.

Vorkapic, C. F., & Rangé, B. (2014). Reducing the symptomatology of panic disorder: The effects of a yoga program alone and in combination with cognitive-behavioral therapy. *Frontiers in Psychiatry, 5*.

Wesner, A. C., Gomes, J. B., Detzel, T., Guimaraes, L. S., & Heldt, E. (2015). Booster Sessions after Cognitive-Behavioural Group Therapy for Panic Disorder: Impact on Resilience, Coping, and Quality of Life. *Behavioural and Cognitive Psychotherapy, 43*, 513-25.

Wrong outcome (n=20)

Basoglu, M., Marks, I. M., Swinson, R. P., Noshirvani, H., O'Sullivan, G., & Kuch, K. (1994). Pre-treatment predictors of treatment outcome in panic disorder and agoraphobia treated with alprazolam and exposure. *Journal of Affective Disorders, 30*, 123-32.

Black, D. W., Wesner, R., Bowers, W., Monahan, P., & Gabel, J. (1995). Acute treatment response in outpatients with panic disorder: high versus low depressive symptoms. *Annals of Clinical Psychiatry, 7*, 181-8.

Black, D. W., Wesner, R. B., Gabel, J., Bowers, W., & Monahan, P. (1994). Predictors of short-term treatment response in 66 patients with panic disorder. *Journal of Affective Disorders, 30*, 233-41.

Carter, F. A., Bell, C. J., & Colhoun, H. C. (2013). Suitability and acceptability of computerised cognitive behaviour therapy for anxiety disorders in secondary care. *Australian and New Zealand Journal of Psychiatry, 47*, 142-52.

Chavira, D. A., Stein, M. B., Golinelli, D., Sherbourne, C. D., Craske, M. G., Sullivan, G., Bystritsky, A., & Roy-Byrne, P. P. (2009). Predictors of clinical improvement in a randomized effectiveness trial for primary care patients with panic disorder. *Journal of Nervous and Mental Disease, 197*, 715-21.

Dusseldorp, E., Spinhoven, P., Bakker, A., van Dyck, R., & van Balkom, A. J. (2007). Which panic disorder patients benefit from which treatment: cognitive therapy or antidepressants? *Psychotherapy and Psychosomatics, 76*, 154-61.

El Alaoui, S., Hedman, E., Ljotsson, B., Bergstrom, J., Andersson, E., Ruck, C., Andersson, G., & Lindefors, N. (2013). Predictors and moderators of internet- and group-based cognitive behaviour therapy for panic disorder. *PloS One, 8*, e79024.

Goldin, P. R., Ziv, M., Jazaieri, H., Hahn, K., Heimberg, R., & Gross, J. J. (2013). Impact of cognitive behavioral therapy for social anxiety disorder on the neural dynamics of cognitive reappraisal of negative self-beliefs: randomized clinical trial. *JAMA Psychiatry, 70*, 1048-56.

Heatley, C., Ricketts, T., & Forrest, J. (2005). Training general practitioners in cognitive behavioural therapy for panic disorder: Randomized-controlled trial. *Journal of Mental Health, 14*, 73-82.

Hellström, L., Bech, P., Hjorthøj, C., Nordentoft, M., Lindschou, J., & Eplov, L. F. (2017). Effect on return to work or education of individual placement and support modified for people with mood and anxiety disorders: Results of a randomised clinical trial. *Occupational and Environmental Medicine, 74*, 717-25.

Katon, W. J., Roy-Byrne, P., Russo, J., & Cowley, D. (2002). Cost-effectiveness and cost offset of a collaborative care intervention for primary care patients with panic disorder. *Archives of General Psychiatry, 59*, 1098-104.

Klass, E. T., Milrod, B. L., Leon, A. C., Kay, S. J., Schwalberg, M., Li, C., & Markowitz, J. C. (2009). Does interpersonal loss preceding panic disorder onset moderate response to psychotherapy? An exploratory study. *Journal of Clinical Psychiatry, 70*, 406-11.

Liebscher, C., Wittmann, A., Gechter, J., Schlagenhauf, F., Lueken, U., Plag, J., Straube, B., Pfleiderer, B., Fehm, L., Gerlach, A. L., Kircher, T., Fydrich, T., Deckert, J., Wittchen, H.-U., Heinz, A., Arolt, V., & Ströhle, A. (2016). Facing the fear – clinical and neural effects of cognitive behavioural and pharmacotherapy in panic disorder with agoraphobia. *European Neuropsychopharmacology, 26*, 431-44.

McCrone, P., Marks, I. M., Mataix-Cols, D., Kenwright, M., & McDonough, M. (2009). Computer-aided self-exposure therapy for phobia/panic disorder: A pilot economic evaluation. *Cognitive Behaviour Therapy, 38*, 91-99.

Meulenbeek, P., Spinhoven, P., Smit, F., van Balkom, A., & Cuijpers, P. (2010). Cognitive mediation of panic reduction during an early intervention for panic. *Acta Psychiatrica Scandinavica, 122*, 20-9.

Michelson, L., Mavissakalian, M., Marchione, K., Ulrich, R. F., Marchione, N., & Testa, S. (1990). Psychophysiological outcome of cognitive, behavioral and psychophysiologically-based treatments of agoraphobia. *Behaviour Research and Therapy, 28*, 127-39.

Murphy, M. T., Michelson, L. K., Marchione, K., Marchione, N., & Testa, S. (1998). The role of self-directed in vivo exposure in combination with cognitive therapy, relaxation training, or therapist-assisted exposure in the treatment of panic disorder with agoraphobia. *Journal of Anxiety Disorders, 12*, 117-38.

Otto, M. W., McHugh, R. K., Simon, N. M., Farach, F. J., Worthington, J. J., & Pollack, M. H. (2010). Efficacy of CBT for benzodiazepine discontinuation in patients with panic disorder: Further evaluation. *Behaviour Research and Therapy, 48*, 720-7.

Roberge, P., Marchand, A., Reinharz, D., Cloutier, K., Mainguy, N., Miller, J. M., Begin, J., & Turcotte, J. (2005). Healthcare utilization following cognitive-behavioral treatment for panic disorder with agoraphobia. *Cognitive Behaviour Therapy, 34*, 79‐88.

White, K. S., Payne, L. A., Gorman, J. M., Shear, M. K., Woods, S. W., Saksa, J. R., & Barlow, D. H. (2013). Does maintenance CBT contribute to long-term treatment response of panic disorder with or without agoraphobia? A randomized controlled clinical trial. *Journal of Consulting and Clinical Psychology, 81*, 47-57.

Wrong population (n=48)

Acierno, R., Knapp, R., Tuerk, P., Gilmore, A. K., Lejuez, C., Ruggiero, K., Muzzy, W., Egede, L., Hernandez-Tejada, M. A., & Foa, E. B. (2017). A non-inferiority trial of Prolonged Exposure for posttraumatic stress disorder: In person versus home-based telehealth. *Behaviour Research and Therapy, 89*, 57-65.

Aderka, I. M., Gillihan, S. J., McLean, C. P., & Foa, E. B. (2013). The relationship between posttraumatic and depressive symptoms during prolonged exposure with and without cognitive restructuring for the treatment of posttraumatic stress disorder. *Journal of Consulting and Clinical Psychology, 81*, 375-82.

Ali, B. S., Rahbar, M. H., Naeem, S., Gul, A., Mubeen, S., & Iqbal, A. (2003). The effectiveness of counseling on anxiety and depression by minimally trained counselors: A randomized controlled trial. *American Journal of Psychotherapy, 57*, 324-26.

Andersen, T. E., Ellegaard, H., Schiøttz-Christensen, B., & Manniche, C. (2018). Somatic experiencing® for patients with low back pain and comorbid posttraumatic stress disorder - Protocol of a randomized controlled trial. *BMC Complementary and Alternative Medicine, 18*.

Asghari, E., Faramarzi, M., & Mohammmadi, A. K. (2016). The effect of cognitive behavioural therapy on anxiety, depression and stress in women with preeclampsia. *Journal of Clinical and Diagnostic Research, 10*, QC04-QC07.

Belaise, C., Fava, G. A., & Marks, I. M. (2005). Alternatives to debriefing and modifications to cognitive behavior therapy for posttraumatic stress disorder. *Psychotherapy and Psychosomatics, 74*, 212-17.

Björneklett, H. G., Lindemalm, C., Rosenblad, A., Ojutkangas, M. L., Letocha, H., Strang, P., & Bergkvist, L. (2012). A randomised controlled trial of support group intervention after breast cancer treatment: Results on anxiety and depression. *Acta Oncologica, 51*, 198-207.

Bormann, J. E., Thorp, S., Wetherell, J. L., & Golshan, S. (2008). A spiritually based group intervention for combat veterans with posttraumatic stress disorder: feasibility study. *Journal of holistic nursing : official journal of the American Holistic Nurses' Association, 26*, 109-16.

Brom, D., Stokar, Y., Lawi, C., Nuriel-Porat, V., Ziv, Y., Lerner, K., & Ross, G. (2017). Somatic Experiencing for Posttraumatic Stress Disorder: A Randomized Controlled Outcome Study. *Journal of Traumatic Stress, 30*, 304-12.

Bryant, R. A., Harvey, A. G., Dang, S. T., Sackville, T., & Basten, C. (1998). Treatment of acute stress disorder: A comparison of cognitive- behavioral therapy and supportive counseling. *Journal of Consulting and Clinical Psychology, 66*, 862-66.

Bryant, R. A., Moulds, M. L., Guthrie, R. M., & Nixon, R. D. V. (2005). The additive benefit of hypnosis and cognitive-behavioral therapy in treating acute stress disorder. *Journal of Consulting and Clinical Psychology, 73*, 334-40.

Carvalho, A. E. V., Linhares, M. B. M., Padovani, F. H. P., & Martinez, F. E. (2009). Anxiety and depression in mothers of preterm infants and psychological intervention during hospitalization in neonatal ICU. *The Spanish Journal of Psychology, 12*, 161-70.

Castillo, D. T., Chee, C. L., Nason, E., Keller, J., C'De Baca, J., Qualls, C., Fallon, S. K., Haaland, K. Y., Miller, M. W., & Keane, T. M. (2016). Group-delivered cognitive/exposure therapy for PTSD in women veterans: A randomized controlled trial. *Psychological trauma : theory, research, practice and policy, 8*, 404-12.

Chlan, L. (1998). Effectiveness of a music therapy intervention on relaxation and anxiety for patients receiving ventilatory assistance. *Heart and Lung: Journal of Acute and Critical Care, 27*, 169-76.

Conrad, P., & Adams, C. (2012). The effects of clinical aromatherapy for anxiety and depression in the high risk postpartum woman - a pilot study. *Complementary Therapies in Clinical Practice, 18*, 164-8.

Cook, J. M., Harb, G. C., Gehrman, P. R., Cary, M. S., Gamble, G. M., Forbes, D., & Ross, R. J. (2010). Imagery rehearsal for posttraumatic nightmares: a randomized controlled trial. *Journal of Traumatic Stress, 23*, 553-63.

Cusack, K., & Spates, C. R. (1999). The cognitive dismantling of Eye Movement Desensitization and Reprocessing (EMDR) treatment of posttraumatic stress disorder (PTSD). *Journal of Anxiety*

Ebert, D. D., Heber, E., Berking, M., Riper, H., Cuijpers, P., Funk, B., & Lehr, D. (2016). Self-guided internet-based and mobile-based stress management for employees: results of a randomised controlled trial. *Occupational and Environmental Medicine, 73*, 315‐23.

Emmelkamp, P. M. G., Benner, A., Kuipers, A., Feiertag, G. A., Koster, H. C., & Van Apeldoorn, F. J. (2006). Comparison of brief dynamic and cognitive-behavioural therapies in avoidant personality disorder. *British Journal of Psychiatry, 189*, 60-64.

Foa, E. B., Jameson, J. S., Turner, R. M., & Payne, L. L. (1980). Massed vs. spaced exposure sessions in the treatment of agoraphobia. *Behaviour Research and Therapy, 18*, 333-8.

Ford, J. D., Grasso, D. J., Greene, C. A., Slivinsky, M., & DeViva, J. C. (2018). Randomized clinical trial pilot study of prolonged exposure versus present centred affect regulation therapy for PTSD and anger problems with male military combat veterans. *Clinical Psychology & Psychotherapy, 25*, 641-49.

Franklin, C. L., Cuccurullo, L. A., Walton, J. L., Arseneau, J. R., & Petersen, N. J. (2017). Face to face but not in the same place: A pilot study of prolonged exposure therapy. *Journal of Trauma and Dissociation, 18*, 116-30.

González-Fernández, S., Fernández-Rodríguez, C., Paz-Caballero, M. D., & Pérez-Álvarez, M. (2018). Treating anxiety and depression of cancer survivors: Behavioral activation versus acceptance and commitment therapy. *Psicothema, 30*, 14-20.

Handley, A. K., Egan, S. J., Kane, R. T., & Rees, C. S. (2015). A randomised controlled trial of group cognitive behavioural therapy for perfectionism. *Behaviour Research and Therapy, 68*, 37-47.

Hinton, D. E., Chhean, D., Pich, V., Safren, S. A., Hofmann, S. G., & Pollack, M. H. (2005). A randomized controlled trial of cognitive-behavior therapy for Cambodian refugees with treatment-resistant PTSD and panic attacks: a cross-over design. *Journal of Traumatic Stress, 18*, 617-29.

Hynninen, M. J., Bjerke, N., Pallesen, S., Bakke, P. S., & Nordhus, I. H. (2010). A randomized controlled trial of cognitive behavioral therapy for anxiety and depression in COPD. *Respiratory Medicine, 104*, 986-94.

Keane, T. M., Fairbank, J. A., Caddell, J. M., & Zimering, R. T. (1989). Implosive (flooding) therapy reduces symptoms of PTSD in Vietnam combat veterans. *Behavior Therapy, 20*, 245-60.

Lilliecreutz, C., Josefsson, A., & Sydsjö, G. (2010). An open trial with cognitive behavioral therapy for blood- and injection phobia in pregnant women-a group intervention program. *Archives of women's mental health, 13*, 259‐65.

Maercker, A., Zöllner, T., Menning, H., Rabe, S., & Karl, A. (2006). Dresden PTSD treatment study: Randomized controlled trial of motor vehicle accident survivors. *BMC Psychiatry, 6*.

Man, A. K. Y., Yap, J. C. M., Kwan, S. Y., Suen, K. L., Yip, H. S., & Chen, P. P. (2003). The effect of intra-operative video on patient anxiety. *Anaesthesia, 58*, 64-68.

Merswolken, M., Siebenhuener, S., Orth-Gomér, K., Zimmermann-Viehoff, F., & Deter, H. C. (2011). Treating anxiety in patients with coronary heart disease: A randomized controlled trial. *Psychotherapy and Psychosomatics, 80*, 365-70.

Moorey, S., Greer, S., Watson, M., Baruch, J. D. R., Robertson, B. M., Mason, A., Rowden, L., Tunmore, R., Law, M., & Bliss, J. M. (1994). Adjuvant psychological therapy for patients with cancer: Outcome at one year. *Psycho-Oncology, 3*, 39-46.

Newby, J. (2016). Outcomes Of The iCanADAPT program: An internet-based CBT program for depression and anxiety in early-stage cancer and cancer survivors. *Asia-Pacific Journal of Clinical Oncology, 12*, 91.

Nobakht, A., Mohraz, M., Rahimzadeh, M., Tehranizadeh, M., Behboodi-Moghadam, Z., & Esmaelzadeh-Saeieh, S. (2018). The effect of cognitive behavioural therapy on depression, anxiety, and stress in women with HIV. *HIV and AIDS Review, 17*, 218-23.

Rees, B. L. (1995). Effect of relaxation with guided imagery on anxiety, depression, and self-esteem in primiparas. *Journal of holistic nursing : official journal of the American Holistic Nurses' Association, 13*, 255-67.

Reinhardt, K. M., Noggle Taylor, J. J., Johnston, J., Zameer, A., Cheema, S., & Khalsa, S. B. S. (2018). Kripalu yoga for military veterans with PTSD: A randomized trial. *Journal of Clinical Psychology, 74*, 93-108.

Rollman, B. L., Belnap, B. H., Abebe, K. Z., Spring, M. B., Rotondi, A. J., Rothenberger, S. D., & Karp, J. F. (2018). Effectiveness of online collaborative care for treating mood and anxiety disorders in primary care: A randomized clinical trial. *JAMA Psychiatry, 75*, 56-64.

Sautter, F. J., Glynn, S. M., Cretu, J. B., Senturk, D., & Vaught, A. S. (2015). Efficacy of structured approach therapy in reducing PTSD in returning veterans: A randomized clinical trial. *Psychological Services, 12*, 199-212.

Sheykhan, R., Mohammadkhani, S., & Hasanabadi, H. (2013). Self-focused attention in treatment of social anxiety: a controlled clinical trial. *Journal of mazandaran university of medical sciences, 23*, 119‐28.

Sizoo, B. B., & Kuiper, E. (2017). Cognitive behavioural therapy and mindfulness based stress reduction may be equally effective in reducing anxiety and depression in adults with autism spectrum disorders. *Research in Developmental Disabilities, 64*, 47-55.

Sloan, D. M., Unger, W., & Gayle Beck, J. (2016). Cognitive-behavioral group treatment for veterans diagnosed with PTSD: Design of a hybrid efficacy-effectiveness clinical trial. *Contemporary Clinical Trials, 47*, 123-30.

Thompson-Hollands, J., Marx, B. P., Lee, D. J., Resick, P. A., & Sloan, D. M. (2018). Long-term treatment gains of a brief exposure-based treatment for PTSD. *Depression and Anxiety, 35*, 985-91.

Trimmer, C., Tyo, R., Pikard, J., McKenna, C., & Naeem, F. (2018). Low-Intensity Cognitive Behavioural Therapy-Based Music Group (CBT-Music) for the Treatment of Symptoms of Anxiety and Depression: A Feasibility Study. *Behavioural and Cognitive Psychotherapy, 46*, 168-81.

van Beek, M. H., Oude Voshaar, R. C., Beek, A. M., van Zijderveld, G. A., Visser, S., Speckens, A. E., Batelaan, N., & van Balkom, A. J. (2013). A brief cognitive-behavioral intervention for treating depression and panic disorder in patients with noncardiac chest pain: a 24-week randomized controlled trial. *Depression and Anxiety, 30*, 670-8.

Van Dongen, M. A. J. C., Nelen, M. W. L. D., IntHout, J., Kremer, J. A. M., & Verhaak, C. M. (2015). E-therapy to reduce symptoms of anxiety and/or depression in women after unsuccessful artificial reproduction technology (ART): A randomised controlled trial. *Human Reproduction, 30*, i35-i36.

Watzke, B., Rüddel, H., Jürgensen, R., Koch, U., Kriston, L., Grothgar, B., & Schulz, H. (2010). Effectiveness of systematic treatment selection for psychodynamic and cognitive-behavioural therapy: Randomised controlled trial in routine mental healthcare. *British Journal of Psychiatry, 197*, 96-105.

Wells, A., & Sembi, S. (2004). Metacognitive therapy for PTSD: A preliminary investigation of a new brief treatment. *Journal of Behavior Therapy and Experimental Psychiatry, 35*, 307-18.

Zoellner, L. A., Feeny, N. C., Fitzgibbons, L. A., & Foa, E. B. (1999). Response of African American and Caucasian women to cognitive behavioral therapy for PTSD. *Behavior Therapy, 30*, 581-95.

**Appendix I - Characteristics of the CBT interventions**

| Unique ID | First author, year | Psychological Interventions | | | | | |
| --- | --- | --- | --- | --- | --- | --- | --- |
|  |  | Provision of therapy by a nationally licensed psychologist, psychiatrist, social worker, nurse / psychology Master or PhD students? | | Was treatment integrity verified? | Was a treatment manual used? | | |
|  |  |  |  |  | Name of the therapy | Manual reference / articles / books upon which the manual is based | |
| 1 | Addis, 2004^1^ | yes | 24 master’s-level therapists provided with a 2-day PCT training | yes | Panic control therapy (PCT) | yes | Craske, M. G., Meadows, E., & Barlow, D. H. (1994). Therapist’s guide for the mastery of your anxiety and panic II & agoraphobia supplement. Albany, NY: Graywind Publications. |
| 2 | Allen 2016^3^ | yes | A registered psychiatrist | NA | Panic course | yes | The treatment is described as manualized but the manual is not available |
| 3 | Bakker, 1999^9^ | yes | Experienced therapists who had received extensive training from experts in the field | yes | Cognitive therapy | yes | Clark DM. A cognitive approach to panic. Behav Res Ther 1986;24: 461–470 |
|  |  |  |  |  |  |  |  |
| 4 | Barlow, 1989^10^ | yes | Therapists were senior graduate students and psychologists who had been trained in the use of each of the three therapeutic procedures | yes | Exposure + cognitive restructuring | yes | Beck A. T. and Emery G. (1979) Cognitive Therapy of Anxiety and Phobic Disorders. Center for Cognitive Therapy, Philadelphia  -  Bernstein, D. A., & Borkovec, T. D. (1973). Progressive relaxation training. Champaign, IL: Research Press |
|  |  |  |  |  | Exposure + cognitive restructuring + relaxation |  |  |
| 5 | Barlow, 2000^11^ | yes | Doctoral level clinicians who underwent extensive training | no | CBT | yes | Barlow DH, Craske MG. Mastery of Your Anxiety and Panic, II. San Antonio, Tex: Graywind Publications Inc/The Psychological Corp; 1994 |
| 6 | Beck, 1992^12^ | yes | A trained cognitive therapist | no | focused cognitive therapy | yes | Beck AT, Emery G. Anxiety disorders and Phobias: A cognitive perspective. New York, basic books 1985 |
| 7 | Berger, 2017^14^ | NA |  | NA | unguided ICBT | yes | Berger, T., et al. (2017). "Effects of a transdiagnostic unguided Internet  intervention ('velibra') for anxiety disorders in primary care: results of a randomized  controlled trial." Psychol Med 47(1):67-80 |
| 8 | Bergstrom, 2010^15^ | yes | The psychologists involved in the treatment were regular staff psychologists not specially trained for participation in the trial. | no | CBT | yes | Telch MJ, Lucas JA, Schmidt NB, Hanna HH, LaNae Jaimez T, Lucas RA: Group cognitive-behavioral treatment of panic disorder. Behav Res Ther 1993, 31(3):279-287 |
|  |  |  |  |  | internet CBT | yes | Bergstrom J, Andersson G, Ljotsson B, et al.Internet versus group administered cognitive  Behaviour therapy for panic disorder in a psychiatric setting: a randomised trial. BMC  Psychiatry 2010;10:54 |
| 9 | Bohni, 2009^18^ | yes | Two therapists, with at least one being an experienced cognitive behavioural psychologist or psychiatrist | no | Massed CBT (M-CBT) | yes | Craske MG, Barlow DH. Panic disorder and agoraphobia. In: Barlow DH, ed. Clinical handbook of psychological disorders: a step-by-step treatment manual, 3rd edn. New York: Guilford Press, 2001:1–59  -  Clark DM. Panic disorder and social phobia. New York: Oxford University Press, 1997 |
|  |  |  |  |  | Standard CBT (S-CBT) |  |  |
| 10 | Botella, 1999^19^ | yes | “Experienced clinical psychologist” | no | Brief and Reduced Therapist Contact Treatment (BRTC) supported by self-help materials. | yes | Botella C, García-Palacios A. The possibility of reducing therapist contact and total length of therapy in the treatment of panic disorder. Behavioural and Cognitive Psychotherapy 1999;27(3):231-47 |
|  |  |  |  |  | Standard CBT | yes | CLARK, D. M., & SALKOVSKIS, P. M. (1989). Cognitive therapy for panic and hypocondriasis. Oxford: Pergamon  -  Clark, D. A., & Salkovskis, P. M. (1986). Cognitive treatment of panic: therapist’s manual. UK: Department of Psychiatry, University of Oxford |
| 11 | Botella, 2007^20^ | yes | The therapists were well trained in CBT programmes for PDA | no | In vivo exposure (IVE) AND Virtual reality exposure (VRE) | yes | Salkovskis, P.M., & Clark, D.M. (1991). Cognitive therapy for panic disorder. Journal of Cognitive Psychotherapy, 5, 215–226.  -  Barlow, D. H., & Craske, M. G. (1988). Mastery of your anxiety and panic. Albany, New York: Graywind Publications. |
| 12 | Brown, 1997^22^ | yes | Therapist of the Center for Cognitive Therapy of the University of Pennsylvania. | yes | Focused cognitive therapy | yes | Beck AT, Emery G. Anxiety disorders and Phobias: A cognitive perspective. New York, basic books 1985  -  Clark DM. A cognitive approach to panic. Behav Res Ther 1986; 24:461-70 |
|  |  |  |  |  | Standard cognitive therapy |  |  |
| 13 | Carlbring, 2006^24^ | Yes | two students in their final year of studying to become psychologists (M.Sc.) and one licensed psychologist (M.Sc.) who were all given regular supervision from a clinician experienced in cognitive behavior therapy | NA | Internet-based bibliotherapy self- help program | yes | Zuercher-White, E. (1998). An end to panic: Breakthrough techniques for  overcoming panic disorder (2nd ed.). Oakland, CA: New Harbinger Publications.  -  Barlow, D. H., & Craske, M. G. (1994). Mastery of your anxiety and panic H. San  Antonio, TX: The Psychological Corporation.  -  Clark, D. M. (1986). A cognitive approach to panic. Behaviour Research and  Therapy, 24,461-470. |
| 14 | Carlbring, 2005^26^ | yes | licensed psychologists, advanced graduate students under supervision | NA | CBT | yes | Clark, D. M. (1989). Anxiety states: panic and generalized anxiety. In K. Hawton, P. Salkovskis, J. Kirk, & D. M. Clark (Eds.), Cognitive behaviour therapy for psychiatric problems: a practical guide (pp. 52–96). Oxford: Oxford University Press |
|  |  |  |  |  | internet-based CBT | yes | Zuercher-White, E. (1998). An end to panic: Breakthrough techniques for overcoming  panic disorder (2nd ed.). Oakland, CA: New Harbinger Publications.  -  Barlow, D. H., & Craske, M. G. (1994). Mastery of your anxiety and panic H. San  Antonio, TX: The Psychological Corporation.  -  Clark, D. M. (1986). A cognitive approach to panic. Behaviour Research and  Therapy, 24,461-470. |
| 15 | Carlbring, 2001^27^ | unclear |  | NA | internet-based CBT | yes | Zuercher-White, E. (1998). An end to panic: Breakthrough techniques for overcoming  panic disorder (2nd ed.). Oakland, CA: New Harbinger Publications.  -  Barlow, D. H., & Craske, M. G. (1994). Mastery of your anxiety and panic H. San  Antonio, TX: The Psychological Corporation.  -  Clark, D. M. (1986). A cognitive approach to panic. Behaviour Research and  Therapy, 24,461-470. |
| 16 | Carter, 2003^28^ | yes | a licensed clinical psychologist with 15 years experience with CBT for anxiety disorders | NA | Panic control therapy (PCT) | yes | Barlow, D. H., &Craske, M.G. (1994). Mastery of your Anxiety and panic – II. San Antonio, TX: Harcourt Brace |
| 17 | Choi, 2005^29^ | unclear |  | no | Experiential cognitive therapy (ExCT) | yes | Vincelli, F., Choi, Y.H., Molinary, E., et al. (2000). Experiential cognitive therapy for  the treatment of panic disorder with agoraphobia: definition of a clinical protocol.  CyberPsychology & Behavior 3:375-85.  -  Wiederhold, B.K., & Wiederhold, M.D. (2004). Virtual Reality therapy for anxiety  disorders: advances in education and treatment. Washington, DC: American  Psychological Association.  -  Riva, G., Botella, C., Légeron, P., et al. (2004). Cybertherapy: Internet and virtual  reality as assessment and rehabilitation tools for clinical psychology and  neuroscience. Amsterdam: IOS Press. Available:  <www.cybertherapy.info/pages/book3.htm>. |
|  |  |  |  |  | Panic control therapy (PCT) | yes | Craske, M. G., Meadows, E., & Barlow, D. H. (1994). Therapist’s guide for the mastery of your anxiety and panic II & agoraphobia supplement. Albany, NY: Graywind Publications |
| 18 | Christoforou, 2017^30^ | NA |  | NA | "Agoraphobia free" app | yes | The treatment is described as manualized but the manual is not available |
|  |  |  |  |  | "Stress free" app | yes | The treatment is described as manualized but the manual is not available |
| 19 | Ciuca, 2018^31^ | yes | Three licensed psychotherapists with formal training in cognitive behavioral therapy and a minimum of 3 years of clinical experience | no | Skype guided PAXPD | yes | Miclea, M., Miclea, Ciuca, A. M., & Budău, O. (2010). Computer-mediated  psychotherapy. Present and prospects. A developer perspective. Cognition, Brain,  Behavior: An Interdisciplinary Journal, 14, 185–208 |
|  |  |  |  |  | Skype unguided PAXPD |  |  |
| 20 | Clark, 1999^33^ | yes | Four clinical psychologists with experience in the use of cognitive and behavioral treatments for anxiety | no | Brief cognitive therapy | yes | Clark (1989). Cognitive Behaviour Therapy for Psychiatric Problems: A Practical Guide. Oxford University Press  -  Salkovskis & Clark, D. M. (1991) Cognitive therapy for panic disorder. Journal of  Cognitive Psychotherapy, 5,215-226 |
|  |  |  |  |  | Full cognitive therapy |  |  |
| 21 | Craske, 2003^35^ | yes | Graduate students and post-doctoral fellows trained by the senior author | yes | PCT + IV | yes | The treatment is described as manualized but the manual is not available |
|  |  |  |  |  | PCT | yes | The treatment is described as manualized but the manual is not available |
| 22 | Craske, 2007^36^ | yes | Senior clinical psychology doctoral students and postdoctoral fellows trained by the principal author | yes | PDA | yes | Barlow, D. H., & Craske, M. G. (1988). Mastery of your anxiety and panic. Albany, New York: Graywind Publications |
|  |  |  |  |  | PDA+C |  |  |
| 23 | Craske, 2005^37^ | yes | Senior clinical psychology doctoral students and postdoctoral fellows trained by the principal author | yes | CBT | yes | Uhde, T. W. (1994). The anxiety disorders: Phenomenology and treatment of core symptoms and associated sleep disturbance. In M. Kryger, T. Roth, & W. Dement (Eds.), Principles and practice of sleep medicine. Philadelphia: W. B. Saunders |
| 24 | Craske, 2011^39^ | yes | 6 social workers, 5 registered nurses, 2 master’slevel psychologists, and1 doctoral-level psychologist received 3 full days of didactic presentations about the CBT program | yes | Coordinated Anxiety Learning and Management (CALM) | yes | The treatment is described as manualized but the manual is not available |
| 25 | Craske, 1997^40^ | yes | Two therapist experienced in CBT | no | cognitive therapy plus interoceptive exposure plus in vivo exposure (CIE) | yes | Barlow, Craske, Cerny, Klosko (1989). Behavioural treatment of panic disorder. Behaviour therapy, 20 261-82 |
|  |  |  |  |  | cognitive therapy plus breathing retraining plus in vivo exposure (CBE) |  |  |
| 26 | de Beurs, 1995^43^ | yes | Experienced behaviour therapist | yes | Panic management (+ exposure) | yes | The treatment is described as manualized but the manual is not available |
| 27 | de Ruiter, 1989^44^ | yes | Four junior clinical psychologists with some prior experience with psychotherapy. They were specially trained in the treatments used | no | Breathing Retraining / cognitive Restrlicturing (BRCR) | yes | Clark, D. A., & Salkovskis, P. M. (1986). Cognitive treatment of panic: therapist’s manual. UK: Department of Psychiatry, University of Oxford |
| 28 | Erickson, 2007^47^ | yes | a senior doctoral-level psychologist, assisted by a senior graduate student in clinical psychology | no | CBT | yes | The treatment is described as manualized but the manual is not available |
| 29 | Fogliati, 2016^50^ | yes | Three accredited and nationally registered psychologists and one CBT-trained counsellor provided treatment | NA | disorder-specific CBT (DS-CBT) | yes | The treatment is described as manualized but the manual is not available |
|  |  |  |  |  | Transdiagnostic CBT (TD-CBT) | yes | The treatment is described as manualized but the manual is not available |
| 30 | Gensichen, 2019^51^ | yes | GP practice teams (GP and practice nurse) instructed on how to administer CBT | yes | practice team–supported exposure training | yes | Margraf J, Barlow DH, Clark DM, Telch MJ: Psychological treatment of panic: work in progress on outcome, active ingredients, and follow-up. Behav Res Ther 1993; 31: 1–8.  -  Wagner EH, Austin BT, Davis C, Hindmarsh M, Schaefer J, Bonomi A: Improving chronic illness care: translating evidence into action. Health Aff (Millwood) 2001; 20: 64–78 |
| 31 | Gloster, 2011^54^ | yes | advanced-level clinical psychology graduate students and postdocs experienced in CBT of anxiety disorders took part in a 3-day training workshop. | yes | CBT (T+ variant) | yes | The treatment is described as manualized but the manual is not available |
|  |  |  |  |  | CBT (T- variant) |  |  |
| 32 | Gould, 1993^56^ | yes | Quote”four therapists” | A random sample of 8 sessions was observed to ensure treatment integrity. | Bibliotherapy (BT) | yes | Clum, G. A. (1990). Coping with panic. Pacific Grove, CA: Brooks/Cole Publishing.  -  Ost, L. G. (1988). Applied relaxation vs. progressive relaxation in the treatment of panic disorder. Behaviour Research and Therapy, 26, 13-22  -  Craske, M. G., & Barlow, D. H. (1987, November). Behavioral treatment of panic: A controlled study. Paper presented at the Association for Advancement of Behavior Therapy, Boston |
|  |  |  |  |  | Individual therapy using Guided Imaginal Coping  (ITGIC) |  |  |
| 33 | Hazen 1996^58^ | yes | Quote: ”Professional therapists” | no | self-help manual | yes | Clum, G. A. (1990). Coping with panic. Pacific Grove, CA: Brooks/Cole Publishing |
|  |  |  |  |  | self-help group |  |  |
|  |  |  |  |  | professionally led group |  |  |
| 34 | Hecker, 1996^59^ | yes | Therapists were a licensed psychologist and three graduate students in clinical psychology. The therapists met weekly for group supervision a | no | self-directed CBT | yes | Barlow, D. H., & Craske, M. G. (1988). Mastery of your anxiety and panic. Albany, New York: Graywind Publications |
|  |  |  |  |  | therapist-directed CBT |  |  |
| 35 | Hendriks, 2010^60^ | yes | The therapists were all psychologists trained at the master of science level with extensive experience in cognitive-behavioural techniques for adults with PD(A) | no | CBT | yes | Craske, M. G., Meadows, E., & Barlow, D. H. (1994). Therapist’s guide for the mastery of your anxiety and panic II & agoraphobia supplement. Albany, NY: Graywind Publications |
| 36 | Kenardy, 2003^66^ | yes | licensed psychologists with extensive experience of CBT | no | CBT-12 | yes | Barlow, Craske, Cerny, Klosko (1989). Behavioural treatment of panic disorder. Behaviour therapy, 20 261-82  -  Clark DM, A cognitive approach to panic, J. Behav. Ther. Exp. Psychiatry, 1986; 24: 461-70 |
|  |  |  |  |  | CBT-6 |  |  |
|  |  |  |  |  | Computer-augmented CBT6 (CBT-6-CA) |  |  |
| 37 | Kiropoulos, 2008^68^ | yes | registered psychologists and one probationary psychologist all trained in CBT for PD. | yes | CBT | yes | Barlow, D. H., & Craske, M. G. (1988). Mastery of your anxiety and panic. Albany, New York: Graywind Publications |
|  |  |  |  |  | Panic Online (PO) | yes | Kiropoulos LA, Klein B, Austin DW, et al. Is  internet-based CBT for panic disorder and  agoraphobia as effective as face-to face CBT? Journal of anxiety disorders 2008;22(8):127384 |
| 38 | Klein, 2009^69^ | yes | Seven registered psychologists and one probationary registered psychologist trained in the administration of the ADIS-IV conducted the online therapy work | NA | Panic Online x1 contact/week | yes | Klein B, Austin D, Pier C, et al. Internet-based  treatment for Panic disorder: does frequency  of therapist contact make a difference?  Cognitive behaviour therapy 2009;38(2):100-13 |
|  |  |  |  |  | Panic Online x3 contacts/week |  |  |
| 39 | Klein, 2001^70^ | NA |  | NA | internet-based program | yes | The therapy is manualized but the manual is not provided |
| 40 | Klein, 2006^71^ | yes | The telephone therapists were two Doctor of Psychology (Clinical) students (one male and one female) both trained in CBT for PD | no | Panic Online | yes | Barlow, D. H., & Craske, M. G. Mastery of your anxiety and panic. Albany, New York: Graywind Publications |
|  |  |  |  |  | manualized CBT workbook |  |  |
| 41 | Klosko, 1990^72^ | yes | PhD psychologists or advanced doctoral students who had been trained in the application of the treatment. | yes | Panic control treatment (PCT) | yes | Barlow, D. H., & Cerny, J. A. (1988). Psychological treatment of panic. New York: Guilford Press |
| 42 | Koszycki, 2011^74^ | NA |  | NA | self-administered CBT (SCBT) | yes | The therapy is manualized but the manual is not provided |
| 43 | Lidren, 1994^75^ | yes | An advanced clinical psychology graduate student led each of the two groups | yes | Bibliotherapy | yes | Clum, G. A. (1990). Coping with panic. Pacific Grove, CA: Brooks/Cole Publishing |
|  |  |  |  |  | Group therapy |  |  |
| 44 | Loerch, 1999^76^ | yes | Quote: “two clinical psychologists” | no | CBT | yes | The therapy is manualized but the manual is not provided |
| 45 | Marchand, 2008^78^ | yes | Licensed psychologists, specifically trained in CBT | yes | graded exposure (GE) | yes | Marks, I. M. (1987). Fears, phobias, and rituals: Panic, anxiety, and their disorders. New York: Oxford University Press |
|  |  |  |  |  | Cognitive therapy (CT) | yes | Beck, A. T. (1988). Cognitive approaches to panic disorder: Theory and therapy. In S. Rachman & J. D. Maser (Eds), Panic: Psychological perspectives Hillsdale, NJ: Erlbaum  -  Clark, D. M. (1988). A cognitive model of panic attacks. In S. Rachman & J. D. Maser (Eds), Panic: Psychological perspectives (pp. 71–89). Hillsdale, NJ: Erlbaum.  -  Meichenbaum, D. (1977). Cognitive-behavior modification: An integrative approach. New York: Plenum Press |
|  |  |  |  |  | supportive therapy | yes | Friedman, W. H. (1989). Practical group therapy. San Francisco: Jossey-Bass.  -  Yalom, I. D. (1970). The theory and practice of group psychotherapy. New York: Basics Books |
| 46 | Marchand, 2007^79^ | yes | Therapist were experienced in the CB treatment of PDA | yes | Standard CBT | yes | Craske MG, Barlow DH. Panic disorder and agoraphobia. In: Barlow DH, ed. Clinical handbook of psychological disorders: a step-by-step treatment manual, 3rd edn. New York: Guilford Press, 2001:1–59  -  Barlow, D. H., & Craske, M. G. (1988). Mastery of your anxiety and panic. Albany, New York: Graywind Publications |
|  |  |  |  |  | Brief cognitive behaviour therapy alone (BCBT-A) | yes | The therapy is manualized but the manual reference is not provided |
|  |  |  |  |  | Brief cognitive behaviour therapy with partner (BCBT-P) | yes | The therapy is manualized but the manual reference is not provided |
| 47 | Meulenbeek, 2010^84^ | Likely yes |  | yes | ‘Don’t Panic’ course | yes | Meulenbeek P, Herzmanatus J, Smit F, Willemse G, Van der Zanden R. Draaiboek: Geen Paniek, Leren Omgaan met Paniekklachten [Manual and Workbook: Don’t Panic, Learn to Cope with Panic Complaints]. Trimbos Institute/GGNet, 2005. |
| 48 | Newman, 1997^92^ | yes | Therapist with extensive experience in the use of CBT techniques. | no | Standard CBT  (CBT12) | yes | Craske, M. G., Rapee, R., & Barlow, D. H. (1987). Information and cognitive plus breathing retraining and exposure protocol. Unpublished manual |
|  |  |  |  |  | Computer assisted CBT (CBT4-CA) |  |  |
| 49 | Nordin, 2010^94^ | NA |  | NA | bibliotherapy | yes | Carlbring, P., & Hanell, Å. (2007). Ingen panik: Fri från panikoch ångestattacker i 10 steg med kognitiv beteendeterapi. [No panic: Free from panic and anxiety]. Stockholm: Natur och Kultur |
| 50 | Oh, 2020^95^ | NA |  | NA | Chat bot APP | yes | The therapy is manualized but the manual reference is not provided |
|  |  |  |  |  | bibliotherapy | yes | J.-Y. Choi, Goodbye Panic Disorder, Sigmabooks, Seoul, 2009. |
| 51 | Oromendia, 2016^96^ | NA |  | NA | “Free from Anxiety” web program | yes | The therapy is manualized but the manual reference is not provided |
| 52 | Ost, 2004^98^ | yes | Three licensed psychotherapists with long experience of CBT | yes | CBT | yes | Beck AT, Emery G. Anxiety disorders and Phobias: A cognitive perspective. New York, basic books 1985  -  Clark DM. A cognitive approach to panic. Behav Res Ther 1986; 24:461-70  -  Clark (1989). Anxiety states: panic and generalized anxiety. In Cognitive Behaviour Therapy for Psychiatric Problems: A Practical Guide (eds K. Hawton, P. Salkovskis, J. Kirk, et al), pp. 52-96.Oxford: Oxford University Press |
| 53 | Pelissolo, 2012^102^ | yes | post-graduate psychologists or psychiatrists, had practiced CBT for at least five years | no | CBT | yes | Landon, T. M., & Barlow, D. H. (2004). Cognitive-behavioral treatment for panic disorder: current status. Journal of Psychiatric Practice, 10, 211-226 |
| 54 | Petterson, 1996^103^ | unclear |  | no | CBT | unclear | No info |
| 55 | Pitti, 2015^104^ | Unclear |  | no | CBT | unclear | No info |
| 56 | Reinecke, 2013^107^ | unclear |  | no | exposure-based CBT | yes | Clark DM (1989): Anxiety states—panic and generalized anxiety. In: Hawton K, Salkovskis PM, Kirk J, Clark DM, editors. Cognitive Therapy for Psychiatric Problems: A Practical Guide. Oxford: Oxford University Press, 52–96  -  Salkovskis PM, Clark DM, Hackmann A, Wells A, Gelder MG (1999): An experimental investigation of the role of safety-seeking behaviours in the maintenance of panic disorder with agoraphobia. Behav Res Ther 37:559–574. |
| 57 | Richards, 2006^108^ | unclear |  | NA | Internet-based CBT (PO1) | yes | The therapy is manualized but the manual reference is not provided |
|  |  |  |  |  | Internet-based CBT plus stress management (P02) | yes | The therapy is manualized but the manual reference is not provided |
| 58 | Roberge, 2008^109^ | yes | Psychologists, psychiatrists, and advanced doctoral-level psychology students and psychiatry residents conducted evaluations and treatments | yes | standard CBT | yes | Craske MG, Barlow DH. Panic disorder and agoraphobia. In: Barlow DH, ed. Clinical handbook of psychological disorders: a step-by-step treatment manual, 3rd edn. New York: Guilford Press, 2001:1–59 |
|  |  |  |  |  | group CBT |  |  |
|  |  |  |  |  | brief CBT |  |  |
| 59 | Roy-Byrne, 2005^110^ | yes | This intervention used therapists who were minimally or not at all trained in CBT | No | CBT modified for primary care setting. | yes | The therapy is manualized but the manual reference is not provided |
| 60 | Roy-Byrne, 2010^111^ | yes | with newly TheACS  personnel received 6 half days of didactics. CBT training also included role-playing and required successful completion of 2 training patients over several months. | no | Coordinated Anxiety Learning and Management (CALM) | yes | Sullivan G, Craske MG, Sherbourne C, et al. Design of the Coordinated Anxiety Learning and Management (CALM) study: innovations in collaborative care for anxiety disorders. GenHosp Psychiatry. 2007; 29(5):379-387 |
| 61 | Ruwaard, 2010^112^ | yes | All therapists had followed advanced courses in CBT, and received additional training in administering web-CBT | yes | WEB CBT | yes | The therapy is manualized but the manual reference is not provided |
| 62 | Schmidt, 1997a^115^ | yes | a licensed psychologist with 10years ofexperience with cognitive-behavioral treatment of anxiety disorders | yes | CBT | yes | The therapy is manualized but the manual reference is not provided |
| 63 | Schmidt, 1997b^116^ | yes | a licensed psychologist with 10years ofexperience with cognitive-behavioral treatment of anxiety disorders | yes | CBT | yes | The therapy is manualized but the manual reference is not provided |
|  |  |  |  |  | respiratory training | yes | The therapy is manualized but the manual reference is not provided |
| 64 | Sharp, 1997^117^ | yes | Quote: “a psychologist therapist” | no | CBT | yes | Barlow DH. Anxiety and its disorders: The nature and treatment of anxiety and panic. New York: Guilford Press, 1988  -  Zinbarg RE, Barlow DH, Brown TA, et al. Cognitive behavioural approaches to the nature and treatment of anxiety disorders. Ann Rev Psychology 1992; 43: 235-267 |
| 65 | Sharp, 2000^118^ | yes | Quote: “A psychologist therapist” | no | CBT (standard) | yes | Barlow, D. H., & Cerny, J. A. (1988). Psychological treatment of panic. New York: Guilford Press |
|  |  |  |  |  | CBT (minimum contact) |  |  |
|  |  |  |  |  | bibliotherapy |  |  |
| 66 | Sharp, 2004^119^ | yes | Quote: “A single therapist delivered all treatments.” | no | Group CBT | yes | Power, K.G., Sharp, D.M., Swanson, V., & Simpson, R.J. (2000). Therapist contact in cognitive behaviour therapy for panic disorder and agoraphobia in primary care. Clinical Psychology and Psychotherapy, 7, 37–46 |
|  |  |  |  |  | Individual CBT |  |  |
| 67 | Shear, 2001^120^ | yes | Cognitive behavior therapists were master’s- or doctoral-level clinicians who completed required training | no | CBT | yes | Barlow, D. H., & Craske, M. G. (1988). Mastery of your anxiety and panic. Albany, New York: Graywind Publications |
| 68 | Silfvernagel, 2012^122^ | yes | three clinical psychology MSc students who had completed their clinical training and who were supervised by experienced clinical psychologists | no | Internet CBT | yes | The therapy is manualized but the manual reference is not provided |
| 69 | Telch, 1993^125^ | yes | All sessions were conducted by one primary therapist and a graduate student assistant. | yes | CBT | yes | Craske, M. G., Meadows, E., & Barlow, D. H. (1994). Therapist’s guide for the mastery of your anxiety and panic II & agoraphobia supplement. Albany, NY: Graywind Publications |
| 70 | Titov, 2010^127^ | yes | Two clinical psychologists | no | iCBT - the Anxiety program | yes | Andrews, G., Creamer, M., Crino, R., Hunt, C., Lampe, L., & Page, A. (2003). The treatment of anxiety disorders: Clinician guides and patient manuals (2nd ed.). UK: Cambridge University Press |
| 71 | Tyrer, 1988^128^ | yes | a team of community nurses who had received training and supervision | no | CBT | yes | Beck AT Cognitive therapy and the emotional disorders. New York: International Universities Press, 1976 |
|  |  |  |  |  | Self-help treatment package | yes | The therapy is manualized but the manual reference is not provided |
| 72 | van Ballegooijen, 2013^130^ | yes | Trained, Master’s-level clinical psychology students | no | Don’t Panic Online | yes | van Ballegooijen W et al. The effects of an Internet based self-help course for reducing panic symptoms--Don't Panic Online: study protocol for a randomised controlled trial. Trials 2011;12:75 |
| 73 | Williams, 1996^133^ | yes | a doctoral-level psychologist and a masters-level counselor, both highly experienced and trained | yes | Cognitive therapy | yes | Beck, A. T. & Emery, G. (1985). Anxiety Disorders and Phobias: A Cognitive Perspective. New York: Basic Books |
|  |  |  |  |  | performance treatment | yes | Williams, S. L. & Zane, G. (1989). Guided mastery and stimulus exposure treatments for severe performance anxiety in agoraphobics. Behaviour Research and Therapy, 27, 237-245.  -  Williams, S. L., Dooseman, G. & Kleifield, E. (1984). Comparative effectiveness of guided mastery and exposure treatments for intractable phobias. Journal of Consulting and Clinical Psychology, 52, 505-518.  -  Williams, S. L. (1990). Guided mastery treatment of agoraphobia: Beyond stimulus exposure. Progress in Behavior Modification, 26, 89 121.  -  Zane, G. & Williams, S. L. (1993). Performance-related anxiety in agoraphobia: treatment procedures and cognitive mechanisms of change. Behavior Therapy, 24, 625-643 |
| 74 | Wims, 2010^134^ | yes | One psychiatry registrar | no | Panic program | yes | The therapy is manualized but the manual reference is not provided |

**Supplement J - Risk of bias of the included studies**

| **Author** | Domain 1 | Domain 2 a | Domain 2 b | Domain 3 | Domain 4 | Domain 5 | **OVERALL RISK OF BIAS** |
| --- | --- | --- | --- | --- | --- | --- | --- |
|  | Risk of bias arising from the randomization process | Risk of bias due to deviations from the intended interventions (effect of assignment to intervention) | Risk of bias due to deviations from the intended interventions (effect of adhering to intervention) | Missing outcome data | Risk of bias in measurement of the outcome | Risk of bias in selection of the reported result |  |
|  | **Risk-of-bias judgement** | **Risk-of-bias judgement** | **Risk-of-bias judgement** | **Risk-of-bias judgement** | **Risk-of-bias judgement** | **Risk-of-bias judgement** |  |
| Addis, 2004 | Some Concerns | Low Risk | Low Risk | Low Risk | Low Risk | Some Concerns | LOW RISK |
| Allen 2016 | Low Risk | Low Risk | Some Concerns | Low Risk | Some Concerns | Low Risk | LOW RISK |
| Bakker, 1999 | Some Concerns | Low Risk | Low Risk | Low Risk | High Risk | Some Concerns | SOME CONCERNS |
| Barlow, 1989 | Some Concerns | High Risk | High Risk | Low Risk | Some Concerns | Some Concerns | HIGH RISK |
| Barlow, 2000 | Some Concerns | Low Risk | Low Risk | Some Concerns | Low Risk | Some Concerns | SOME CONCERNS |
| Beck, 1992 | Some Concerns | High Risk | Some Concerns | Low Risk | Low Risk | Some Concerns | HIGH RISK |
| Berger, 2017 | Low Risk | Low Risk | Low Risk | Low Risk | Some Concerns | Low Risk | LOW RISK |
| Bergstrom, 2010 | Some Concerns | Low Risk | Some Concerns | Low Risk | Low Risk | Some Concerns | SOME CONCERNS |
| Bohni, 2009 | Some Concerns | Low Risk | Low Risk | Low Risk | Some Concerns | Some Concerns | SOME CONCERNS |
| Botella, 1999 | Some Concerns | Some Concerns | Low Risk | Low Risk | High Risk | Some Concerns | HIGH RISK |
| Botella, 2007 | Low Risk | Some Concerns | Some Concerns | Low Risk | Low Risk | Some Concerns | SOME CONCERNS |
| Brown, 1997 | Some Concerns | Low Risk | Low Risk | Some Concerns | Low Risk | Some Concerns | SOME CONCERNS |
| Carlbring, 2006 | Some Concerns | Low Risk | Some Concerns | Low Risk | Low Risk | Some Concerns | SOME CONCERNS |
| Carlbring, 2005 | Some Concerns | Low Risk | Low Risk | Low Risk | Low Risk | Some Concerns | LOW RISK |
| Carlbring, 2001 | Some Concerns | Low Risk | Some Concerns | Low Risk | Some Concerns | Some Concerns | HIGH RISK |
| Carter, 2003 | Some Concerns | High Risk | Low Risk | High Risk | Some Concerns | Some Concerns | HIGH RISK |
| Choi, 2005 | Some Concerns | Some Concerns | Low Risk | Low Risk | Low Risk | Some Concerns | SOME CONCERNS |
| Christoforou, 2017 | Low Risk | Low Risk | Some Concerns | Some Concerns | Low Risk | Some Concerns | SOME CONCERNS |
| Ciuca, 2018 | Low Risk | Low Risk | Some Concerns | Some Concerns | Some Concerns | Low Risk | SOME CONCERNS |
| Clark, 1999 | Some Concerns | Some Concerns | Low Risk | Low Risk | Some Concerns | Some Concerns | HIGH RISK |
| Craske, 2003 | Some Concerns | Some Concerns | Low Risk | High Risk | Some Concerns | Some Concerns | HIGH RISK |
| Craske, 2007 | Some Concerns | Some Concerns | Low Risk | Low Risk | Low Risk | Some Concerns | SOME CONCERNS |
| Craske, 2005 | High Risk | Some Concerns | Low Risk | Low Risk | Some Concerns | Some Concerns | HIGH RISK |
| Craske, 2011 | Low Risk | Some Concerns | High Risk | Low Risk | Some Concerns | Some Concerns | HIGH RISK |
| Craske, 1997 | Some Concerns | High Risk | High Risk | Some Concerns | Low Risk | Some Concerns | HIGH RISK |
| de Beurs, 1995 | Some Concerns | High Risk | High Risk | Some Concerns | Low Risk | Some Concerns | HIGH RISK |
| de Ruiter, 1989 | High Risk | High Risk | Low Risk | Low Risk | Low Risk | Some Concerns | HIGH RISK |
| Erickson, 2007 | Some Concerns | High Risk | Low Risk | Some Concerns | Some Concerns | Some Concerns | HIGH RISK |
| Fogliati 2016 | Some Concerns | Low Risk | Low Risk | Low Risk | Low Risk | Low Risk | LOW RISK |
| Gensichen, 2020 | Some Concerns | Low Risk | Low Risk | Low Risk | Some Concerns | Low Risk | LOW RISK |
| Gloster, 2011 | Some Concerns | Low Risk | Low Risk | Low Risk | Low Risk | Some Concerns | LOW RISK |
| Gould, 1993 | Some Concerns | High Risk | High Risk | Low Risk | Some Concerns | Some Concerns | HIGH RISK |
| Hazen, 1996 | Some Concerns | High Risk | High Risk | Low Risk | Some Concerns | Some Concerns | HIGH RISK |
| Hecker, 1996 | Some Concerns | High Risk | Low Risk | Some Concerns | Low Risk | Some Concerns | HIGH RISK |
| Hendriks, 2010 | Low Risk | Low Risk | Some Concerns | Low Risk | Some Concerns | Some Concerns | SOME CONCERNS |
| Kenardy, 2003 | High Risk | High Risk | Low Risk | Some Concerns | Some Concerns | Some Concerns | HIGH RISK |
| Kiropoulos, 2008 | High Risk | Low Risk | Some Concerns | Low Risk | Low Risk | Some Concerns | SOME CONCERNS |
| Klein, 2009 | Some Concerns | Low Risk | Low Risk | Some Concerns | Low Risk | Some Concerns | SOME CONCERNS |
| Klein, 2001 | Some Concerns | High Risk | High Risk | Low Risk | Some Concerns | Some Concerns | HIGH RISK |
| Klein, 2006 | Some Concerns | Low Risk | Some Concerns | Low Risk | High Risk | Some Concerns | HIGH RISK |
| Klosko, 1990 | Some Concerns | High Risk | High Risk | High Risk | Low Risk | Some Concerns | HIGH RISK |
| Koszycki, 2011 | Low Risk | Low Risk | Some Concerns | Some Concerns | Low Risk | Some Concerns | SOME CONCERNS |
| Lidren, 1994 | Some Concerns | High Risk | High Risk | Low Risk | Some Concerns | Some Concerns | HIGH RISK |
| Loerch, 1999 | Some Concerns | Low Risk | Low Risk | High Risk | Some Concerns | Some Concerns | HIGH RISK |
| Marchand, 2008 | Some Concerns | High Risk | Low Risk | Low Risk | Some Concerns | Some Concerns | HIGH RISK |
| Marchand, 2007 | Some Concerns | Some Concerns | Low Risk | Low Risk | Some Concerns | Some Concerns | HIGH RISK |
| Meulenbeek, 2010 | Some Concerns | Low Risk | Low Risk | Low Risk | Some Concerns | Low Risk | LOW RISK |
| Newman, 1997 | Some Concerns | Some Concerns | High Risk | Low Risk | Low Risk | Some Concerns | HIGH RISK |
| Nordin, 2010 | Some Concerns | Low Risk | Low Risk | Low Risk | Some Concerns | Some Concerns | SOME CONCERNS |
| Oh, 2020 | Some Concerns | Some Concerns | High Risk | Low Risk | Low Risk | Low Risk | SOME CONCERNS |
| Oromendia, 2016 | Some Concerns | Low Risk | Some Concerns | High Risk | Some Concerns | Some Concerns | HIGH RISK |
| Ost, 2004 | Some Concerns | Low Risk | Some Concerns | Low Risk | Low Risk | Some Concerns | SOME CONCERNS |
| Pelissolo, 2012 | Some Concerns | High Risk | Some Concerns | Some Concerns | Some Concerns | Some Concerns | HIGH RISK |
| Petterson, 1996 | Some Concerns | High Risk | Some Concerns | Some Concerns | Some Concerns | Some Concerns | HIGH RISK |
| Pitti, 2015 | Low Risk | High Risk | High Risk | High Risk | Low Risk | Some Concerns | HIGH RISK |
| Reinecke, 2013 | Some Concerns | Some Concerns | Low Risk | Low Risk | Some Concerns | Some Concerns | HIGH RISK |
| Richards, 2006 | High Risk | Low Risk | Low Risk | Some Concerns | Some Concerns | Some Concerns | HIGH RISK |
| Roberge, 2008 | Some Concerns | Low Risk | Some Concerns | Low Risk | Low Risk | Some Concerns | SOME CONCERNS |
| Roy-Byrne, 2005 | Some Concerns | Low Risk | Low Risk | Some Concerns | Some Concerns | Some Concerns | HIGH RISK |
| Roy-Byrne, 2010 | Low Risk | Low Risk | Low Risk | Some Concerns | Low Risk | Some Concerns | LOW RISK |
| Ruwaard, 2010 | Some Concerns | Low Risk | Low Risk | Low Risk | Some Concerns | Some Concerns | SOME CONCERNS |
| Schmidt, 1997a | Some Concerns | High Risk | Low Risk | Some Concerns | Some Concerns | Some Concerns | HIGH RISK |
| Schmidt, 1997b | Some Concerns | High Risk | Low Risk | Low Risk | Some Concerns | Some Concerns | HIGH RISK |
| Sharp, 1997 | Some Concerns | Some Concerns | Low Risk | Some Concerns | Low Risk | Some Concerns | HIGH RISK |
| Sharp, 2000 | Some Concerns | Some Concerns | Low Risk | Low Risk | Low Risk | Some Concerns | SOME CONCERNS |
| Sharp, 2004 | High Risk | Some Concerns | Low Risk | Some Concerns | Some Concerns | Some Concerns | HIGH RISK |
| Shear, 2001 | Some Concerns | Low Risk | Low Risk | High Risk | Low Risk | Some Concerns | SOME CONCERNS |
| Silfvernagel, 2012 | Some Concerns | Low Risk | Low Risk | High Risk | Low Risk | Some Concerns | SOME CONCERNS |
| Telch, 1993 | Some Concerns | Low Risk | Low Risk | Low Risk | Some Concerns | Some Concerns | SOME CONCERNS |
| Titov, 2010 | Some Concerns | Low Risk | Low Risk | Low Risk | Some Concerns | Some Concerns | SOME CONCERNS |
| Tyrer, 1988 | Some Concerns | High Risk | Some Concerns | Low Risk | Low Risk | Some Concerns | HIGH RISK |
| van Ballegooijen, 2013 | Some Concerns | Low Risk | Some Concerns | Some Concerns | Some Concerns | Some Concerns | HIGH RISK |
| Williams, 1996 | Some Concerns | Low Risk | Some Concerns | Low Risk | Some Concerns | Some Concerns | HIGH RISK |
| Wims, 2010 | Some Concerns | Low Risk | Low Risk | Some Concerns | High Risk | Some Concerns | HIGH RISK |

**Risk of bias 2 graph:** review authors' judgements about each risk of bias item presented as percentages across all included studies


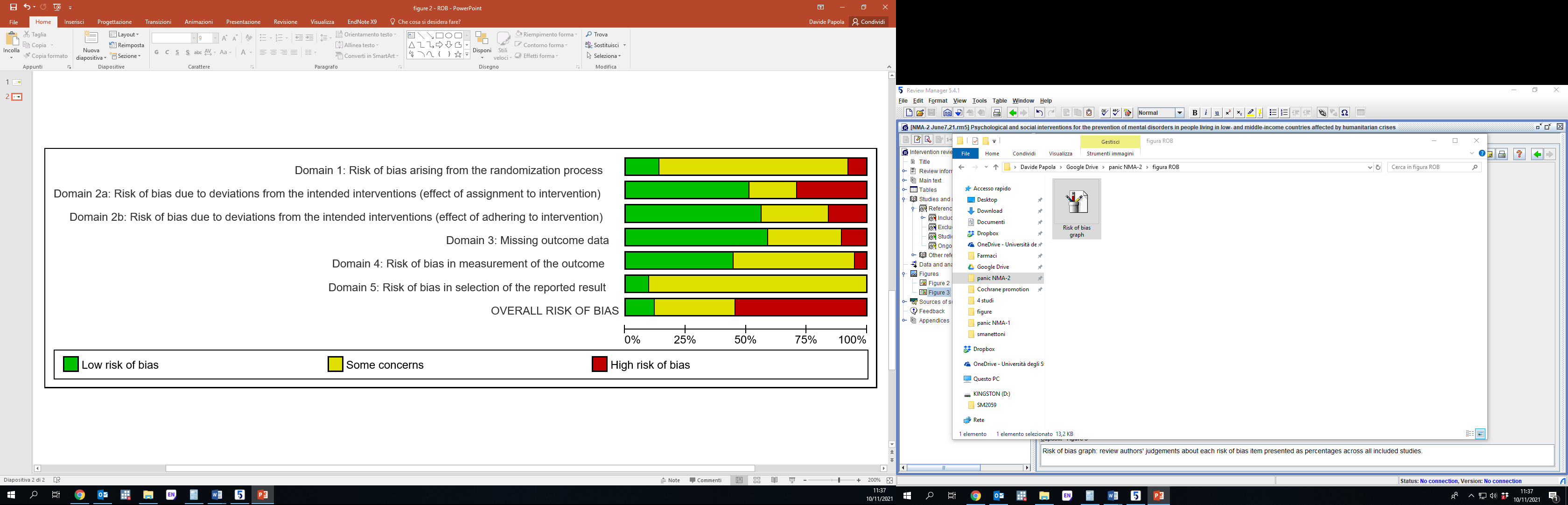


Risk of bias 2 graph: review authors' judgements about each risk of bias item presented as percentages across all included studies. 40 of the 74 RCTs included in the systematic review (54.2%) were found to be at high risk of bias, for 25 (33,7%) studies there were “some concerns”, and 9 (12.1%) were judged to be at low risk of bias.

**Risk of bias tables**

Note. Y = yes; N = no; PY = probably yes; PN = probably no; NI = no information.

### Addis 2004

#### Risk of bias table

| **Bias** | **Authors' judgement** | **Support for judgement** |
| --- | --- | --- |
| Domain 1: Risk of bias arising from the randomization process | Some concerns | 1.1 Was the allocation sequence random? PY  1.2 Was the allocation sequence concealed until participants were enrolled and assigned to interventions? NI;  Comment. Quote: "participants were randomly assigned". No further information.  1.3 Did baseline differences between intervention groups suggest a problem with the randomization process? PN  Comment. Quote:" We found no significant differences between PCT and TAU on any of the pretreatment measures of anxiety, depression, or general well-being. The treatments did not differ on percentages of participants with comorbid axis I diagnoses or medication use at pretreatment. Perceptions of treatment credibility following the second session, as measured by the TCQ, also did not differ between the treatments" |
| Domain 2a: Risk of bias due to deviations from the intended interventions (effect of assignment to intervention) | Low risk | 2.6 Was an appropriate analysis used to estimate the effect of assignment to intervention? Y  Comment. The analysis was carried out on a intention-to-treat basis. |
| Domain 2b: Risk of bias due to deviations from the intended interventions (effect of adhering to intervention) | Low risk | 2.3 Were important non-protocol interventions balanced across intervention groups? Y  Comment. Quote:"To determine possible covariates for the repeated measures analyses we correlated [...] pretreatment medication use with pre–post change scores on the PDSS, FQ, OQ–45, and BDI–1. None of these correlations were statistically significant". The backing analysis is reported in a supplementary file".  2.4. Were there failures in implementing the intervention that could have affected the outcome? PN  2.5. Was there non-adherence to the assigned intervention regimen that could have affected participants’ outcomes? PN |
| Domain 3: Missing outcome data | Low risk | 3.1 Were data for this outcome available for all, or nearly all, participants randomized? PY  Comment. Quote:"Fifteen participants (18.8%, 10 PCT, 5 TAU, Fisher’s exact test, ns) dropped out from the study" |
| Domain 4: Risk of bias in measurement of the outcome | Low risk | 4.1 Was the method of measuring the outcome inappropriate? N  4.2 Could measurement or ascertainment of the outcome have differed between intervention groups? N  4.3 If N/PN/NI to 4.1 and 4.2: Were outcome assessors aware of the intervention received by study participants? N  Comment. Quote: "All raters were blind to treatment condition." |
| Domain 5: Risk of bias in selection of the reported result | Some concerns | 5.1 Were the data that produced this result analysed in accordance with a pre-specified analysis plan that was finalized before unblinded outcome data were available for analysis? PN  Comment. No protocol available.  5.2 Is the numerical result being assessed likely to have been selected, on the basis of the results, from multiple eligible outcome measurements within the outcome domain? N  5.3 Is the numerical result being assessed likely to have been selected, on the basis of the results, from multiple eligible analyses of the data? PN |
| OVERALL RISK OF BIAS | Low risk |  |

### Allen 2016

#### Risk of bias table

| **Bias** | **Authors' judgement** | **Support for judgement** |
| --- | --- | --- |
| Domain 1: Risk of bias arising from the randomization process | Low risk | 1.1 Was the allocation sequence random? Y  1.2 Was the allocation sequence concealed until participants were enrolled and assigned to interventions? Y  Comment. Quote:"Random numbers were generated using a computer random number generator (www.random.org) by a team member who was not involved in the study; this team member placed the group allocation numbers in a sealed opaque envelope. Group allocation was therefore concealed from the interviewer until the offer of participation was made, and the interviewer opened the sealed envelope to inform the participant of their allocated group."  1.3 Did baseline differences between intervention groups suggest a problem with the randomization process? NI  Comment. No information provided. |
| Domain 2a: Risk of bias due to deviations from the intended interventions (effect of assignment to intervention) | Low risk | 2.6 Was an appropriate analysis used to estimate the effect of assignment to intervention? PY  Comment. the analysis was carried out on a intention-to-treat basis. |
| Domain 2b: Risk of bias due to deviations from the intended interventions (effect of adhering to intervention) | Some concerns | 2.3 Were important non-protocol interventions balanced across intervention groups? NI  Comment. No information provided on the balancing of off-protocol medications or other off-protocol interventions across comparison groups.  2.4. Were there failures in implementing the intervention that could have affected the outcome? PN  2.5. Was there non-adherence to the assigned intervention regimen that could have affected participants’ outcomes? PN  2.6. Was an appropriate analysis used to estimate the effect of adhering to the intervention? Y  Comment. Intention-to-treat analysis. |
| Domain 3: Missing outcome data | Low risk | 3.1 Were data for this outcome available for all, or nearly all, participants randomized? Y  Comment. 3 dropouts in the treatment condition, only 1 in the control condition. |
| Domain 4: Risk of bias in measurement of the outcome | Some concerns | 4.1 Was the method of measuring the outcome inappropriate? PN  4.2 Could measurement or ascertainment of the outcome have differed between intervention groups? N  4.3 Were outcome assessors aware of the intervention received by study participants? NI  Comment. only self-reported measures.  4.4 Could assessment of the outcome have been influenced by knowledge of intervention received? PY  Comment. Participants were aware of their treatment allocation and judged the treatment outcome by means of a self-reported questionnaire. Participants allocated to the intervention group may have been more prone to judge favourably the treatment just knowing they had not been allocated to the WL condition.  4.5 Is it likely that assessment of the outcome was influenced by knowledge of intervention received? PN |
| Domain 5: Risk of bias in selection of the reported result | Low risk | 5.1 Were the data that produced this result analysed in accordance with a pre-specified analysis plan that was finalized before unblinded outcome data were available for analysis? Y  Comment. the trial was registered as ACTRN12611001120965  5.2 Is the numerical result being assessed likely to have been selected, on the basis of the results, from multiple eligible outcome measurements within the outcome domain? N  5.3 Is the numerical result being assessed likely to have been selected, on the basis of the results, from multiple eligible analyses of the data? PN |
| OVERALL RISK OF BIAS | Low risk |  |

### Bakker 1999

#### Risk of bias table

| **Bias** | **Authors' judgement** | **Support for judgement** |
| --- | --- | --- |
| Domain 1: Risk of bias arising from the randomization process | Some concerns | 1.1 Was the allocation sequence random? PY  1.2 Was the allocation sequence concealed until participants were enrolled and assigned to interventions? NI  Comment. Quote: "participants were randomly assigned". No further information.  1.3 Did baseline differences between intervention groups suggest a problem with the randomization process? PN  Comment. Quote:"There were no significant differences at pretest between the treatment groups on any of the demographic characteristics or efficacy measures". |
| Domain 2a: Risk of bias due to deviations from the intended interventions (effect of assignment to intervention) | Low risk | 2.6 Was an appropriate analysis used to estimate the effect of assignment to intervention? Y  Comment. the analysis was carried out on a intention-to-treat basis. |
| Domain 2b: Risk of bias due to deviations from the intended interventions (effect of adhering to intervention) | Low risk | 2.3 Were important non-protocol interventions balanced across intervention groups? Y  Comment. Quote:"No concurrent cognitive-behavioral therapy was given during treatment with medication; during cognitive therapy, no psychopharmacologic agents were provided. In all 4 conditions, the use of additional benzodiazepines was prohibited and was monitored by urine tests."  2.4. Were there failures in implementing the intervention that could have affected the outcome? PN  2.5. Was there non-adherence to the assigned intervention regimen that could have affected participants’ outcomes? PN |
| Domain 3: Missing outcome data | Low risk | 3.1 Were data for this outcome available for all, or nearly all, participants randomized? PY  Comment. Quote:"Four patients (12.5%) in the paroxetine group, 3 (9.4%) in the clomipramine group, 9 (25.7%) in the cognitive therapy group, and 2 (6.3%) in the placebo group dropped out between pretest and posttest". |
| Domain 4: Risk of bias in measurement of the outcome | High risk | 4.1 Was the method of measuring the outcome inappropriate? N  4.2 Could measurement or ascertainment of the outcome have differed between intervention groups? N  4.3 Were outcome assessors aware of the intervention received by study participants? NI  Comment. no information provided.  4.4 Could assessment of the outcome have been influenced by knowledge of intervention received? NI  4.5 Is it likely that assessment of the outcome was influenced by knowledge of intervention received? NI |
| Domain 5: Risk of bias in selection of the reported result | Some concerns | 5.1 Were the data that produced this result analysed in accordance with a pre-specified analysis plan that was finalized before unblinded outcome data were available for analysis? PN  Comment. No protocol available.  5.2 Is the numerical result being assessed likely to have been selected, on the basis of the results, from multiple eligible outcome measurements within the outcome domain? N  5.3 Is the numerical result being assessed likely to have been selected, on the basis of the results, from multiple eligible analyses of the data? PN |
| OVERALL RISK OF BIAS | Some concerns | . |

### Barlow 1989

#### Risk of bias table

| **Bias** | **Authors' judgement** | **Support for judgement** |
| --- | --- | --- |
| Domain 1: Risk of bias arising from the randomization process | Some concerns | 1.1 Was the allocation sequence random? PY  1.2 Was the allocation sequence concealed until participants were enrolled and assigned to interventions? NI  Comment. Quote:"Clients were randomly assigned to one of four treatment conditions". No further information provided.  1.3 Did baseline differences between intervention groups suggest a problem with the randomization process? PN  Comment. Quote:"The groups did not differ in terms of subject characteristics- age, duration of the disorder, or sex. Also, they did not differ in terms of any outcome measure- interview scores, standardized self-report questionnaire scores or self-monitoring data. Nor did they differ in terms of the number of stressful events occuring in the six months prior to treatment." |
| Domain 2a: Risk of bias due to deviations from the intended interventions (effect of assignment to intervention) | High risk | 2.6 Was an appropriate analysis used to estimate the effect of assignment to intervention? N  Comment. Analyses were performed on data from completers.  2.7 Was there potential for a substantial impact (on the result) of the failure to analyse participants in the group to which they were randomized? NI |
| Domain 2b: Risk of bias due to deviations from the intended interventions (effect of adhering to intervention) | High risk | 2.3 Were important non-protocol interventions balanced across intervention groups? NI  Comment. No information provided on the balancing of off-protocol medications or other off-protocol interventions across comparison groups.  2.4. Were there failures in implementing the intervention that could have affected the outcome? PN  2.5. Was there non-adherence to the assigned intervention regimen that could have affected participants’ outcomes? PN  2.6. Was an appropriate analysis used to estimate the effect of adhering to the intervention? PN  Comment. Only completers were analyzed. |
| Domain 3: Missing outcome data | Low risk | 3.1 Were data for this outcome available for all, or nearly all, participants randomized? PY  Comment. Quote:"One subject dropped from the WL condition, five from the R condition, one from the E & C condition and four from the combined condition. The number of subjects who completed each condition were (in respective order) 15, 10, 15 and 20. The percentage of dropouts for each condition were, therefore, 6%, 33%, 6%, and 17%." |
| Domain 4: Risk of bias in measurement of the outcome | Some concerns | 4.1 Was the method of measuring the outcome inappropriate? PN  4.2 Could measurement or ascertainment of the outcome have differed between intervention groups? N  4.3 Were outcome assessors aware of the intervention received by study participants? NI  Comment. Quote:"[...]This interview was repeated at post-treatment and at the various follow-up assessments by a blind, independent rater". Nonetheless, the Fear Questionnaire is a self-reported measure and this could have led people in the active groups to perceive therapies as more efficacious than those allocated in the WL group.  4.4 Could assessment of the outcome have been influenced by knowledge of intervention received? PY  Comment. Participants were aware of their treatment allocation and judged the treatment outcome by means of a self-reported questionnaire. Participants allocated to the intervention group may have been more prone to judge favourably the treatment just knowing they had not been allocated to the WL condition.  4.5 Is it likely that assessment of the outcome was influenced by knowledge of intervention received? PN |
| Domain 5: Risk of bias in selection of the reported result | Some concerns | 5.1 Were the data that produced this result analysed in accordance with a pre-specified analysis plan that was finalized before unblinded outcome data were available for analysis? PN  Comment. No protocol available.  5.2 Is the numerical result being assessed likely to have been selected, on the basis of the results, from multiple eligible outcome measurements within the outcome domain? N  5.3 Is the numerical result being assessed likely to have been selected, on the basis of the results, from multiple eligible analyses of the data? PN |
| OVERALL RISK OF BIAS | High risk |  |

### Barlow 2000

#### Risk of bias table

| **Bias** | **Authors' judgement** | **Support for judgement** |
| --- | --- | --- |
| Domain 1: Risk of bias arising from the randomization process | Some concerns | 1.1 Was the allocation sequence random? Y  1.2 Was the allocation sequence concealed until participants were enrolled and assigned to interventions? NI  Comment. Quote: "Randomization was stratified by site and presence of DSM III defined current major depression and was blocked within stratum". No information on allocation concealment.  1.3 Did baseline differences between intervention groups suggest a problem with the randomization process? PN  Comment. Quote: "There were no significant differences on demographic measures or on baseline PDSS score among the 5 randomized groups". |
| Domain 2a: Risk of bias due to deviations from the intended interventions (effect of assignment to intervention) | Low risk | 2.6 Was an appropriate analysis used to estimate the effect of assignment to intervention? Y  Comment. the analysis was carried out on a intention-to-treat basis. |
| Domain 2b: Risk of bias due to deviations from the intended interventions (effect of adhering to intervention) | Low risk | 2.3 Were important non-protocol interventions balanced across intervention groups? PY  Comment. Quote: "Rates of urine samples that tested positive for benzodiazepine use among the 5 treatments were equivalent and low. Thus, we believe benzodiazepine use did not play a significant role in our results."  2.4. Were there failures in implementing the intervention that could have affected the outcome? PN  2.5. Was there non-adherence to the assigned intervention regimen that could have affected participants’ outcomes? PN |
| Domain 3: Missing outcome data | Some concerns | 3.1 Were data for this outcome available for all, or nearly all, participants randomized? PN  Comment. Dropout rate was high:CBT. Radomized: 77; dropout: 21 (27%); TCA (Imipramine).Radomized: 83; dropout: 32 (38%); Placebo.Radomized: 24; dropout: 10 (41%); CBT+Imipramine.Radomized: 65; dropout: 18 (28%); CBT+placebo.Radomized: 63; dropout: 18 (28,5%).  3.2 Is there evidence that the result was not biased by missing outcome data? NI  3.3 Could missingness in the outcome depend on its true value? NI  3.4 Is it likely that missingness in the outcome depended on its true value? NI |
| Domain 4: Risk of bias in measurement of the outcome | Low risk | 4.1 Was the method of measuring the outcome inappropriate? N  4.2 Could measurement or ascertainment of the outcome have differed between intervention groups? N  4.3 Were outcome assessors aware of the intervention received by study participants? N  Comment. Quote: "Evaluator assessments occurred at baseline and after acute, maintenance, and follow-up phases, and evaluators were blind to treatment assignment." |
| Domain 5: Risk of bias in selection of the reported result | Some concerns | 5.1 Were the data that produced this result analysed in accordance with a pre-specified analysis plan that was finalized before unblinded outcome data were available for analysis? PN  Comment. No protocol available.  5.2 Is the numerical result being assessed likely to have been selected, on the basis of the results, from multiple eligible outcome measurements within the outcome domain? N  5.3 Is the numerical result being assessed likely to have been selected, on the basis of the results, from multiple eligible analyses of the data? PN |
| OVERALL RISK OF BIAS | Some concerns | . |

### Beck 1992

#### Risk of bias table

| **Bias** | **Authors' judgement** | **Support for judgement** |
| --- | --- | --- |
| Domain 1: Risk of bias arising from the randomization process | Some concerns | 1.1 Was the allocation sequence random? PY  1.2 Was the allocation sequence concealed until participants were enrolled and assigned to interventions? NI  Comment. Quote: "participants were randomized". No further information.  1.3 Did baseline differences between intervention groups suggest a problem with the randomization process? PN  Comment. Quote: "There were no significant differences between the group that received cognitive therapy only and the group that received brief supportive psychotherapy on any of the demographic and diagnostic variables." |
| Domain 2a: Risk of bias due to deviations from the intended interventions (effect of assignment to intervention) | High risk | 2.6 Was an appropriate analysis used to estimate the effect of assignment to intervention? N  Comment. Per protocol analysis.  2.7 Was there potential for a substantial impact (on the result) of the failure to analyse participants in the group to which they were randomized? PY  Comment. The potential impact (on the estimated effect of intervention) of the failure to analyse participants in the group to which they were randomized was substantial. |
| Domain 2b: Risk of bias due to deviations from the intended interventions (effect of adhering to intervention) | Some concerns | 2.3 Were important non-protocol interventions balanced across intervention groups? NI  Comment. Quote: "it was not possibile to investigate systematically the influence of continued use of medication on the effectiveness of cognitive therapy".  2.4. Were there failures in implementing the intervention that could have affected the outcome? PN  2.5. Was there non-adherence to the assigned intervention regimen that could have affected participants’ outcomes? PN  2.6. Was an appropriate analysis used to estimate the effect of adhering to the intervention? NI |
| Domain 3: Missing outcome data | Low risk | 3.1 Were data for this outcome available for all, or nearly all, participants randomized? Y  Comment. No participant dropped out from the study. |
| Domain 4: Risk of bias in measurement of the outcome | Low risk | 4.1 Was the method of measuring the outcome inappropriate? PN  4.2 Could measurement or ascertainment of the outcome have differed between intervention groups? N  4.3 Were outcome assessors aware of the intervention received by study participants? NI  Comment. Quote: "Data on panic frequency and intensity and depression were obtained from the subjects’ self-reports and the ratings of independent clinicians": it is likely that the "Specific fear inventory" is a self-reported questionnaire.  4.4 Could assessment of the outcome have been influenced by knowledge of intervention received? PN  Comment. Participants were aware of their treatment allocation and judged the treatment outcome by means of a self-reported questionnaire. Nonetheless, people from both groups knew they were both receiving an active intervention, thus participants probably had high and equivalent expectancies in both treatment groups. |
| Domain 5: Risk of bias in selection of the reported result | Some concerns | 5.1 Were the data that produced this result analysed in accordance with a pre-specified analysis plan that was finalized before unblinded outcome data were available for analysis? PN  Comment. No protocol available.  5.2 Is the numerical result being assessed likely to have been selected, on the basis of the results, from multiple eligible outcome measurements within the outcome domain? N  5.3 Is the numerical result being assessed likely to have been selected, on the basis of the results, from multiple eligible analyses of the data? PN |
| OVERALL RISK OF BIAS | High risk |  |

### Berger 2017

#### Risk of bias table

| **Bias** | **Authors' judgement** | **Support for judgement** |
| --- | --- | --- |
| Domain 1: Risk of bias arising from the randomization process | Low risk | 1.1 Was the allocation sequence random? Y  1.2 Was the allocation sequence concealed until participants were enrolled and assigned to interventions? Y  Comment. Quote:"The allocation lists were made using a computerized random number generator and were concealed from the investigators and participants. After the randomization, the participants received an automated email regarding their group allocation.  1.3 Did baseline differences between intervention groups suggest a problem with the randomization process? PN  Comment. Quote:"There were no between-group differences on demographic characteristics or other variables". |
| Domain 2a: Risk of bias due to deviations from the intended interventions (effect of assignment to intervention) | Low risk | 2.6 Was an appropriate analysis used to estimate the effect of assignment to intervention? Y  Comment. Quote:"According to an intention-to-treat principle, dropouts were treated as treatment failures." |
| Domain 2b: Risk of bias due to deviations from the intended interventions (effect of adhering to intervention) | Low risk | 2.3 Were important non-protocol interventions balanced across intervention groups? PY  Comment. Quote:"A stratified randomization procedure was applied, such that a balanced distribution of primary diagnosis, medication and concurrent psychotherapy in the two conditions was ensured."  2.4. Were there failures in implementing the intervention that could have affected the outcome? PN  2.5. Was there non-adherence to the assigned intervention regimen that could have affected participants’ outcomes? PN |
| Domain 3: Missing outcome data | Low risk | 3.1 Were data for this outcome available for all, or nearly all, participants randomized? PY  Comment. 8/48 (16%) patients dropped out from the intervention group, 5/41 (12%) participants dropped out from the comparison group. |
| Domain 4: Risk of bias in measurement of the outcome | Some concerns | 4.1 Was the method of measuring the outcome inappropriate? PN  4.2 Could measurement or ascertainment of the outcome have differed between intervention groups? N  4.3 Were outcome assessors aware of the intervention received by study participants? NI  Comment. Only self-reported measures.  4.4 Could assessment of the outcome have been influenced by knowledge of intervention received? PY  Comment. Participants were aware of their treatment allocation and judged the treatment outcome by means of a self-reported questionnaire. Participants allocated to the intervention group may have been more prone to judge favourably the treatment just knowing they had not been allocated to the WL condition.  4.5 Is it likely that assessment of the outcome was influenced by knowledge of intervention received? PN |
| Domain 5: Risk of bias in selection of the reported result | Low risk | 5.1 Were the data that produced this result analysed in accordance with a pre-specified analysis plan that was finalized before unblinded outcome data were available for analysis? Y  Comment. Quote:"The protocol of this study was approved by the Ethics Committee of the Canton of Bern, and the trial was registered at www.controlled-trials.com (ISRCTN81412545)."  5.2 Is the numerical result being assessed likely to have been selected, on the basis of the results, from multiple eligible outcome measurements within the outcome domain? N  5.3 Is the numerical result being assessed likely to have been selected, on the basis of the results, from multiple eligible analyses of the data? PN |
| OVERALL RISK OF BIAS | Low risk |  |

### Bergstrom 2010

#### Risk of bias table

| **Bias** | **Authors' judgement** | **Support for judgement** |
| --- | --- | --- |
| Domain 1: Risk of bias arising from the randomization process | Some concerns | 1.1 Was the allocation sequence random? Y  1.2 Was the allocation sequence concealed until participants were enrolled and assigned to interventions? Y  Comment. Quote: "The participants were divided into two groups by an independent random-number procedure, where each patient was assigned to either treatment by the opening of sealed numbered envelopes."  1.3 Did baseline differences between intervention groups suggest a problem with the randomization process? PY  Comment. Quote:"Although the proportion of patients taking any psychotropic medication did not differ between groups, patients randomised to the group treatment were to a larger extent on benzodiazepine derivate or neuroleptic medication, and fewer were on SSRI/SNRI medication, than was the Internet group (see Table 1)". |
| Domain 2a: Risk of bias due to deviations from the intended interventions (effect of assignment to intervention) | Low risk | 2.6 Was an appropriate analysis used to estimate the effect of assignment to intervention? Y  Comment. Quote:" a mixed effects models approach was used in the statistical analysis to adjust for these missing values." |
| Domain 2b: Risk of bias due to deviations from the intended interventions (effect of adhering to intervention) | Some concerns | 2.3 Were important non-protocol interventions balanced across intervention groups? PN  Comment. Quote"Patients randomised to the group treatment were to a larger extent on benzodiazepine derivate or neuroleptic medication, and fewer were on SSRI/SNRI medication, than was the Internet group".  2.4. Were there failures in implementing the intervention that could have affected the outcome? PN  2.5. Was there non-adherence to the assigned intervention regimen that could have affected participants’ outcomes? PN  2.6 Was an appropriate analysis used to estimate the effect of adhering to the intervention? Y  Comment. Quote:" a mixed effects models approach was used in the statistical analysis to adjust for these missing values." |
| Domain 3: Missing outcome data | Low risk | 3.1 Were data for this outcome available for all, or nearly all, participants randomized? PY  Comment. Dropout rate: CBT: 60 participants randomized. 11 dropouts (18%) Internet CBT: 53 participants randomized, 9 dropouts (17%) |
| Domain 4: Risk of bias in measurement of the outcome | Low risk | 4.1 Was the method of measuring the outcome inappropriate? N  4.2 Could measurement or ascertainment of the outcome have differed between intervention groups? N  4.3 Were outcome assessors aware of the intervention received by study participants? N  Comment. Quote:"The psychiatrists performing the clinical interviews at post-treatment and follow-up were blind to treatment condition." |
| Domain 5: Risk of bias in selection of the reported result | Some concerns | 5.1 Were the data that produced this result analysed in accordance with a pre-specified analysis plan that was finalized before unblinded outcome data were available for analysis? PN  Comment. No protocol available.  5.2 Is the numerical result being assessed likely to have been selected, on the basis of the results, from multiple eligible outcome measurements within the outcome domain? N  5.3 Is the numerical result being assessed likely to have been selected, on the basis of the results, from multiple eligible analyses of the data? PN |
| OVERALL RISK OF BIAS | Some concerns | . |

### Bohni 2009

#### Risk of bias table

| **Bias** | **Authors' judgement** | **Support for judgement** |
| --- | --- | --- |
| Domain 1: Risk of bias arising from the randomization process | Some concerns | 1.1 Was the allocation sequence random? Y  1.2 Was the allocation sequence concealed until participants were enrolled and assigned to interventions? NI  Comment. Quote:"patients were randomly assigned to the two treatment schedules in blocks of 12 patients. Randomization was performed by a secretary at another institute."  1.3 Did baseline differences between intervention groups suggest a problem with the randomization process? PN  Comment. Quote:"There were no significant differences between groups on any of the baseline characteristics or on any of the measures at pre- treatment (Table 1)". |
| Domain 2a: Risk of bias due to deviations from the intended interventions (effect of assignment to intervention) | Low risk | 2.6 Was an appropriate analysis used to estimate the effect of assignment to intervention? Y  Comment. Last observation carried forward was used to impute missing data at end-point. |
| Domain 2b: Risk of bias due to deviations from the intended interventions (effect of adhering to intervention) | Low risk | 2.3 Were important non-protocol interventions balanced across intervention groups? PY  Comment. Quote:"were no significant differences between groups in terms of changes in medication (P > 0.05)".  2.4. Were there failures in implementing the intervention that could have affected the outcome? PN  2.5. Was there non-adherence to the assigned intervention regimen that could have affected participants’ outcomes? PN |
| Domain 3: Missing outcome data | Low risk | 3.1 Were data for this outcome available for all, or nearly all, participants randomized? Y  Comment. Only one drop out in the SCBT group. |
| Domain 4: Risk of bias in measurement of the outcome | Some concerns | 4.1 Was the method of measuring the outcome inappropriate? PN  4.2 Could measurement or ascertainment of the outcome have differed between intervention groups? N  4.3 Were outcome assessors aware of the intervention received by study participants? NI  Comment. Quote:"The raters were not blinded as to group membership due to logistics within the clinic". Furtermore, ASI is a self-reported instrument.  4.4 Could assessment of the outcome have been influenced by knowledge of intervention received? PN  Comment. Participants were aware of their treatment allocation and judged the treatment outcome by means of a self-reported questionnaire. Nonetheless, people from both groups knew they were both receiving an active intervention, thus participants probably had high and equivalent expectancies in both treatment groups. |
| Domain 5: Risk of bias in selection of the reported result | Some concerns | 5.1 Were the data that produced this result analysed in accordance with a pre-specified analysis plan that was finalized before unblinded outcome data were available for analysis? PN  Comment. No protocol available  5.2 Is the numerical result being assessed likely to have been selected, on the basis of the results, from multiple eligible outcome measurements within the outcome domain? N  5.3 Is the numerical result being assessed likely to have been selected, on the basis of the results, from multiple eligible analyses of the data? PN |
| OVERALL RISK OF BIAS | Some concerns | . |

### Botella 1999

#### Risk of bias table

| **Bias** | **Authors' judgement** | **Support for judgement** |
| --- | --- | --- |
| Domain 1: Risk of bias arising from the randomization process | Some concerns | 1.1 Was the allocation sequence random? PY  1.2 Was the allocation sequence concealed until participants were enrolled and assigned to interventions? NI  Comment. Quote:" Participants were randomly assigned". No further details.  1.3 Did baseline differences between intervention groups suggest a problem with the randomization process? NI  Comment. no information provided. |
| Domain 2a: Risk of bias due to deviations from the intended interventions (effect of assignment to intervention) | Some concerns | 2.6 Was an appropriate analysis used to estimate the effect of assignment to intervention? PN  Comment. no information are provided about how the authors dealt with missing data.  2.7 Was there potential for a substantial impact (on the result) of the failure to analyse participants in the group to which they were randomized? PN  Comment. Only 10% of dropouts in both RCT arms. |
| Domain 2b: Risk of bias due to deviations from the intended interventions (effect of adhering to intervention) | Low risk | 2.3 Were important non-protocol interventions balanced across intervention groups? PY  Comment. Quote: "They were asked to maintain the same medication and the same dosage throughout the research period. However, if a patient increased the dosage or changed the medication, he was excluded from the study. [...] When we compared patients who were taking medication to those who were not, we did not find statistical differences in improvement.".  2.4. Were there failures in implementing the intervention that could have affected the outcome? PN  2.5. Was there non-adherence to the assigned intervention regimen that could have affected participants’ outcomes? PN |
| Domain 3: Missing outcome data | Low risk | 3.1 Were data for this outcome available for all, or nearly all, participants randomized? Y  Comment. Only three dropouts. |
| Domain 4: Risk of bias in measurement of the outcome | High risk | 4.1 Was the method of measuring the outcome inappropriate? N  4.2 Could measurement or ascertainment of the outcome have differed between intervention groups? N  4.3 Were outcome assessors aware of the intervention received by study participants? PY  Comment. Quote:"we have not used an independent assessor because of some practical difficulties."  4.4 Could assessment of the outcome have been influenced by knowledge of intervention received? PY  Comment. If there was an intellectual conflict of interest it is likely that this fact could have influenced the outcome of the study.  4.5 Is it likely that assessment of the outcome was influenced by knowledge of intervention received? NI |
| Domain 5: Risk of bias in selection of the reported result | Some concerns | 5.1 Were the data that produced this result analysed in accordance with a pre-specified analysis plan that was finalized before unblinded outcome data were available for analysis? PN  Comment. No protocol available.  5.2 Is the numerical result being assessed likely to have been selected, on the basis of the results, from multiple eligible outcome measurements within the outcome domain? N  5.3 Is the numerical result being assessed likely to have been selected, on the basis of the results, from multiple eligible analyses of the data? PN |
| OVERALL RISK OF BIAS | High risk |  |

### Carlbring 2001

#### Risk of bias table

| **Bias** | **Authors' judgement** | **Support for judgement** |
| --- | --- | --- |
| Domain 1: Risk of bias arising from the randomization process | Some concerns | 1.1 Was the allocation sequence random? PN  1.2 Was the allocation sequence concealed until participants were enrolled and assigned to interventions? NI  Comment. Quote:"Participants were divided into two groups by the drawing of lots. These were drawn for the two treatment groupings pairwise for participants who had completed their baseline measurements.". Comment: not transparent enough.  1.3 Did baseline differences between intervention groups suggest a problem with the randomization process? NI  Comment. Quote:"The two groups did not differ significantly on any of the measures at pre-treatment. " but no data are shown. |
| Domain 2a: Risk of bias due to deviations from the intended interventions (effect of assignment to intervention) | Low risk | 2.6 Was an appropriate analysis used to estimate the effect of assignment to intervention? Y  Comment. The analysis was carried out on a intention-to-treat basis. |
| Domain 2b: Risk of bias due to deviations from the intended interventions (effect of adhering to intervention) | Some concerns | 2.3 Were important non-protocol interventions balanced across intervention groups? NI  Comment. It is stated that 3 participants withdrew from the study because of "recent commencment of medication", but no other information is provided on the balancing of medication across groups.  2.4. Were there failures in implementing the intervention that could have affected the outcome? PN  2.5. Was there non-adherence to the assigned intervention regimen that could have affected participants’ outcomes? PN |
| Domain 3: Missing outcome data | Low risk | 3.1 Were data for this outcome available for all, or nearly all, participants randomized? PY  Comment. Quote:"After randomization, five people dropped out during the course of the study." |
| Domain 4: Risk of bias in measurement of the outcome | Some concerns | 4.1 Was the method of measuring the outcome inappropriate? PN  4.2 Could measurement or ascertainment of the outcome have differed between intervention groups? N  4.3 Were outcome assessors aware of the intervention received by study participants? NI  Comment. The ACQ is a self-reported questionnaire.  4.4 Could assessment of the outcome have been influenced by knowledge of intervention received? PY  Comment. Participants were aware of their treatment allocation and judged the treatment outcome by means of a self-reported questionnaire. Participants allocated to the intervention group may have been more prone to judge favourably the treatment with respect to those allocated to the WL condition.  4.5 Is it likely that assessment of the outcome was influenced by knowledge of intervention received? NI |
| Domain 5: Risk of bias in selection of the reported result | Some concerns | 5.1 Were the data that produced this result analysed in accordance with a pre-specified analysis plan that was finalized before unblinded outcome data were available for analysis? PN  Comment. No protocol available  5.2 Is the numerical result being assessed likely to have been selected, on the basis of the results, from multiple eligible outcome measurements within the outcome domain? N  5.3 Is the numerical result being assessed likely to have been selected, on the basis of the results, from multiple eligible analyses of the data? PN |
| OVERALL RISK OF BIAS | High risk |  |

### Carlbring 2005

#### Risk of bias table

| **Bias** | **Authors' judgement** | **Support for judgement** |
| --- | --- | --- |
| Domain 1: Risk of bias arising from the randomization process | Some concerns | 1.1 Was the allocation sequence random? Y  1.2 Was the allocation sequence concealed until participants were enrolled and assigned to interventions? NI  Comment. Quote:"Participants were divided into two groups, live therapy (LIVE) or Internet-based (IT) by a true random-number-service (http://www.random.org)."  1.3 Did baseline differences between intervention groups suggest a problem with the randomization process? PN  Comment. Quote:"The two groups did not differ significantly on any of the measures at pre-treatment". |
| Domain 2a: Risk of bias due to deviations from the intended interventions (effect of assignment to intervention) | Low risk | 2.6 Was an appropriate analysis used to estimate the effect of assignment to intervention? Y  Comment. The analysis was carried out on a intention-to-treat basis. |
| Domain 2b: Risk of bias due to deviations from the intended interventions (effect of adhering to intervention) | Low risk | 2.3 Were important non-protocol interventions balanced across intervention groups? PY  Comment. Table 1 shows that participants were taking drugs in a balanced fashion across comparison groups.  2.4. Were there failures in implementing the intervention that could have affected the outcome? PN  2.5. Was there non-adherence to the assigned intervention regimen that could have affected participants’ outcomes? PN |
| Domain 3: Missing outcome data | Low risk | 3.1 Were data for this outcome available for all, or nearly all, participants randomized? PY  Comment. Quote:"After randomization, six people dropped out during the course of the study. There were three dropouts from the LIVE therapy group and three from the IT group. Lack of time was given as the main reason for discontinuing. However, in accordance with the intention to treat paradigm post- treatment data were collected from all dropouts. Six participants did not return their follow-up questionnaires, and their post-treatment scores were carried forward to the follow-up assessment point." |
| Domain 4: Risk of bias in measurement of the outcome | Low risk | 4.1 Was the method of measuring the outcome inappropriate? PN  4.2 Could measurement or ascertainment of the outcome have differed between intervention groups? N  4.3 Were outcome assessors aware of the intervention received by study participants? NI  Comment. The ACQ is a self-reported questionnaire.  4.4 Could assessment of the outcome have been influenced by knowledge of intervention received? PY  Comment. Participants were aware of their treatment allocation and judged the treatment outcome by means of a self-reported questionnaire. Nonetheless, people from both groups knew they were both receiving an active intervention, thus participants probably had high and equivalent expectancies in both treatment groups.  4.5 Is it likely that assessment of the outcome was influenced by knowledge of intervention received? PN  Comment. Quote:"All outcome measures had adequate psychometric properties and were administered via the Internet". |
| Domain 5: Risk of bias in selection of the reported result | Some concerns | 5.1 Were the data that produced this result analysed in accordance with a pre-specified analysis plan that was finalized before unblinded outcome data were available for analysis? PN  Comment. No protocol available  5.2 Is the numerical result being assessed likely to have been selected, on the basis of the results, from multiple eligible outcome measurements within the outcome domain? N  5.3 Is the numerical result being assessed likely to have been selected, on the basis of the results, from multiple eligible analyses of the data? PN |
| OVERALL RISK OF BIAS | Low risk |  |

### Botella 2007

#### Risk of bias table

| **Bias** | **Authors' judgement** | **Support for judgement** |
| --- | --- | --- |
| Domain 1: Risk of bias arising from the randomization process | Low risk | 1.1 Was the allocation sequence random? Y  1.2 Was the allocation sequence concealed until participants were enrolled and assigned to interventions? PY  Comment. Random numbers table was used. Allocation concealment: randomization was performed by an experimenter who did not participate in the study (personal communication).  1.3 Did baseline differences between intervention groups suggest a problem with the randomization process? PN  Comment. Quote:"No differences between the three groups were found at pre-treatment in any of the demographic and clinical variables". |
| Domain 2a: Risk of bias due to deviations from the intended interventions (effect of assignment to intervention) | Some concerns | 2.6 Was an appropriate analysis used to estimate the effect of assignment to intervention? NI  Comment. No information provided.  2.7 Was there potential for a substantial impact (on the result) of the failure to analyse participants in the group to which they were randomized? NI |
| Domain 2b: Risk of bias due to deviations from the intended interventions (effect of adhering to intervention) | Some concerns | 2.3 Were important non-protocol interventions balanced across intervention groups? NI  Comment. Quote:"66.6% of the sample was taking medication for their problem." no other information on the balancing of medication across groups".  2.4. Were there failures in implementing the intervention that could have affected the outcome? PN  2.5. Was there non-adherence to the assigned intervention regimen that could have affected participants’ outcomes? PN  2.6. Was an appropriate analysis used to estimate the effect of adhering to the intervention? NI  Comment. It is difficult to understand how the study authors dealt with data analysis. Nonetheless no participant dropped out from the study. |
| Domain 3: Missing outcome data | Low risk | 3.1 Were data for this outcome available for all, or nearly all, participants randomized? Y  Comment. All of the patients were assessed at study end-point |
| Domain 4: Risk of bias in measurement of the outcome | Low risk | 4.1 Was the method of measuring the outcome inappropriate? N  4.2 Could measurement or ascertainment of the outcome have differed between intervention groups? N  4.3 Were outcome assessors aware of the intervention received by study participants? N  Comment. Assessors were blind to the conditions (personal communication). |
| Domain 5: Risk of bias in selection of the reported result | Some concerns | 5.1 Were the data that produced this result analysed in accordance with a pre-specified analysis plan that was finalized before unblinded outcome data were available for analysis? PN  Comment. No protocol available  5.2 Is the numerical result being assessed likely to have been selected, on the basis of the results, from multiple eligible outcome measurements within the outcome domain? N  5.3 Is the numerical result being assessed likely to have been selected, on the basis of the results, from multiple eligible analyses of the data? PN |
| OVERALL RISK OF BIAS | Some concerns | . |

### Brown 1997

#### Risk of bias table

| **Bias** | **Authors' judgement** | **Support for judgement** |
| --- | --- | --- |
| Domain 1: Risk of bias arising from the randomization process | Some concerns | 1.1 Was the allocation sequence random? PY  1.2 Was the allocation sequence concealed until participants were enrolled and assigned to interventions? NI  Comment. Quote: "Patients were randomly assigned to either FCT or SCT following the initial diagnostic interview". No further information.  1.3 Did baseline differences between intervention groups suggest a problem with the randomization process? NI  Comment. no information provided. |
| Domain 2a: Risk of bias due to deviations from the intended interventions (effect of assignment to intervention) | Low risk | 2.6 Was an appropriate analysis used to estimate the effect of assignment to intervention? PY  Comment. Quote:" We chose to use separate analyses of each measure because of minor differences in the degree of missing data among each of the outcome measures and to facilitate comparisons with other studies." |
| Domain 2b: Risk of bias due to deviations from the intended interventions (effect of adhering to intervention) | Low risk | 2.3 Were important non-protocol interventions balanced across intervention groups? Y  Comment. Quote:"Repeated MANCOVAS were conducted on each outcome measure by medication status (i.e., those patients who took medication throughout the study; those who discontinued medication; those who did not take medication). Results failed to reveal any significant main effect for medication status or any significant medication status by time inter- actions for any outcome measure. In addition, there were no significant group by medication status by time interactions for any dependent measure."  2.4. Were there failures in implementing the intervention that could have affected the outcome? PN  2.5. Was there non-adherence to the assigned intervention regimen that could have affected participants’ outcomes? PN |
| Domain 3: Missing outcome data | Some concerns | 3.1 Were data for this outcome available for all, or nearly all, participants randomized? PN  Comment. Focused cognitive therapy: 21 participants randomized, 4 dropouts (19%); Standard cognitive therapy: 19 participants randomized, 4 dropouts (21%).  3.2 Is there evidence that the result was not biased by missing outcome data? PY  Comment. Drop-out were balanced across the comparison groups. |
| Domain 4: Risk of bias in measurement of the outcome | Low risk | 4.1 Was the method of measuring the outcome inappropriate? N  4.2 Could measurement or ascertainment of the outcome have differed between intervention groups? N  4.3 Were outcome assessors aware of the intervention received by study participants? N  Comment. Quote:"Ratings of patients’ panic attacks, anxiety and depression levels were conducted by postdoctoral independent raters who were blind to the assigned treatment group." |
| Domain 5: Risk of bias in selection of the reported result | Some concerns | 5.1 Were the data that produced this result analysed in accordance with a pre-specified analysis plan that was finalized before unblinded outcome data were available for analysis? PN  Comment. No protocol available  5.2 Is the numerical result being assessed likely to have been selected, on the basis of the results, from multiple eligible outcome measurements within the outcome domain? N  5.3 Is the numerical result being assessed likely to have been selected, on the basis of the results, from multiple eligible analyses of the data? PN |
| OVERALL RISK OF BIAS | Some concerns | . |

### Carlbring 2006

#### Risk of bias table

| **Bias** | **Authors' judgement** | **Support for judgement** |
| --- | --- | --- |
| Domain 1: Risk of bias arising from the randomization process | Some concerns | 1.1 Was the allocation sequence random? Y  1.2 Was the allocation sequence concealed until participants were enrolled and assigned to interventions? NI  Comment. Quote:"The participants were divided into two groups, treatment or a waiting list, by a true random-number service."  1.3 Did baseline differences between intervention groups suggest a problem with the randomization process? PN  Comment. Quote:"The two groups did not differ significantly on any of the measures at pretreatment". |
| Domain 2a: Risk of bias due to deviations from the intended interventions (effect of assignment to intervention) | Low risk | 2.6 Was an appropriate analysis used to estimate the effect of assignment to intervention? Y  Comment. the analysis was carried out on a intention-to-treat basis. |
| Domain 2b: Risk of bias due to deviations from the intended interventions (effect of adhering to intervention) | Some concerns | 2.3 Were important non-protocol interventions balanced across intervention groups? NI  Comment. Quote:"If the participant was taking prescribed drugs for panic dis- order, a) the dosage had to be constant for 3 months before starting treatment, and b) the participant had to agree to keep the dosage constant throughout the study". But no information is reported on the balancing of medication intake across the groups. It is only stated that "Fifty-four percent were taking medication."  2.4. Were there failures in implementing the intervention that could have affected the outcome? PN  2.5. Was there non-adherence to the assigned intervention regimen that could have affected participants’ outcomes? PN  2.6. Was an appropriate analysis used to estimate the effect of adhering to the intervention? Y  Comment. the analysis was carried out on a intention-to-treat basis. |
| Domain 3: Missing outcome data | Low risk | 3.1 Were data for this outcome available for all, or nearly all, participants randomized? Y  Comment. Quote:"Two participants in the treatment condition and one on the waiting list did not re- turn their posttreatment questionnaires. Therefore, their pretreatment scores were carried forward to the posttreat- ment assessment point. Hence, all 60 participants who were randomly assigned to one of the two conditions were included in the statistical analysis." |
| Domain 4: Risk of bias in measurement of the outcome | Low risk | 4.1 Was the method of measuring the outcome inappropriate? PN  4.2 Could measurement or ascertainment of the outcome have differed between intervention groups? N  4.3 Were outcome assessors aware of the intervention received by study participants? NI  Comment. The manuscript is not clear with regard to this matter but it is likely that the assessors were the same authors that administered the therapy. On the other hand, to determine whether participants still fulfilled the criteria for panic disorder at endpoint a clinical telophone interview was administered by independent research assistants, blinded to treatment allocation. At the same time ACQ is a self-reported questionnaire.  4.4 Could assessment of the outcome have been influenced by knowledge of intervention received? PY  Comment. Participants were aware of their treatment allocation and judged the treatment outcome by means of a self-reported questionnaire. Nonetheless, people from both groups knew they were both receiving an active intervention, thus participants probably had high and equivalent expectancies in both treatment groups.  4.5 Is it likely that assessment of the outcome was influenced by knowledge of intervention received? PN  Comment. Quote:"All outcome measures had adequate psychometric properties and were administered via the Internet". |
| Domain 5: Risk of bias in selection of the reported result | Some concerns | 5.1 Were the data that produced this result analysed in accordance with a pre-specified analysis plan that was finalized before unblinded outcome data were available for analysis? PN  Comment. No protocol available  5.2 Is the numerical result being assessed likely to have been selected, on the basis of the results, from multiple eligible outcome measurements within the outcome domain? N  5.3 Is the numerical result being assessed likely to have been selected, on the basis of the results, from multiple eligible analyses of the data? PN |
| OVERALL RISK OF BIAS | Some concerns | . |

### Carter 2003

#### Risk of bias table

| **Bias** | **Authors' judgement** | **Support for judgement** |
| --- | --- | --- |
| Domain 1: Risk of bias arising from the randomization process | Some concerns | 1.1 Was the allocation sequence random? Y  1.2 Was the allocation sequence concealed until participants were enrolled and assigned to interventions? NI  Comment. Quote:"all participants were randomly assigned to either cognitive behavior group treatment or a wait-list condition of equal length. " No further information.  1.3 Did baseline differences between intervention groups suggest a problem with the randomization process? PN  Comment. Quote:"There were no differences between groups on the demographic variables (see Table I)". |
| Domain 2a: Risk of bias due to deviations from the intended interventions (effect of assignment to intervention) | High risk | 2.6 Was an appropriate analysis used to estimate the effect of assignment to intervention? N  Comment. Per protocol analysis. Quote:"We report the data from the remaining 25 patients who completed either treatment or the wait-list assessment."  2.7 Was there potential for a substantial impact (on the result) of the failure to analyse participants in the group to which they were randomized? PY  Comment. Noncompletion rate. Treatment group: 17.6%; waiting list: 26.6%. |
| Domain 2b: Risk of bias due to deviations from the intended interventions (effect of adhering to intervention) | Low risk | 2.3 Were important non-protocol interventions balanced across intervention groups? Y  Comment. Quote:"None of the patients were currently taking antianxiety or antidepressant medications. Only two patients in the treatment group and one in the wait-list condition had previously received treatment. Each described their therapy as “general” and focusing on family issues or depression. None described their treatment as effective."  2.4. Were there failures in implementing the intervention that could have affected the outcome? PN  2.5. Was there non-adherence to the assigned intervention regimen that could have affected participants’ outcomes? PN |
| Domain 3: Missing outcome data | High risk | 3.1 Were data for this outcome available for all, or nearly all, participants randomized? PN  Comment. Noncompletion rate. Treatment group: 17.6%; waiting list: 26.6%.  3.2 Is there evidence that the result was not biased by missing outcome data? N |
| Domain 4: Risk of bias in measurement of the outcome | Some concerns | 4.1 Was the method of measuring the outcome inappropriate? PN  4.2 Could measurement or ascertainment of the outcome have differed between intervention groups? N  4.3 Were outcome assessors aware of the intervention received by study participants? NI  Comment. Quote:"All interviews were administered by advanced clinical psychology graduate students blind to group assignment at pre- and posttest". Nonetheless, the ASI is a self-reported questionnaire.  4.4 Could assessment of the outcome have been influenced by knowledge of intervention received? PY  Comment. Participants were aware of their treatment allocation and judged the treatment outcome by means of a self-reported questionnaire. Participants allocated to the intervention group may have been more prone to judge favourably the treatment with respect to those allocated to the WL condition.  4.5 Is it likely that assessment of the outcome was influenced by knowledge of intervention received? NI |
| Domain 5: Risk of bias in selection of the reported result | Some concerns | 5.1 Were the data that produced this result analysed in accordance with a pre-specified analysis plan that was finalized before unblinded outcome data were available for analysis? PN  Comment. No protocol available  5.2 Is the numerical result being assessed likely to have been selected, on the basis of the results, from multiple eligible outcome measurements within the outcome domain? N  5.3 Is the numerical result being assessed likely to have been selected, on the basis of the results, from multiple eligible analyses of the data? PN |
| OVERALL RISK OF BIAS | High risk |  |

### Choi 2005

#### Risk of bias table

| **Bias** | **Authors' judgement** | **Support for judgement** |
| --- | --- | --- |
| Domain 1: Risk of bias arising from the randomization process | Some concerns | 1.1 Was the allocation sequence random? PY  1.2 Was the allocation sequence concealed until participants were enrolled and assigned to interventions? NI  Comment. Quote:" 20 subjects were assigned randomly into ExCT, and the re- mainder 20 subjects into PCP." No further information.  1.3 Did baseline differences between intervention groups suggest a problem with the randomization process? PN  Comment. Quote:"There were no significant differences in gender, age, education, marital status, and duration of illness between the two groups (Table1)." |
| Domain 2a: Risk of bias due to deviations from the intended interventions (effect of assignment to intervention) | Some concerns | 2.6 Was an appropriate analysis used to estimate the effect of assignment to intervention? NI  Comment. Quote:"To compare pretest and posttest scores in the ExCT And PCP groups, the data were analyzed using t-tests." This is the only sentence about the statistical analysis in the manuscript.  2.7 Was there potential for a substantial impact (on the result) of the failure to analyse participants in the group to which they were randomized? Apparently no study participant dropped out from the study, but no information on drop out rate is reported in the text. |
| Domain 2b: Risk of bias due to deviations from the intended interventions (effect of adhering to intervention) | Low risk | 2.3 Were important non-protocol interventions balanced across intervention groups? PY  Comment. Quote:"The rate of discontinuation of medication was not different significantly between two groups at post-treatment".  2.4. Were there failures in implementing the intervention that could have affected the outcome? PN  2.5. Was there non-adherence to the assigned intervention regimen that could have affected participants’ outcomes? PN |
| Domain 3: Missing outcome data | Low risk | 3.1 Were data for this outcome available for all, or nearly all, participants randomized? PY  Comment. Apparently no study participant dropped out from the study, but no information on drop out rate is reported in the text. |
| Domain 4: Risk of bias in measurement of the outcome | Low risk | 4.1 Was the method of measuring the outcome inappropriate? PN  4.2 Could measurement or ascertainment of the outcome have differed between intervention groups? N  4.3 Were outcome assessors aware of the intervention received by study participants? NI  Comment. the ASI is a self-reported questionnaire.  4.4 Could assessment of the outcome have been influenced by knowledge of intervention received? PN  Comment. Participants were aware of their treatment allocation and judged the treatment outcome by means of a self-reported questionnaire. Nonetheless, people from both groups knew they were both receiving an active intervention, thus participants probably had high and equivalent expectancies in both treatment groups. |
| Domain 5: Risk of bias in selection of the reported result | Some concerns | 5.1 Were the data that produced this result analysed in accordance with a pre-specified analysis plan that was finalized before unblinded outcome data were available for analysis? PN  Comment. No protocol available  5.2 Is the numerical result being assessed likely to have been selected, on the basis of the results, from multiple eligible outcome measurements within the outcome domain? N  5.3 Is the numerical result being assessed likely to have been selected, on the basis of the results, from multiple eligible analyses of the data? PN |
| OVERALL RISK OF BIAS | Some concerns | . |

### Christoforou 2017

#### Risk of bias table

| **Bias** | **Authors' judgement** | **Support for judgement** |
| --- | --- | --- |
| Domain 1: Risk of bias arising from the randomization process | Low risk | 1.1 Was the allocation sequence random? Y  1.2 Was the allocation sequence concealed until participants were enrolled and assigned to interventions? Y  Comment. Randomization process and allocation concealment strategies were carefully described.  1.3 Did baseline differences between intervention groups suggest a problem with the randomization process? PN  Comment. Quote:"Table 2 shows the demographic and clinical baseline characteristics of the participants by group. The 2 groups did not differ statistically on any of those characteristics at baseline (all P>.05)." |
| Domain 2a: Risk of bias due to deviations from the intended interventions (effect of assignment to intervention) | Low risk | 2.6 Was an appropriate analysis used to estimate the effect of assignment to intervention? Y  Comment. The analysis was carried out on a intention-to-treat basis. |
| Domain 2b: Risk of bias due to deviations from the intended interventions (effect of adhering to intervention) | Some concerns | 2.3 Were important non-protocol interventions balanced across intervention groups? NI  Comment. no information provided on the balancing of off-protocol medications or other off-protocol interventions across comparison groups.  2.4. Were there failures in implementing the intervention that could have affected the outcome? PN  2.5. Was there non-adherence to the assigned intervention regimen that could have affected participants’ outcomes? PN  2.6. Was an appropriate analysis used to estimate the effect of adhering to the intervention? Y  Comment. The analysis was carried out on a intention-to-treat basis. |
| Domain 3: Missing outcome data | Some concerns | 3.1 Were data for this outcome available for all, or nearly all, participants randomized? PN  Comment. Less than half of the randomized participants were evaluated at endpoint.  3.2 Is there evidence that the result was not biased by missing outcome data? PY  Comment. ITT analysis was conducted taking in account also participants who dropped out from the study |
| Domain 4: Risk of bias in measurement of the outcome | Low risk | 4.1 Was the method of measuring the outcome inappropriate? PN  4.2 Could measurement or ascertainment of the outcome have differed between intervention groups? N  4.3 Were outcome assessors aware of the intervention received by study participants? PN  Comment. Quote:"The trial was assessor blinded, as researchers were blinded to treatment allocation throughout the trial and during the statistical analysis. This was achieved by having a person outside the research team to manage treatment allocation and personal communications with the participants." Nonetheless the questionnaire was self-administred (PAS - panic and agoraphobia scale). |
| Domain 5: Risk of bias in selection of the reported result | Some concerns | 5.1 Were the data that produced this result analysed in accordance with a pre-specified analysis plan that was finalized before unblinded outcome data were available for analysis? PN  Comment. No protocol available  5.2 Is the numerical result being assessed likely to have been selected, on the basis of the results, from multiple eligible outcome measurements within the outcome domain? N  5.3 Is the numerical result being assessed likely to have been selected, on the basis of the results, from multiple eligible analyses of the data? PN |
| OVERALL RISK OF BIAS | Some concerns | . |

### Ciuca 2018

#### Risk of bias table

| **Bias** | **Authors' judgement** | **Support for judgement** |
| --- | --- | --- |
| Domain 1: Risk of bias arising from the randomization process | Low risk | 1.1 Was the allocation sequence random? Y  1.2 Was the allocation sequence concealed until participants were enrolled and assigned to interventions? Y  Comment. Randomization process and allocation concealment strategies were carefully described.  1.3 Did baseline differences between intervention groups suggest a problem with the randomization process? PN  Comment. Quote:"Groups did not differ significantly in regard to age, computer skills and income level, comorbidity or other demographic characteristics (see Table 2)." |
| Domain 2a: Risk of bias due to deviations from the intended interventions (effect of assignment to intervention) | Low risk | 2.6 Was an appropriate analysis used to estimate the effect of assignment to intervention? Y  Comment. the analysis was carried out on a intention-to-treat basis. |
| Domain 2b: Risk of bias due to deviations from the intended interventions (effect of adhering to intervention) | Some concerns | 2.3 Were important non-protocol interventions balanced across intervention groups? NI  Comment. No information provided on the balancing of off-protocol medications or other off-protocol interventions across comparison groups.  2.4. Were there failures in implementing the intervention that could have affected the outcome? PN  2.5. Was there non-adherence to the assigned intervention regimen that could have affected participants’ outcomes? PN  2.6. Was an appropriate analysis used to estimate the effect of adhering to the intervention? Y  Comment. The analysis was carried out on a intention-to-treat basis. |
| Domain 3: Missing outcome data | Some concerns | 3.1 Were data for this outcome available for all, or nearly all, participants randomized? PN  Comment. Quote:"In total, 30 participants (27%) failed to complete the post-treatment assessment (7 in the guided treatment condition, 12 in the unguided treatment condition and 11 in the WL)."  3.2 Is there evidence that the result was not biased by missing outcome data? PY  Comment. ITT analysis was conducted taking in account also participants who dropped out from the study. |
| Domain 4: Risk of bias in measurement of the outcome | Some concerns | 4.1 Was the method of measuring the outcome inappropriate? PN  4.2 Could measurement or ascertainment of the outcome have differed between intervention groups? N  4.3 Were outcome assessors aware of the intervention received by study participants? NI  Comment. Quote:"The assessors could not be kept completely blind regarding group allocation because some participants revealed information about the treatment during the interview." The PDSS-SR is a self-reported measure.  4.4 Could assessment of the outcome have been influenced by knowledge of intervention received? PY  Comment. Participants were aware of their treatment allocation and judged the treatment outcome by means of a self-reported questionnaire. Participants allocated to the intervention group may have been more prone to judge favourably the treatment just knowing they had not been allocated to the WL condition.  4.5 Is it likely that assessment of the outcome was influenced by knowledge of intervention received? PN |
| Domain 5: Risk of bias in selection of the reported result | Low risk | 5.1 Were the data that produced this result analysed in accordance with a pre-specified analysis plan that was finalized before unblinded outcome data were available for analysis? Y  Comment. Protocol available with rigorous description of statistical data planning.  5.2 Is the numerical result being assessed likely to have been selected, on the basis of the results, from multiple eligible outcome measurements within the outcome domain? N  5.3 Is the numerical result being assessed likely to have been selected, on the basis of the results, from multiple eligible analyses of the data? PN |
| OVERALL RISK OF BIAS | Some concerns | . |

### Craske 2003

#### Risk of bias table

| **Bias** | **Authors' judgement** | **Support for judgement** |
| --- | --- | --- |
| Domain 1: Risk of bias arising from the randomization process | Some concerns | 1.1 Was the allocation sequence random? PY  1.2 Was the allocation sequence concealed until participants were enrolled and assigned to interventions? NI  Comment. Quote:" Participants were randomly assigned". No further details.  1.3 Did baseline differences between intervention groups suggest a problem with the randomization process? PN  Comment. The only imbalance was about the age of the participants in the two groups. |
| Domain 2a: Risk of bias due to deviations from the intended interventions (effect of assignment to intervention) | Some concerns | 2.6 Was an appropriate analysis used to estimate the effect of assignment to intervention? PN  Comment. No information provided weather the analysis was IIT or per protocol.  2.7 Was there potential for a substantial impact (on the result) of the failure to analyse participants in the group to which they were randomized? PN  Comment. Quote:"Of the 68 participants that were randomized, a total of 16.2% dropped (n=9) or were removed (n=2) throughout the intervention: 11.8% of those assigned to PCT+IV compared to 20.6% of those assigned to PCT, non-significant (ns)". |
| Domain 2b: Risk of bias due to deviations from the intended interventions (effect of adhering to intervention) | Low risk | 2.3 Were important non-protocol interventions balanced across intervention groups? PY  Comment. Roughly the same percentage of participants took medications in each comparison group.  2.4. Were there failures in implementing the intervention that could have affected the outcome? PN  2.5. Was there non-adherence to the assigned intervention regimen that could have affected participants’ outcomes? PN |
| Domain 3: Missing outcome data | High risk | 3.1 Were data for this outcome available for all, or nearly all, participants randomized? PN  Comment. Drop-out rate was around 20% for the PCT group and 12% for PCT+IV group.  3.2 Is there evidence that the result was not biased by missing outcome data? PN  Comment. Taking that the data shown in table 3 are from a per protocol analysis approach, a imbalance between drop-out rates in the 2 comparison groups could have lead to biased results.  3.3 Could missingness in the outcome depend on its true value? NI  3.4 Is it likely that missingness in the outcome depended on its true value? NI |
| Domain 4: Risk of bias in measurement of the outcome | Some concerns | 4.1 Was the method of measuring the outcome inappropriate? N  4.2 Could measurement or ascertainment of the outcome have differed between intervention groups? N  4.3 Were outcome assessors aware of the intervention received by study participants? NI  Comment. no information provided.  4.4 Could assessment of the outcome have been influenced by knowledge of intervention received? NI  4.5 Is it likely that assessment of the outcome was influenced by knowledge of intervention received?  NI |
| Domain 5: Risk of bias in selection of the reported result | Some concerns | 5.1 Were the data that produced this result analysed in accordance with a pre-specified analysis plan that was finalized before unblinded outcome data were available for analysis? PN  Comment. No protocol available.  5.2 Is the numerical result being assessed likely to have been selected, on the basis of the results, from multiple eligible outcome measurements within the outcome domain? N  5.3 Is the numerical result being assessed likely to have been selected, on the basis of the results, from multiple eligible analyses of the data? PN |
| OVERALL RISK OF BIAS | High risk |  |

### Clark 1999

#### Risk of bias table

| **Bias** | **Authors' judgement** | **Support for judgement** |
| --- | --- | --- |
| Domain 1: Risk of bias arising from the randomization process | Some concerns | 1.1 Was the allocation sequence random? PY  1.2 Was the allocation sequence concealed until participants were enrolled and assigned to interventions? NI  Comment. Quote: "participants were randomized". No further information.  1.3 Did baseline differences between intervention groups suggest a problem with the randomization process? PN  Comment. Quote:"At pretreatment no tests were significant (all ps > .30), indicating that the groups did not differ before the start of treatment or wait list." but no data are shown. |
| Domain 2a: Risk of bias due to deviations from the intended interventions (effect of assignment to intervention) | Some concerns | 2.6 Was an appropriate analysis used to estimate the effect of assignment to intervention? PN  Comment. The analyses were carried out on the data collected at every time point. No mention of how the authors handled missing data.  2.7 Was there potential for a substantial impact (on the result) of the failure to analyse participants in the group to which they were randomized? N  Comment. Just 1 drop-out out of 43 randomized participants |
| Domain 2b: Risk of bias due to deviations from the intended interventions (effect of adhering to intervention) | Low risk | 2.3 Were important non-protocol interventions balanced across intervention groups? PY  Comment. Quote:"No patients increased their medication during the trial, but 50% (5/10) of treated patients (2 FCT, 3 BCT) and 25% (1/4) of wait-list patients discontinued their medication between the pretreatment/wait-list and posttreatment/wait-list assessments. Given this pattern of results, it seems highly unlikely that medication could account for the effectiveness of FCT and BCT."  2.4. Were there failures in implementing the intervention that could have affected the outcome? PN  2.5. Was there non-adherence to the assigned intervention regimen that could have affected participants’ outcomes? PN |
| Domain 3: Missing outcome data | Low risk | 3.1 Were data for this outcome available for all, or nearly all, participants randomized? Y  Comment. Only 1 drop-out among 43 randomized participants. |
| Domain 4: Risk of bias in measurement of the outcome | Some concerns | 4.1 Was the method of measuring the outcome inappropriate? PN  4.2 Could measurement or ascertainment of the outcome have differed between intervention groups? N  4.3 Were outcome assessors aware of the intervention received by study participants? NI  Comment. Assessors were blind to treatment allocation, but the ACQ is a self-reported instrument.  4.4 Could assessment of the outcome have been influenced by knowledge of intervention received? PY  Comment. Participants were aware of their treatment allocation and judged the treatment outcome by means of a self-reported questionnaire. Participants allocated to the intervention group may have been more prone to judge favourably the treatment just knowing they had not been allocated to the WL condition.  4.5 Is it likely that assessment of the outcome was influenced by knowledge of intervention received? PN |
| Domain 5: Risk of bias in selection of the reported result | Some concerns | 5.1 Were the data that produced this result analysed in accordance with a pre-specified analysis plan that was finalized before unblinded outcome data were available for analysis? PN  Comment. No protocol available.  5.2 Is the numerical result being assessed likely to have been selected, on the basis of the results, from multiple eligible outcome measurements within the outcome domain? N  5.3 Is the numerical result being assessed likely to have been selected, on the basis of the results, from multiple eligible analyses of the data? PN |
| OVERALL RISK OF BIAS | High risk |  |

### Craske 1997

#### Risk of bias table

| **Bias** | **Authors' judgement** | **Support for judgement** |
| --- | --- | --- |
| Domain 1: Risk of bias arising from the randomization process | Some concerns | 1.1 Was the allocation sequence random? PY  1.2 Was the allocation sequence concealed until participants were enrolled and assigned to interventions? NI  Comment.  1.3 Did baseline differences between intervention groups suggest a problem with the randomization process? PN  Comment. Quote:"CIE (N = 20) and CBE (N = 18) completers were compared on pre-demographics, diagnostic profile and dependent measures, using Fisher’s exact tests and independent t tests. The groups did not differ (alpha = .05)." |
| Domain 2a: Risk of bias due to deviations from the intended interventions (effect of assignment to intervention) | High risk | 2.6 Was an appropriate analysis used to estimate the effect of assignment to intervention? N  Comment. Only completers were analyzed (per-protocol approach).  2.7 Was there potential for a substantial impact (on the result) of the failure to analyse participants in the group to which they were randomized? PY  Comment. Drop-out rate above 20%. |
| Domain 2b: Risk of bias due to deviations from the intended interventions (effect of adhering to intervention) | High risk | 2.3 Were important non-protocol interventions balanced across intervention groups? NI  Comment. No information provided on the balancing of off-protocol medications or other off-protocol interventions across comparison groups.  2.4. Were there failures in implementing the intervention that could have affected the outcome? PN  2.5. Was there non-adherence to the assigned intervention regimen that could have affected participants’ outcomes? PN  2.6. Was an appropriate analysis used to estimate the effect of adhering to the intervention? N  Comment. Data available from table 3 refer to a per protocol analysis. |
| Domain 3: Missing outcome data | Some concerns | 3.1 Were data for this outcome available for all, or nearly all, participants randomized? PN  Comment. Attrition rate higher than 20%.  3.2 Is there evidence that the result was not biased by missing outcome data? NI  3.3 Could missingness in the outcome depend on its true value? NI  3.4 Is it likely that missingness in the outcome depended on its true value? NI |
| Domain 4: Risk of bias in measurement of the outcome | Low risk | 4.1 Was the method of measuring the outcome inappropriate? N  4.2 Could measurement or ascertainment of the outcome have differed between intervention groups? N  4.3 Were outcome assessors aware of the intervention received by study participants? N  Comment. Quote:"An abbreviated ADIS-R was readministered at post-treatment and follow-up by independent assessors, blind to treatment condition".  4.4 Could assessment of the outcome have been influenced by knowledge of intervention received? PN  Comment. Participants were aware of their treatment allocation and judged the treatment outcome by means of a self-reported questionnaire. Nonetheless, people from both groups knew they were both receiving an active intervention, thus participants probably had high and equivalent expectancies in both treatment groups. |
| Domain 5: Risk of bias in selection of the reported result | Some concerns | 5.1 Were the data that produced this result analysed in accordance with a pre-specified analysis plan that was finalized before unblinded outcome data were available for analysis? PN  Comment. No protocol available  5.2 Is the numerical result being assessed likely to have been selected, on the basis of the results, from multiple eligible outcome measurements within the outcome domain? N  5.3 Is the numerical result being assessed likely to have been selected, on the basis of the results, from multiple eligible analyses of the data? PN |
| OVERALL RISK OF BIAS | High risk |  |

### Craske 2005

#### Risk of bias table

| **Bias** | **Authors' judgement** | **Support for judgement** |
| --- | --- | --- |
| Domain 1: Risk of bias arising from the randomization process | High risk | 1.1 Was the allocation sequence random? PY  1.2 Was the allocation sequence concealed until participants were enrolled and assigned to interventions? NI  Comment. Quote:" Participants were randomly assigned". No further details.  1.3 Did baseline differences between intervention groups suggest a problem with the randomization process? PY  Comment. Quote:"CBT and WL groups did not differ on any demographic, psychological or percent withdrawn from medication variables, except rates of social anxiety disorder: 44.4% of CBT group versus 6.3% of the WL group." |
| Domain 2a: Risk of bias due to deviations from the intended interventions (effect of assignment to intervention) | Some concerns | 2.6 Was an appropriate analysis used to estimate the effect of assignment to intervention? PN  Comment. No information provided weather the analysis was IIT or per protocol.  2.7 Was there potential for a substantial impact (on the result) of the failure to analyse participants in the group to which they were randomized?  Comment. 3 out of 27 withdrew from CBT group, none withdrew from the WL group. |
| Domain 2b: Risk of bias due to deviations from the intended interventions (effect of adhering to intervention) | Low risk | 2.3 Were important non-protocol interventions balanced across intervention groups? PY  Comment. Quote:"We selected patients who were free from medications and other sleep-related disorders that may account for their sleep distur- bance and who were suffering from nocturnal panic attacks on a regular basis, averaging six per month."  2.4. Were there failures in implementing the intervention that could have affected the outcome? PN  2.5. Was there non-adherence to the assigned intervention regimen that could have affected participants’ outcomes? PN |
| Domain 3: Missing outcome data | Low risk | 3.1 Were data for this outcome available for all, or nearly all, participants randomized? Y  Comment. only 4 drop-outs out of 43 participants. |
| Domain 4: Risk of bias in measurement of the outcome | Some concerns | 4.1 Was the method of measuring the outcome inappropriate? PN  4.2 Could measurement or ascertainment of the outcome have differed between intervention groups? N  4.3 Were outcome assessors aware of the intervention received by study participants? NI  Comment. No information provided. The ASI is a self-reported outcome.  4.4 Could assessment of the outcome have been influenced by knowledge of intervention received? PY  Comment. Participants were aware of their treatment allocation and judged the treatment outcome by means of a self-reported questionnaire. Participants allocated to the intervention group may have been more prone to judge favourably the treatment just knowing they had not been allocated to the WL condition.  4.5 Is it likely that assessment of the outcome was influenced by knowledge of intervention received? PN |
| Domain 5: Risk of bias in selection of the reported result | Some concerns | 5.1 Were the data that produced this result analysed in accordance with a pre-specified analysis plan that was finalized before unblinded outcome data were available for analysis? PN  Comment. No protocol available  5.2 Is the numerical result being assessed likely to have been selected, on the basis of the results, from multiple eligible outcome measurements within the outcome domain? N  5.3 Is the numerical result being assessed likely to have been selected, on the basis of the results, from multiple eligible analyses of the data? PN |
| OVERALL RISK OF BIAS | High risk |  |

### Craske 2007

#### Risk of bias table

| **Bias** | **Authors' judgement** | **Support for judgement** |
| --- | --- | --- |
| Domain 1: Risk of bias arising from the randomization process | Some concerns | 1.1 Was the allocation sequence random? PY  1.2 Was the allocation sequence concealed until participants were enrolled and assigned to interventions? NI  Comment. Quote:"Of 65 participants, 33 were randomized to PDA and 32 to PDA+C."  1.3 Did baseline differences between intervention groups suggest a problem with the randomization process? PN  Comment. PDA+C and PDA groups did not differ on any demographic, diagnostic, self-report or medication variables (see Tables 1 and 2. |
| Domain 2a: Risk of bias due to deviations from the intended interventions (effect of assignment to intervention) | Some concerns | 2.6 Was an appropriate analysis used to estimate the effect of assignment to intervention? PN  Comment. Both per protocol and ITT analyses were carried out but tables' data are only available for per protocol analysis.  2.7 Was there potential for a substantial impact (on the result) of the failure to analyse participants in the group to which they were randomized? PN  Comment. Quote:"Ten participants (15%) withdrew during treatment; 4 (12.5%) from PDA+C and 6 (18.2%) from PDA, ns." |
| Domain 2b: Risk of bias due to deviations from the intended interventions (effect of adhering to intervention) | Low risk | 2.3 Were important non-protocol interventions balanced across intervention groups? Y  Comment. Quote:"The groups did not differ in the proportions receiving medications at baseline (PDA+C: 54.5%; PDA: 39.1%), at post-treatment (PDA+C: 40%; PDA: 45.8%) or at follow-up (PDA+C: 43.5%; PDA: 54.5%). Similarly, no differences were found in the proportions receiving additional psychotherapy at baseline and at post-treatment (PDA+C: 24%; PDA: 18.5%) or at follow-up (PDA+C: 26.1%; PDA: 17.4%)."  2.4. Were there failures in implementing the intervention that could have affected the outcome? PN  2.5. Was there non-adherence to the assigned intervention regimen that could have affected participants’ outcomes? PN |
| Domain 3: Missing outcome data | Low risk | 3.1 Were data for this outcome available for all, or nearly all, participants randomized? PY  Comment. Drop-out rates were below 20% of participants in both groups. |
| Domain 4: Risk of bias in measurement of the outcome | Low risk | 4.1 Was the method of measuring the outcome inappropriate? N  4.2 Could measurement or ascertainment of the outcome have differed between intervention groups? N  4.3 Were outcome assessors aware of the intervention received by study participants? N  Comment. Quote:"diagnostic interview was repeated at each assessment by interviewers who were blind to the participant’s treatment assignment." The ASI is a self-reported questionnaire.  4.4 Could assessment of the outcome have been influenced by knowledge of intervention received? PN  Comment. Participants were aware of their treatment allocation and judged the treatment outcome by means of a self-reported questionnaire. Nonetheless, people from both groups knew they were both receiving an active intervention, thus participants probably had high and equivalent expectancies in both treatment groups. |
| Domain 5: Risk of bias in selection of the reported result | Some concerns | 5.1 Were the data that produced this result analysed in accordance with a pre-specified analysis plan that was finalized before unblinded outcome data were available for analysis? PN  Comment. No protocol available.  5.2 Is the numerical result being assessed likely to have been selected, on the basis of the results, from multiple eligible outcome measurements within the outcome domain? N  5.3 Is the numerical result being assessed likely to have been selected, on the basis of the results, from multiple eligible analyses of the data? PN |
| OVERALL RISK OF BIAS | Some concerns | . |

### Craske 2011

#### Risk of bias table

| **Bias** | **Authors' judgement** | **Support for judgement** |
| --- | --- | --- |
| Domain 1: Risk of bias arising from the randomization process | Low risk | 1.1 Was the allocation sequence random? Y  1.2 Was the allocation sequence concealed until participants were enrolled and assigned to interventions? Y  Comment. Quote: "After baseline assessment, participants were randomized using stratified (by clinic and presence ofcomorbid major depression) permuted block randomization to receive ITV or UC by an automated program at RAND.Block size was masked to all clinical site study members."  1.3 Did baseline differences between intervention groups suggest a problem with the randomization process? PN  Comment. Quote:"Most demographic characteristics were similar across the ITV and UC groups in each principal anxiety disorder group (Table 1). There was some imbalance in educational achievement, ethnicity, number of comorbid anxiety disorders, and GAD for PD only, which were used as covariates in the analyses." |
| Domain 2a: Risk of bias due to deviations from the intended interventions (effect of assignment to intervention) | Some concerns | 2.6 Was an appropriate analysis used to estimate the effect of assignment to intervention? PN  Comment. the general analysis plan is described as ITT but the specific result for PD subpopulation was reported by taking into account completers only.  2.7 Was there potential for a substantial impact (on the result) of the failure to analyse participants in the group to which they were randomized? PN |
| Domain 2b: Risk of bias due to deviations from the intended interventions (effect of adhering to intervention) | High risk | 2.3 Were important non-protocol interventions balanced across intervention groups? NI  Comment. no information provided on the balancing of off-protocol medications or other off-protocol interventions across comparison groups.  2.4. Were there failures in implementing the intervention that could have affected the outcome? PN  2.5. Was there non-adherence to the assigned intervention regimen that could have affected participants’ outcomes? PN  2.6. Was an appropriate analysis used to estimate the effect of adhering to the intervention? PN  Comment. Data available from table 3 refer to a per protocol analysis. |
| Domain 3: Missing outcome data | Low risk | 3.1 Were data for this outcome available for all, or nearly all, participants randomized? PY  Comment. 15/126 (12%) patients dropped out from the intervention group, 24/136 (18%) participants dropped out from the comparison group. |
| Domain 4: Risk of bias in measurement of the outcome | Some concerns | 4.1 Was the method of measuring the outcome inappropriate? N  4.2 Could measurement or ascertainment of the outcome have differed between intervention groups? N  4.3 Were outcome assessors aware of the intervention received by study participants? NI  Comment. assessments were described as "blinded", but the PDSS-SR is a self-reported questionnaire.  4.4 Could assessment of the outcome have been influenced by knowledge of intervention received? PY  Comment. Participants were aware of their treatment allocation and judged the treatment outcome by means of a self-reported questionnaire. Participants allocated to the intervention group may have been more prone to judge favourably the treatment just knowing they had not been allocated to the WL condition.  4.5 Is it likely that assessment of the outcome was influenced by knowledge of intervention received? PN |
| Domain 5: Risk of bias in selection of the reported result | Some concerns | 5.1 Were the data that produced this result analysed in accordance with a pre-specified analysis plan that was finalized before unblinded outcome data were available for analysis? PN  Comment. No protocol available.  5.2 Is the numerical result being assessed likely to have been selected, on the basis of the results, from multiple eligible outcome measurements within the outcome domain? N  5.3 Is the numerical result being assessed likely to have been selected, on the basis of the results, from multiple eligible analyses of the data? PN |
| OVERALL RISK OF BIAS | High risk |  |

### de Beurs 1995

#### Risk of bias table

| **Bias** | **Authors' judgement** | **Support for judgement** |
| --- | --- | --- |
| Domain 1: Risk of bias arising from the randomization process | Some concerns | 1.1 Was the allocation sequence random? PY  1.2 Was the allocation sequence concealed until participants were enrolled and assigned to interventions? NI  Comment. Quote:" Patients were randomly assigned". No further details.  1.3 Did baseline differences between intervention groups suggest a problem with the randomization process? PN  Comment. Quote:"there were no statitstically significant differences in demographics among the subgroups". |
| Domain 2a: Risk of bias due to deviations from the intended interventions (effect of assignment to intervention) | High risk | 2.6 Was an appropriate analysis used to estimate the effect of assignment to intervention? PN  Comment. Only completers were analyzed (per-protocol approach). Authors mention an ITT double-check, but data are not shown.  2.7 Was there potential for a substantial impact (on the result) of the failure to analyse participants in the group to which they were randomized? PY  Comment. 20/96 participants dropped out from the study. |
| Domain 2b: Risk of bias due to deviations from the intended interventions (effect of adhering to intervention) | High risk | 2.3 Were important non-protocol interventions balanced across intervention groups? NI  Comment. no information provided on the balancing of off-protocol medications or other off-protocol interventions across comparison groups.  2.4. Were there failures in implementing the intervention that could have affected the outcome? PN  2.5. Was there non-adherence to the assigned intervention regimen that could have affected participants’ outcomes? PN  2.6. Was an appropriate analysis used to estimate the effect of adhering to the intervention? PN  Comment. the analysis was carried out on a per protocol basis. Authors state that they double-checked results running a ITT as well, but data are not shown. |
| Domain 3: Missing outcome data | Some concerns | 3.1 Were data for this outcome available for all, or nearly all, participants randomized? PN  Comment. Attrition rate higher than 20%.  3.2 Is there evidence that the result was not biased by missing outcome data? NI  3.3 Could missingness in the outcome depend on its true value? NI  3.4 Is it likely that missingness in the outcome depended on its true value? NI |
| Domain 4: Risk of bias in measurement of the outcome | Low risk | 4.1 Was the method of measuring the outcome inappropriate? PN  4.2 Could measurement or ascertainment of the outcome have differed between intervention groups? N  4.3 Were outcome assessors aware of the intervention received by study participants? NI  Comment. Only self-reported measures were analyzed, but patients randomized in the fluvoxamine or placebo were blinded to treatment condition.  4.4 Could assessment of the outcome have been influenced by knowledge of intervention received? PN  Comment. Participants were aware of their treatment allocation and judged the treatment outcome by means of a self-reported questionnaire. Nonetheless, people from all groups knew or at least cannot exclude (the pharmacological arms were double-blinded) they were receiving an active intervention, thus participants probably had high and equivalent expectancies in both treatment groups. |
| Domain 5: Risk of bias in selection of the reported result | Some concerns | 5.1 Were the data that produced this result analysed in accordance with a pre-specified analysis plan that was finalized before unblinded outcome data were available for analysis? PN  Comment. No protocol available.  5.2 Is the numerical result being assessed likely to have been selected, on the basis of the results, from multiple eligible outcome measurements within the outcome domain? N  5.3 Is the numerical result being assessed likely to have been selected, on the basis of the results, from multiple eligible analyses of the data? PN |
| OVERALL RISK OF BIAS | High risk |  |

### de Ruiter 1989

#### Risk of bias table

| **Bias** | **Authors' judgement** | **Support for judgement** |
| --- | --- | --- |
| Domain 1: Risk of bias arising from the randomization process | High risk | 1.1 Was the allocation sequence random? PN  1.2 Was the allocation sequence concealed until participants were enrolled and assigned to interventions? NI  Comment. The trial is described as randomized but no other information on the randomization procedure nor on the allocation concealment are available. The authors stated that they "tried to achieve equal sex distributions across treatments"; in order to achieve that, some sort of manipulation of the randomization process is likely to have occurred.  1.3 Did baseline differences between intervention groups suggest a problem with the randomization process? PN  Comment. Quote:"The analyses revealed no differences between the three groups with regard to sex and duration of disorder" |
| Domain 2a: Risk of bias due to deviations from the intended interventions (effect of assignment to intervention) | High risk | 2.6 Was an appropriate analysis used to estimate the effect of assignment to intervention? N  Comment. Only completers were analyzed (per-protocol approach).  2.7 Was there potential for a substantial impact (on the result) of the failure to analyse participants in the group to which they were randomized? PY  Comment. The overall attrition rate was around 18% but drop out were unbalanced across comparison groups: 4 (24%) for BRCR, 4 (24%) for EXP and 1 (6%) for BRCR + EXP. |
| Domain 2b: Risk of bias due to deviations from the intended interventions (effect of adhering to intervention) | Low risk | 2.3 Were important non-protocol interventions balanced across intervention groups?  2.4. Were there failures in implementing the intervention that could have affected the outcome?  2.5. Was there non-adherence to the assigned intervention regimen that could have affected participants’ outcomes?  2.6. Was an appropriate analysis used to estimate the effect of adhering to the intervention?  Comment. |
| Domain 3: Missing outcome data | Low risk | 3.1 Were data for this outcome available for all, or nearly all, participants randomized? Y  Comment. Quote:"A dichotomous variable (yes vs no medication) was created, to compare medication usage across treatment groups. Frequency of use of psychotropic medication across groups was not significantly different". |
| Domain 4: Risk of bias in measurement of the outcome | Low risk | 4.1 Was the method of measuring the outcome inappropriate? PN  4.2 Could measurement or ascertainment of the outcome have differed between intervention groups? N  4.3 Were outcome assessors aware of the intervention received by study participants? NI  Comment. Quote:"Fear Surrey Schedule-III (FSS-IZZ). The FSS-III is a 76-item self report inventory of phobic anxiety".  4.4 Could assessment of the outcome have been influenced by knowledge of intervention received? PN  Comment. Participants were aware of their treatment allocation and judged the treatment outcome by means of a self-reported questionnaire. Nonetheless, people from both groups knew they were both receiving an active intervention, thus participants probably had high and equivalent expectancies in both treatment groups. |
| Domain 5: Risk of bias in selection of the reported result | Some concerns | 5.1 Were the data that produced this result analysed in accordance with a pre-specified analysis plan that was finalized before unblinded outcome data were available for analysis? PN  Comment. No protocol available.  5.2 Is the numerical result being assessed likely to have been selected, on the basis of the results, from multiple eligible outcome measurements within the outcome domain? N  5.3 Is the numerical result being assessed likely to have been selected, on the basis of the results, from multiple eligible analyses of the data? PN |
| OVERALL RISK OF BIAS | High risk |  |

### Erickson 2007

#### Risk of bias table

| **Bias** | **Authors' judgement** | **Support for judgement** |
| --- | --- | --- |
| Domain 1: Risk of bias arising from the randomization process | Some concerns | 1.1 Was the allocation sequence random? PY  1.2 Was the allocation sequence concealed until participants were enrolled and assigned to interventions? NI  Comment. Quote:"patients were randomly assigned". No further information.  1.3 Did baseline differences between intervention groups suggest a problem with the randomization process? PN  Comment. no significant differences at baseline. |
| Domain 2a: Risk of bias due to deviations from the intended interventions (effect of assignment to intervention) | High risk | 2.6 Was an appropriate analysis used to estimate the effect of assignment to intervention? N  Comment. in the panic disorder subsample only data for patients who completed seven or more CBT group sessions are shown.  2.7 Was there potential for a substantial impact (on the result) of the failure to analyse participants in the group to which they were randomized? NI |
| Domain 2b: Risk of bias due to deviations from the intended interventions (effect of adhering to intervention) | Low risk | 2.3 Were important non-protocol interventions balanced across intervention groups? PY  Comment. Quote:"Patients were instructed not to taken any anxiety-reducing or antidepressant drugs during the experimental trial".  2.4. Were there failures in implementing the intervention that could have affected the outcome? PN  2.5. Was there non-adherence to the assigned intervention regimen that could have affected participants’ outcomes? PN |
| Domain 3: Missing outcome data | Some concerns | 3.1 Were data for this outcome available for all, or nearly all, participants randomized? NI  Comment. No information provided for the PD subsample.  3.2 Is there evidence that the result was not biased by missing outcome data? NI  3.3 Could missingness in the outcome depend on its true value? NI  3.4 Is it likely that missingness in the outcome depended on its true value? NI |
| Domain 4: Risk of bias in measurement of the outcome | Some concerns | 4.1 Was the method of measuring the outcome inappropriate? PN  4.2 Could measurement or ascertainment of the outcome have differed between intervention groups? N  4.3 Were outcome assessors aware of the intervention received by study participants? Y  Comment. only self-reported measures.  4.4 Could assessment of the outcome have been influenced by knowledge of intervention received? PY  Comment. Participants were aware of their treatment allocation and judged the treatment outcome by means of a self-reported questionnaire. Participants allocated to the intervention group may have been more prone to judge favourably the treatment just knowing they had not been allocated to the WL condition.  4.5 Is it likely that assessment of the outcome was influenced by knowledge of intervention received? PN |
| Domain 5: Risk of bias in selection of the reported result | Some concerns | 5.1 Were the data that produced this result analysed in accordance with a pre-specified analysis plan that was finalized before unblinded outcome data were available for analysis? PN  Comment. No protocol available.  5.2 Is the numerical result being assessed likely to have been selected, on the basis of the results, from multiple eligible outcome measurements within the outcome domain? N  5.3 Is the numerical result being assessed likely to have been selected, on the basis of the results, from multiple eligible analyses of the data? PN |
| OVERALL RISK OF BIAS | High risk |  |

### Fogliati 2016

#### Risk of bias table

| **Bias** | **Authors' judgement** | **Support for judgement** |
| --- | --- | --- |
| Domain 1: Risk of bias arising from the randomization process | Some concerns | 1.1 Was the allocation sequence random? PY  1.2 Was the allocation sequence concealed until participants were enrolled and assigned to interventions? NI  Comment. Quote:"participants were randomized". No further information.  1.3 Did baseline differences between intervention groups suggest a problem with the randomization process? PN  Comment. Quote:"There were no differences between the TD-CBT and DS-CBT or the CG-CBT and SG-CBT groups on the demographic variables (ps > .01) with the exception that a slightly higher proportion of participants in SGCBT group reported a history of mental health treatment compared to participants in the CG-CBT group." |
| Domain 2a: Risk of bias due to deviations from the intended interventions (effect of assignment to intervention) | Low risk | 2.6 Was an appropriate analysis used to estimate the effect of assignment to intervention? Y  Comment. Quote:"Consistent with the principles of intention-to-treat analyses, separate GEE models utilising random intercepts were employed to impute missing data." |
| Domain 2b: Risk of bias due to deviations from the intended interventions (effect of adhering to intervention) | Low risk | 2.3 Were important non-protocol interventions balanced across intervention groups? PY  Comment. Roughly the same percentage of participants were taking medications in each comparin group at study baseline.  2.4. Were there failures in implementing the intervention that could have affected the outcome? PN  2.5. Was there non-adherence to the assigned intervention regimen that could have affected participants’ outcomes? PN |
| Domain 3: Missing outcome data | Low risk | 3.1 Were data for this outcome available for all, or nearly all, participants randomized? Y  Comment. 68/73 (93%) in the TD group, 64/72 (88,8%) were analyzed in the TD group. |
| Domain 4: Risk of bias in measurement of the outcome | Low risk | 4.1 Was the method of measuring the outcome inappropriate? PN  4.2 Could measurement or ascertainment of the outcome have differed between intervention groups? N  4.3 Were outcome assessors aware of the intervention received by study participants? NI  Comment. Only self-reported measures.  4.4 Could assessment of the outcome have been influenced by knowledge of intervention received? PN  Comment. Participants were aware of their treatment allocation and judged the treatment outcome by means of a self-reported questionnaire. Nonetheless, people from both groups knew they were both receiving an active intervention, thus participants probably had high and equivalent expectancies in both treatment groups. |
| Domain 5: Risk of bias in selection of the reported result | Low risk | 5.1 Were the data that produced this result analysed in accordance with a pre-specified analysis plan that was finalized before unblinded outcome data were available for analysis? Y  Comment. Quote: "the trial was registered on the Australian and New Zealand Clinical Trials Registry (ANZCTR) as ACTRN12612000431820".  5.2 Is the numerical result being assessed likely to have been selected, on the basis of the results, from multiple eligible outcome measurements within the outcome domain? N  5.3 Is the numerical result being assessed likely to have been selected, on the basis of the results, from multiple eligible analyses of the data? PN |
| OVERALL RISK OF BIAS | Low risk |  |

### Gensichen 2020

#### Risk of bias table

| **Bias** | **Authors' judgement** | **Support for judgement** |
| --- | --- | --- |
| Domain 1: Risk of bias arising from the randomization process | Some concerns | 1.1 Was the allocation sequence random? PY  1.2 Was the allocation sequence concealed until participants were enrolled and assigned to interventions? NI  Comment. Quote:"cluster randomization of the GP practices was performed". No further information.  1.3 Did baseline differences between intervention groups suggest a problem with the randomization process? PN  Comment. Quote: "The two treatment groups were similar with regard to practice and patient characteristics (Tables 1a and b)". |
| Domain 2a: Risk of bias due to deviations from the intended interventions (effect of assignment to intervention) | Low risk | 2.6 Was an appropriate analysis used to estimate the effect of assignment to intervention? Y  Comment. Quote:"Intention-to-treat (ITT) analyses were performed". |
| Domain 2b: Risk of bias due to deviations from the intended interventions (effect of adhering to intervention) | Low risk | 2.3 Were important non-protocol interventions balanced across intervention groups? PY  Comment. Quote:"Intake of psychotropic medications was reported by 55% of patients; there was no difference in this respect at the 12-month follow-up between the two groups."  2.4. Were there failures in implementing the intervention that could have affected the outcome? PN  2.5. Was there non-adherence to the assigned intervention regimen that could have affected participants’ outcomes? PN |
| Domain 3: Missing outcome data | Low risk | 3.1 Were data for this outcome available for all, or nearly all, participants randomized? PY  Comment. Quote:" The 6-month follow-up was responded to by 175/230 (76%) patients of the intervention group and by 163/189 (86%) patients of the control group". |
| Domain 4: Risk of bias in measurement of the outcome | Some concerns | 4.1 Was the method of measuring the outcome inappropriate? PN  4.2 Could measurement or ascertainment of the outcome have differed between intervention groups? N  4.3 Were outcome assessors aware of the intervention received by study participants? Y  Comment. GPs where not blind to treatment allocation. The mobility index is a self-rating instrument.  4.4 Could assessment of the outcome have been influenced by knowledge of intervention received? PY  Comment. Participants were aware of their treatment allocation and judged the treatment outcome by means of a self-reported questionnaire. Participants allocated to the intervention group may have been more prone to judge favourably the treatment just knowing they had not been allocated to the TAU condition.  4.5 Is it likely that assessment of the outcome was influenced by knowledge of intervention received? PN |
| Domain 5: Risk of bias in selection of the reported result | Low risk | 5.1 Were the data that produced this result analysed in accordance with a pre-specified analysis plan that was finalized before unblinded outcome data were available for analysis? Y  Comment. Current Controlled Trials (www.isrctn.com/ISRCTN64669297).  5.2 Is the numerical result being assessed likely to have been selected, on the basis of the results, from multiple eligible outcome measurements within the outcome domain? N  5.3 Is the numerical result being assessed likely to have been selected, on the basis of the results, from multiple eligible analyses of the data? PN |
| OVERALL RISK OF BIAS | Low risk |  |

### Gloster 2011

#### Risk of bias table

| **Bias** | **Authors' judgement** | **Support for judgement** |
| --- | --- | --- |
| Domain 1: Risk of bias arising from the randomization process | Some concerns | 1.1 Was the allocation sequence random? Y  1.2 Was the allocation sequence concealed until participants were enrolled and assigned to interventions? Y  Comment. Quote:"The randomization list was generated at the clinical coordination center (Dresden) by personnel not associated with patient care. The study centers were blind to the assignment of subsequent cases and were informed of treatment status only after a fax documenting the included patient was sent to the clinical coordination center. More numbers for each center were drawn than necessary so that treatment condition of final patients in each study center remained unpredictable, thereby ensuring blinding of the randomization throughout the study."  1.3 Did baseline differences between intervention groups suggest a problem with the randomization process? PY  Comment. Quote:"The groups differed on some variables at BL despite randomization. Patients in the "T- condition" were more frequently diagnosed with depression, were more likely to report at least one panic attack in the previous week and had a higher global severity". |
| Domain 2a: Risk of bias due to deviations from the intended interventions (effect of assignment to intervention) | Low risk | 2.6 Was an appropriate analysis used to estimate the effect of assignment to intervention? Y  Comment. Quote:"For therapy effects, intent-to-treat analyses as well as completer analyses (based on 306 completers at post) were conducted." Table 2 shows data of primary outcomes measures using LOCF. |
| Domain 2b: Risk of bias due to deviations from the intended interventions (effect of adhering to intervention) | Some concerns | 2.3 Were important non-protocol interventions balanced across intervention groups? Y  Comment. Quote:" Patients had to agree to discontinue all psychopharmaco- logical medication and were not allowed to have any concomitant psychotherapy. Patients on psychopharmacological medication un- derwent a washout period prior to baseline."  2.4. Were there failures in implementing the intervention that could have affected the outcome? PN  2.5. Was there non-adherence to the assigned intervention regimen that could have affected participants’ outcomes? PN |
| Domain 3: Missing outcome data | Low risk | 3.1 Were data for this outcome available for all, or nearly all, participants randomized? PY  Comment. Across active treatment groups, 59 (19.6%) dropped out of treatment prior to post and an additional 26 (8.6%) dropped out between post and FU-6. Differences in attrition between T- and T+ were not significant". |
| Domain 4: Risk of bias in measurement of the outcome | Low risk | 4.1 Was the method of measuring the outcome inappropriate? N  4.2 Could measurement or ascertainment of the outcome have differed between intervention groups? N  4.3 Were outcome assessors aware of the intervention received by study participants? N  Comment. Quote:"All raters were blind to treatment condition". |
| Domain 5: Risk of bias in selection of the reported result | Some concerns | 5.1 Were the data that produced this result analysed in accordance with a pre-specified analysis plan that was finalized before unblinded outcome data were available for analysis? PN  Comment. No protocol available.  5.2 Is the numerical result being assessed likely to have been selected, on the basis of the results, from multiple eligible outcome measurements within the outcome domain? N  5.3 Is the numerical result being assessed likely to have been selected, on the basis of the results, from multiple eligible analyses of the data? PN |
| OVERALL RISK OF BIAS | Low risk |  |

### Gould 1993

#### Risk of bias table

| **Bias** | **Authors' judgement** | **Support for judgement** |
| --- | --- | --- |
| Domain 1: Risk of bias arising from the randomization process | Some concerns | 1.1 Was the allocation sequence random? PY  1.2 Was the allocation sequence concealed until participants were enrolled and assigned to interventions? NI  Comment. Quote:"participants were randomly assigned". No further information  1.3 Did baseline differences between intervention groups suggest a problem with the randomization process? PN  Comment. Quote:"Treatment groups did not significantly differ from each other for any of these measures at pretreament." |
| Domain 2a: Risk of bias due to deviations from the intended interventions (effect of assignment to intervention) | High risk | 2.6 Was an appropriate analysis used to estimate the effect of assignment to intervention? PN  Comment. There are no information on the type of analysis that was carried out, but it is likely that it has been conducted on a "per-protocol basis".  2.7 Was there potential for a substantial impact (on the result) of the failure to analyse participants in the group to which they were randomized? NI |
| Domain 2b: Risk of bias due to deviations from the intended interventions (effect of adhering to intervention) | High risk | 2.3 Were important non-protocol interventions balanced across intervention groups? NI  Comment. Though it is stated that:"Subjects taking medication for anxiety or depression were allowed to participate if they had been stabilized on the medication for at least four weeks (n = 6)" there are no further insights on the balancing of the drug intake across comparison groups.  2.4. Were there failures in implementing the intervention that could have affected the outcome? PN  2.5. Was there non-adherence to the assigned intervention regimen that could have affected participants’ outcomes? PN  2.6 Was an appropriate analysis used to estimate the effect of adhering to the intervention? PN  Comment. No info provided but likely the analysis was on a per-protocol basis. |
| Domain 3: Missing outcome data | Low risk | 3.1 Were data for this outcome available for all, or nearly all, participants randomized? Y  Comment. Quote:"Only 2 subjects dropped out: 1 from the WL group and 1 from the BT group." |
| Domain 4: Risk of bias in measurement of the outcome | Some concerns | 4.1 Was the method of measuring the outcome inappropriate? PN  4.2 Could measurement or ascertainment of the outcome have differed between intervention groups? N  4.3 Were outcome assessors aware of the intervention received by study participants? NI  Comment. No information provided on the masking of the raters. The ASI is a self-reported instrument.  4.4 Could assessment of the outcome have been influenced by knowledge of intervention received? PY  Comment. Participants were aware of their treatment allocation and judged the treatment outcome by means of a self-reported questionnaire. Participants allocated to the intervention group may have been more prone to judge favourably the treatment just knowing they had not been allocated to the WL condition.  4.5 Is it likely that assessment of the outcome was influenced by knowledge of intervention received? PN |
| Domain 5: Risk of bias in selection of the reported result | Some concerns | 5.1 Were the data that produced this result analysed in accordance with a pre-specified analysis plan that was finalized before unblinded outcome data were available for analysis? PN  Comment. No protocol available.  5.2 Is the numerical result being assessed likely to have been selected, on the basis of the results, from multiple eligible outcome measurements within the outcome domain? N  5.3 Is the numerical result being assessed likely to have been selected, on the basis of the results, from multiple eligible analyses of the data? PN |
| OVERALL RISK OF BIAS | High risk |  |

### Hazen 1996

#### Risk of bias table

| **Bias** | **Authors' judgement** | **Support for judgement** |
| --- | --- | --- |
| Domain 1: Risk of bias arising from the randomization process | Some concerns | 1.1 Was the allocation sequence random? PY  1.2 Was the allocation sequence concealed until participants were enrolled and assigned to interventions? NI  Comment. Quote:"subjects were randomly assigned".  1.3 Did baseline differences between intervention groups suggest a problem with the randomization process? NI  Comment. No information provided. |
| Domain 2a: Risk of bias due to deviations from the intended interventions (effect of assignment to intervention) | High risk | 2.6 Was an appropriate analysis used to estimate the effect of assignment to intervention? PN  Comment. Only data on completers are reported.  2.7 Was there potential for a substantial impact (on the result) of the failure to analyse participants in the group to which they were randomized? NI  Comment. No information on the balancing of dropouts. |
| Domain 2b: Risk of bias due to deviations from the intended interventions (effect of adhering to intervention) | High risk | 2.3 Were important non-protocol interventions balanced across intervention groups? NI  Comment. Low doses of benzodiazepines (equivalent of 20 mg of diazepam or less) or stable doses of antidepressants (i.e., prescribed for at least 6 months and stable dose for at least 3 months prior to entry) were allowed. No information on the balancing of antidepressant across grups. BZP were balanced across comparison groups.  2.4. Were there failures in implementing the intervention that could have affected the outcome? PN  2.5. Was there non-adherence to the assigned intervention regimen that could have affected participants’ outcomes? PN  2.6 Was an appropriate analysis used to estimate the effect of adhering to the intervention? PN  Comment. Only completers were analyzed. |
| Domain 3: Missing outcome data | Low risk | 3.1 Were data for this outcome available for all, or nearly all, participants randomized? PY  Comment. Quote:”Of the 117 subjects enrolled in the evaluation study, 106 completed the Anxiety Sensitivity Index at pre- and posttreatment. These subjects comprised the sample for the present study.“ No further detail about these 11 dropouts is reported. |
| Domain 4: Risk of bias in measurement of the outcome | Some concerns | 4.1 Was the method of measuring the outcome inappropriate? PN  4.2 Could measurement or ascertainment of the outcome have differed between intervention groups? N  4.3 Were outcome assessors aware of the intervention received by study participants? NI  Comment. Quote:"Over the course of the study, the assessor remained blind to subjects’ treatment group status in order to ensure that unbiased ratings were made." The SPRAS is a self-reportd instrument.  4.4 Could assessment of the outcome have been influenced by knowledge of intervention received? PY  Comment. Participants were aware of their treatment allocation and judged the treatment outcome by means of a self-reported questionnaire. Participants allocated to the intervention group may have been more prone to judge favourably the treatment just knowing they had not been allocated to the WL condition.  4.5 Is it likely that assessment of the outcome was influenced by knowledge of intervention received? PN |
| Domain 5: Risk of bias in selection of the reported result | Some concerns | 5.1 Were the data that produced this result analysed in accordance with a pre-specified analysis plan that was finalized before unblinded outcome data were available for analysis? PN  Comment. No protocol available.  5.2 Is the numerical result being assessed likely to have been selected, on the basis of the results, from multiple eligible outcome measurements within the outcome domain? N  5.3 Is the numerical result being assessed likely to have been selected, on the basis of the results, from multiple eligible analyses of the data? PN |
| OVERALL RISK OF BIAS | High risk |  |

### Hecker 1996

#### Risk of bias table

| **Bias** | **Authors' judgement** | **Support for judgement** |
| --- | --- | --- |
| Domain 1: Risk of bias arising from the randomization process | Some concerns | 1.1 Was the allocation sequence random? PY  1.2 Was the allocation sequence concealed until participants were enrolled and assigned to interventions? NI  Comment. Quote:"Sixteen individuals with panic disorder were randomly assigned to the treatment conditions".  1.3 Did baseline differences between intervention groups suggest a problem with the randomization process? NI  Comment. No information provided. |
| Domain 2a: Risk of bias due to deviations from the intended interventions (effect of assignment to intervention) | High risk | 2.6 Was an appropriate analysis used to estimate the effect of assignment to intervention? PN  Comment. Only data on completers are reported.  2.7 Was there potential for a substantial impact (on the result) of the failure to analyse participants in the group to which they were randomized? PY  Comment. 3/8 drop-out in the self-directed group and 0/8 in the therapist-directed one. Drop-outs were unbalanced across the two groups. |
| Domain 2b: Risk of bias due to deviations from the intended interventions (effect of adhering to intervention) | Low risk | 2.3 Were important non-protocol interventions balanced across intervention groups? PY  Comment. Quote:"Participants taking medications showed significant changes from pre- to posttreatment on these measures, but their scores were no different than those of participants who were not using medication at posttreatment".  2.4. Were there failures in implementing the intervention that could have affected the outcome? PN  2.5. Was there non-adherence to the assigned intervention regimen that could have affected participants’ outcomes? PN |
| Domain 3: Missing outcome data | Some concerns | 3.1 Were data for this outcome available for all, or nearly all, participants randomized? PN  Comment. In one group 3/8 (almost 40%) of participants were lost to follow-up.  3.2 Is there evidence that the result was not biased by missing outcome data? NI  3.3 Could missingness in the outcome depend on its true value? NI  3.4 Is it likely that missingness in the outcome depended on its true value? NI |
| Domain 4: Risk of bias in measurement of the outcome | Low risk | 4.1 Was the method of measuring the outcome inappropriate? PN  4.2 Could measurement or ascertainment of the outcome have differed between intervention groups? N  4.3 Were outcome assessors aware of the intervention received by study participants? NI  Comment. Only self-reported measures.  4.4 Could assessment of the outcome have been influenced by knowledge of intervention received? PN  Comment. Participants were aware of their treatment allocation and judged the treatment outcome by means of a self-reported questionnaire. Nonetheless, people from both groups knew they were both receiving an active intervention, thus participants probably had high and equivalent expectancies in both treatment groups. |
| Domain 5: Risk of bias in selection of the reported result | Some concerns | 5.1 Were the data that produced this result analysed in accordance with a pre-specified analysis plan that was finalized before unblinded outcome data were available for analysis? PN  Comment. No protocol available.  5.2 Is the numerical result being assessed likely to have been selected, on the basis of the results, from multiple eligible outcome measurements within the outcome domain? N  5.3 Is the numerical result being assessed likely to have been selected, on the basis of the results, from multiple eligible analyses of the data? PN |
| OVERALL RISK OF BIAS | High risk |  |

### Hendriks 2010

#### Risk of bias table

| **Bias** | **Authors' judgement** | **Support for judgement** |
| --- | --- | --- |
| Domain 1: Risk of bias arising from the randomization process | Low risk | 1.1 Was the allocation sequence random? Y  1.2 Was the allocation sequence concealed until participants were enrolled and assigned to interventions? Y  Comment. Quote:"To this end, a sealed envelop was randomly selected from an initial total of 75 envelopes containing the treatment assignments, with 30 being labelled as "CBT", 30 as "paroxetine" and 15 as "waiting list". The randomization schedule was based on the assumption that the waiting-list condition would show no effects.  1.3 Did baseline differences between intervention groups suggest a problem with the randomization process? PN  Comment. Quote:"There were no significant between-group differ- ences for patient demographics or any of the baseline outcome measures, except for psychiatric co-morbidity (higher in the paroxetine condition; Table 1)". |
| Domain 2a: Risk of bias due to deviations from the intended interventions (effect of assignment to intervention) | Low risk | 2.6 Was an appropriate analysis used to estimate the effect of assignment to intervention? Y  Comment. Quote:"Outcome scores were analysed using a mixed- model procedure, which allows all available data for all subjects to be entered into the analyses, preventing the loss of subjects for whom data were incomplete and thus precluding ad-hoc (e.g. "Last observation carried forward") solutions. |
| Domain 2b: Risk of bias due to deviations from the intended interventions (effect of adhering to intervention) | Some concerns | 2.3 Were important non-protocol interventions balanced across intervention groups? NI  Comment. no information provided on the balancing of off-protocol medications or other off-protocol interventions across comparison groups.  2.4. Were there failures in implementing the intervention that could have affected the outcome? PN  2.5. Was there non-adherence to the assigned intervention regimen that could have affected participants’ outcomes? PN  2.6 Was an appropriate analysis used to estimate the effect of adhering to the intervention? Y  Comment. The analysis was carried out on a intention-to-treat basis. |
| Domain 3: Missing outcome data | Low risk | 3.1 Were data for this outcome available for all, or nearly all, participants randomized? Y  Comment. Quote:"With one patient (1 ⁄ 20, 5%) in the CBT and three (3 ⁄ 14, 17.6%) in the paroxetine condition dropping out, attrition rates were low." |
| Domain 4: Risk of bias in measurement of the outcome | Some concerns | 4.1 Was the method of measuring the outcome inappropriate? PN  4.2 Could measurement or ascertainment of the outcome have differed between intervention groups? N  4.3 Were outcome assessors aware of the intervention received by study participants? NI  Comment. Quote:" All assessments were administered by trained, independent psychologists who were blind to the study and treatments delivered." Nonetheless, the ACQ is a self-administred rating scale.  4.4 Could assessment of the outcome have been influenced by knowledge of intervention received? PY  Comment. Participants were aware of their treatment allocation and judged the treatment outcome by means of a self-reported questionnaire. Participants allocated to the intervention group may have been more prone to judge favourably the treatment just knowing they had not been allocated to the WL condition.  4.5 Is it likely that assessment of the outcome was influenced by knowledge of intervention received? PN |
| Domain 5: Risk of bias in selection of the reported result | Some concerns | 5.1 Were the data that produced this result analysed in accordance with a pre-specified analysis plan that was finalized before unblinded outcome data were available for analysis? PN  Comment. No protocol available.  5.2 Is the numerical result being assessed likely to have been selected, on the basis of the results, from multiple eligible outcome measurements within the outcome domain? N  5.3 Is the numerical result being assessed likely to have been selected, on the basis of the results, from multiple eligible analyses of the data? PN |
| OVERALL RISK OF BIAS | Some concerns | . |

### Kenardy 2003

#### Risk of bias table

| **Bias** | **Authors' judgement** | **Support for judgement** |
| --- | --- | --- |
| Domain 1: Risk of bias arising from the randomization process | High risk | 1.1 Was the allocation sequence random? PY  1.2 Was the allocation sequence concealed until participants were enrolled and assigned to interventions? NI  Comment. Quote:"participants were randomly assigned". No further information.  1.3 Did baseline differences between intervention groups suggest a problem with the randomization process? PY  Comment. Quote:"Patients in Scotland had a significantly greater mean duration current episode compared with the Australian patients, there was also a significant difference between sites on education level. Significantly more of the Australian patients (46.4%) were prescribed concurrent benzodiazepines compared with Scottish patients." |
| Domain 2a: Risk of bias due to deviations from the intended interventions (effect of assignment to intervention) | High risk | 2.6 Was an appropriate analysis used to estimate the effect of assignment to intervention? PN  Comment. Althoug there is no information available in the manuscript, it is likely that the analysis has been carried out on a per protocol basis.  2.7 Was there potential for a substantial impact (on the result) of the failure to analyse participants in the group to which they were randomized? NI  Comment. There are only generic information on drop-outs and it is not possible to tell if the completers were balanced across the comparison groups. |
| Domain 2b: Risk of bias due to deviations from the intended interventions (effect of adhering to intervention) | Low risk | 2.3 Were important non-protocol interventions balanced across intervention groups? Y  Comment. Quote:"Concurrent use of anxiolytic or antidepressant medication did not predict improvement on the composite pre- to posttreatment or maintenance post- treatment to follow-up. When the primary analysis of the composite score was repeated including only those patients who were medication free, it yielded the same pattern of results as for the whole sample".  2.4. Were there failures in implementing the intervention that could have affected the outcome? PN  2.5. Was there non-adherence to the assigned intervention regimen that could have affected participants’ outcomes? PN |
| Domain 3: Missing outcome data | Some concerns | 3.1 Were data for this outcome available for all, or nearly all, participants randomized? NI  Comment. Information are not detailed enough.  3.2 Is there evidence that the result was not biased by missing outcome data? NI  3.3 Could missingness in the outcome depend on its true value? NI  3.4 Is it likely that missingness in the outcome depended on its true value? NI |
| Domain 4: Risk of bias in measurement of the outcome | Some concerns | 4.1 Was the method of measuring the outcome inappropriate? PN  4.2 Could measurement or ascertainment of the outcome have differed between intervention groups? N  4.3 Were outcome assessors aware of the intervention received by study participants? NI  Comment. No information provided on the masking of the raters. The ACQ is a self-reported instrument.  4.4 Could assessment of the outcome have been influenced by knowledge of intervention received? PY  Comment. Participants were aware of their treatment allocation and judged the treatment outcome by means of a self-reported questionnaire. Participants allocated to the intervention group may have been more prone to judge favourably the treatment just knowing they had not been allocated to the WL condition.  4.5 Is it likely that assessment of the outcome was influenced by knowledge of intervention received? PN |
| Domain 5: Risk of bias in selection of the reported result | Some concerns | 5.1 Were the data that produced this result analysed in accordance with a pre-specified analysis plan that was finalized before unblinded outcome data were available for analysis? PN  Comment. No protocol available.  5.2 Is the numerical result being assessed likely to have been selected, on the basis of the results, from multiple eligible outcome measurements within the outcome domain? N  5.3 Is the numerical result being assessed likely to have been selected, on the basis of the results, from multiple eligible analyses of the data? PN |
| OVERALL RISK OF BIAS | High risk |  |

### Kiropoulos 2008

#### Risk of bias table

| **Bias** | **Authors' judgement** | **Support for judgement** |
| --- | --- | --- |
| Domain 1: Risk of bias arising from the randomization process | High risk | 1.1 Was the allocation sequence random? Y  1.2 Was the allocation sequence concealed until participants were enrolled and assigned to interventions? NI  Comment. Quote:"participants were randomly allocated using a random numbers table to either the PO or face-to-face CBT treatment condition."  1.3 Did baseline differences between intervention groups suggest a problem with the randomization process? PY  Comment. 55,6% of the patients randomized in the PO arm were on psychotropic medications, against 40% of those in the face-to-face arm. |
| Domain 2a: Risk of bias due to deviations from the intended interventions (effect of assignment to intervention) | Low risk | 2.6 Was an appropriate analysis used to estimate the effect of assignment to intervention? Y  Comment. Quote:"Data analysis involved intention-to-treat analyses. Participants who had missing post-assessment ques- tionnaire(s) (n = 22) or who discontinued treatment (n = 7) were treated as ‘‘intention to treat’’ (ITT)." |
| Domain 2b: Risk of bias due to deviations from the intended interventions (effect of adhering to intervention) | Some concerns | 2.3 Were important non-protocol interventions balanced across intervention groups? PN  Comment. 55,6% of the patients randomized in the PO arm were on psychotropic medications, against 40% of those in the face-to-face arm.  2.4. Were there failures in implementing the intervention that could have affected the outcome? PN  2.5. Was there non-adherence to the assigned intervention regimen that could have affected participants’ outcomes? PN  2.6 Was an appropriate analysis used to estimate the effect of adhering to the intervention? Y  Comment. The analysis was carried out on a intention-to-treat basis. |
| Domain 3: Missing outcome data | Low risk | 3.1 Were data for this outcome available for all, or nearly all, participants randomized? Y  Comment. Of the 86 who were eligible and commenced the study, seven discontinued during treatment. The overall attrition rate for the present study was therefore 8.1%. The attrition rates were 10.9% (5/46) and 5% (2/40) for the PO and face-to-face treatment conditions, respectively. |
| Domain 4: Risk of bias in measurement of the outcome | Low risk | 4.1 Was the method of measuring the outcome inappropriate? N  4.2 Could measurement or ascertainment of the outcome have differed between intervention groups? N  4.3 Were outcome assessors aware of the intervention received by study participants? N  Comment. Quote:"All assessors were blind to treatment allocation of eligible participants into the study". |
| Domain 5: Risk of bias in selection of the reported result | Some concerns | 5.1 Were the data that produced this result analysed in accordance with a pre-specified analysis plan that was finalized before unblinded outcome data were available for analysis? PN  Comment. No protocol available.  5.2 Is the numerical result being assessed likely to have been selected, on the basis of the results, from multiple eligible outcome measurements within the outcome domain? N  5.3 Is the numerical result being assessed likely to have been selected, on the basis of the results, from multiple eligible analyses of the data? PN |
| OVERALL RISK OF BIAS | Some concerns | . |

### Klein 2001

#### Risk of bias table

| **Bias** | **Authors' judgement** | **Support for judgement** |
| --- | --- | --- |
| Domain 1: Risk of bias arising from the randomization process | Some concerns | 1.1 Was the allocation sequence random? PY  1.2 Was the allocation sequence concealed until participants were enrolled and assigned to interventions? NI  Comment. Quote:"participants were randomly assigned". No further information.  1.3 Did baseline differences between intervention groups suggest a problem with the randomization process? NI  Comment. No information provided. |
| Domain 2a: Risk of bias due to deviations from the intended interventions (effect of assignment to intervention) | High risk | 2.6 Was an appropriate analysis used to estimate the effect of assignment to intervention? PN  Comment. Quote:"One participant failed to complete the study and subsequently her data were excluded". From this sentence it can be acknowledged that there were only one drop out but that the analysis didn't include her data. |
| Domain 2b: Risk of bias due to deviations from the intended interventions (effect of adhering to intervention) | High risk | 2.3 Were important non-protocol interventions balanced across intervention groups? NI  Comment. Quote:"Nine participants (41%) reported the use of anxiety medication but subsequently all reported no alteration in their dosage levels throughout the 3 weeks". No information on the balancing of these participants across comparison groups.  2.4. Were there failures in implementing the intervention that could have affected the outcome? PN  2.5. Was there non-adherence to the assigned intervention regimen that could have affected participants’ outcomes? PN  2.6 Was an appropriate analysis used to estimate the effect of adhering to the intervention? PN  Comment. No info provided. |
| Domain 3: Missing outcome data | Low risk | 3.1 Were data for this outcome available for all, or nearly all, participants randomized? PY  Comment. There are no clear information on drop-out rate, but probably only one participant discontinued the trial. |
| Domain 4: Risk of bias in measurement of the outcome | Some concerns | 4.1 Was the method of measuring the outcome inappropriate? N  4.2 Could measurement or ascertainment of the outcome have differed between intervention groups? N  4.3 Were outcome assessors aware of the intervention received by study participants? NI  Comment. No information on the masking of the assessors  4.4 Could assessment of the outcome have been influenced by knowledge of intervention received? PY  Comment. Participants were aware of their treatment allocation and judged the treatment outcome by means of a self-reported questionnaire. Participants allocated to the intervention group may have been more prone to judge favourably the treatment just knowing they had not been allocated to the WL condition.  4.5 Is it likely that assessment of the outcome was influenced by knowledge of intervention received? PY |
| Domain 5: Risk of bias in selection of the reported result | Some concerns | 5.1 Were the data that produced this result analysed in accordance with a pre-specified analysis plan that was finalized before unblinded outcome data were available for analysis? PN  Comment. No protocol available.  5.2 Is the numerical result being assessed likely to have been selected, on the basis of the results, from multiple eligible outcome measurements within the outcome domain? N  5.3 Is the numerical result being assessed likely to have been selected, on the basis of the results, from multiple eligible analyses of the data? PN |
| OVERALL RISK OF BIAS | High risk |  |

### Klein 2006

#### Risk of bias table

| **Bias** | **Authors' judgement** | **Support for judgement** |
| --- | --- | --- |
| Domain 1: Risk of bias arising from the randomization process | Some concerns | 1.1 Was the allocation sequence random? PY  1.2 Was the allocation sequence concealed until participants were enrolled and assigned to interventions? NI  Comment. Quote:"all participants were randomly assigned sequentially (i.e., ABC, ABC) using a block design". No further information.  1.3 Did baseline differences between intervention groups suggest a problem with the randomization process? NI  Comment. No information provided. |
| Domain 2a: Risk of bias due to deviations from the intended interventions (effect of assignment to intervention) | Low risk | 2.6 Was an appropriate analysis used to estimate the effect of assignment to intervention? Y  Comment. Quote:"Data analysis involved intention-to-treat analyses. That is, for those participants who discontinued their involvement during treatment (n = 9), their pre-assessment scores were carried forward and used in both the post-treatment. |
| Domain 2b: Risk of bias due to deviations from the intended interventions (effect of adhering to intervention) | Some concerns | 2.3 Were important non-protocol interventions balanced across intervention groups? NI  Comment. Quote:"Those participants who were taking medication for anxiety or depression were accepted if they had been stabilised on their medication for at least 4 weeks but continued to experience panic symptoms and met a diagnosis of PD." No information on the balancing of these participants across comparison groups.  2.4. Were there failures in implementing the intervention that could have affected the outcome? PN  2.5. Was there non-adherence to the assigned intervention regimen that could have affected participants’ outcomes? PN  2.6 Was an appropriate analysis used to estimate the effect of adhering to the intervention? Y  Comment. The analysis was carried out on a intention-to-treat basis. |
| Domain 3: Missing outcome data | Low risk | 3.1 Were data for this outcome available for all, or nearly all, participants randomized?  Comment. Quote:"Of the 55 people who commenced this study, nine discontinued during treatment. The attrition rate for the PO condition was 5% (1/19), 17% (3/18) in the MAN condition, and 28% (5/18) in the IC condition. A Fishers exact test revealed no differences in attrition rates between the three conditions". |
| Domain 4: Risk of bias in measurement of the outcome | High risk | 4.1 Was the method of measuring the outcome inappropriate? N  4.2 Could measurement or ascertainment of the outcome have differed between intervention groups? N  4.3 Were outcome assessors aware of the intervention received by study participants? PN  Comment. Quote:"The assessors were blind to which treatment the participant would be assigned to until after the pre-assessment was completed". Comment: as it is laid down in the manuscript, it seems that at the moment of the endpoint assessment raters were aware of the arm allocation of study participants. |
| Domain 5: Risk of bias in selection of the reported result | Some concerns | 5.1 Were the data that produced this result analysed in accordance with a pre-specified analysis plan that was finalized before unblinded outcome data were available for analysis? PN  Comment. No protocol available.  5.2 Is the numerical result being assessed likely to have been selected, on the basis of the results, from multiple eligible outcome measurements within the outcome domain? N  5.3 Is the numerical result being assessed likely to have been selected, on the basis of the results, from multiple eligible analyses of the data? PN |
| OVERALL RISK OF BIAS | High risk |  |

### Klein 2009

#### Risk of bias table

| **Bias** | **Authors' judgement** | **Support for judgement** |
| --- | --- | --- |
| Domain 1: Risk of bias arising from the randomization process | Some concerns | 1.1 Was the allocation sequence random? Y  1.2 Was the allocation sequence concealed until participants were enrolled and assigned to interventions? NI  Comment. Quote:"all participants were randomly assigned (via a computer-generated random numbers table without any restriction procedure) to either the FC or IC condition."  1.3 Did baseline differences between intervention groups suggest a problem with the randomization process? PN  Comment. No differences at baseline (Table 1). |
| Domain 2a: Risk of bias due to deviations from the intended interventions (effect of assignment to intervention) | Low risk | 2.6 Was an appropriate analysis used to estimate the effect of assignment to intervention? Y  Comment. Quote:"Data analysis involved intention-to-treat analyses (ITT). That is, for those participants who discontinued their involvement during treatment (n514) or had missing post-assessment questionnaire data (n515), their pre-assessment scores were carried forward and used in posttreatment." |
| Domain 2b: Risk of bias due to deviations from the intended interventions (effect of adhering to intervention) | Low risk | 2.3 Were important non-protocol interventions balanced across intervention groups? Y  Comment. Quote:"No significant differences between the two groups were found using chi-square analyses (for nominal data) or one-way ANOVA for level of education, occurrence of comorbid secondary diagnosis, medication use, and gender."  2.4. Were there failures in implementing the intervention that could have affected the outcome? PN  2.5. Was there non-adherence to the assigned intervention regimen that could have affected participants’ outcomes? PN |
| Domain 3: Missing outcome data | Some concerns | 3.1 Were data for this outcome available for all, or nearly all, participants randomized? PN  Comment. Quote:"Of the 57 people who commenced the study, 14 discontinued after random assignment to a treatment condition, for an overall attrition rate of 24.6%. The attrition rate was 21.4% (6/28) and 27.6% (8/29) for the FC and IC conditions, respectively. A Fisher’s exact test revealed no difference in attrition rates between the two treatment conditions".  3.2 Is there evidence that the result was not biased by missing outcome data? PY  Comment. Drop-outs were balanced across the two comparison groups. |
| Domain 4: Risk of bias in measurement of the outcome | Low risk | 4.1 Was the method of measuring the outcome inappropriate? N  4.2 Could measurement or ascertainment of the outcome have differed between intervention groups? N  4.3 Were outcome assessors aware of the intervention received by study participants? N  Comment. Quote:"The pre and postclinical assessments (ADIS-IV) were con- ducted over the telephone. All assessors were blind to the group membership of partici- pants, and the assessors did not treat any participant they interviewed." |
| Domain 5: Risk of bias in selection of the reported result | Some concerns | 5.1 Were the data that produced this result analysed in accordance with a pre-specified analysis plan that was finalized before unblinded outcome data were available for analysis? PN  Comment. No protocol available.  5.2 Is the numerical result being assessed likely to have been selected, on the basis of the results, from multiple eligible outcome measurements within the outcome domain? N  5.3 Is the numerical result being assessed likely to have been selected, on the basis of the results, from multiple eligible analyses of the data? PN |
| OVERALL RISK OF BIAS | Some concerns | . |

### Klosko 1990

#### Risk of bias table

| **Bias** | **Authors' judgement** | **Support for judgement** |
| --- | --- | --- |
| Domain 1: Risk of bias arising from the randomization process | Some concerns | 1.1 Was the allocation sequence random? PY  1.2 Was the allocation sequence concealed until participants were enrolled and assigned to interventions? NI  Comment. The study is described as randomized, but no further details are provided.  1.3 Did baseline differences between intervention groups suggest a problem with the randomization process? PN  Comment. Quote:"Analyses across groups of all demographic characteristics were nonsignificant." |
| Domain 2a: Risk of bias due to deviations from the intended interventions (effect of assignment to intervention) | High risk | 2.6 Was an appropriate analysis used to estimate the effect of assignment to intervention? N  Comment. Only completers were analyzed (per-protocol approach).  2.7 Was there potential for a substantial impact (on the result) of the failure to analyse participants in the group to which they were randomized? PY |
| Domain 2b: Risk of bias due to deviations from the intended interventions (effect of adhering to intervention) | High risk | 2.3 Were important non-protocol interventions balanced across intervention groups? N  Comment. Quote:"Subjects assigned to the waiting-list condition were taking significantly more medication pretreatment and were not required to withdraw from medications. Although pretreatment severity ratings between waiting-list and other groups were not significantly different, presumably these subjects would have fared worse if they had been required to withdraw from medications."  2.4. Were there failures in implementing the intervention that could have affected the outcome? PN  2.5. Was there non-adherence to the assigned intervention regimen that could have affected participants’ outcomes? PN  2.6 Was an appropriate analysis used to estimate the effect of adhering to the intervention? PN  Comment. No info provided. |
| Domain 3: Missing outcome data | High risk | 3.1 Were data for this outcome available for all, or nearly all, participants randomized? PN  Comment. Quote:"Out of 69 initial subjects, 57 subjects completed the study, and 12 subjects dropped out. A higher rate of dropout was observed in the placebo group compared with the other three groups. One subject out of 17 (5.9%) dropped from the alprazolam group, 7 out of 18 (38.9%) from the placebo group, 3 out of 18 (16.7%) from the PCT group, and 1 out of 16 (6.3%) from the waiting-list group."  3.2 Is there evidence that the result was not biased by missing outcome data? PN  Comment. Dropouts are unbalanced across comparison groups.  3.3 Could missingness in the outcome depend on its true value? NI  3.4 Is it likely that missingness in the outcome depended on its true value? NI |
| Domain 4: Risk of bias in measurement of the outcome | Low risk | 4.1 Was the method of measuring the outcome inappropriate? N  4.2 Could measurement or ascertainment of the outcome have differed between intervention groups? N  4.3 Were outcome assessors aware of the intervention received by study participants? N  Comment. Raters are described as independent as they only saw the subjects once at post-test. |
| Domain 5: Risk of bias in selection of the reported result | Some concerns | 5.1 Were the data that produced this result analysed in accordance with a pre-specified analysis plan that was finalized before unblinded outcome data were available for analysis? PN  Comment. No protocol available.  5.2 Is the numerical result being assessed likely to have been selected, on the basis of the results, from multiple eligible outcome measurements within the outcome domain? N  5.3 Is the numerical result being assessed likely to have been selected, on the basis of the results, from multiple eligible analyses of the data? PN |
| OVERALL RISK OF BIAS | High risk |  |

### Koszycki 2011

#### Risk of bias table

| **Bias** | **Authors' judgement** | **Support for judgement** |
| --- | --- | --- |
| Domain 1: Risk of bias arising from the randomization process | Low risk | 1.1 Was the allocation sequence random? Y  1.2 Was the allocation sequence concealed until participants were enrolled and assigned to interventions? Y  Comment. Quote:" Patients were randomly allocated to one of four groups by a computer- generated randomization code. [...] Investigators at each site were provided with a sealed envelope that contained the identification of the study drug being administered to the patient."  1.3 Did baseline differences between intervention groups suggest a problem with the randomization process? PN  Comment. Quote:"Differences among the treatment groups were not statistically significant". |
| Domain 2a: Risk of bias due to deviations from the intended interventions (effect of assignment to intervention) | Low risk | 2.6 Was an appropriate analysis used to estimate the effect of assignment to intervention? Y  Comment. The analysis was carried out on a intention-to-treat basis. |
| Domain 2b: Risk of bias due to deviations from the intended interventions (effect of adhering to intervention) | Some concerns | 2.3 Were important non-protocol interventions balanced across intervention groups? NI  Comment. No information provided on the balancing of off-protocol medications or other off-protocol interventions across comparison groups.  2.4. Were there failures in implementing the intervention that could have affected the outcome? PN  2.5. Was there non-adherence to the assigned intervention regimen that could have affected participants’ outcomes? PN  2.6 Was an appropriate analysis used to estimate the effect of adhering to the intervention? Y  Comment. The analysis was carried out on a intention-to-treat basis. |
| Domain 3: Missing outcome data | Some concerns | 3.1 Were data for this outcome available for all, or nearly all, participants randomized? PN  Comment. Quote:"Seventy-one patients (28.7%) discontinued acute treatment prematurely."  3.2 Is there evidence that the result was not biased by missing outcome data? PY |
| Domain 4: Risk of bias in measurement of the outcome | Low risk | 4.1 Was the method of measuring the outcome inappropriate? PN  4.2 Could measurement or ascertainment of the outcome have differed between intervention groups? N  4.3 Were outcome assessors aware of the intervention received by study participants? PN  Comment. Quote:". Outcome assessments were made by investigators who were blind to allocation of the drug and who were not told whether the patient was assigned to SCBT. Patients were instructed not to divulge their SCBT assignment to the investigators." The ACQ is a self-reported instrument.  4.4 Could assessment of the outcome have been influenced by knowledge of intervention received? PN  Comment. Participants were aware of their treatment allocation and judged the treatment outcome by means of a self-reported questionnaire. Nonetheless, people from both groups knew they were both receiving an active intervention, thus participants probably had high and equivalent expectancies in both treatment groups. |
| Domain 5: Risk of bias in selection of the reported result | Some concerns | 5.1 Were the data that produced this result analysed in accordance with a pre-specified analysis plan that was finalized before unblinded outcome data were available for analysis? PN  Comment. No protocol available.  5.2 Is the numerical result being assessed likely to have been selected, on the basis of the results, from multiple eligible outcome measurements within the outcome domain? N  5.3 Is the numerical result being assessed likely to have been selected, on the basis of the results, from multiple eligible analyses of the data? PN |
| OVERALL RISK OF BIAS | Some concerns | . |

### Lidren 1994

#### Risk of bias table

| **Bias** | **Authors' judgement** | **Support for judgement** |
| --- | --- | --- |
| Domain 1: Risk of bias arising from the randomization process | Some concerns | 1.1 Was the allocation sequence random? PY  1.2 Was the allocation sequence concealed until participants were enrolled and assigned to interventions? NI  Comment. Quote: "participants were randomized". No further information.  1.3 Did baseline differences between intervention groups suggest a problem with the randomization process? NI  Comment. No information provided. |
| Domain 2a: Risk of bias due to deviations from the intended interventions (effect of assignment to intervention) | High risk | 2.6 Was an appropriate analysis used to estimate the effect of assignment to intervention? NI  Comment. No information provided weather the analysis was IIT or per protocol.  2.7 Was there potential for a substantial impact (on the result) of the failure to analyse participants in the group to which they were randomized? NI |
| Domain 2b: Risk of bias due to deviations from the intended interventions (effect of adhering to intervention) | High risk | 2.3 Were important non-protocol interventions balanced across intervention groups? PN  Comment. 33% of the patients randomized in the intervention arms were on psychotropic medications, against 50% of those in the WL arm.  2.4. Were there failures in implementing the intervention that could have affected the outcome? PN  2.5. Was there non-adherence to the assigned intervention regimen that could have affected participants’ outcomes? PN  2.6 Was an appropriate analysis used to estimate the effect of adhering to the intervention? PN  Comment. No info provided. |
| Domain 3: Missing outcome data | Low risk | 3.1 Were data for this outcome available for all, or nearly all, participants randomized? Y  Comment. Quote:"Attrition rates were zero for all three conditions. |
| Domain 4: Risk of bias in measurement of the outcome | Some concerns | 4.1 Was the method of measuring the outcome inappropriate? PN  4.2 Could measurement or ascertainment of the outcome have differed between intervention groups? N  4.3 Were outcome assessors aware of the intervention received by study participants? NI  Comment. no information provided. The MI is a self-reported instrument.  4.4 Could assessment of the outcome have been influenced by knowledge of intervention received? PY  Comment. Participants were aware of their treatment allocation and judged the treatment outcome by means of a self-reported questionnaire. Participants allocated to the intervention group may have been more prone to judge favourably the treatment just knowing they had not been allocated to the WL condition.  4.5 Is it likely that assessment of the outcome was influenced by knowledge of intervention received? PN |
| Domain 5: Risk of bias in selection of the reported result | Some concerns | 5.1 Were the data that produced this result analysed in accordance with a pre-specified analysis plan that was finalized before unblinded outcome data were available for analysis? PN  Comment. No protocol available.  5.2 Is the numerical result being assessed likely to have been selected, on the basis of the results, from multiple eligible outcome measurements within the outcome domain? N  5.3 Is the numerical result being assessed likely to have been selected, on the basis of the results, from multiple eligible analyses of the data? PN |
| OVERALL RISK OF BIAS | High risk |  |

### Loerch 1999

#### Risk of bias table

| **Bias** | **Authors' judgement** | **Support for judgement** |
| --- | --- | --- |
| Domain 1: Risk of bias arising from the randomization process | Some concerns | 1.1 Was the allocation sequence random? PY  1.2 Was the allocation sequence concealed until participants were enrolled and assigned to interventions? NI  Comment. Quote: "participants were randomly assigned". No further information.  1.3 Did baseline differences between intervention groups suggest a problem with the randomization process? PN  Comment. Quote:"There were no significant baseline differences either with respect to all outcome variables listed above". |
| Domain 2a: Risk of bias due to deviations from the intended interventions (effect of assignment to intervention) | Low risk | 2.6 Was an appropriate analysis used to estimate the effect of assignment to intervention? Y  Comment. Quote:"planned initially to be carried out with the intention-to-treat (ITT) population, using end-points with last observation-carried-forward (LOCF)." |
| Domain 2b: Risk of bias due to deviations from the intended interventions (effect of adhering to intervention) | Low risk | 2.3 Were important non-protocol interventions balanced across intervention groups? NI  Comment. No information provided on the balancing of off-protocol medications or other off-protocol interventions across comparison groups.  2.4. Were there failures in implementing the intervention that could have affected the outcome? PN  2.5. Was there non-adherence to the assigned intervention regimen that could have affected participants’ outcomes? PN  2.6 Was an appropriate analysis used to estimate the effect of adhering to the intervention? Y  Comment. The analysis was carried out on a intention-to-treat basis. |
| Domain 3: Missing outcome data | High risk | 3.1 Were data for this outcome available for all, or nearly all, participants randomized? PN  Comment. Quote: "With 44% (7/16) the dropout rate in the moclobemide plus clinical management group was more than twice as high as in the moclobemide plus CBT group (21%, 3/14). The attrition rate was 7% in the placebo plus CBT group (1/14) and 18% in the placebo plus clinical management group (2/11)".  3.2 Is there evidence that the result was not biased by missing outcome data? PN  Comment. Quote: "The differences were considerable (exact test, P<0.13)".  3.3 Could missingness in the outcome depend on its true value? NI  3.4 Is it likely that missingness in the outcome depended on its true value? NI |
| Domain 4: Risk of bias in measurement of the outcome | Some concerns | 4.1 Was the method of measuring the outcome inappropriate? PN  4.2 Could measurement or ascertainment of the outcome have differed between intervention groups? N  4.3 Were outcome assessors aware of the intervention received by study participants? NI  Comment. Quote:"The ratings administered by the two independent psychiatrist". Nonetheless, the MI is a self-reported instrument.  4.4 Could assessment of the outcome have been influenced by knowledge of intervention received? PY  Comment. Participants were aware of their treatment allocation and judged the treatment outcome by means of a self-reported questionnaire. Participants allocated to the intervention group may have been more prone to judge favourably the treatment just knowing they had not been allocated to the WL condition.  4.5 Is it likely that assessment of the outcome was influenced by knowledge of intervention received? PN |
| Domain 5: Risk of bias in selection of the reported result | Some concerns | 5.1 Were the data that produced this result analysed in accordance with a pre-specified analysis plan that was finalized before unblinded outcome data were available for analysis? PN  Comment. No protocol available.  5.2 Is the numerical result being assessed likely to have been selected, on the basis of the results, from multiple eligible outcome measurements within the outcome domain? N  5.3 Is the numerical result being assessed likely to have been selected, on the basis of the results, from multiple eligible analyses of the data? PN |
| OVERALL RISK OF BIAS | High risk |  |

### Malbos 2011

###

| **Bias** | **Authors' judgement** | **Support for judgement** |
| --- | --- | --- |
| Domain 1: Risk of bias arising from the randomization process | Some concerns | 1.1 Was the allocation sequence random? PY  1.2 Was the allocation sequence concealed until participants were enrolled and assigned to interventions? NI  Comment. Quote: "participants were randomly assigned". No further information.  1.3 Did baseline differences between intervention groups suggest a problem with the randomization process? NI  Comment. No information provided. |
| Domain 2a: Risk of bias due to deviations from the intended interventions (effect of assignment to intervention) | High risk | 2.6 Was an appropriate analysis used to estimate the effect of assignment to intervention? PN  Comment. No information provided weather the analysis was IIT or per protocol.  2.7 Was there potential for a substantial impact (on the result) of the failure to analyse participants in the group to which they were randomized? NI |
| Domain 2b: Risk of bias due to deviations from the intended interventions (effect of adhering to intervention) | High risk | 2.3 Were important non-protocol interventions balanced across intervention groups? NI  Comment. No information provided on the balancing of off-protocol medications or other off-protocol interventions across comparison groups.  2.4. Were there failures in implementing the intervention that could have affected the outcome? PN  2.5. Was there non-adherence to the assigned intervention regimen that could have affected participants’ outcomes? PN  2.6 Was an appropriate analysis used to estimate the effect of adhering to the intervention? PN  Comment. No info provided. |
| Domain 3: Missing outcome data | Some concerns | 3.1 Were data for this outcome available for all, or nearly all, participants randomized? NI  Comment. No information on missing data.  3.2 Is there evidence that the result was not biased by missing outcome data? NI  3.3 Could missingness in the outcome depend on its true value? NI  3.4 Is it likely that missingness in the outcome depended on its true value? NI |
| Domain 4: Risk of bias in measurement of the outcome | Low risk | 4.1 Was the method of measuring the outcome inappropriate? PN  4.2 Could measurement or ascertainment of the outcome have differed between intervention groups? N  4.3 Were outcome assessors aware of the intervention received by study participants? NI  Comment. No information provided. The ASQ is a self-reported questionnaire.  4.4 Could assessment of the outcome have been influenced by knowledge of intervention received? PN  Comment. Participants were aware of their treatment allocation and judged the treatment outcome by means of a self-reported questionnaire. Nonetheless, people from both groups knew they were both receiving an active intervention, thus participants probably had high and equivalent expectancies in both treatment groups. |
| Domain 5: Risk of bias in selection of the reported result | Some concerns | 5.1 Were the data that produced this result analysed in accordance with a pre-specified analysis plan that was finalized before unblinded outcome data were available for analysis? PN  Comment. No protocol available.  5.2 Is the numerical result being assessed likely to have been selected, on the basis of the results, from multiple eligible outcome measurements within the outcome domain? N  5.3 Is the numerical result being assessed likely to have been selected, on the basis of the results, from multiple eligible analyses of the data? PN |
| OVERALL RISK OF BIAS | High risk |  |

### Marchand 2007

#### Risk of bias table

| **Bias** | **Authors' judgement** | **Support for judgement** |
| --- | --- | --- |
| Domain 1: Risk of bias arising from the randomization process | Some concerns | 1.1 Was the allocation sequence random? PN  1.2 Was the allocation sequence concealed until participants were enrolled and assigned to interventions? NI  Comment. The trial never mention the word "random" / "randomized". The authors simply state that patients were divided among groups.  1.3 Did baseline differences between intervention groups suggest a problem with the randomization process? NI  Comment. No information provided. |
| Domain 2a: Risk of bias due to deviations from the intended interventions (effect of assignment to intervention) | Some concerns | 2.6 Was an appropriate analysis used to estimate the effect of assignment to intervention? N  Comment. Only completers were analyzed (per-protocol approach).  2.7 Was there potential for a substantial impact (on the result) of the failure to analyse participants in the group to which they were randomized?  Comment. Quote:"We carried out analyses that included the 9 subjects who dropped out during the treatment, by using their scores to the pre-treatment measures as their scores at post-treatment,The results of the analyses were similar to the results obtained from the analyses carried out including only the participants who completed the treatment. |
| Domain 2b: Risk of bias due to deviations from the intended interventions (effect of adhering to intervention) | Low risk | 2.3 Were important non-protocol interventions balanced across intervention groups? PY  Comment. Quote:"Patients under medication were included in the study only if they took a light dosage and if withdrawal seemed possible. Withdrawal was done over a few weeks, depending on dosage, under psychiatric supervision". Efforts were done to reduce the possible interference of drug effect on outcome.  2.4. Were there failures in implementing the intervention that could have affected the outcome? PN  2.5. Was there non-adherence to the assigned intervention regimen that could have affected participants’ outcomes? PN |
| Domain 3: Missing outcome data | Low risk | 3.1 Were data for this outcome available for all, or nearly all, participants randomized? PY  Comment. Quote:"9 (10.46%) individuals dropped out of the study during the treatment [...]." These drop-outs came from the three treatment groups (BCBT-A: 4 individuals; BCBT-P: 2 individuals; SCB: 3 individuals), but the difference did not reach statistical significance." |
| Domain 4: Risk of bias in measurement of the outcome | Some concerns | 4.1 Was the method of measuring the outcome inappropriate? PN  4.2 Could measurement or ascertainment of the outcome have differed between intervention groups? N  4.3 Were outcome assessors aware of the intervention received by study participants? NI  Comment. Quote:"Assessments with this instrument used to establish diagnosis were completed by two independent assessors: a psychiatrist who was unaware of treatment allocation of each individual participant, and another assessor, blind to the diagnosis by the psychiatrist, who also served as therapist."  4.4 Could assessment of the outcome have been influenced by knowledge of intervention received? PY  Comment. Participants were aware of their treatment allocation and judged the treatment outcome by means of a self-reported questionnaire. Participants allocated to the intervention group may have been more prone to judge favourably the treatment just knowing they had not been allocated to the WL condition.  4.5 Is it likely that assessment of the outcome was influenced by knowledge of intervention received? PN |
| Domain 5: Risk of bias in selection of the reported result | Some concerns | 5.1 Were the data that produced this result analysed in accordance with a pre-specified analysis plan that was finalized before unblinded outcome data were available for analysis? PN  Comment. No protocol available.  5.2 Is the numerical result being assessed likely to have been selected, on the basis of the results, from multiple eligible outcome measurements within the outcome domain? N  5.3 Is the numerical result being assessed likely to have been selected, on the basis of the results, from multiple eligible analyses of the data? PN |
| OVERALL RISK OF BIAS | High risk |  |

### Marchand 2008

#### Risk of bias table

| **Bias** | **Authors' judgement** | **Support for judgement** |
| --- | --- | --- |
| Domain 1: Risk of bias arising from the randomization process | Some concerns | 1.1 Was the allocation sequence random? Y  1.2 Was the allocation sequence concealed until participants were enrolled and assigned to interventions? NI  Comment. Quote:"randomization technique used a computer random number generator to select four large random blocks every 6 months".  1.3 Did baseline differences between intervention groups suggest a problem with the randomization process? PN  Comment. Quote: ". No significant differences at an alpha level of .05 between groups were observed in terms of gender, age, marital status, education level, number of years with PDA, depression, anxiety, and the GSSS." |
| Domain 2a: Risk of bias due to deviations from the intended interventions (effect of assignment to intervention) | High risk | 2.6 Was an appropriate analysis used to estimate the effect of assignment to intervention? N  Comment. of the 154 patients that were initially randomized only 122 (completers) were analyzed.  2.7 Was there potential for a substantial impact (on the result) of the failure to analyse participants in the group to which they were randomized? PY |
| Domain 2b: Risk of bias due to deviations from the intended interventions (effect of adhering to intervention) | Low risk | 2.3 Were important non-protocol interventions balanced across intervention groups? PY  Comment. Quote:"participants had to agree to withdraw from their medication (only the study medication/placebo was permitted as well as small doses of oxazepam [15 mg] limited to 20 pills/ month). The withdrawal period was 4 weeks, and participants were stabilized for 6 weeks before beginning the psychotherapy."  2.4. Were there failures in implementing the intervention that could have affected the outcome? PN  2.5. Was there non-adherence to the assigned intervention regimen that could have affected participants’ outcomes? PN |
| Domain 3: Missing outcome data | Low risk | 3.1 Were data for this outcome available for all, or nearly all, participants randomized? PY  Comment. 17 out of 154 participants were lost to follow up at study end-point, but there are no information on the balance of drop-outs between the comparison groups. |
| Domain 4: Risk of bias in measurement of the outcome | Some concerns | 4.1 Was the method of measuring the outcome inappropriate? PN  4.2 Could measurement or ascertainment of the outcome have differed between intervention groups? N  4.3 Were outcome assessors aware of the intervention received by study participants? PY  Comment. Quote: "all study personnel were unaware of the medication assignments and research hypotheses for the duration of the study."  4.4 Could assessment of the outcome have been influenced by knowledge of intervention received? PY  Comment. Participants were aware of their treatment allocation and judged the treatment outcome by means of a self-reported questionnaire. Participants allocated to the intervention group may have been more prone to judge favourably the treatment just knowing they had not been allocated to the TAU condition.  4.5 Is it likely that assessment of the outcome was influenced by knowledge of intervention received? PN |
| Domain 5: Risk of bias in selection of the reported result | Some concerns | 5.1 Were the data that produced this result analysed in accordance with a pre-specified analysis plan that was finalized before unblinded outcome data were available for analysis? PN  Comment. No protocol available.  5.2 Is the numerical result being assessed likely to have been selected, on the basis of the results, from multiple eligible outcome measurements within the outcome domain? N  5.3 Is the numerical result being assessed likely to have been selected, on the basis of the results, from multiple eligible analyses of the data? PN |
| OVERALL RISK OF BIAS | High risk |  |

### Meulenbeek 2010

#### Risk of bias table

| **Bias** | **Authors' judgement** | **Support for judgement** |
| --- | --- | --- |
| Domain 1: Risk of bias arising from the randomization process | Some concerns | 1.1 Was the allocation sequence random? Y  1.2 Was the allocation sequence concealed until participants were enrolled and assigned to interventions? NI  Comment. Quote:"A blocked randomisation scheme was used, stratified by mental health centre, subthreshold panic disorder v. mild panic disorder, and by presence v. absence of co-occurring agoraphobia."  1.3 Did baseline differences between intervention groups suggest a problem with the randomization process? PN  Comment. Quote:"The early intervention and the control group did not differ significantly with regard to socio-demographic and clinical characteristics". |
| Domain 2a: Risk of bias due to deviations from the intended interventions (effect of assignment to intervention) | Low risk | 2.6 Was an appropriate analysis used to estimate the effect of assignment to intervention? Y  Comment. Quote:"All analyses were conducted in agreement with the intentionto-treat principle, hence all participants were analysed in the group to which they were randomised, and missing end-points at follow-up were imputed using a regression model with the best available predictors of outcome and the best predictors for drop out." |
| Domain 2b: Risk of bias due to deviations from the intended interventions (effect of adhering to intervention) | Low risk | 2.3 Were important non-protocol interventions balanced across intervention groups? PY  Comment. Quote:"In the early intervention group, 36 (33%) participants used medication at baseline, 3 (3%) started medication during the course and 9 (8%) stopped using medication. In the control group, 48 (44%) participants used medication at baseline, 7 (6%) started and 8 (7%) stopped medication in the period between baseline and T1. Therefore, it is unlikely that the present findings can be explained by changes in medication use."  2.4. Were there failures in implementing the intervention that could have affected the outcome? PN  2.5. Was there non-adherence to the assigned intervention regimen that could have affected participants’ outcomes? PN |
| Domain 3: Missing outcome data | Low risk | 3.1 Were data for this outcome available for all, or nearly all, participants randomized? PY  Comment. Arrived at study endpoint: 96/109 (88%) intervention group; 98/108 (91%) control group. |
| Domain 4: Risk of bias in measurement of the outcome | Some concerns | 4.1 Was the method of measuring the outcome inappropriate? PN  4.2 Could measurement or ascertainment of the outcome have differed between intervention groups? N  4.3 Were outcome assessors aware of the intervention received by study participants? NI  Comment. Quote:"The interviewers were masked to the randomisation status of the participants." The PDSS-SR is a self-reported instrument.  4.4 Could assessment of the outcome have been influenced by knowledge of intervention received? PY  Comment. Participants were aware of their treatment allocation and judged the treatment outcome by means of a self-reported questionnaire. Participants allocated to the intervention group may have been more prone to judge favourably the treatment just knowing they had not been allocated to the WL condition.  4.5 Is it likely that assessment of the outcome was influenced by knowledge of intervention received? PN |
| Domain 5: Risk of bias in selection of the reported result | Low risk | 5.1 Were the data that produced this result analysed in accordance with a pre-specified analysis plan that was finalized before unblinded outcome data were available for analysis? PY  Comment. The trial has been registered in advance (ISRCTN33407455).  5.2 Is the numerical result being assessed likely to have been selected, on the basis of the results, from multiple eligible outcome measurements within the outcome domain? N  5.3 Is the numerical result being assessed likely to have been selected, on the basis of the results, from multiple eligible analyses of the data? PN |
| OVERALL RISK OF BIAS | Low risk |  |

### Newman 1997

#### Risk of bias table

| **Bias** | **Authors' judgement** | **Support for judgement** |
| --- | --- | --- |
| Domain 1: Risk of bias arising from the randomization process | Some concerns | 1.1 Was the allocation sequence random? PY  1.2 Was the allocation sequence concealed until participants were enrolled and assigned to interventions? NI  Comment. Quote: "participants were randomly assigned". No further information.  1.3 Did baseline differences between intervention groups suggest a problem with the randomization process? PN  Comment. Quote:"We found no difference between the two groups in duration of panic, number of persons who met criteria for agoraphobia, had received previous therapy or who were on anxiety medications." |
| Domain 2a: Risk of bias due to deviations from the intended interventions (effect of assignment to intervention) | Some concerns | 2.6 Was an appropriate analysis used to estimate the effect of assignment to intervention? N  Comment. Only completers were analyzed (per-protocol approach).  2.7 Was there potential for a substantial impact (on the result) of the failure to analyse participants in the group to which they were randomized? PN  Comment. There were only one drop out per condition. |
| Domain 2b: Risk of bias due to deviations from the intended interventions (effect of adhering to intervention) | High risk | 2.3 Were important non-protocol interventions balanced across intervention groups? NI  Comment. no information provided on the balancing of off-protocol medications or other off-protocol interventions across comparison groups.  2.4. Were there failures in implementing the intervention that could have affected the outcome? PN  2.5. Was there non-adherence to the assigned intervention regimen that could have affected participants’ outcomes? PN  2.6 Was an appropriate analysis used to estimate the effect of adhering to the intervention? N  Comment. Only completers were analyzed. |
| Domain 3: Missing outcome data | Low risk | 3.1 Were data for this outcome available for all, or nearly all, participants randomized? PY  Comment. Of the 20 clients who entered treatment, 18 completed it with 9 clients and 1 dropout per condition. |
| Domain 4: Risk of bias in measurement of the outcome | Low risk | 4.1 Was the method of measuring the outcome inappropriate? PN  4.2 Could measurement or ascertainment of the outcome have differed between intervention groups? N  4.3 Were outcome assessors aware of the intervention received by study participants? NI  Comment. The Fear Questionnaire is a self-reported measure.  4.4 Could assessment of the outcome have been influenced by knowledge of intervention received? PN  Comment. Participants were aware of their treatment allocation and judged the treatment outcome by means of a self-reported questionnaire. Nonetheless, people from both groups knew they were both receiving an active intervention, thus participants probably had high and equivalent expectancies in both treatment groups. |
| Domain 5: Risk of bias in selection of the reported result | Some concerns | 5.1 Were the data that produced this result analysed in accordance with a pre-specified analysis plan that was finalized before unblinded outcome data were available for analysis? PN  Comment. No protocol available.  5.2 Is the numerical result being assessed likely to have been selected, on the basis of the results, from multiple eligible outcome measurements within the outcome domain? N  5.3 Is the numerical result being assessed likely to have been selected, on the basis of the results, from multiple eligible analyses of the data? PN |
| OVERALL RISK OF BIAS | High risk |  |

### Nordin 2010

#### Risk of bias table

| **Bias** | **Authors' judgement** | **Support for judgement** |
| --- | --- | --- |
| Domain 1: Risk of bias arising from the randomization process | Some concerns | 1.1 Was the allocation sequence random? Y  1.2 Was the allocation sequence concealed until participants were enrolled and assigned to interventions? NI  Comment. Quote:"Participants were either randomized into the treatment or control group. Randomization was arranged by an independent person without contact with the participants. This person used the www. random.org website to generate a list that was then sent to the researchers."  1.3 Did baseline differences between intervention groups suggest a problem with the randomization process? PN  Comment. Quote:"There were no significant differences between the two groups before the start of the treatment". |
| Domain 2a: Risk of bias due to deviations from the intended interventions (effect of assignment to intervention) | Low risk | 2.6 Was an appropriate analysis used to estimate the effect of assignment to intervention? Y  Comment. Quote:"Intention-totreat analysis was used, with the last observation carried forward procedure assuming no change in scores." |
| Domain 2b: Risk of bias due to deviations from the intended interventions (effect of adhering to intervention) | Low risk | 2.3 Were important non-protocol interventions balanced across intervention groups? PY  Comment. Quote:" Medication status did not moderate the outcome on the PDSS, as there were no significant interaction between medication status and outcome for the treatment group (p = .15), but statistical power for this analysis was limited."  2.4. Were there failures in implementing the intervention that could have affected the outcome? PN  2.5. Was there non-adherence to the assigned intervention regimen that could have affected participants’ outcomes? PN |
| Domain 3: Missing outcome data | Low risk | 3.1 Were data for this outcome available for all, or nearly all, participants randomized? Y  Comment. Only one drop out out of 39 participants. |
| Domain 4: Risk of bias in measurement of the outcome | Some concerns | 4.1 Was the method of measuring the outcome inappropriate? N  4.2 Could measurement or ascertainment of the outcome have differed between intervention groups? N  4.3 Were outcome assessors aware of the intervention received by study participants? NI  Comment. No information provided.  4.4 Could assessment of the outcome have been influenced by knowledge of intervention received? NI  4.5 Is it likely that assessment of the outcome was influenced by knowledge of intervention received? NI |
| Domain 5: Risk of bias in selection of the reported result | Some concerns | 5.1 Were the data that produced this result analysed in accordance with a pre-specified analysis plan that was finalized before unblinded outcome data were available for analysis? PN  Comment. No protocol available.  5.2 Is the numerical result being assessed likely to have been selected, on the basis of the results, from multiple eligible outcome measurements within the outcome domain? N  5.3 Is the numerical result being assessed likely to have been selected, on the basis of the results, from multiple eligible analyses of the data? PN |
| OVERALL RISK OF BIAS | Some concerns | . |

### Oh 2020

#### Risk of bias table

| **Bias** | **Authors' judgement** | **Support for judgement** |
| --- | --- | --- |
| Domain 1: Risk of bias arising from the randomization process | Some concerns | 1.1 Was the allocation sequence random? Y  1.2 Was the allocation sequence concealed until participants were enrolled and assigned to interventions? NI  Comment. Quote:"Assignment. Forty-five patients were randomly assigned to either the chatbot group or control group (23 and 22, respectively)."  1.3 Did baseline differences between intervention groups suggest a problem with the randomization process? PN  Comment. Quote:"No significant differences in baseline demographic and clinical characteristics including medication were found between the two groups." |
| Domain 2a: Risk of bias due to deviations from the intended interventions (effect of assignment to intervention) | Some concerns | 2.6 Was an appropriate analysis used to estimate the effect of assignment to intervention? N  Comment. Only completers were analyzed (per-protocol approach).  2.7 Was there potential for a substantial impact (on the result) of the failure to analyse participants in the group to which they were randomized? PN  Comment. There were only two drop-outs per condition. |
| Domain 2b: Risk of bias due to deviations from the intended interventions (effect of adhering to intervention) | High risk | 2.3 Were important non-protocol interventions balanced across intervention groups? PN  Comment. Quote:"Although medication doses were unchanged during the study period, the effects of medications cannot be ruled out because emergency medications for panic attacks including benzodiazepine were not prohibited and the two groups did not take the same types or doses of medication."  2.4. Were there failures in implementing the intervention that could have affected the outcome? PN  2.5. Was there non-adherence to the assigned intervention regimen that could have affected participants’ outcomes? PN  2.6 Was an appropriate analysis used to estimate the effect of adhering to the intervention? N  Comment. Only completers were analyzed. |
| Domain 3: Missing outcome data | Low risk | 3.1 Were data for this outcome available for all, or nearly all, participants randomized? PY  Comment. Only two drop outs out of 45 participants. |
| Domain 4: Risk of bias in measurement of the outcome | Low risk | 4.1 Was the method of measuring the outcome inappropriate? N  4.2 Could measurement or ascertainment of the outcome have differed between intervention groups? N  4.3 Were outcome assessors aware of the intervention received by study participants? N  Comment. Quote:"post-treatment and follow-up assessment by the author who was kept blind to the treatment the patients had received." |
| Domain 5: Risk of bias in selection of the reported result | Low risk | 5.1 Were the data that produced this result analysed in accordance with a pre-specified analysis plan that was finalized before unblinded outcome data were available for analysis? Y  Comment. Quote:"The study design and protocol were approved by the Institutional Review Board of Yonsei University Gangnam Severance hospital."  5.2 Is the numerical result being assessed likely to have been selected, on the basis of the results, from multiple eligible outcome measurements within the outcome domain? N  5.3 Is the numerical result being assessed likely to have been selected, on the basis of the results, from multiple eligible analyses of the data? PN |
| OVERALL RISK OF BIAS | Some concerns | . |

### Oromendia 2016

#### Risk of bias table

| **Bias** | **Authors' judgement** | **Support for judgement** |
| --- | --- | --- |
| Domain 1: Risk of bias arising from the randomization process | Some concerns | 1.1 Was the allocation sequence random? Y  1.2 Was the allocation sequence concealed until participants were enrolled and assigned to interventions? NI  Comment. Quote:"77 participants were randomly assigned to one of the three experimental conditions: NPS, SPS, or WL. An independent researcher made the allocation schedule, using a computerized random number generator. The allocation list was generated via a simple randomization process and divided into three groups".  1.3 Did baseline differences between intervention groups suggest a problem with the randomization process? PN  Comment. Quote:"Pre-treatment analyses revealed no group differences in demographic variables or pre-treatment measures". |
| Domain 2a: Risk of bias due to deviations from the intended interventions (effect of assignment to intervention) | Low risk | 2.6 Was an appropriate analysis used to estimate the effect of assignment to intervention? Y  Comment. Quote:"Nine individuals (six from NPS and three from SPS) could not be evaluated, so the missing data were imputed using the last observation carried forward (LOCF) method." |
| Domain 2b: Risk of bias due to deviations from the intended interventions (effect of adhering to intervention) | Some concerns | 2.3 Were important non-protocol interventions balanced across intervention groups? NI  Comment. No information provided on the balancing of off-protocol medications or other off-protocol interventions across comparison groups.  2.4. Were there failures in implementing the intervention that could have affected the outcome? PN  2.5. Was there non-adherence to the assigned intervention regimen that could have affected participants’ outcomes? PY  Comment. Quote:"The mean of completed modules by individuals of the NPS group was 3.54 (SD = 1.35), whereas the individuals of the SPS group completed a mean of 5.46 modules (SD = 1.64). This difference in the number of completed modules was statistically significant".  2.6 Was an appropriate analysis used to estimate the effect of adhering to the intervention? PY  Comment. The analysis was carried out on a intention-to-treat basis. |
| Domain 3: Missing outcome data | High risk | 3.1 Were data for this outcome available for all, or nearly all, participants randomized? PN  Comment. Out of the 52 participants of the treatment groups, 10 (20.8%) dropped out of treatment, 2 individuals from the SPS group (8.3%), and 8 from the NPS group (33.3%).  3.2 Is there evidence that the result was not biased by missing outcome data? PN  Comment. The difference in the number of dropouts of both groups was statistically significant.  3.3 Could missingness in the outcome depend on its true value? NI  3.4 Is it likely that missingness in the outcome depended on its true value? NI |
| Domain 4: Risk of bias in measurement of the outcome | Some concerns | 4.1 Was the method of measuring the outcome inappropriate? PN  4.2 Could measurement or ascertainment of the outcome have differed between intervention groups? N  4.3 Were outcome assessors aware of the intervention received by study participants? NI  Comment. No information provided. The PDSS-SR is a self-reported instrument.  4.4 Could assessment of the outcome have been influenced by knowledge of intervention received? PY  Comment. Participants were aware of their treatment allocation and judged the treatment outcome by means of a self-reported questionnaire. Participants allocated to the intervention group may have been more prone to judge favourably the treatment just knowing they had not been allocated to the WL condition.  4.5 Is it likely that assessment of the outcome was influenced by knowledge of intervention received? PN |
| Domain 5: Risk of bias in selection of the reported result | Some concerns | 5.1 Were the data that produced this result analysed in accordance with a pre-specified analysis plan that was finalized before unblinded outcome data were available for analysis? PN  Comment. No protocol available.  5.2 Is the numerical result being assessed likely to have been selected, on the basis of the results, from multiple eligible outcome measurements within the outcome domain? N  5.3 Is the numerical result being assessed likely to have been selected, on the basis of the results, from multiple eligible analyses of the data? PN |
| OVERALL RISK OF BIAS | High risk |  |

### Ost 2004

#### Risk of bias table

| **Bias** | **Authors' judgement** | **Support for judgement** |
| --- | --- | --- |
| Domain 1: Risk of bias arising from the randomization process | Some concerns | 1.1 Was the allocation sequence random? PY  1.2 Was the allocation sequence concealed until participants were enrolled and assigned to interventions? NI  Comment. Quote:"The patients were randomly assigned to three conditions". No further information.  1.3 Did baseline differences between intervention groups suggest a problem with the randomization process? NI  Comment. No information provided. |
| Domain 2a: Risk of bias due to deviations from the intended interventions (effect of assignment to intervention) | Low risk | 2.6 Was an appropriate analysis used to estimate the effect of assignment to intervention? Y  Comment. Quote:"intent-to-treat analysis was used and all patients who entered treatment were included. For the dropouts, their last values, i.e. the pre-treatment scores were used at post-treatment yielding a conservative evaluation." |
| Domain 2b: Risk of bias due to deviations from the intended interventions (effect of adhering to intervention) | Some concerns | 2.3 Were important non-protocol interventions balanced across intervention groups? NI  Comment. no information provided on the balancing of off-protocol medications or other off-protocol interventions across comparison groups.  2.4. Were there failures in implementing the intervention that could have affected the outcome? PN  2.5. Was there non-adherence to the assigned intervention regimen that could have affected participants’ outcomes? PN  2.6 Was an appropriate analysis used to estimate the effect of adhering to the intervention? PY  Comment. The analysis was carried out on a intention-to-treat basis. |
| Domain 3: Missing outcome data | Low risk | 3.1 Were data for this outcome available for all, or nearly all, participants randomized? PY  Comment. Quote:"Seven patients dropped out of treatment before completion; 3 (13%) in the E-group, 2 (8%) in the CBT-group, and 2 (9%) in the WLC-group, a non-significant difference." |
| Domain 4: Risk of bias in measurement of the outcome | Low risk | 4.1 Was the method of measuring the outcome inappropriate? N  4.2 Could measurement or ascertainment of the outcome have differed between intervention groups? N  4.3 Were outcome assessors aware of the intervention received by study participants? N  Comment. Quote:"An independent research assistant not involved with the treatment performed all the diagnostic interviews and ratings." |
| Domain 5: Risk of bias in selection of the reported result | Some concerns | 5.1 Were the data that produced this result analysed in accordance with a pre-specified analysis plan that was finalized before unblinded outcome data were available for analysis? PN  Comment. No protocol available.  5.2 Is the numerical result being assessed likely to have been selected, on the basis of the results, from multiple eligible outcome measurements within the outcome domain? N  5.3 Is the numerical result being assessed likely to have been selected, on the basis of the results, from multiple eligible analyses of the data? PN |
| OVERALL RISK OF BIAS | Some concerns | . |

### Pelissolo 2012

#### Risk of bias table

| **Bias** | **Authors' judgement** | **Support for judgement** |
| --- | --- | --- |
| Domain 1: Risk of bias arising from the randomization process | Some concerns | 1.1 Was the allocation sequence random? PY  1.2 Was the allocation sequence concealed until participants were enrolled and assigned to interventions? NI  Comment. Quote:"subjects were randomized". No further information.  1.3 Did baseline differences between intervention groups suggest a problem with the randomization process? PN  Comment. Quote:"Demographic and baseline clinical characteristics of the three groups found no significant between-group differences (see Table 1)". |
| Domain 2a: Risk of bias due to deviations from the intended interventions (effect of assignment to intervention) | High risk | 2.6 Was an appropriate analysis used to estimate the effect of assignment to intervention? NI  Comment. It is difficult to understand how the study authors dealt with data analysis.  2.7 Was there potential for a substantial impact (on the result) of the failure to analyse participants in the group to which they were randomized? NI |
| Domain 2b: Risk of bias due to deviations from the intended interventions (effect of adhering to intervention) | Some concerns | 2.3 Were important non-protocol interventions balanced across intervention groups? PY  Comment. Quote:"Eligible patients were not allowed to take any psychotropic medication, with the exception of low doses hypnotics, and could not receive psychotherapy during the study".  2.4. Were there failures in implementing the intervention that could have affected the outcome? PN  2.5. Was there non-adherence to the assigned intervention regimen that could have affected participants’ outcomes? PN |
| Domain 3: Missing outcome data | Some concerns | 3.1 Were data for this outcome available for all, or nearly all, participants randomized? N  Comment. Drop out rate: 10/29 VRET group; 7/31 CBT group; 12/32 WL group.  3.2 Is there evidence that the result was not biased by missing outcome data? NI  3.3 Could missingness in the outcome depend on its true value? NI  3.4 Is it likely that missingness in the outcome depended on its true value? NI |
| Domain 4: Risk of bias in measurement of the outcome | Some concerns | 4.1 Was the method of measuring the outcome inappropriate? N  4.2 Could measurement or ascertainment of the outcome have differed between intervention groups? N  4.3 Were outcome assessors aware of the intervention received by study participants? NI  Comment. No information provided  4.4 Could assessment of the outcome have been influenced by knowledge of intervention received? NI  4.5 Is it likely that assessment of the outcome was influenced by knowledge of intervention received? NI |
| Domain 5: Risk of bias in selection of the reported result | Some concerns | 5.1 Were the data that produced this result analysed in accordance with a pre-specified analysis plan that was finalized before unblinded outcome data were available for analysis? PN  Comment. No protocol available.  5.2 Is the numerical result being assessed likely to have been selected, on the basis of the results, from multiple eligible outcome measurements within the outcome domain? N  5.3 Is the numerical result being assessed likely to have been selected, on the basis of the results, from multiple eligible analyses of the data? PN |
| OVERALL RISK OF BIAS | High risk |  |

### Petterson 1996

#### Risk of bias table

| **Bias** | **Authors' judgement** | **Support for judgement** |
| --- | --- | --- |
| Domain 1: Risk of bias arising from the randomization process | Some concerns | 1.1 Was the allocation sequence random? PY  1.2 Was the allocation sequence concealed until participants were enrolled and assigned to interventions? NI  Comment. Quote:"The patients were randomly assigned". No further information.  1.3 Did baseline differences between intervention groups suggest a problem with the randomization process? PN  Comment. Quote:"Descriptive data analysis of the subjects’ demographic information was conducted on the variables of age, gender, race, marital status, education, and annual income; no significant differences between Treatment and Control groups were found". |
| Domain 2a: Risk of bias due to deviations from the intended interventions (effect of assignment to intervention) | High risk | 2.6 Was an appropriate analysis used to estimate the effect of assignment to intervention? NI  Comment. It is difficult to understand how the study authors dealt with data analysis.  2.7 Was there potential for a substantial impact (on the result) of the failure to analyse participants in the group to which they were randomized? NI |
| Domain 2b: Risk of bias due to deviations from the intended interventions (effect of adhering to intervention) | Some concerns | 2.3 Were important non-protocol interventions balanced across intervention groups? NI  Comment. no information provided on the balancing of off-protocol medications or other off-protocol interventions across comparison groups.  2.4. Were there failures in implementing the intervention that could have affected the outcome? PN  2.5. Was there non-adherence to the assigned intervention regimen that could have affected participants’ outcomes? PN  2.6 Was an appropriate analysis used to estimate the effect of adhering to the intervention? NI  Comment. It is difficult to understand how the study authors dealt with data analysis. |
| Domain 3: Missing outcome data | Some concerns | 3.1 Were data for this outcome available for all, or nearly all, participants randomized? NI  Comment. no information on drop-out rates.  3.2 Is there evidence that the result was not biased by missing outcome data? NI  3.3 Could missingness in the outcome depend on its true value? NI  3.4 Is it likely that missingness in the outcome depended on its true value? NI |
| Domain 4: Risk of bias in measurement of the outcome | Some concerns | 4.1 Was the method of measuring the outcome inappropriate? PN  4.2 Could measurement or ascertainment of the outcome have differed between intervention groups? N  4.3 Were outcome assessors aware of the intervention received by study participants? NI  Comment. No information provided. The ASI is a self-reported instrument.  4.4 Could assessment of the outcome have been influenced by knowledge of intervention received? PY  Comment. Participants were aware of their treatment allocation and judged the treatment outcome by means of a self-reported questionnaire. Participants allocated to the intervention group may have been more prone to judge favourably the treatment just knowing they had not been allocated to the WL condition..  4.5 Is it likely that assessment of the outcome was influenced by knowledge of intervention received? PN |
| Domain 5: Risk of bias in selection of the reported result | Some concerns | 5.1 Were the data that produced this result analysed in accordance with a pre-specified analysis plan that was finalized before unblinded outcome data were available for analysis? PN  Comment. No protocol available.  5.2 Is the numerical result being assessed likely to have been selected, on the basis of the results, from multiple eligible outcome measurements within the outcome domain? N  5.3 Is the numerical result being assessed likely to have been selected, on the basis of the results, from multiple eligible analyses of the data? PN |
| OVERALL RISK OF BIAS | High risk |  |

### Pitti 2015

#### Risk of bias table

| **Bias** | **Authors' judgement** | **Support for judgement** |
| --- | --- | --- |
| Domain 1: Risk of bias arising from the randomization process | Low risk | 1.1 Was the allocation sequence random? PY  1.2 Was the allocation sequence concealed until participants were enrolled and assigned to interventions? PY  Comment. Quote:"Patients were assigned to any of the three treatment groups (PX-CBT, PXCBT-VRET, and PX) according to a random computergenerated sequence. Consecutive numbers were assigned to patients when they accepted to participate."  1.3 Did baseline differences between intervention groups suggest a problem with the randomization process? PN  Comment. Quote: "no significant differences were found according to sex or diagnosis", no further information. |
| Domain 2a: Risk of bias due to deviations from the intended interventions (effect of assignment to intervention) | High risk | 2.6 Was an appropriate analysis used to estimate the effect of assignment to intervention? NI  Comment. It is difficult to understand how the study authors dealt with data analysis.  2.7 Was there potential for a substantial impact (on the result) of the failure to analyse participants in the group to which they were randomized? NI |
| Domain 2b: Risk of bias due to deviations from the intended interventions (effect of adhering to intervention) | High risk | 2.3 Were important non-protocol interventions balanced across intervention groups? PN  Comment. Quote:"Of the 20 patients assessed in the PX- CBT group, 10 (50%) had begun to decrease the dose of paroxetine or discontinue its use. In the PX-CBT-VRET group, 19 patients were assessed. In this group, the proportion of patients quitting paroxetine was higher: 15 patients (78.9%) had begun to discontinue the medication."  2.4. Were there failures in implementing the intervention that could have affected the outcome? PN  2.5. Was there non-adherence to the assigned intervention regimen that could have affected participants’ outcomes? PN  2.6 Was an appropriate analysis used to estimate the effect of adhering to the intervention? NI  Comment. It is difficult to understand how the study authors dealt with data analysis. |
| Domain 3: Missing outcome data | High risk | 3.1 Were data for this outcome available for all, or nearly all, participants randomized? PN  Comment. 7/27 (26%) patients in the PX+CBT group and 8/27 (29%) in the PX+CBT+VRET group dropped out.  3.2 Is there evidence that the result was not biased by missing outcome data? PN  Comment. Drop outs are unbalanced (no one dropped out from the PX group).  3.3 Could missingness in the outcome depend on its true value? NI  3.4 Is it likely that missingness in the outcome depended on its true value? NI |
| Domain 4: Risk of bias in measurement of the outcome | Low risk | 4.1 Was the method of measuring the outcome inappropriate? PN  4.2 Could measurement or ascertainment of the outcome have differed between intervention groups? N  4.3 Were outcome assessors aware of the intervention received by study participants? NI  Comment. no information provided. The ACQ is a self-reported instrument.  4.4 Could assessment of the outcome have been influenced by knowledge of intervention received? PN  Comment. Participants were aware of their treatment allocation and judged the treatment outcome by means of a self-reported questionnaire. Nonetheless, people from both groups knew they were both receiving an active intervention, thus participants probably had high and equivalent expectancies in both treatment groups. |
| Domain 5: Risk of bias in selection of the reported result | Some concerns | 5.1 Were the data that produced this result analysed in accordance with a pre-specified analysis plan that was finalized before unblinded outcome data were available for analysis? PN  Comment. No protocol available.  5.2 Is the numerical result being assessed likely to have been selected, on the basis of the results, from multiple eligible outcome measurements within the outcome domain? N  5.3 Is the numerical result being assessed likely to have been selected, on the basis of the results, from multiple eligible analyses of the data? PN |
| OVERALL RISK OF BIAS | High risk |  |

### Reinecke 2013

#### Risk of bias table

| **Bias** | **Authors' judgement** | **Support for judgement** |
| --- | --- | --- |
| Domain 1: Risk of bias arising from the randomization process | Some concerns | 1.1 Was the allocation sequence random? PY  1.2 Was the allocation sequence concealed until participants were enrolled and assigned to interventions? NI  Comment. Quote:"They were randomly assigned to one of two experimental conditions". No further information.  1.3 Did baseline differences between intervention groups suggest a problem with the randomization process? PN  Comment. Quote:"The two groups were not different with respect to gender, age, years of education, and verbal intelligence; and they experienced similar degrees of panic severity and panic attack frequency at baseline. Furthermore, they were well-matched in terms of primary diagnosis". |
| Domain 2a: Risk of bias due to deviations from the intended interventions (effect of assignment to intervention) | Some concerns | 2.6 Was an appropriate analysis used to estimate the effect of assignment to intervention? NI  Comment. No information provided.  2.7 Was there potential for a substantial impact (on the result) of the failure to analyse participants in the group to which they were randomized? PN  Comment. No participant withdrew from the study. |
| Domain 2b: Risk of bias due to deviations from the intended interventions (effect of adhering to intervention) | Low risk | 2.3 Were important non-protocol interventions balanced across intervention groups? PY  Comment. Off-protocol medication where balanced.  2.4. Were there failures in implementing the intervention that could have affected the outcome? PN  2.5. Was there non-adherence to the assigned intervention regimen that could have affected participants’ outcomes? PN |
| Domain 3: Missing outcome data | Low risk | 3.1 Were data for this outcome available for all, or nearly all, participants randomized? Y  Comment. All patients were assessed at study end-point. |
| Domain 4: Risk of bias in measurement of the outcome | Some concerns | 4.1 Was the method of measuring the outcome inappropriate? PN  4.2 Could measurement or ascertainment of the outcome have differed between intervention groups? N  4.3 Were outcome assessors aware of the intervention received by study participants? NI  Comment. No information provided. The ACQ is a self-reported instrument.  4.4 Could assessment of the outcome have been influenced by knowledge of intervention received? PY  Comment. Participants were aware of their treatment allocation and judged the treatment outcome by means of a self-reported questionnaire. Participants allocated to the intervention group may have been more prone to judge favourably the treatment just knowing they had not been allocated to the TAU condition.  4.5 Is it likely that assessment of the outcome was influenced by knowledge of intervention received? PN |
| Domain 5: Risk of bias in selection of the reported result | Some concerns | 5.1 Were the data that produced this result analysed in accordance with a pre-specified analysis plan that was finalized before unblinded outcome data were available for analysis? PN  Comment. No protocol available.  5.2 Is the numerical result being assessed likely to have been selected, on the basis of the results, from multiple eligible outcome measurements within the outcome domain? N  5.3 Is the numerical result being assessed likely to have been selected, on the basis of the results, from multiple eligible analyses of the data? PN |
| OVERALL RISK OF BIAS | High risk |  |

### Richards 2006

#### Risk of bias table

| **Bias** | **Authors' judgement** | **Support for judgement** |
| --- | --- | --- |
| Domain 1: Risk of bias arising from the randomization process | High risk | 1.1 Was the allocation sequence random? PY  1.2 Was the allocation sequence concealed until participants were enrolled and assigned to interventions? NI  Comment. Quote:"The patients were randomly assigned". No further information.  1.3 Did baseline differences between intervention groups suggest a problem with the randomization process? PY  Comment. Quote: "A significant pre-assessment difference was found on DASS depression". |
| Domain 2a: Risk of bias due to deviations from the intended interventions (effect of assignment to intervention) | Low risk | 2.6 Was an appropriate analysis used to estimate the effect of assignment to intervention? Y  Comment. Quote:"Data analysis involved intention-to-treat analyses. That is, for those participants who discontinued their involvement during treatment (n=5), their preassessment scores were carried forward and used in both the posttreatment and follow-up assessments. |
| Domain 2b: Risk of bias due to deviations from the intended interventions (effect of adhering to intervention) | Low risk | 2.3 Were important non-protocol interventions balanced across intervention groups? Y  Comment. Quote:"No significant differences between the three groups were found using chi-square analyses (for nominal data) or one-way ANOVA for medication use".  2.4. Were there failures in implementing the intervention that could have affected the outcome? PN  2.5. Was there non-adherence to the assigned intervention regimen that could have affected participants’ outcomes? PN |
| Domain 3: Missing outcome data | Some concerns | 3.1 Were data for this outcome available for all, or nearly all, participants randomized? PY  Comment. Quote:"Of the 32 people who commenced the study, five discontinued during treatment. The overall attrition rate for the present study was therefore 15.6%. The attrition rate for PO1 was 16.7% (2/12), 9% (1/11) in PO2 and 22% (2/9) in IC." |
| Domain 4: Risk of bias in measurement of the outcome | Some concerns | 4.1 Was the method of measuring the outcome inappropriate? N  4.2 Could measurement or ascertainment of the outcome have differed between intervention groups? N  4.3 Were outcome assessors aware of the intervention received by study participants? PY  Comment. Quote:"The two assessors were the second author of the present study and a probationary registered psychologist/ PhD candidate. The second author was not blind to treatment allocation, although the other assessor was."  4.4 Could assessment of the outcome have been influenced by knowledge of intervention received? NI  4.5 Is it likely that assessment of the outcome was influenced by knowledge of intervention received? NI |
| Domain 5: Risk of bias in selection of the reported result | Some concerns | 5.1 Were the data that produced this result analysed in accordance with a pre-specified analysis plan that was finalized before unblinded outcome data were available for analysis? PN  Comment. No protocol available.  5.2 Is the numerical result being assessed likely to have been selected, on the basis of the results, from multiple eligible outcome measurements within the outcome domain? N  5.3 Is the numerical result being assessed likely to have been selected, on the basis of the results, from multiple eligible analyses of the data? PN |
| OVERALL RISK OF BIAS | High risk |  |

### Roberge 2008

#### Risk of bias table

| **Bias** | **Authors' judgement** | **Support for judgement** |
| --- | --- | --- |
| Domain 1: Risk of bias arising from the randomization process | Some concerns | 1.1 Was the allocation sequence random? PY  1.2 Was the allocation sequence concealed until participants were enrolled and assigned to interventions? NI  Comment. Quote:"Participants were randomly assigned". No further information.  1.3 Did baseline differences between intervention groups suggest a problem with the randomization process? PN  Comment. Quote:"demonstrated in Table 1, there was no evidence of significant differences on baseline clinical characteristics for the three treatment conditions". |
| Domain 2a: Risk of bias due to deviations from the intended interventions (effect of assignment to intervention) | Low risk | 2.6 Was an appropriate analysis used to estimate the effect of assignment to intervention? Y  Comment. Quote:"We conducted an intent-to-treat analysis with all randomized participants" |
| Domain 2b: Risk of bias due to deviations from the intended interventions (effect of adhering to intervention) | Some concerns | 2.3 Were important non-protocol interventions balanced across intervention groups? NI  Comment. no information provided on the balancing of off-protocol medications or other off-protocol interventions across comparison groups.  2.4. Were there failures in implementing the intervention that could have affected the outcome? PN  2.5. Was there non-adherence to the assigned intervention regimen that could have affected participants’ outcomes? PN  2.6 Was an appropriate analysis used to estimate the effect of adhering to the intervention? Y  Comment. The analysis was carried out on a intention-to-treat basis. |
| Domain 3: Missing outcome data | Low risk | 3.1 Were data for this outcome available for all, or nearly all, participants randomized? PY  Comment. 16/100 (16%) participants discontinued treatment for various reasons. They were found in brief (n = 8), group (n = 5), and standard (n = 3) treatment conditions, but the difference did not reach statistical significance. |
| Domain 4: Risk of bias in measurement of the outcome | Low risk | 4.1 Was the method of measuring the outcome inappropriate? PN  4.2 Could measurement or ascertainment of the outcome have differed between intervention groups? N  4.3 Were outcome assessors aware of the intervention received by study participants? NI  Comment. No information provided. The ACQ is a self-reported instrument.  4.4 Could assessment of the outcome have been influenced by knowledge of intervention received? PN  Comment. Participants were aware of their treatment allocation and judged the treatment outcome by means of a self-reported questionnaire. Nonetheless, people from both groups knew they were both receiving an active intervention, thus participants probably had high and equivalent expectancies in both treatment groups. |
| Domain 5: Risk of bias in selection of the reported result | Some concerns | 5.1 Were the data that produced this result analysed in accordance with a pre-specified analysis plan that was finalized before unblinded outcome data were available for analysis? PN  Comment. No protocol available.  5.2 Is the numerical result being assessed likely to have been selected, on the basis of the results, from multiple eligible outcome measurements within the outcome domain? N  5.3 Is the numerical result being assessed likely to have been selected, on the basis of the results, from multiple eligible analyses of the data? PN |
| OVERALL RISK OF BIAS | Some concerns | . |

### Roy-Byrne 2005

#### Risk of bias table

| **Bias** | **Authors' judgement** | **Support for judgement** |
| --- | --- | --- |
| Domain 1: Risk of bias arising from the randomization process | Some concerns | 1.1 Was the allocation sequence random? Y  1.2 Was the allocation sequence concealed until participants were enrolled and assigned to interventions? NI  Comment. Quote:"study coordinator whorandomized subjects using alternating assignment, stratified within site by comorbid major depression and referral status (referred vs screened)".  1.3 Did baseline differences between intervention groups suggest a problem with the randomization process? PN  Comment. Quote:"Intervention and usual care groups were comparable at baseline on all measures." |
| Domain 2a: Risk of bias due to deviations from the intended interventions (effect of assignment to intervention) | Low risk | 2.6 Was an appropriate analysis used to estimate the effect of assignment to intervention? Y  Comment. Quote:"We conducted intent-to-treat analyses, where all randomized patients were included in the analysis whether they continued in the study." |
| Domain 2b: Risk of bias due to deviations from the intended interventions (effect of adhering to intervention) | Low risk | 2.3 Were important non-protocol interventions balanced across intervention groups? PY  Comment. Off-protocol medication where balanced.  2.4. Were there failures in implementing the intervention that could have affected the outcome? PN  2.5. Was there non-adherence to the assigned intervention regimen that could have affected participants’ outcomes? PN |
| Domain 3: Missing outcome data | Some concerns | 3.1 Were data for this outcome available for all, or nearly all, participants randomized? Y  Comment. 24/113 (21%) dropouts in the usual care group and 29/119 (24%) in the intervention group. |
| Domain 4: Risk of bias in measurement of the outcome | Some concerns | 4.1 Was the method of measuring the outcome inappropriate? PN  4.2 Could measurement or ascertainment of the outcome have differed between intervention groups? N  4.3 Were outcome assessors aware of the intervention received by study participants? NI  Comment. Quote:"Assessments were derived from telephone interviewer administered questionnaires, queried by interviewers blind to subject intervention status, at baseline and every 3 months during the course of the study." The ASI is a self-reported questionnaire.  4.4 Could assessment of the outcome have been influenced by knowledge of intervention received? PY  Comment. Participants were aware of their treatment allocation and judged the treatment outcome by means of a self-reported questionnaire. Participants allocated to the intervention group may have been more prone to judge favourably the treatment just knowing they had not been allocated to the TAU condition.  4.5 Is it likely that assessment of the outcome was influenced by knowledge of intervention received? PN |
| Domain 5: Risk of bias in selection of the reported result | Some concerns | 5.1 Were the data that produced this result analysed in accordance with a pre-specified analysis plan that was finalized before unblinded outcome data were available for analysis? PN  Comment. No protocol available.  5.2 Is the numerical result being assessed likely to have been selected, on the basis of the results, from multiple eligible outcome measurements within the outcome domain? N  5.3 Is the numerical result being assessed likely to have been selected, on the basis of the results, from multiple eligible analyses of the data? PN |
| OVERALL RISK OF BIAS | High risk |  |

### Roy-Byrne 2010

#### Risk of bias table

| **Bias** | **Authors' judgement** | **Support for judgement** |
| --- | --- | --- |
| Domain 1: Risk of bias arising from the randomization process | Low risk | 1.1 Was the allocation sequence random? Y  1.2 Was the allocation sequence concealed until participants were enrolled and assigned to interventions? Y  Comment. Quote:"Patients were randomized to intervention or UC, using an automated computer program at RAND Corporation (Santa Monica, California), where all posteligibility assessments were conducted by telephone. Randomization was stratified by clinic and presence of comorbid major depression using a permuted block design. Block size was masked to all clinical site study members."  1.3 Did baseline differences between intervention groups suggest a problem with the randomization process? PN  Comment. Quote:"The intervention and UC groups were comparable on all baseline characteristics". |
| Domain 2a: Risk of bias due to deviations from the intended interventions (effect of assignment to intervention) | Low risk | 2.6 Was an appropriate analysis used to estimate the effect of assignment to intervention? Y  Comment. Intention-to-treat analysis was used. It includes all the patients with a baseline assessment. |
| Domain 2b: Risk of bias due to deviations from the intended interventions (effect of adhering to intervention) | Low risk | 2.3 Were important non-protocol interventions balanced across intervention groups? PY  Comment. Off-protocol medication where balanced (table 2).  2.4. Were there failures in implementing the intervention that could have affected the outcome? PN  2.5. Was there non-adherence to the assigned intervention regimen that could have affected participants’ outcomes? PN |
| Domain 3: Missing outcome data | Some concerns | 3.1 Were data for this outcome available for all, or nearly all, participants randomized? NI  Comment. Data were not available with regard to the PD patients subsample.  3.2 Is there evidence that the result was not biased by missing outcome data? NI  3.3 Could missingness in the outcome depend on its true value? NI  3.4 Is it likely that missingness in the outcome depended on its true value? NI |
| Domain 4: Risk of bias in measurement of the outcome | Low risk | 4.1 Was the method of measuring the outcome inappropriate? N  4.2 Could measurement or ascertainment of the outcome have differed between intervention groups? N  4.3 Were outcome assessors aware of the intervention received by study participants? N  Comment. Assessments were described as "blinded". |
| Domain 5: Risk of bias in selection of the reported result | Some concerns | 5.1 Were the data that produced this result analysed in accordance with a pre-specified analysis plan that was finalized before unblinded outcome data were available for analysis? PN  Comment. No protocol available.  5.2 Is the numerical result being assessed likely to have been selected, on the basis of the results, from multiple eligible outcome measurements within the outcome domain? N  5.3 Is the numerical result being assessed likely to have been selected, on the basis of the results, from multiple eligible analyses of the data? PN |
| OVERALL RISK OF BIAS | Low risk |  |

### Ruwaard 2010

#### Risk of bias table

| **Bias** | **Authors' judgement** | **Support for judgement** |
| --- | --- | --- |
| Domain 1: Risk of bias arising from the randomization process | Some concerns | 1.1 Was the allocation sequence random? PY  1.2 Was the allocation sequence concealed until participants were enrolled and assigned to interventions? NI  Comment. Quote: "The participants were randomly assigned to two groups".  1.3 Did baseline differences between intervention groups suggest a problem with the randomization process? PN  Comment. No major differences between the two comparison groups have been identified inspecting table 1. |
| Domain 2a: Risk of bias due to deviations from the intended interventions (effect of assignment to intervention) | Low risk | 2.6 Was an appropriate analysis used to estimate the effect of assignment to intervention? Y  Comment. Quote:"The RCT analysis was conducted on an intention-to-treat basis and included all participants. Participants failing to complete posttest measurements were assumed to have gained nothing. Their pretest scores served as their posttest scores." |
| Domain 2b: Risk of bias due to deviations from the intended interventions (effect of adhering to intervention) | Low risk | 2.3 Were important non-protocol interventions balanced across intervention groups? PY  Comment. Quote:"With respect to the primary outcome measures, those who received additional treatment did not score differently from those without additional treatment."  2.4. Were there failures in implementing the intervention that could have affected the outcome? PN  2.5. Was there non-adherence to the assigned intervention regimen that could have affected participants’ outcomes? PN |
| Domain 3: Missing outcome data | Low risk | 3.1 Were data for this outcome available for all, or nearly all, participants randomized? Y  Comment. 1/30 dropped out form the WL group and 3/24 dropped out from the intervention group. |
| Domain 4: Risk of bias in measurement of the outcome | Some concerns | 4.1 Was the method of measuring the outcome inappropriate? PN  4.2 Could measurement or ascertainment of the outcome have differed between intervention groups? N  4.3 Were outcome assessors aware of the intervention received by study participants? NI  Comment. Only self-reported measures were analyzed.  4.4 Could assessment of the outcome have been influenced by knowledge of intervention received? PY  Comment. Participants were aware of their treatment allocation and judged the treatment outcome by means of a self-reported questionnaire. Participants allocated to the intervention group may have been more prone to judge favourably the treatment just knowing they had not been allocated to the TAU condition.  4.5 Is it likely that assessment of the outcome was influenced by knowledge of intervention received? PN |
| Domain 5: Risk of bias in selection of the reported result | Some concerns | 5.1 Were the data that produced this result analysed in accordance with a pre-specified analysis plan that was finalized before unblinded outcome data were available for analysis? PN  Comment. No protocol available.  5.2 Is the numerical result being assessed likely to have been selected, on the basis of the results, from multiple eligible outcome measurements within the outcome domain? N  5.3 Is the numerical result being assessed likely to have been selected, on the basis of the results, from multiple eligible analyses of the data? PN |
| OVERALL RISK OF BIAS | Some concerns | . |

### Schmidt 1997a

#### Risk of bias table

| **Bias** | **Authors' judgement** | **Support for judgement** |
| --- | --- | --- |
| Domain 1: Risk of bias arising from the randomization process | Some concerns | 1.1 Was the allocation sequence random? PY  1.2 Was the allocation sequence concealed until participants were enrolled and assigned to interventions? NI  Comment. Quote:"Patients were randomized to treatment condition following base-line assessment". No further information.  1.3 Did baseline differences between intervention groups suggest a problem with the randomization process? PN  Comment. Quote:"Subjects in the two groups did not differ significantly on any of the demographic or clinical variables at baseline". |
| Domain 2a: Risk of bias due to deviations from the intended interventions (effect of assignment to intervention) | High risk | 2.6 Was an appropriate analysis used to estimate the effect of assignment to intervention? PN  Comment. It is difficult to understand how the study authors dealt with data analysis, but it is likely that they didn't take into consideration data from dropouts.  2.7 Was there potential for a substantial impact (on the result) of the failure to analyse participants in the group to which they were randomized? NI |
| Domain 2b: Risk of bias due to deviations from the intended interventions (effect of adhering to intervention) | Low risk | 2.3 Were important non-protocol interventions balanced across intervention groups? PY  Comment. Quote:"Medicated patients receiving treatment did not sig- nificantly differ from unmedicated patientson any ofthe clinical measures at base line, posttreatment, or follow-up (p > 0.05)."  2.4. Were there failures in implementing the intervention that could have affected the outcome? PN  2.5. Was there non-adherence to the assigned intervention regimen that could have affected participants’ outcomes? PN |
| Domain 3: Missing outcome data | Some concerns | 3.1 Were data for this outcome available for all, or nearly all, participants randomized? PN  Comment. Dropouts were similar across conditions, with 20% of patients in the immediate treatment condition(n = 5) and 25% of patients in the delayed treatment condition (n = 3) discontinuing their participation.  3.2 Is there evidence that the result was not biased by missing outcome data? NI  3.3 Could missingness in the outcome depend on its true value? NI  3.4 Is it likely that missingness in the outcome depended on its true value? NI |
| Domain 4: Risk of bias in measurement of the outcome | Some concerns | 4.1 Was the method of measuring the outcome inappropriate? PN  4.2 Could measurement or ascertainment of the outcome have differed between intervention groups? N  4.3 Were outcome assessors aware of the intervention received by study participants? NI  Comment. Only self-reported measures were analyzed.  4.4 Could assessment of the outcome have been influenced by knowledge of intervention received? PY  Comment. Participants were aware of their treatment allocation and judged the treatment outcome by means of a self-reported questionnaire. Participants allocated to the intervention group may have been more prone to judge favourably the treatment just knowing they had not been allocated to the TAU condition.  4.5 Is it likely that assessment of the outcome was influenced by knowledge of intervention received? PN |
| Domain 5: Risk of bias in selection of the reported result | Some concerns | 5.1 Were the data that produced this result analysed in accordance with a pre-specified analysis plan that was finalized before unblinded outcome data were available for analysis? PN  Comment. No protocol available.  5.2 Is the numerical result being assessed likely to have been selected, on the basis of the results, from multiple eligible outcome measurements within the outcome domain? N  5.3 Is the numerical result being assessed likely to have been selected, on the basis of the results, from multiple eligible analyses of the data? PN |
| OVERALL RISK OF BIAS | High risk |  |

### Schmidt 1997b

#### Risk of bias table

| **Bias** | **Authors' judgement** | **Support for judgement** |
| --- | --- | --- |
| Domain 1: Risk of bias arising from the randomization process | Some concerns | 1.1 Was the allocation sequence random? PY  1.2 Was the allocation sequence concealed until participants were enrolled and assigned to interventions? NI  Comment. Quote:"participants were arandomly assigned". No further information.  1.3 Did baseline differences between intervention groups suggest a problem with the randomization process? PN  Comment. Groups did not differ on demographic characteristics. |
| Domain 2a: Risk of bias due to deviations from the intended interventions (effect of assignment to intervention) | High risk | 2.6 Was an appropriate analysis used to estimate the effect of assignment to intervention? PN  Comment. Of those patients assigned to the treatment conditions (54), 34 were assessed at posttreatment.  2.7 Was there potential for a substantial impact (on the result) of the failure to analyse participants in the group to which they were randomized? NI |
| Domain 2b: Risk of bias due to deviations from the intended interventions (effect of adhering to intervention) | Low risk | 2.3 Were important non-protocol interventions balanced across intervention groups? PY  Comment. Quote:"Medication status was not associated with subjective response, physiological response, or panic during either serial CO2 challenge (ps > .05)."  2.4. Were there failures in implementing the intervention that could have affected the outcome? PN  2.5. Was there non-adherence to the assigned intervention regimen that could have affected participants’ outcomes? PN |
| Domain 3: Missing outcome data | Low risk | 3.1 Were data for this outcome available for all, or nearly all, participants randomized? PY  Comment. Quote:"there were two dropouts in each condition. Four individuals from the delayed treatment condition discontinued their participation prior to posttesting". |
| Domain 4: Risk of bias in measurement of the outcome | Some concerns | 4.1 Was the method of measuring the outcome inappropriate? PN  4.2 Could measurement or ascertainment of the outcome have differed between intervention groups? N  4.3 Were outcome assessors aware of the intervention received by study participants? NI  Comment. Quote:"Overall clinical impression of severity of illness were made based on a semistructured clinical interview using raters unaware of experimental condition". Nonetheless, the ASI is a self-reported measure.  4.4 Could assessment of the outcome have been influenced by knowledge of intervention received? PY  Comment. Participants were aware of their treatment allocation and judged the treatment outcome by means of a self-reported questionnaire. Participants allocated to the intervention group may have been more prone to judge favourably the treatment just knowing they had not been allocated to the TAU condition.  4.5 Is it likely that assessment of the outcome was influenced by knowledge of intervention received? PN |
| Domain 5: Risk of bias in selection of the reported result | Some concerns | 5.1 Were the data that produced this result analysed in accordance with a pre-specified analysis plan that was finalized before unblinded outcome data were available for analysis? PN  Comment. No protocol available.  5.2 Is the numerical result being assessed likely to have been selected, on the basis of the results, from multiple eligible outcome measurements within the outcome domain? N  5.3 Is the numerical result being assessed likely to have been selected, on the basis of the results, from multiple eligible analyses of the data? PN |
| OVERALL RISK OF BIAS | High risk | . |

### Sharp 1997

#### Risk of bias table

| **Bias** | **Authors' judgement** | **Support for judgement** |
| --- | --- | --- |
| Domain 1: Risk of bias arising from the randomization process | Some concerns | 1.1 Was the allocation sequence random? PY  1.2 Was the allocation sequence concealed until participants were enrolled and assigned to interventions? NI  Comment. Quote: "Patients were randomly allocated to one of five treatment groups". No further information.  1.3 Did baseline differences between intervention groups suggest a problem with the randomization process? NI  Comment. Scant information are given on baseline demographic and clinical features. |
| Domain 2a: Risk of bias due to deviations from the intended interventions (effect of assignment to intervention) | Some concerns | 2.6 Was an appropriate analysis used to estimate the effect of assignment to intervention? PN  Comment. Authors considered as completers participants that completed at least 42 days of treatment and who provided adequate end-point data. Doing this, authors opted neither for a ITT approach nor for a classical per-protocol analysis. Anyway, the analyzed sample was composed of 149 completers and "defined completers" out of the 193 patients initially randomized.  2.7 Was there potential for a substantial impact (on the result) of the failure to analyse participants in the group to which they were randomized? PY |
| Domain 2b: Risk of bias due to deviations from the intended interventions (effect of adhering to intervention) | Low risk | 2.3 Were important non-protocol interventions balanced across intervention groups? PY  Comment. Quote:"no psychotropic medication in the 28 days prior to entry and throughout the study treatment period".  2.4. Were there failures in implementing the intervention that could have affected the outcome? PN  2.5. Was there non-adherence to the assigned intervention regimen that could have affected participants’ outcomes? PN |
| Domain 3: Missing outcome data | Some concerns | 3.1 Were data for this outcome available for all, or nearly all, participants randomized? PN  Comment. 44 participants out of 193 dropped from the study. No information on balance or unbalance of drop out rates among comparison groups.  3.2 Is there evidence that the result was not biased by missing outcome data? NI  3.3 Could missingness in the outcome depend on its true value? NI  3.4 Is it likely that missingness in the outcome depended on its true value? NI |
| Domain 4: Risk of bias in measurement of the outcome | Low risk | 4.1 Was the method of measuring the outcome inappropriate? PN  4.2 Could measurement or ascertainment of the outcome have differed between intervention groups? N  4.3 Were outcome assessors aware of the intervention received by study participants? NI  Comment. No information provided. The GHQ is a self-reported instrument.  4.4 Could assessment of the outcome have been influenced by knowledge of intervention received? PN  Comment. Participants were aware of their treatment allocation and judged the treatment outcome by means of a self-reported questionnaire. Nonetheless, people from both groups knew they were both receiving an active intervention, thus participants probably had high and equivalent expectancies in both treatment groups. |
| Domain 5: Risk of bias in selection of the reported result | Some concerns | 5.1 Were the data that produced this result analysed in accordance with a pre-specified analysis plan that was finalized before unblinded outcome data were available for analysis? PN  Comment. No protocol available.  5.2 Is the numerical result being assessed likely to have been selected, on the basis of the results, from multiple eligible outcome measurements within the outcome domain? N  5.3 Is the numerical result being assessed likely to have been selected, on the basis of the results, from multiple eligible analyses of the data? PN |
| OVERALL RISK OF BIAS | High risk |  |

### Sharp 2000

#### Risk of bias table

| **Bias** | **Authors' judgement** | **Support for judgement** |
| --- | --- | --- |
| Domain 1: Risk of bias arising from the randomization process | Some concerns | 1.1 Was the allocation sequence random? Y  1.2 Was the allocation sequence concealed until participants were enrolled and assigned to interventions? NI  Comment. Quote:"Randomisation was conducted according to a computer-generated randomisation schedule, with individual allocations unavailable until after initial assessment."  1.3 Did baseline differences between intervention groups suggest a problem with the randomization process? PN  Comment. Quote:"There were no statistically significant differences between treatment groups on any clinical or demographic measures at entry". |
| Domain 2a: Risk of bias due to deviations from the intended interventions (effect of assignment to intervention) | Some concerns | 2.6 Was an appropriate analysis used to estimate the effect of assignment to intervention? PN  Comment. Authors considered as completers participants that completed at least 42 days of treatment and who provided adequate end-point data. Doing this, authors opted neither for a ITT approach nor for a classical per-protocol analysis. Anyway, the analyzed sample was composed of 91 completers and "defined completers" out of the 104 patients initially randomized.  2.7 Was there potential for a substantial impact (on the result) of the failure to analyse participants in the group to which they were randomized? PY |
| Domain 2b: Risk of bias due to deviations from the intended interventions (effect of adhering to intervention) | Low risk | 2.3 Were important non-protocol interventions balanced across intervention groups? PY  Comment. Quote:" In the current study, there were no statistically significant differ- ences between groups in the proportions of patients taking psychotropic medications during the study; the relatively small sample size employed, however, did not permit the investigation of possible medication by treatment group interactions".  2.4. Were there failures in implementing the intervention that could have affected the outcome? PN  2.5. Was there non-adherence to the assigned intervention regimen that could have affected participants’ outcomes? PN |
| Domain 3: Missing outcome data | Low risk | 3.1 Were data for this outcome available for all, or nearly all, participants randomized? PY  Comment. 13 participants out of 104 dropped from the study. No information on balance or unbalance of drop out rates among comparison groups. |
| Domain 4: Risk of bias in measurement of the outcome | Low risk | 4.1 Was the method of measuring the outcome inappropriate? N  4.2 Could measurement or ascertainment of the outcome have differed between intervention groups? N  4.3 Were outcome assessors aware of the intervention received by study participants? N  Comment. Quote:"Treatment end-point assessments were conducted by an independent clinical psychologist and patients were instructed not to discuss their treatment with the blind endpoint assessor." |
| Domain 5: Risk of bias in selection of the reported result | Some concerns | 5.1 Were the data that produced this result analysed in accordance with a pre-specified analysis plan that was finalized before unblinded outcome data were available for analysis? PN  Comment. No protocol available.  5.2 Is the numerical result being assessed likely to have been selected, on the basis of the results, from multiple eligible outcome measurements within the outcome domain? N  5.3 Is the numerical result being assessed likely to have been selected, on the basis of the results, from multiple eligible analyses of the data? PN |
| OVERALL RISK OF BIAS | Some concerns | . |

### Sharp 2004

#### Risk of bias table

| **Bias** | **Authors' judgement** | **Support for judgement** |
| --- | --- | --- |
| Domain 1: Risk of bias arising from the randomization process | High risk | 1.1 Was the allocation sequence random? PY  1.2 Was the allocation sequence concealed until participants were enrolled and assigned to interventions?NI  Comment. Quote:"patients were randomly allocated".  1.3 Did baseline differences between intervention groups suggest a problem with the randomization process? PY  Comment. Mean age, mean duration current episode, concurrent psychotropic medication (number of patients) were unbalanced at baseline. |
| Domain 2a: Risk of bias due to deviations from the intended interventions (effect of assignment to intervention) | Some concerns | 2.6 Was an appropriate analysis used to estimate the effect of assignment to intervention? PN  Comment. Authors considered as completers participants that completed at least 42 days of treatment and who provided adequate end-point data. Doing this, authors opted neither for a ITT approach nor for a classical per-protocol analysis. Anyway, the analyzed sample was composed of 70 completers and "defined completers" out of the 97 patients initially randomized.  2.7 Was there potential for a substantial impact (on the result) of the failure to analyse participants in the group to which they were randomized? PY |
| Domain 2b: Risk of bias due to deviations from the intended interventions (effect of adhering to intervention) | Low risk | 2.3 Were important non-protocol interventions balanced across intervention groups? PY  Comment. Quote:"There were no significant differences between treatment groups in the proportion of patients taking either psychotropic or non-psychotropic medication."  2.4. Were there failures in implementing the intervention that could have affected the outcome? PN  2.5. Was there non-adherence to the assigned intervention regimen that could have affected participants’ outcomes? PN |
| Domain 3: Missing outcome data | Some concerns | 3.1 Were data for this outcome available for all, or nearly all, participants randomized? PN  Comment. 27 participants out of 70 dropped from the study. No information on balance or unbalance of drop out rates among comparison groups.  3.2 Is there evidence that the result was not biased by missing outcome data? NI  3.3 Could missingness in the outcome depend on its true value? NI  3.4 Is it likely that missingness in the outcome depended on its true value? NI |
| Domain 4: Risk of bias in measurement of the outcome | Some concerns | 4.1 Was the method of measuring the outcome inappropriate? PN  4.2 Could measurement or ascertainment of the outcome have differed between intervention groups? N  4.3 Were outcome assessors aware of the intervention received by study participants? NI  Comment. Quote:"Treatment end-point assessments were conducted by an independent clinical psychologist ". Nonetheless, the FQ is a self-reported measure.  4.4 Could assessment of the outcome have been influenced by knowledge of intervention received? PY  Comment. Participants were aware of their treatment allocation and judged the treatment outcome by means of a self-reported questionnaire. Participants allocated to the intervention group may have been more prone to judge favourably the treatment just knowing they had not been allocated to the TAU condition.  4.5 Is it likely that assessment of the outcome was influenced by knowledge of intervention received? PN |
| Domain 5: Risk of bias in selection of the reported result | Some concerns | 5.1 Were the data that produced this result analysed in accordance with a pre-specified analysis plan that was finalized before unblinded outcome data were available for analysis? PN  Comment. No protocol available.  5.2 Is the numerical result being assessed likely to have been selected, on the basis of the results, from multiple eligible outcome measurements within the outcome domain? N  5.3 Is the numerical result being assessed likely to have been selected, on the basis of the results, from multiple eligible analyses of the data? PN |
| OVERALL RISK OF BIAS | High risk |  |

### Shear 2001

#### Risk of bias table

| **Bias** | **Authors' judgement** | **Support for judgement** |
| --- | --- | --- |
| Domain 1: Risk of bias arising from the randomization process | Some concerns | 1.1 Was the allocation sequence random? PY  1.2 Was the allocation sequence concealed until participants were enrolled and assigned to interventions? NI  Comment.Quote:"Subjects were randomly assigned to receive emotion-focused psychotherapy in a 1:6 ratio".  1.3 Did baseline differences between intervention groups suggest a problem with the randomization process? PN  Comment. Quote:"There were no significant differences on demographic or clinical measures among the different treatment groups at baseline, nor was there a significant difference in treatment expectations across the four treatment groups". |
| Domain 2a: Risk of bias due to deviations from the intended interventions (effect of assignment to intervention) | Low risk | 2.6 Was an appropriate analysis used to estimate the effect of assignment to intervention? Y  Comment. The analysis was carried out on a intention-to-treat basis. |
| Domain 2b: Risk of bias due to deviations from the intended interventions (effect of adhering to intervention) | Low risk | 2.3 Were important non-protocol interventions balanced across intervention groups? PY  Comment. Quote:"subjects were required to discontinue any psychotropic medication and ongoing psychotherapy".  2.4. Were there failures in implementing the intervention that could have affected the outcome? PN  2.5. Was there non-adherence to the assigned intervention regimen that could have affected participants’ outcomes? PN |
| Domain 3: Missing outcome data | High risk | 3.1 Were data for this outcome available for all, or nearly all, participants randomized? N  Comment. 40 out of 113 (35%) participants dropped out from the study. The attrition rate was: 7/30 patients receiving emotion-focused psychotherapy (23%), 14/36 patients receiving cognitive behavior therapy (39%), 19/23 patients given placebo (39%), and 10/24 patients receiving imipramine (42%).  3.2 Is there evidence that the result was not biased by missing outcome data? PN  Comment. Drop outs were not perfectly balanced across treatment groups: E-F psychotherapy: 7/30 (23%); Imipramine: 10/24 (41%); CBT: 14/36 (39%); Placebo: 9/23 (39%).  3.3 Could missingness in the outcome depend on its true value? NI  3.4 Is it likely that missingness in the outcome depended on its true value? NI |
| Domain 4: Risk of bias in measurement of the outcome | Low risk | 4.1 Was the method of measuring the outcome inappropriate? N  4.2 Could measurement or ascertainment of the outcome have differed between intervention groups? N  4.3 Were outcome assessors aware of the intervention received by study participants? N  Comment. Quote:"Study assessments were performed by independent evaluators". |
| Domain 5: Risk of bias in selection of the reported result | Some concerns | 5.1 Were the data that produced this result analysed in accordance with a pre-specified analysis plan that was finalized before unblinded outcome data were available for analysis? PN  Comment. No protocol available.  5.2 Is the numerical result being assessed likely to have been selected, on the basis of the results, from multiple eligible outcome measurements within the outcome domain? N  5.3 Is the numerical result being assessed likely to have been selected, on the basis of the results, from multiple eligible analyses of the data? PN |
| OVERALL RISK OF BIAS | Some concerns | . |

### Silfvernagel 2012

#### Risk of bias table

| **Bias** | **Authors' judgement** | **Support for judgement** |
| --- | --- | --- |
| Domain 1: Risk of bias arising from the randomization process | Some concerns | 1.1 Was the allocation sequence random? Y  1.2 Was the allocation sequence concealed until participants were enrolled and assigned to interventions? NI  Comment. Quote:"The blocked randomization process was conducted through an online true random number-generation service (random.org) independent of the investigators and therapists".  1.3 Did baseline differences between intervention groups suggest a problem with the randomization process? NI  Comment. The only reported information is about the mean number of completed modules in the young adult group and the corresponding number in the adult group. |
| Domain 2a: Risk of bias due to deviations from the intended interventions (effect of assignment to intervention) | Low risk | 2.6 Was an appropriate analysis used to estimate the effect of assignment to intervention? Y  Comment. The analysis was carried out on a intention-to-treat basis. |
| Domain 2b: Risk of bias due to deviations from the intended interventions (effect of adhering to intervention) | Low risk | 2.3 Were important non-protocol interventions balanced across intervention groups? PY  Comment. Off-protocol medication where balanced (table 2).  2.4. Were there failures in implementing the intervention that could have affected the outcome? PN  2.5. Was there non-adherence to the assigned intervention regimen that could have affected participants’ outcomes? PN |
| Domain 3: Missing outcome data | High risk | 3.1 Were data for this outcome available for all, or nearly all, participants randomized? PN  Comment. 10/29 participants discontinued the trial in the intervention group, 2/28 in the WL group.  3.2 Is there evidence that the result was not biased by missing outcome data? PN  Comment. There was an imbalance between the two comparison groups with regard to drop-out rates.  3.3 Could missingness in the outcome depend on its true value? NI  3.4 Is it likely that missingness in the outcome depended on its true value? NI |
| Domain 4: Risk of bias in measurement of the outcome | Low risk | 4.1 Was the method of measuring the outcome inappropriate? N  4.2 Could measurement or ascertainment of the outcome have differed between intervention groups? N  4.3 Were outcome assessors aware of the intervention received by study participants? N  Comment. Quote:"At posttreatment participants were instructed via email to complete the follow-up questionnaires and to participate in a semistructured telephone interview carried out by a blinded assessor". |
| Domain 5: Risk of bias in selection of the reported result | Some concerns | 5.1 Were the data that produced this result analysed in accordance with a pre-specified analysis plan that was finalized before unblinded outcome data were available for analysis? PN  Comment. No protocol available.  5.2 Is the numerical result being assessed likely to have been selected, on the basis of the results, from multiple eligible outcome measurements within the outcome domain? N  5.3 Is the numerical result being assessed likely to have been selected, on the basis of the results, from multiple eligible analyses of the data? PN |
| OVERALL RISK OF BIAS | Some concerns | . |

### Telch 1993

#### Risk of bias table

| **Bias** | **Authors' judgement** | **Support for judgement** |
| --- | --- | --- |
| Domain 1: Risk of bias arising from the randomization process | Some concerns | 1.1 Was the allocation sequence random? PY  1.2 Was the allocation sequence concealed until participants were enrolled and assigned to interventions? NI  Comment. Quote:"patients were randomly allocated".  1.3 Did baseline differences between intervention groups suggest a problem with the randomization process? PN  Comment. Quote:"Subjects in the two groups did not differ si~ificantly on any of the demographic variables at intake with the exception that a greater percentage of delayed treatment controls had received psychosocial treatment for panic". |
| Domain 2a: Risk of bias due to deviations from the intended interventions (effect of assignment to intervention) | Low risk | 2.6 Was an appropriate analysis used to estimate the effect of assignment to intervention? NI  Comment. No information reported.  2.7 Was there potential for a substantial impact (on the result) of the failure to analyse participants in the group to which they were randomized? PN  Comment. There were no drop-outs, it is not important to know how data were handled. |
| Domain 2b: Risk of bias due to deviations from the intended interventions (effect of adhering to intervention) | Low risk | 2.3 Were important non-protocol interventions balanced across intervention groups? PY  Comment. Quote:"Medicated Ss receiving group panic inoculation treatment did not differ significantly from unmedicated Ss on any of the clinical measures at baseline, posttreatment or 6 month follow-up."  2.4. Were there failures in implementing the intervention that could have affected the outcome? PN  2.5. Was there non-adherence to the assigned intervention regimen that could have affected participants’ outcomes? PN |
| Domain 3: Missing outcome data | Low risk | 3.1 Were data for this outcome available for all, or nearly all, participants randomized? NI  Comment. Apparently , none dropped-out from the study. |
| Domain 4: Risk of bias in measurement of the outcome | Some concerns | 4.1 Was the method of measuring the outcome inappropriate? PN  4.2 Could measurement or ascertainment of the outcome have differed between intervention groups? N  4.3 Were outcome assessors aware of the intervention received by study participants? NI  Comment. Measures were self-reported.  4.4 Could assessment of the outcome have been influenced by knowledge of intervention received? PY  Comment. Participants were aware of their treatment allocation and judged the treatment outcome by means of a self-reported questionnaire. Participants allocated to the intervention group may have been more prone to judge favourably the treatment just knowing they had not been allocated to the TAU condition.  4.5 Is it likely that assessment of the outcome was influenced by knowledge of intervention received? PN |
| Domain 5: Risk of bias in selection of the reported result | Some concerns | 5.1 Were the data that produced this result analysed in accordance with a pre-specified analysis plan that was finalized before unblinded outcome data were available for analysis? PN  Comment. No protocol available.  5.2 Is the numerical result being assessed likely to have been selected, on the basis of the results, from multiple eligible outcome measurements within the outcome domain? N  5.3 Is the numerical result being assessed likely to have been selected, on the basis of the results, from multiple eligible analyses of the data? PN |
| OVERALL RISK OF BIAS | Some concerns | . |

### Titov 2010

#### Risk of bias table

| **Bias** | **Authors' judgement** | **Support for judgement** |
| --- | --- | --- |
| Domain 1: Risk of bias arising from the randomization process | Some concerns | 1.1 Was the allocation sequence random? Y  1.2 Was the allocation sequence concealed until participants were enrolled and assigned to interventions? NI  Comment. Quote:"applicants met all inclusion criteria and were randomized by NT via a true randomization process (www.random.org)".  1.3 Did baseline differences between intervention groups suggest a problem with the randomization process? PN  Comment. Quote:"No between-group differences on demographic characteristics or pretreatment measures were observed using one-way". |
| Domain 2a: Risk of bias due to deviations from the intended interventions (effect of assignment to intervention) | Low risk | 2.6 Was an appropriate analysis used to estimate the effect of assignment to intervention? Y  Comment. Quote:"An intention-to-treat model using the baseline-observation-carried-forward principle was employed for data analyses."  2.7 Was there potential for a substantial impact (on the result) of the failure to analyse participants in the group to which they were randomized?  Comment. |
| Domain 2b: Risk of bias due to deviations from the intended interventions (effect of adhering to intervention) | Low risk | 2.3 Were important non-protocol interventions balanced across intervention groups? PY  Comment. Off-protocol medication where balanced (table 1).  2.4. Were there failures in implementing the intervention that could have affected the outcome? PN  2.5. Was there non-adherence to the assigned intervention regimen that could have affected participants’ outcomes? PN |
| Domain 3: Missing outcome data | Low risk | 3.1 Were data for this outcome available for all, or nearly all, participants randomized? Y  Comment. No participant dropped out from the panic attack subgroup. |
| Domain 4: Risk of bias in measurement of the outcome | Some concerns | 4.1 Was the method of measuring the outcome inappropriate? PN  4.2 Could measurement or ascertainment of the outcome have differed between intervention groups? N  4.3 Were outcome assessors aware of the intervention received by study participants? NI  Comment. Measures were self-reported.  4.4 Could assessment of the outcome have been influenced by knowledge of intervention received? PY  Comment. Participants were aware of their treatment allocation and judged the treatment outcome by means of a self-reported questionnaire. Participants allocated to the intervention group may have been more prone to judge favourably the treatment just knowing they had not been allocated to the TAU condition.  4.5 Is it likely that assessment of the outcome was influenced by knowledge of intervention received? PN |
| Domain 5: Risk of bias in selection of the reported result | Some concerns | 5.1 Were the data that produced this result analysed in accordance with a pre-specified analysis plan that was finalized before unblinded outcome data were available for analysis? PN  Comment. No protocol available.  5.2 Is the numerical result being assessed likely to have been selected, on the basis of the results, from multiple eligible outcome measurements within the outcome domain? N  5.3 Is the numerical result being assessed likely to have been selected, on the basis of the results, from multiple eligible analyses of the data? PN |
| OVERALL RISK OF BIAS | Some concerns | . |

### Tyrer 1988

#### Risk of bias table

| **Bias** | **Authors' judgement** | **Support for judgement** |
| --- | --- | --- |
| Domain 1: Risk of bias arising from the randomization process | Some concerns | 1.1 Was the allocation sequence random? PY  1.2 Was the allocation sequence concealed until participants were enrolled and assigned to interventions? Y  Comment. Quote:"were allocated to one of five treatments, each given for 6 weeks and then withdrawn over the next 4 weeks. The randomised treatments were indicated by opening a sealed envelope once patients satisfied the inclusion criteria for the study."  1.3 Did baseline differences between intervention groups suggest a problem with the randomization process? NI  Comment. No information provided on the PD subsample |
| Domain 2a: Risk of bias due to deviations from the intended interventions (effect of assignment to intervention) | High risk | 2.6 Was an appropriate analysis used to estimate the effect of assignment to intervention? NI  Comment. No information reported.  2.7 Was there potential for a substantial impact (on the result) of the failure to analyse participants in the group to which they were randomized? NI |
| Domain 2b: Risk of bias due to deviations from the intended interventions (effect of adhering to intervention) | Some concerns | 2.3 Were important non-protocol interventions balanced across intervention groups? NI  Comment. No information provided on the balancing of off-protocol medications or other off-protocol interventions across comparison groups.  2.4. Were there failures in implementing the intervention that could have affected the outcome? PN  2.5. Was there non-adherence to the assigned intervention regimen that could have affected participants’ outcomes? PN  2.6 Was an appropriate analysis used to estimate the effect of adhering to the intervention? NI |
| Domain 3: Missing outcome data | Low risk | 3.1 Were data for this outcome available for all, or nearly all, participants randomized? Y  Comment. Only 1 dropout in the PD subsamples (from the CBT group). |
| Domain 4: Risk of bias in measurement of the outcome | Low risk | 4.1 Was the method of measuring the outcome inappropriate? N  4.2 Could measurement or ascertainment of the outcome have differed between intervention groups? N  4.3 Were outcome assessors aware of the intervention received by study participants? N  Comment. Quote:"the assessors were masked with respect to both treatment and diagnosis". |
| Domain 5: Risk of bias in selection of the reported result | Some concerns | 5.1 Were the data that produced this result analysed in accordance with a pre-specified analysis plan that was finalized before unblinded outcome data were available for analysis? PN  Comment. No protocol available.  5.2 Is the numerical result being assessed likely to have been selected, on the basis of the results, from multiple eligible outcome measurements within the outcome domain? N  5.3 Is the numerical result being assessed likely to have been selected, on the basis of the results, from multiple eligible analyses of the data? PN |
| OVERALL RISK OF BIAS | High risk |  |

### van Ballegooijen 2013

#### Risk of bias table

| **Bias** | **Authors' judgement** | **Support for judgement** |
| --- | --- | --- |
| Domain 1: Risk of bias arising from the randomization process | Some concerns | 1.1 Was the allocation sequence random? PY  1.2 Was the allocation sequence concealed until participants were enrolled and assigned to interventions? NI  Comment. Quote:"Randomization lists were generated automatically using a computer program".  1.3 Did baseline differences between intervention groups suggest a problem with the randomization process? PN  Comment. Quote:"The control group had slightly higher baseline scores than theintervention group, but there were no to little further differences between the intervention group and control group. |
| Domain 2a: Risk of bias due to deviations from the intended interventions (effect of assignment to intervention) | Low risk | 2.6 Was an appropriate analysis used to estimate the effect of assignment to intervention? Y  Comment. The data were analyzed in agreement with the intention-to-treat (ITT) principle. |
| Domain 2b: Risk of bias due to deviations from the intended interventions (effect of adhering to intervention) | Some concerns | 2.3 Were important non-protocol interventions balanced across intervention groups? NI  Comment. Quote:"all participants in our trial were free to use medication and find other treatment." no further information.  2.4. Were there failures in implementing the intervention that could have affected the outcome? PN  2.5. Was there non-adherence to the assigned intervention regimen that could have affected participants’ outcomes? PN  2.6 Was an appropriate analysis used to estimate the effect of adhering to the intervention? PY  Comment. The analysis was carried out on a intention-to-treat basis. |
| Domain 3: Missing outcome data | Some concerns | 3.1 Were data for this outcome available for all, or nearly all, participants randomized? N  Comment. 34/63 participants in the intervention group and 39/64 participants in the control group dropped out from the study before end-point.  3.2 Is there evidence that the result was not biased by missing outcome data? NI  3.3 Could missingness in the outcome depend on its true value? NI  3.4 Is it likely that missingness in the outcome depended on its true value? NI |
| Domain 4: Risk of bias in measurement of the outcome | Some concerns | 4.1 Was the method of measuring the outcome inappropriate? PN  4.2 Could measurement or ascertainment of the outcome have differed between intervention groups? N  4.3 Were outcome assessors aware of the intervention received by study participants? NI  Comment. Measures were self-reported.  4.4 Could assessment of the outcome have been influenced by knowledge of intervention received? PY  Comment. Participants were aware of their treatment allocation and judged the treatment outcome by means of a self-reported questionnaire. Participants allocated to the intervention group may have been more prone to judge favourably the treatment just knowing they had not been allocated to the WL condition.  4.5 Is it likely that assessment of the outcome was influenced by knowledge of intervention received? PN |
| Domain 5: Risk of bias in selection of the reported result | Some concerns | 5.1 Were the data that produced this result analysed in accordance with a pre-specified analysis plan that was finalized before unblinded outcome data were available for analysis? PN  Comment. No protocol available.  5.2 Is the numerical result being assessed likely to have been selected, on the basis of the results, from multiple eligible outcome measurements within the outcome domain? N  5.3 Is the numerical result being assessed likely to have been selected, on the basis of the results, from multiple eligible analyses of the data? PN |
| OVERALL RISK OF BIAS | High risk |  |

### Williams 1996

#### Risk of bias table

| **Bias** | **Authors' judgement** | **Support for judgement** |
| --- | --- | --- |
| Domain 1: Risk of bias arising from the randomization process | Some concerns | 1.1 Was the allocation sequence random? PY  1.2 Was the allocation sequence concealed until participants were enrolled and assigned to interventions? NI  Comment. Quote:"Subjects were randomly assigned to one of four treatment conditions".  1.3 Did baseline differences between intervention groups suggest a problem with the randomization process? PN  Comment. Quote:"one-way analyses of variance performed on all dependent and demographic measures at pretreatment revealed no significant differences among treatment groups." |
| Domain 2a: Risk of bias due to deviations from the intended interventions (effect of assignment to intervention) | Low risk | 2.6 Was an appropriate analysis used to estimate the effect of assignment to intervention? NI  Comment. No information provided.  2.7 Was there potential for a substantial impact (on the result) of the failure to analyse participants in the group to which they were randomized? PN  Comment. No participant withdrew from the study |
| Domain 2b: Risk of bias due to deviations from the intended interventions (effect of adhering to intervention) | Some concerns | 2.3 Were important non-protocol interventions balanced across intervention groups? NI  Comment. No information provided on the balancing of off-protocol medications or other off-protocol interventions across comparison groups.  2.4. Were there failures in implementing the intervention that could have affected the outcome? PN  2.5. Was there non-adherence to the assigned intervention regimen that could have affected participants’ outcomes? PN  2.6 Was an appropriate analysis used to estimate the effect of adhering to the intervention? NI  Comment. It is difficult to understand how the study authors dealt with data analysis. |
| Domain 3: Missing outcome data | Low risk | 3.1 Were data for this outcome available for all, or nearly all, participants randomized? Y  Comment. All patients were assessed at study end-point. |
| Domain 4: Risk of bias in measurement of the outcome | Some concerns | 4.1 Was the method of measuring the outcome inappropriate? PN  4.2 Could measurement or ascertainment of the outcome have differed between intervention groups? N  4.3 Were outcome assessors aware of the intervention received by study participants? NI  Comment. No information provided. The ACQ is a self-reported instrument.  4.4 Could assessment of the outcome have been influenced by knowledge of intervention received? PY  Comment. Participants were aware of their treatment allocation and judged the treatment outcome by means of a self-reported questionnaire. Participants allocated to the intervention group may have been more prone to judge favourably the treatment just knowing they had not been allocated to the WL condition.  4.5 Is it likely that assessment of the outcome was influenced by knowledge of intervention received? PN |
| Domain 5: Risk of bias in selection of the reported result | Some concerns | 5.1 Were the data that produced this result analysed in accordance with a pre-specified analysis plan that was finalized before unblinded outcome data were available for analysis? PN  Comment. No protocol available.  5.2 Is the numerical result being assessed likely to have been selected, on the basis of the results, from multiple eligible outcome measurements within the outcome domain? N  5.3 Is the numerical result being assessed likely to have been selected, on the basis of the results, from multiple eligible analyses of the data? PN |
| OVERALL RISK OF BIAS | High risk |  |

### Wims 2010

#### Risk of bias table

| **Bias** | **Authors' judgement** | **Support for judgement** |
| --- | --- | --- |
| Domain 1: Risk of bias arising from the randomization process | Some concerns | 1.1 Was the allocation sequence random? PY  1.2 Was the allocation sequence concealed until participants were enrolled and assigned to interventions? NI  Comment. Quote:"were randomized via a true randomization process (www.random.org)".  1.3 Did baseline differences between intervention groups suggest a problem with the randomization process? PN  Comment. Quote:"No between group differences in pre-treatment scores were observed using one way ANOVAs". |
| Domain 2a: Risk of bias due to deviations from the intended interventions (effect of assignment to intervention) | Low risk | 2.6 Was an appropriate analysis used to estimate the effect of assignment to intervention? Y  Comment. Quote:"All post-treatment analyses adopt an intention-to-treat (ITT) design." |
| Domain 2b: Risk of bias due to deviations from the intended interventions (effect of adhering to intervention) | Low risk | 2.3 Were important non-protocol interventions balanced across intervention groups? PY  Comment. off-protocol medication where balanced (table 1).  2.4. Were there failures in implementing the intervention that could have affected the outcome? PN  2.5. Was there non-adherence to the assigned intervention regimen that could have affected participants’ outcomes? PN |
| Domain 3: Missing outcome data | Some concerns | 3.1 Were data for this outcome available for all, or nearly all, participants randomized? PN  Comment. 10/32 and 5/27 participants withdrew from the study.  3.2 Is there evidence that the result was not biased by missing outcome data? NI  3.3 Could missingness in the outcome depend on its true value? NI  3.4 Is it likely that missingness in the outcome depended on its true value? NI |
| Domain 4: Risk of bias in measurement of the outcome | High risk | 4.1 Was the method of measuring the outcome inappropriate? N  4.2 Could measurement or ascertainment of the outcome have differed between intervention groups? N  4.3 Were outcome assessors aware of the intervention received by study participants? Y  Comment. Quote:"One psychiatry registrar (EW) provided all clinical contact with participants."  4.4 Could assessment of the outcome have been influenced by knowledge of intervention received? NI  4.5 Is it likely that assessment of the outcome was influenced by knowledge of intervention received? NI |
| Domain 5: Risk of bias in selection of the reported result | Some concerns | 5.1 Were the data that produced this result analysed in accordance with a pre-specified analysis plan that was finalized before unblinded outcome data were available for analysis? PN  Comment. No protocol available.  5.2 Is the numerical result being assessed likely to have been selected, on the basis of the results, from multiple eligible outcome measurements within the outcome domain? N  5.3 Is the numerical result being assessed likely to have been selected, on the basis of the results, from multiple eligible analyses of the data? PN |
| OVERALL RISK OF BIAS | High risk |  |

# Supplement K: Transitivity assessment and meta-regression

|  | HETEROGENEITY  P-value by treatment delivery formats | TRASITIVITY  P-value by set of diffent interventions | |
| --- | --- | --- | --- |
|  | meta-regression (association with outcome) | Kruskal-Wallis equality-of-populations rank test | Fisher exact test |
| Mean age | 0.221 | 0.533 |  |
| Percentage of women | 0.613 | 0.590 |  |
| Percentage of agoraphobics | 0.591 | 0.432 |  |
| number of psychotherapy sessions | 0.201 | 0.271 |  |
| provision of psychotherapy by specifically trained therapists | 0.801 |  | 0.267 |
| treatment integrity verification | 0.789 |  | 0.066 |
| treatment manual guidance | 0.996 |  | 0.593 |
| Type of outcome scale | 0.150 |  | 0.021 |

Age

Percentage of women

Percentage of agoraphobics

number of psychotherapy sessions

provision of psychotherapy by specifically trained therapists

treatment integrity verification

treatment manual guidance

Type of outcome scale

Most of the comparisons had similar mean age, percentage of women, number of psychotherapy sessions, provision of psychotherapy by specifically trained therapists, treatment manual guidance. There were a few comparisons which had relatively different percentages of agoraphobics and treatment integrity verification. The type of outcome measures was found to be generally balanced across all other comparisons, with preponderance of “scales specifically focused on panic disorder” over “global symptoms scales. The statistical imbalance signaled by the Fisher’s test (p = 0.021) is to be attributed to the presence of comparisons informed by one study only. This is the case for the ABC (individual VS group VS waiting list – Sharp, 2004), ABG (individual VS group VS placebo – Klosko, 1990), BH (individual VS psychological placebo – Loerch, 1999), and BE (individual VS unguided SH - Sharp, 2000) bars. The significance of the Fisher test is then to be interpreted as a statistical artifact, as the test does not adjust for the number of studies informing each comparison. Meta-regression of covariates did not show they had an impact on the network estimates.

**Supplement L - primary outcome: efficacy**

Outcome type: continuous

56 studies

**Treatment codes**

| Waiting list | WL | 1 | A |
| --- | --- | --- | --- |
| Individual | I | 2 | B |
| Group | G | 3 | C |
| Guided self-help | gSH | 4 | D |
| Unguided self-help | uSH | 5 | E |
| Treatment as usual | TAU | 6 | F |
| Placebo | PL | 7 | G |
| Psychological placebo | PP | 8 | h |

**Network map**

```
Pairwise meta-analysis


           Study     |     ES    [95% Conf. Interval]
---------------------+---------------------------------------------------
     F - B
Addis, 2004          |   -0.016    -0.455     0.423
Craske, 2011         |    0.345     0.081     0.610
Gensichen, 2020      |    0.206     0.013     0.399
Petterson, 1996      |    1.086     0.266     1.905
Pitti, 2015          |    0.549     0.072     1.026
Roy-Byrne, 2005      |    0.587     0.324     0.850
Roy-Byrne, 2010      |    0.214     0.033     0.394
 Sub-total           |
  D+L pooled ES      |    0.337     0.170     0.504
---------------------+---------------------------------------------------
     E - A
Allen, 2016          |   -0.972    -1.610    -0.335
Berger, 2017         |   -0.428    -0.850    -0.006
Ciuca, 2018          |   -0.815    -1.281    -0.349
Gould, 1993          |   -0.390    -1.233     0.452
Hazen 1996           |   -0.802    -1.276    -0.327
Lidren, 1994         |   -1.648    -2.547    -0.750
Nordin, 2010         |   -1.490    -2.198    -0.782
Oromendia, 2016      |   -0.789    -1.348    -0.230
 Sub-total           |
  D+L pooled ES      |   -0.848    -1.111    -0.585
---------------------+---------------------------------------------------
     G - B
Bakker, 1999         |   -0.140    -0.620     0.340
Barlow, 2000         |    0.483     0.020     0.947
Klosko, 1990         |    0.535    -0.253     1.322
Sharp, 1997          |    1.025     0.475     1.576
Shear, 2001          |    0.429    -0.101     0.958
 Sub-total           |
  D+L pooled ES      |    0.449     0.063     0.836
---------------------+---------------------------------------------------
     B - A
Barlow, 1989         |   -0.929    -1.576    -0.281
Beck, 1992           |   -0.643    -1.345     0.059
Botella, 2007        |   -1.694    -2.501    -0.887
Clark, 1999          |   -2.199    -3.008    -1.390
Craske, 2005         |   -1.016    -1.734    -0.299
Gloster, 2011        |   -0.832    -1.102    -0.562
Gould, 1993          |    0.374    -0.512     1.261
Hendriks, 2010       |   -0.605    -1.338     0.127
Kenardy, 2003        |   -1.258    -1.638    -0.878
Klosko, 1990         |   -1.350    -2.130    -0.570
Ost, 2004            |   -0.888    -1.486    -0.291
Reinecke, 2013       |   -0.447    -1.198     0.305
Sharp, 2004          |   -1.435    -2.057    -0.813
Williams, 1996       |   -1.342    -2.297    -0.387
 Sub-total           |
  D+L pooled ES      |   -1.019    -1.280    -0.758
---------------------+---------------------------------------------------
     D - C
Bergstrom, 2010      |    0.000    -0.407     0.407
 Sub-total           |
  D+L pooled ES      |    0.000    -0.407     0.407
---------------------+---------------------------------------------------
     D - A
Carlbring, 2006      |   -1.943    -2.563    -1.322
Carlbring, 2001      |   -1.415    -2.106    -0.723
Ciuca, 2018          |   -1.218    -1.702    -0.733
Oromendia, 2016      |   -1.725    -2.346    -1.103
Ruwaard, 2010        |   -0.547    -1.073    -0.021
Silfvernagel, 2012   |   -1.395    -1.978    -0.812
Titov, 2010          |   -1.254    -2.207    -0.301
van Ballegooijen, 20 |   -0.304    -0.656     0.047
Wims, 2010           |   -0.586    -1.133    -0.039
 Sub-total           |
  D+L pooled ES      |   -1.123    -1.533    -0.714
---------------------+---------------------------------------------------
     D - B
Carlbring, 2005      |    0.047    -0.513     0.607
Kiropoulos, 2008     |    0.116    -0.326     0.559
 Sub-total           |
  D+L pooled ES      |    0.090    -0.257     0.437
---------------------+---------------------------------------------------
     C - A
Carter, 2003         |   -1.811    -2.770    -0.852
Erickson, 2007       |   -0.306    -1.169     0.556
Hazen 1996           |   -1.387    -1.958    -0.816
Lidren, 1994         |   -1.473    -2.353    -0.593
Meulenbeek, 2010     |   -0.675    -0.948    -0.401
Schmidt, 1997b       |   -2.262    -3.078    -1.446
Sharp, 2004          |   -0.641    -1.278    -0.003
Telch, 1993          |   -1.825    -2.400    -1.250
 Sub-total           |
  D+L pooled ES      |   -1.266    -1.736    -0.797
---------------------+---------------------------------------------------
     E - D
Ciuca, 2018          |    0.402    -0.060     0.865
Fogliati, 2016       |   -0.215    -0.557     0.127
Oromendia, 2016      |    0.935     0.370     1.500
 Sub-total           |
  D+L pooled ES      |    0.345    -0.314     1.004
---------------------+---------------------------------------------------
     E - B
Gould, 1993          |   -0.764    -1.669     0.140
Sharp, 2000          |    0.712     0.259     1.166
 Sub-total           |
  D+L pooled ES      |    0.028    -1.415     1.471
---------------------+---------------------------------------------------
     E - C
Hazen 1996           |    0.585     0.110     1.060
Lidren, 1994         |   -0.175    -0.977     0.626
 Sub-total           |
  D+L pooled ES      |    0.276    -0.456     1.008
---------------------+---------------------------------------------------
     F - D
Klein, 2006          |    0.580     0.006     1.155
Richards, 2006       |    0.452    -0.328     1.232
 Sub-total           |
  D+L pooled ES      |    0.535     0.073     0.998
---------------------+---------------------------------------------------
     G - A
Klosko, 1990         |   -0.815    -1.616    -0.015
 Sub-total           |
  D+L pooled ES      |   -0.815    -1.616    -0.015
---------------------+---------------------------------------------------
     F - E
Koszycki, 2011       |    0.456     0.030     0.882
 Sub-total           |
  D+L pooled ES      |    0.456     0.030     0.882
---------------------+---------------------------------------------------
     H - B
Loerch, 1999         |    0.831     0.001     1.661
 Sub-total           |
  D+L pooled ES      |    0.831     0.001     1.661
---------------------+---------------------------------------------------
     H - C
Marchand, 2008       |    0.386    -0.122     0.893
 Sub-total           |
  D+L pooled ES      |    0.386    -0.122     0.893
---------------------+---------------------------------------------------
     C - B
Roberge, 2008        |   -0.143    -0.555     0.268
Sharp, 2004          |    0.794     0.216     1.373
 Sub-total           |
  D+L pooled ES      |    0.303    -0.615     1.220
---------------------+---------------------------------------------------

Test(s) of heterogeneity:
               Heterogeneity  degrees of
                 statistic     freedom      P    I-squared**   Tau-squared

F - B               13.06          6      0.042     54.0%       0.0243
E - A               11.24          7      0.128     37.7%       0.0523
G - B               10.03          4      0.040     60.1%       0.1149
B - A               31.28         13      0.003     58.4%       0.1301
D - C                0.00          0         .         .%       0.0000
D - A               38.25          8      0.000     79.1%       0.3004
D - B                0.04          1      0.849      0.0%       0.0000
C - A               31.74          7      0.000     77.9%       0.3320
E - D               12.87          2      0.002     84.5%       0.2841
E - B                8.18          1      0.004     87.8%       0.9571
E - C                2.56          1      0.110     60.9%       0.1761
F - D                0.07          1      0.795      0.0%       0.0000
G - A                0.00          0         .         .%       0.0000
F - E                0.00          0         .         .%       0.0000
H - B                0.00          0         .         .%       0.0000
H - C                0.00          0         .         .%       0.0000
C - B                6.70          1      0.010     85.1%       0.3737
** I-squared: the variation in ES attributable to heterogeneity)


Significance test(s) of ES=0

F - B                 z=  3.95     p = 0.000
E - A                 z=  6.33     p = 0.000
G - B                 z=  2.28     p = 0.023
B - A                 z=  7.66     p = 0.000
D - C                 z=  0.00     p = 1.000
D - A                 z=  5.37     p = 0.000
D - B                 z=  0.51     p = 0.612
C - A                 z=  5.29     p = 0.000
E - D                 z=  1.03     p = 0.305
E - B                 z=  0.04     p = 0.970
E - C                 z=  0.74     p = 0.459
F - D                 z=  2.27     p = 0.023
G - A                 z=  2.00     p = 0.046
F - E                 z=  2.10     p = 0.036
H - B                 z=  1.96     p = 0.050
H - C                 z=  1.49     p = 0.136
C - B                 z=  0.65     p = 0.518
-------------------------------------------------------------------------

Interval plot
```

**Net league table**

| G | 0.05 (-0.27,0.37) | 0.05 (-0.30,0.39) | 0.26 (-0.10,0.62) | 0.47 (0.07,0.87) | 0.48 (-0.03,0.98) | 0.58 (-0.12,1.27) | 1.13 (0.84,1.42) |
| --- | --- | --- | --- | --- | --- | --- | --- |
| -0.05 (-0.37,0.27) | I | 0.00 (-0.28,0.28) | 0.21 (-0.09,0.52) | 0.43 (0.15,0.70) | 0.43 (0.03,0.83) | 0.53 (-0.18,1.24) | 1.08 (0.87,1.30) |
| -0.05 (-0.39,0.30) | -0.00 (-0.28,0.28) | gSH | 0.21 (-0.10,0.52) | 0.42 (0.07,0.77) | 0.43 (-0.05,0.91) | 0.53 (-0.21,1.27) | 1.08 (0.83,1.33) |
| -0.26 (-0.62,0.10) | -0.21 (-0.52,0.09) | -0.21 (-0.52,0.10) | uSH | 0.21 (-0.16,0.58) | 0.22 (-0.28,0.71) | 0.32 (-0.43,1.06) | 0.87 (0.60,1.14) |
| **-0.47 (-0.87,-0.07)** | **-0.43 (-0.70,-0.15)** | **-0.42 (-0.77,-0.07)** | -0.21 (-0.58,0.16) | TAU | 0.00 (-0.47,0.48) | 0.10 (-0.65,0.86) | 0.66 (0.33,0.98) |
| -0.48 (-0.98,0.03) | **-0.43 (-0.83,-0.03)** | -0.43 (-0.91,0.05) | -0.22 (-0.71,0.28) | -0.00 (-0.48,0.47) | PL | 0.10 (-0.71,0.91) | 0.65 (0.21,1.10) |
| -0.58 (-1.27,0.12) | -0.53 (-1.24,0.18) | -0.53 (-1.27,0.21) | -0.32 (-1.06,0.43) | -0.10 (-0.86,0.65) | -0.10 (-0.91,0.71) | PP | 0.55 (-0.16,1.27) |
| **-1.13 (-1.42,-0.84)** | **-1.08 (-1.30,-0.87)** | **-1.08 (-1.33,-0.83)** | **-0.87 (-1.14,-0.60)** | **-0.66 (-0.98,-0.33)** | **-0.65 (-1.10,-0.21)** | -0.55 (-1.27,0.16) | WL |

```
Net league table: head-to-head comparisons. Effect sizes (ES) and 95% confidence intervals (CIs) are reported. Negative ES favour the column-defining treatment. 95% CIs not including the point of no difference (0) are in boldface.

Evaluation of heterogeneity and incoherence

Overall heterogeneity
SD=0.36; Restricted likelihood ratio test for heterogeneity: LRT = 41.28 (d.f. = 1) P = 0.000
Overall incoherence
chi2( 45) =   15.97
Prob > chi2 =    0.5950

Loop-specific approach

+-----------------------------------------------------------------------------+
  |  Loop |    IF |  seIF | z_value | p_value |       CI_95 | Loop_Heterog_tau2 |
  |-------+-------+-------+---------+---------+-------------+-------------------|
  | A-B-C | 0.559 | 0.490 |   1.140 |   0.254 | (0.00,1.52) |             0.217 |
  | B-C-E | 0.474 | 0.942 |   0.503 |   0.615 | (0.00,2.32) |             0.457 |
  | B-E-F | 0.405 | 0.548 |   0.739 |   0.460 | (0.00,1.48) |             0.048 |
  | B-D-E | 0.311 | 0.748 |   0.417 |   0.677 | (0.00,1.78) |             0.281 |
  | B-D-F | 0.291 | 0.344 |   0.845 |   0.398 | (0.00,0.96) |             0.016 |
  | B-C-H | 0.273 | 0.525 |   0.520 |   0.603 | (0.00,1.30) |             0.000 |
  | D-E-F | 0.270 | 0.719 |   0.375 |   0.708 | (0.00,1.68) |             0.214 |
  | A-B-G | 0.269 | 0.660 |   0.407 |   0.684 | (0.00,1.56) |             0.132 |
  | A-D-E | 0.207 | 0.407 |   0.509 |   0.611 | (0.00,1.01) |             0.203 |
  | B-C-D | 0.196 | 0.648 |   0.302 |   0.762 | (0.00,1.47) |             0.156 |
  | A-C-E | 0.176 | 0.496 |   0.355 |   0.722 | (0.00,1.15) |             0.203 |
  | A-B-D | 0.174 | 0.436 |   0.399 |   0.690 | (0.00,1.03) |             0.186 |
  | A-C-D | 0.140 | 0.676 |   0.207 |   0.836 | (0.00,1.47) |             0.314 |
  | C-D-E | 0.083 | 0.773 |   0.107 |   0.914 | (0.00,1.60) |             0.264 |
  | A-B-E | 0.003 | 0.398 |   0.008 |   0.994 | (0.00,0.78) |             0.095 |
  +-----------------------------------------------------------------------------+
```

```
Consistency between direct and indirect estimates
Side    Direct                Indirect              Difference
        Coef.      Std. Err.  Coef.      Std. Err.  Coef.      Std. Err.  P>|z|
A B     -1.022414   .1350313   -1.21964   .2009831   .1972264   .2421461  0.415
A C     -1.228389   .1786247  -.9149542   .2670029  -.3134346   .3225249  0.331
A D     -1.098813    .159157  -1.054725    .210439   -.044088   .2632889  0.867
A E     -.8622711   .1714787   -.887868    .245175    .025597   .3003524  0.932
A G      -.816423   .5499204  -.6196372   .2494129  -.1967858   .6036372  0.744
B C      .2743533   .3137563  -.1681771   .1925688   .4425304   .3683232  0.230
B D      .0843913   .3181813  -.0205006   .1634613    .104892    .357699  0.769
B E      .2052962    .353329   .2155567   .1757887  -.0102605   .3949485  0.979
B F      .3739006   .1557705   .6273984   .3047834  -.2534978   .3426352  0.459
B G *    .4509182    .207561  -.0228271    .972432   .4737453   .9930944  0.633
B H      .8309899   .5605322   .3113679   .4800814   .5196221   .7380191  0.481
C D     -1.29e-06   .4254334    .059597    .196056  -.0595983   .4684352  0.899
C E      .2874255   .3446884    .250805    .218352   .0366204   .4080557  0.928
C H      .3858921   .4493032   .9059608   .5854754  -.5200687    .738007  0.481
D E      .3178752   .2528703   .1390787   .2098895   .1787966    .329021  0.587
D F      .5251907   .3555626   .3908405   .2091645   .1343502   .4125863  0.745
E F      .4558424   .4266162   .1528757   .2125874   .3029666   .4766495  0.525

* Warning: all the evidence about these contrasts comes from the trials which directly compare them.

SUCRA and cumulative probability plots


Estimated probabilities (%) of each treatment being the best (and other ranks)
- assuming the minimum parameter is the best
- using 5000 draws
- allowing for parameter uncertainty
----------------------------------------------------------
trial_cod |
e and     |                   Treatment
Rank      |    1     2     3     4     5     6     7     8
----------+-----------------------------------------------
1         |
     Best |  0.0  24.0  44.6  25.7   1.0   0.0   0.8   3.8
      2nd |  0.0  35.1  26.0  30.6   4.3   0.2   1.0   2.8
      3rd |  0.0  31.8  20.3  29.7  11.0   0.8   3.1   3.4
      4th |  0.0   8.4   7.4  11.4  47.4   6.8  10.1   8.6
      5th |  0.0   0.8   1.5   2.3  25.7  30.6  24.1  15.0
      6th |  0.0   0.0   0.2   0.2   8.9  40.7  33.7  16.3
      7th |  6.3   0.0   0.0   0.1   1.8  20.9  27.0  44.0
    Worst | 93.7   0.0   0.0   0.0   0.0   0.0   0.2   6.1
MEAN RANK |  7.9   2.3   2.0   2.3   4.3   5.7   5.7   5.8
    SUCRA |  0.0   0.8   0.9   0.8   0.5   0.3   0.3   0.3
----------------------------------------------------------

Treatment Relative Ranking of Model 1


  +--------------------------------------+
  | Treatmet | SUCRA | PrBest | MeanRank |
  |----------+-------+--------+----------|
  |       WL |   0.9 |    0.0 |      7.9 |
  |        I |  81.9 |   24.0 |      2.3 |
  |        G |  86.3 |   44.6 |      2.0 |
  |      gSH |  80.7 |   25.7 |      2.3 |
  |      uSH |  53.4 |    1.0 |      4.3 |
  |      TAU |  32.4 |    0.0 |      5.7 |
  |       PL |  33.5 |    0.8 |      5.7 |
  |       PP |  30.9 |    3.8 |      5.8 |
  +--------------------------------------+
```

**Funnel plot**

One comparison with 10 one more studies: WL-I (14 studies).

1) WL-I (14 studies)

```
Egger's test for small-study effects:
Regress standard normal deviate of intervention
effect estimate against its standard error

Number of studies =  14                                Root MSE      =   1.607
------------------------------------------------------------------------------
     Std_Eff |      Coef.   Std. Err.      t    P>|t|     [95% Conf. Interval]
-------------+----------------------------------------------------------------
       slope |  -.8998469   .3194401    -2.82   0.016    -1.595847   -.2038468
        bias |  -.3591176   1.098546    -0.33   0.749    -2.752644    2.034408
------------------------------------------------------------------------------

Test of H0: no small-study effects          P = 0.749


GRADE appraisal (CINeMA)

The analysis of the certainty of the evidence was performed with the online application CINeMA, which follows the principles of the GRADE methodology. The following criteria were applied:
```

- Within-study bias: the “overall” risk of bias of each study was calculated as follows: (a) LOW risk if there “some concerns” on max two domains of the Cochrane RoB 2; (b) SOME CONCERNS if three domains were judged as having some concerns OR one domain was considered at high risk and two leaving some concerns; (c) HIGH RISK in all other cases (two or more high risk domains; three domains leaving some concerns + one high risk domain; 4 or more domains leaving some concerns). For each comparison, the histogram was interpreted according to a “Average risk of bias” rule;
- Across-studies bias was considered “undetected” when was not possible to evaluate the risk of publication bias;
- Imprecision: an effect size of 0.3 was considered as clinically important;
- Heterogeneity: an effect size of 0.3 was considered as clinically important;
- Incoherence: for all the comparisons for which only a direct or indirect estimation was available (Inconsistency measures: Not applicable) we reported “some concern”.

Final report

| Comparison | Number of studies | Within-study bias | Reporting bias | Indirectness | Imprecision | Heterogeneity | Incoherence | Confidence rating |
| --- | --- | --- | --- | --- | --- | --- | --- | --- |
| face-to-face group:face-to-face individual | 2 | Some concerns | Low risk | No concerns | Some concerns | Some concerns | No concerns | Low |
| face-to-face group:guided self-help | 1 | Some concerns | Low risk | No concerns | Some concerns | Some concerns | No concerns | Low |
| face-to-face group:psychological placebo | 1 | Major concerns | Low risk | No concerns | Some concerns | Some concerns | No concerns | Very low |
| face-to-face group:unguided self-help | 2 | Some concerns | Low risk | No concerns | Some concerns | Some concerns | No concerns | Low |
| face-to-face group:waiting list | 8 | Some concerns | Low risk | No concerns | No concerns | No concerns | No concerns | Moderate |
| face-to-face individual:guided self-help | 2 | Some concerns | Low risk | No concerns | No concerns | Major concerns | No concerns | Low |
| face-to-face individual:placebo pill | 5 | Some concerns | Low risk | No concerns | No concerns | Major concerns | No concerns | Low |
| face-to-face individual:psychological placebo | 1 | Major concerns | Low risk | No concerns | Some concerns | Some concerns | No concerns | Very low |
| face-to-face individual:treatment as usual | 7 | Some concerns | Low risk | No concerns | No concerns | Some concerns | No concerns | Moderate |
| face-to-face individual:unguided self-help | 2 | Some concerns | Low risk | No concerns | Some concerns | Some concerns | No concerns | Low |
| face-to-face individual:waiting list | 14 | Some concerns | Low risk | No concerns | No concerns | No concerns | No concerns | Moderate |
| guided self-help:treatment as usual | 2 | Some concerns | Low risk | No concerns | No concerns | Major concerns | No concerns | Low |
| guided self-help:unguided self-help | 3 | Some concerns | Low risk | No concerns | Some concerns | Some concerns | No concerns | Low |
| guided self-help:waiting list | 9 | Some concerns | Low risk | No concerns | No concerns | No concerns | No concerns | Moderate |
| placebo pill:waiting list | 1 | Some concerns | Low risk | No concerns | No concerns | Some concerns | No concerns | Moderate |
| treatment as usual:unguided self-help | 1 | Some concerns | Low risk | No concerns | Some concerns | Some concerns | No concerns | Low |
| unguided self-help:waiting list | 8 | Some concerns | Low risk | No concerns | No concerns | No concerns | No concerns | Moderate |
| face-to-face group:placebo pill | 0 | Some concerns | Low risk | No concerns | Some concerns | Some concerns | No concerns | Low |
| face-to-face group:treatment as usual | 0 | Some concerns | Low risk | No concerns | No concerns | Some concerns | No concerns | Moderate |
| guided self-help:placebo pill | 0 | Some concerns | Low risk | No concerns | Some concerns | Some concerns | No concerns | Low |
| guided self-help:psychological placebo | 0 | Major concerns | Low risk | No concerns | Some concerns | Some concerns | No concerns | Very low |
| placebo pill:psychological placebo | 0 | Major concerns | Low risk | No concerns | Major concerns | No concerns | No concerns | Very low |
| placebo pill:treatment as usual | 0 | Some concerns | Low risk | No concerns | Major concerns | No concerns | No concerns | Low |
| placebo pill:unguided self-help | 0 | Some concerns | Low risk | No concerns | Some concerns | Some concerns | No concerns | Low |
| psychological placebo:treatment as usual | 0 | Major concerns | Low risk | No concerns | Major concerns | No concerns | No concerns | Very low |
| psychological placebo:unguided self-help | 0 | Major concerns | Low risk | No concerns | Major concerns | No concerns | No concerns | Very low |
| psychological placebo:waiting list | 0 | Major concerns | Low risk | No concerns | Some concerns | Some concerns | No concerns | Very low |
| treatment as usual:waiting list | 0 | Some concerns | Low risk | No concerns | No concerns | Some concerns | No concerns | Moderate |

**Supplement M - primary outcome: acceptability**

Outcome type: binary

51 studies

**Treatment codes**

| Waiting list | WL | 1 | A |
| --- | --- | --- | --- |
| Individual | I | 2 | B |
| Group | G | 3 | C |
| Guided self-help | gSH | 4 | D |
| Unguided self-help | uSH | 5 | E |
| Treatment as usual | TAU | 6 | F |
| Placebo | PL | 7 | G |
| Psychological placebo | PP | 8 | H |

**Network map**

```
Pairwise meta-analysis


       Study     |     ES    [95% Conf. Interval]
---------------------+---------------------------------------------------
     F - B
Addis, 2004          |    0.452     0.170     1.205
Craske, 2011         |    1.482     0.815     2.695
Gensichen, 2020      |    0.575     0.376     0.880
Pitti, 2015          |    0.054     0.003     0.869
Roy-Byrne, 2005      |    0.872     0.542     1.402
 Sub-total           |
  D+L pooled ES      |    0.725     0.431     1.221
---------------------+---------------------------------------------------
     E - A
Allen, 2016          |    3.700     0.405    33.774
Berger, 2017         |    1.367     0.485     3.854
Ciuca, 2018          |    1.120     0.567     2.215
Gould, 1993          |    1.000     0.119     8.403
Lidren, 1994         |    1.000     0.021    46.703
Nordin, 2010         |    0.333     0.014     7.724
Oromendia, 2016      |   15.786     0.958   260.050
 Sub-total           |
  D+L pooled ES      |    1.315     0.788     2.196
---------------------+---------------------------------------------------
     G - B
Bakker, 1999         |    0.243     0.057     1.042
Barlow, 2000         |    1.528     0.840     2.777
Klosko, 1990         |    2.333     0.714     7.626
Shear, 2001          |    1.006     0.523     1.935
 Sub-total           |
  D+L pooled ES      |    1.109     0.573     2.146
---------------------+---------------------------------------------------
     B - A
Barlow, 1989         |    2.000     0.253    15.807
Beck, 1992           |    0.944     0.020    44.985
Botella, 2007        |    0.520     0.011    24.737
Clark, 1999          |    1.500     0.065    34.657
Craske, 2005         |    4.250     0.234    77.338
Gloster, 2011        |    1.130     0.399     3.198
Gould, 1993          |    0.433     0.020     9.548
Hendriks, 2010       |    1.857     0.082    42.267
Klosko, 1990         |    2.667     0.307    23.138
Ost, 2004            |    0.846     0.130     5.523
Reinecke, 2013       |    1.000     0.021    47.183
Sharp, 2004          |    1.189     0.330     4.284
Williams, 1996       |    0.714     0.015    33.067
 Sub-total           |
  D+L pooled ES      |    1.250     0.699     2.235
---------------------+---------------------------------------------------
     D - C
Bergstrom, 2010      |    0.926     0.416     2.061
 Sub-total           |
  D+L pooled ES      |    0.926     0.416     2.061
---------------------+---------------------------------------------------
     D - A
Carlbring, 2006      |    2.000     0.191    20.898
Carlbring, 2001      |    3.810     0.465    31.234
Ciuca, 2018          |    0.672     0.293     1.542
Oromendia, 2016      |    5.000     0.252    99.161
Ruwaard, 2010        |    3.444     0.380    31.198
Silfvernagel, 2012   |    4.828     1.159    20.105
Titov, 2010          |    1.091     0.024    50.431
van Ballegooijen, 20 |    1.208     0.800     1.826
Wims, 2010           |    1.688     0.657     4.335
 Sub-total           |
  D+L pooled ES      |    1.390     0.947     2.041
---------------------+---------------------------------------------------
     D - B
Carlbring, 2005      |    0.960     0.214     4.298
Kiropoulos, 2008     |    2.875     0.590    14.016
 Sub-total           |
  D+L pooled ES      |    1.612     0.543     4.789
---------------------+---------------------------------------------------
     C - A
Carter, 2003         |    0.662     0.176     2.493
Erickson, 2007       |    0.875     0.403     1.901
Lidren, 1994         |    1.000     0.021    46.703
Meulenbeek, 2010     |    1.288     0.590     2.811
Schmidt, 1997b       |    0.421     0.120     1.480
Sharp, 2004          |    3.474     1.152    10.474
Telch, 1993          |    0.971     0.020    47.581
 Sub-total           |
  D+L pooled ES      |    1.073     0.645     1.786
---------------------+---------------------------------------------------
     E - D
Ciuca, 2018          |    1.668     0.741     3.755
Fogliati, 2016       |    0.986     0.143     6.814
Oromendia, 2016      |    3.157     0.856    11.645
 Sub-total           |
  D+L pooled ES      |    1.841     0.962     3.523
---------------------+---------------------------------------------------
     E - B
Gould, 1993          |    2.308     0.105    50.849
Sharp, 2000          |    1.641     0.597     4.509
 Sub-total           |
  D+L pooled ES      |    1.696     0.649     4.433
---------------------+---------------------------------------------------
     F - E
Klein, 2001          |    2.769     0.124    61.653
Koszycki, 2011       |    0.996     0.591     1.680
 Sub-total           |
  D+L pooled ES      |    1.024     0.612     1.715
---------------------+---------------------------------------------------
     F - D
Klein, 2006          |    0.222     0.013     3.917
Richards, 2006       |    1.704     0.339     8.564
 Sub-total           |
  D+L pooled ES      |    0.881     0.136     5.706
---------------------+---------------------------------------------------
     G - A
Klosko, 1990         |    6.222     0.856    45.252
 Sub-total           |
  D+L pooled ES      |    6.222     0.856    45.252
---------------------+---------------------------------------------------
     E - C
Lidren, 1994         |    1.000     0.021    46.703
 Sub-total           |
  D+L pooled ES      |    1.000     0.021    46.703
---------------------+---------------------------------------------------
     H - B
Loerch, 1999         |    2.545     0.264    24.561
 Sub-total           |
  D+L pooled ES      |    2.545     0.264    24.561
---------------------+---------------------------------------------------
     C - B
Roberge, 2008        |    0.844     0.319     2.236
Sharp, 2004          |    2.921     1.305     6.536
 Sub-total           |
  D+L pooled ES      |    1.620     0.481     5.461
---------------------+---------------------------------------------------

Test(s) of heterogeneity:
               Heterogeneity  degrees of
                 statistic     freedom      P    I-squared**   Tau-squared

F - B               11.32          4      0.023     64.7%       0.1969
E - A                4.90          6      0.557      0.0%       0.0000
G - B                6.74          3      0.081     55.5%       0.2374
B - A                2.40         12      0.998      0.0%       0.0000
D - C                0.00          0         .         .%       0.0000
D - A                8.74          8      0.364      8.5%       0.0334
D - B                0.97          1      0.324      0.0%       0.0000
C - A                7.47          6      0.280     19.7%       0.0910
E - D                1.11          2      0.573      0.0%       0.0000
E - B                0.04          1      0.837      0.0%       0.0000
F - E                0.41          1      0.524      0.0%       0.0000
F - D                1.47          1      0.225     32.0%       0.6634
G - A                0.00          0         .         .%       0.0000
E - C                0.00          0         .         .%       0.0000
H - B                0.00          0         .         .%       0.0000
C - B                3.71          1      0.054     73.0%       0.5626
** I-squared: the variation in ES attributable to heterogeneity)


Significance test(s) of ES=1

F - B                 z=  1.21     p = 0.227
E - A                 z=  1.05     p = 0.295
G - B                 z=  0.31     p = 0.758
B - A                 z=  0.75     p = 0.451
D - C                 z=  0.19     p = 0.851
D - A                 z=  1.68     p = 0.092
D - B                 z=  0.86     p = 0.390
C - A                 z=  0.27     p = 0.786
E - D                 z=  1.84     p = 0.065
E - B                 z=  1.08     p = 0.281
F - E                 z=  0.09     p = 0.927
F - D                 z=  0.13     p = 0.895
G - A                 z=  1.81     p = 0.071
E - C                 z=  0.00     p = 1.000
H - B                 z=  0.81     p = 0.419
C - B                 z=  0.78     p = 0.436
-------------------------------------------------------------------------
Interval plot
```

**Net League**

| G | 0.94 (0.56,1.57) | 1.05 (0.62,1.77) | 1.19 (0.65,2.16) | 0.81 (0.45,1.48) | 1.14 (0.55,2.36) | 2.39 (0.22,26.52) | 0.79 (0.51,1.21) |
| --- | --- | --- | --- | --- | --- | --- | --- |
| 1.07 (0.64,1.78) | I | 1.12 (0.68,1.84) | 1.27 (0.76,2.10) | 0.87 (0.59,1.27) | 1.22 (0.72,2.06) | 2.55 (0.24,26.72) | 0.84 (0.54,1.30) |
| 0.95 (0.56,1.61) | 0.89 (0.54,1.47) | gSH | 1.13 (0.68,1.89) | 0.78 (0.44,1.36) | 1.09 (0.53,2.23) | 2.28 (0.21,25.17) | 0.75 (0.50,1.12) |
| 0.84 (0.46,1.53) | 0.79 (0.48,1.31) | 0.88 (0.53,1.48) | uSH | 0.69 (0.40,1.17) | 0.96 (0.47,1.99) | 2.01 (0.18,22.29) | 0.66 (0.41,1.07) |
| 1.23 (0.67,2.24) | 1.15 (0.79,1.69) | 1.29 (0.73,2.26) | 1.46 (0.86,2.48) | TAU | 1.41 (0.74,2.68) | 2.93 (0.27,31.76) | 0.97 (0.57,1.63) |
| 0.87 (0.42,1.80) | 0.82 (0.49,1.39) | 0.92 (0.45,1.88) | 1.04 (0.50,2.14) | 0.71 (0.37,1.36) | PL | 2.09 (0.19,23.23) | 0.69 (0.35,1.34) |
| 0.42 (0.04,4.64) | 0.39 (0.04,4.12) | 0.44 (0.04,4.86) | 0.50 (0.04,5.51) | 0.34 (0.03,3.69) | 0.48 (0.04,5.33) | PP | 0.33 (0.03,3.60) |
| 1.27 (0.83,1.95) | 1.19 (0.77,1.85) | 1.33 (0.90,1.99) | 1.51 (0.93,2.44) | 1.03 (0.61,1.75) | 1.45 (0.74,2.84) | 3.04 (0.28,33.19) | WL |

```
Net league table: head-to-head comparisons. Effect sizes (ES) and 95% confidence intervals (CIs) are reported. Negative ES favour the column-defining treatment. 95% CIs not including the point of no difference (0) are in boldface.

Evaluation of heterogeneity and incoherence

Overall heterogeneity
SD=0.31; Restricted likelihood ratio test for heterogeneity: LRT =   3.23 (d.f. = 1) P = 0.034
Overall incoherence
chi2( 43) =   16.13
Prob > chi2 =  0.5144

Loop-specific approach
* 14 triangular loops found

 Evaluation of inconsistency using loop-specific heterogeneity estimates:

+----------------------------------------------------------------------+
  |  Loop |   ROR | z_value | p_value |        CI_95 | Loop_Heterog_tau2 |
  |-------+-------+---------+---------+--------------+-------------------|
  | A-B-G | 4.411 |   1.377 |   0.168 | (1.00,36.44) |             0.000 |
  | B-E-F | 2.512 |   1.112 |   0.266 | (1.00,12.74) |             0.149 |
  | B-D-F | 2.190 |   0.655 |   0.512 | (1.00,22.85) |             0.196 |
  | A-B-C | 2.065 |   1.425 |   0.154 |  (1.00,5.59) |             0.000 |
  | A-D-E | 2.061 |   1.578 |   0.115 |  (1.00,5.06) |             0.000 |
  | D-E-F | 1.807 |   0.710 |   0.478 |  (1.00,9.25) |             0.000 |
  | B-D-E | 1.750 |   0.689 |   0.491 |  (1.00,8.59) |             0.000 |
  | C-D-E | 1.705 |   0.263 |   0.793 | (1.00,91.19) |             0.000 |
  | A-B-E | 1.675 |   0.816 |   0.414 |  (1.00,5.79) |             0.000 |
  | A-B-D | 1.516 |   0.640 |   0.522 |  (1.00,5.43) |             0.000 |
  | A-C-D | 1.432 |   0.625 |   0.532 |  (1.00,4.41) |             0.058 |
  | A-C-E | 1.259 |   0.114 |   0.909 | (1.00,65.95) |             0.055 |
  | B-C-D | 1.079 |   0.064 |   0.949 | (1.00,11.08) |             0.417 |
  | B-C-E | 1.058 |   0.025 |   0.980 | (1.00,81.18) |             0.316 |
  +----------------------------------------------------------------------+
```

```
Consistency between direct and indirect estimates

Side    Direct                Indirect              Difference
        Coef.      Std. Err.  Coef.      Std. Err.  Coef.      Std. Err.  P>|z|
A B      .1027571   .3209735   .2506354   .3138556  -.1478783    .444433  0.739
A C      .1318185     .26743   .5033101   .4281787  -.3714916    .513251  0.469
A D      .3537884   .2480588     .14349   .3834628   .2102984   .4546865  0.644
A E      .4076408   .3225636   .4354766   .4013661  -.0278358    .517842  0.957
A G      1.819958   .9172961   .1212304     .37319   1.698728   1.002896  0.090
B C      .5921236    .366233  -.3576141   .3353054   .9497377   .4959906  0.056
B D      .4817197   .6005359   .0316346   .2812814   .4500851   .6630842  0.497
B E      .4926994   .5659483   .1686324   .2941033    .324067   .6385049  0.612
B F     -.2837235   .2079641    .505864   .4402115  -.7895874   .4865564  0.105
B G *     .138777   .2710066   2.866268   2.132251  -2.727492   2.162408  0.207
B H *    .9343092   1.199594  -.4561249   171.2606   1.390434   171.2678  0.994
C D     -.0766175    .532402   .1043563   .3186992  -.1809739   .6205007  0.771
C E      8.93e-12   1.987189   .1773478   .3106465  -.1773478   2.011323  0.930
D E      .6137502   .3846269  -.2858676   .3494363   .8996178   .5241078  0.086
D F      .0059198   .7633233  -.2987752   .3130268   .3046949   .8238701  0.712
E F      .0559741   .3914183  -.7328822    .357319   .7888563   .5305158  0.137


SUCRA and cumulative probability plots

Estimated probabilities (%) of each treatment being the best (and other ranks)
- assuming the minimum parameter is the best
- using 5000 draws
- allowing for parameter uncertainty
----------------------------------------------------------
trial_cod |
e and     |                   Treatment
Rank      |    1     2     3     4     5     6     7     8
----------+-----------------------------------------------
1         |
     Best | 17.4   1.1   1.0   0.9  40.6  27.6   2.0   9.5
      2nd | 32.3  13.7   1.4   3.9   9.6  31.9   1.6   5.6
      3rd | 27.8  25.1   1.8   9.1   6.8  22.8   1.8   4.8
      4th | 16.7  31.2   4.3  19.7   8.7  11.4   2.7   5.3
      5th |  5.3  19.5   9.5  34.5  10.7   4.9   5.2  10.4
      6th |  0.4   7.8  27.2  24.4  11.8   1.1  11.6  15.6
      7th |  0.0   1.5  37.3   6.8   7.7   0.3  24.5  22.0
    Worst |  0.0   0.1  17.5   0.8   4.2   0.0  50.6  26.8
MEAN RANK |  2.6   3.8   6.4   4.9   3.3   2.4   6.9   5.7
    SUCRA |  0.8   0.6   0.2   0.4   0.7   0.8   0.2   0.3
----------+-----------------------------------------------
Treatment Relative Ranking of Model 1


  +--------------------------------------+
  | Treatm~t | SUCRA | PrBest | MeanRank |
  |----------+-------+--------+----------|
  |       WL |  77.0 |   17.4 |      2.6 |
  |        I |  59.4 |    1.1 |      3.8 |
  |        G |  23.1 |    1.0 |      6.4 |
  |      gSH |  44.7 |    0.9 |      4.9 |
  |      uSH |  67.6 |   40.6 |      3.3 |
  |      TAU |  80.2 |   27.6 |      2.4 |
  |       PL |  15.2 |    2.0 |      6.9 |
  |       PP |  32.8 |    9.5 |      5.7 |
  +--------------------------------------
```

**Funnel plot**

Only 1 comparison with more than 10 studies: Individual-waiting list (13 studies).

```
Egger's test for small-study effects:
Regress standard normal deviate of intervention
effect estimate against its standard error

Number of studies =  13                                Root MSE      =   .4672
------------------------------------------------------------------------------
     Std_Eff |      Coef.   Std. Err.      t    P>|t|     [95% Conf. Interval]
-------------+----------------------------------------------------------------
       slope |   .2282293   .3134016     0.73   0.482     -.461563    .9180216
        bias |  -.0051252   .2931549    -0.02   0.986    -.6503548    .6401045
------------------------------------------------------------------------------

Test of H0: no small-study effects          P = 0.986


GRADE appraisal (CINeMA)
```

- Within-study bias: the “overall” risk of bias of each study was calculated as follows: (a) LOW risk if there “some concerns” on max two domains of the Cochrane RoB 2; (b) SOME CONCERNS if three domains were judged as having some concerns OR one domain was considered at high risk and two leaving some concerns; (c) HIGH RISK in all other cases (two or more high risk domains; three domains leaving some concerns + one high risk domain; 4 or more domains leaving some concerns).

For each comparison, the histogram was interpreted according to a “Average risk of bias” rule;

- Across-studies bias was considered “undetected” when was not possible to evaluate the risk of publication bias;
- Imprecision: risk ratio between 0.3 to 3.333 was considered as a clinically important size of effect;
- Heterogeneity: risk ratio between 0.3 to 3.333 was considered as a clinically important size of effect;
- Incoherence: for all the comparisons for which only a direct or indirect estimation was available (Inconsistency measures: Not applicable) we reported “some concern”.

Final report

| Comparison | Number of studies | Within-study bias | Reporting bias | Indirectness | Imprecision | Heterogeneity | Incoherence | Confidence rating |
| --- | --- | --- | --- | --- | --- | --- | --- | --- |
| face-to-face group:face-to-face individual | 2 | Some concerns | Low risk | No concerns | No concerns | No concerns | Some concerns | Moderate |
| face-to-face group:guided self-help | 1 | Some concerns | Low risk | No concerns | No concerns | No concerns | No concerns | Moderate |
| face-to-face group:unguided self-help | 1 | Some concerns | Low risk | No concerns | No concerns | No concerns | No concerns | Moderate |
| face-to-face group:waiting list | 7 | Some concerns | Low risk | No concerns | No concerns | No concerns | No concerns | Moderate |
| face-to-face individual:guided self-help | 2 | Some concerns | Low risk | No concerns | No concerns | No concerns | No concerns | Moderate |
| face-to-face individual:placebo pill | 4 | Some concerns | Low risk | No concerns | No concerns | No concerns | No concerns | Moderate |
| face-to-face individual:psychological placebo | 1 | Major concerns | Low risk | No concerns | Major concerns | No concerns | No concerns | Very low |
| face-to-face individual:treatment as usual | 5 | Some concerns | Low risk | No concerns | No concerns | No concerns | Some concerns | Moderate |
| face-to-face individual:unguided self-help | 2 | Some concerns | Low risk | No concerns | No concerns | No concerns | No concerns | Moderate |
| face-to-face individual:waiting list | 13 | Some concerns | Low risk | No concerns | No concerns | No concerns | No concerns | Moderate |
| guided self-help:treatment as usual | 2 | Some concerns | Low risk | No concerns | No concerns | No concerns | No concerns | Moderate |
| guided self-help:unguided self-help | 3 | Some concerns | Low risk | No concerns | No concerns | No concerns | Some concerns | Moderate |
| guided self-help:waiting list | 9 | Some concerns | Low risk | No concerns | No concerns | No concerns | No concerns | Moderate |
| placebo pill:waiting list | 1 | Some concerns | Low risk | No concerns | No concerns | No concerns | No concerns | Moderate |
| treatment as usual:unguided self-help | 2 | Some concerns | Low risk | No concerns | No concerns | No concerns | No concerns | Moderate |
| unguided self-help:waiting list | 7 | Some concerns | Low risk | No concerns | No concerns | No concerns | No concerns | Moderate |
| face-to-face group:placebo pill | 0 | Some concerns | Low risk | No concerns | No concerns | No concerns | No concerns | Moderate |
| face-to-face group:psychological placebo | 0 | Major concerns | Low risk | No concerns | Major concerns | No concerns | No concerns | Very low |
| face-to-face group:treatment as usual | 0 | Some concerns | Low risk | No concerns | No concerns | No concerns | No concerns | Moderate |
| guided self-help:placebo pill | 0 | Some concerns | Low risk | No concerns | No concerns | No concerns | No concerns | Moderate |
| guided self-help:psychological placebo | 0 | Some concerns | Low risk | No concerns | Major concerns | No concerns | No concerns | Low |
| placebo pill:psychological placebo | 0 | Major concerns | Low risk | No concerns | Major concerns | No concerns | No concerns | Very low |
| placebo pill:treatment as usual | 0 | Some concerns | Low risk | No concerns | No concerns | No concerns | No concerns | Moderate |
| placebo pill:unguided self-help | 0 | Some concerns | Low risk | No concerns | No concerns | No concerns | No concerns | Moderate |
| psychological placebo:treatment as usual | 0 | Major concerns | Low risk | No concerns | Major concerns | No concerns | No concerns | Very low |
| psychological placebo:unguided self-help | 0 | Some concerns | Low risk | No concerns | Major concerns | No concerns | No concerns | Low |
| psychological placebo:waiting list | 0 | Some concerns | Low risk | No concerns | Major concerns | No concerns | No concerns | Low |
| treatment as usual:waiting list | 0 | Some concerns | Low risk | No concerns | No concerns | No concerns | No concerns | Moderate |

**Supplement N - Sensitivity analyses**

| **Analysis** | **Forest plot** | Overall heterogeneity |
| --- | --- | --- |
| Excluding trials with an overall high risk of bias according to the RoB2 definition |  | SD=0.30; Restricted likelihood ratio test for heterogeneity: LRT = 14.51 (d.f. = 1) P = 0.000 |
|  |  | Overall incoherence |
|  |  | chi2(8) = 4.62  Prob > chi2 = 0.7977 |
|  |  | Loop-specific approach |
|  |  | 1/8 loops (B-E-F) showed inconsistency (P = 0.003) |
|  |  | Consistency between direct and indirect estimates |
|  |  | No inconsistencies |

| **Analysis** | **Forest plot** | Overall heterogeneity |
| --- | --- | --- |
| Excluding trials with imputed data |  | SD=0.37; Restricted likelihood ratio test for heterogeneity: LRT = 39.3 (d.f. = 1) P = 0.000 |

| **Analysis** | **Forest plot** | Overall heterogeneity |
| --- | --- | --- |
| Alternative outcome hierarchy (placing panic frequency/severity at the top of the hierarchy) |  | SD=0.39; Restricted likelihood ratio test for heterogeneity: LRT = 55.08 (d.f. = 1) P = 0.000 |
|  |  | Overall incoherence |
|  |  | chi2(8) = 9.64  Prob > chi2 = 0.9430 |
|  |  | Loop-specific approach |
|  |  | 3/15 loops (B-E-F /B-D-F /B-C-H) showed inconsistency (P < 0.05) |
|  |  | Consistency between direct and indirect estimates |
|  |  | No inconsistencies |
